# Toward a General Protocol for Catalytic Oxidative Transformations Using Electrochemically Generated Hypervalent Iodine Species

**DOI:** 10.1021/acs.joc.2c02309

**Published:** 2023-01-23

**Authors:** Mohamed Elsherbini, Wesley J. Moran

**Affiliations:** Department of Chemical Sciences, University of Huddersfield, Queensgate, Huddersfield HD1 3DH, U.K.

## Abstract

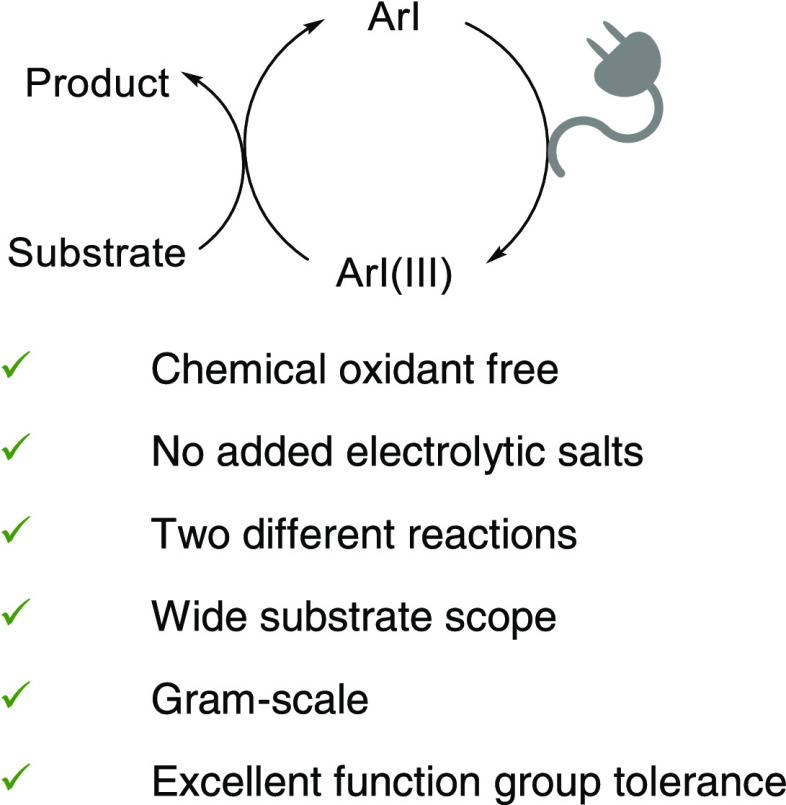

A simple catalytic electrosynthetic protocol for oxidative transformations mediated by hypervalent iodine reagents has been developed. In this protocol, electricity drives the iodine(I)/iodine(III) catalytic cycle enabling catalysis with *in situ* generated hypervalent iodine species, thereby eliminating chemical oxidants and the inevitable chemical waste associated with their mode of action. In addition, no added electrolytic salts are needed in this process. The developed method has been validated using two different hypervalent iodine-mediated transformations: (i) the oxidative cyclization of *N*-allylic and *N*-homoallylic amides to the corresponding dihydrooxazole and dihydro-1,3-oxazine derivatives, respectively, and (ii) the α-tosyloxylation of ketones. Both reactions proceeded smoothly under the developed catalytic electrosynthetic conditions without reoptimization, featuring a wide substrate scope and excellent functional group tolerance. In addition, scale-up to gram-scale and catalyst recovery were easily achieved maintaining the high efficiency of the process.

## Introduction

Hypervalent iodine reagents are readily available mild oxidants that are considered to be environmentally benign alternatives to metal-based oxidants and are widely used in modern organic synthesis.^1−5^ Their synthetic applications are tremendous and span a wide range of oxidative transformations such as oxidative cyclization/heterocyclization,^6−11^ difunctionalization of alkenes,^12−14^ phenol dearomatization,^15−17^ oxidation of sulfur compounds,^18,19^ α-functionalization of carbonyl compounds,^20−23^ and molecular rearrangement reactions.^24,25^ Hence, they are valuable tools in the synthetic organic chemistry toolbox.

**Scheme 1:**
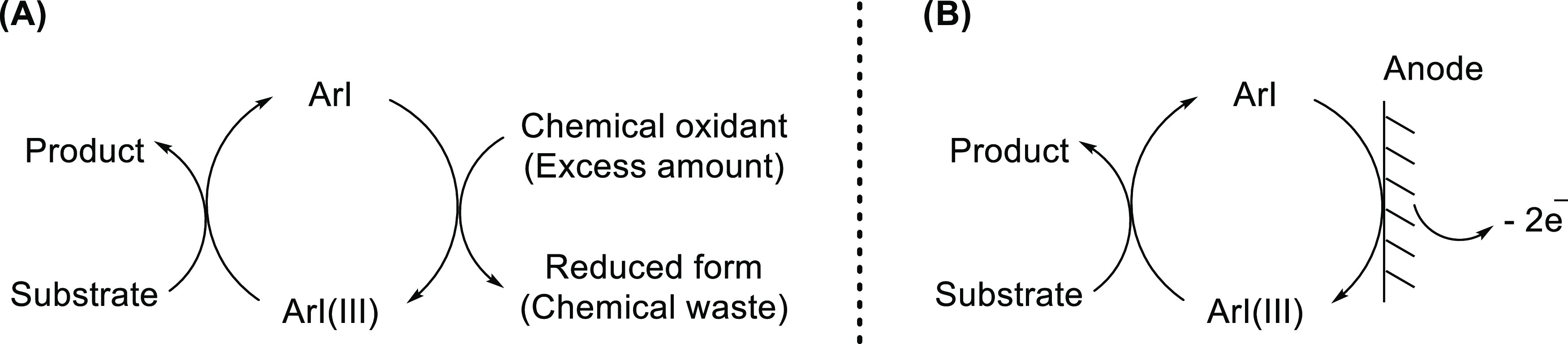
Catalytic Transformations *via* Iodine(I)/Iodine(III) Catalytic Cycle

One of the major advances in the long history of hypervalent iodine chemistry is the development of catalytic protocols relying on the iodine(I)/iodine(III) catalytic cycle.^26−32^ Although a wide range of efficient hypervalent iodine-mediated oxidative transformations under catalytic conditions has been reported, the protocol has intrinsic limitations and drawbacks. According to the general catalytic cycle (Scheme 1A),^31,32^ the use of hazardous and mostly expensive oxidants such as 3-chloroperbenzoic acid (*m*CPBA) and Selectfluor are required as terminal oxidants. These are typically required in excess quantities and the generation of chemical waste is inevitable. These problems could be addressed in principle by replacing chemical oxidants with traceless and relatively cheap electricity, where the pre-catalyst ArI is oxidized to the reactive λ^3^-iodane catalyst [ArI(III)] *via* anodic oxidation (Scheme 1B).

Herein, we report a simple catalytic electrosynthetic protocol for hypervalent iodine-mediated oxidative transformations that could form a basis for the development of a catalytic protocol applicable to a range of oxidative transformations.

## Results and Discussion

Despite the impressive developments in the chemistry of electrochemically generated hypervalent iodine reagents,^33−38^ the vast majority of the reported methods use stoichiometric amounts of iodine compounds^39−45^ and the development of catalytic protocols^46−48^ using iodine reagents as redox-active mediators is far behind. The reported catalytic methods using iodine compounds under electrolysis conditions are scarce and suffer from several limitations such as the necessity of large amounts of added electrolytes and narrow applicability. Therefore, we envisaged that the development of an efficient catalytic electrosynthetic method relying on the iodine(I)/iodine(III) catalytic cycle (Scheme 1B) is of considerable importance in this field of research. To achieve this goal, we selected the hypervalent iodine-mediated oxidative cyclization of *N*-allylamides to the corresponding oxazolidine derivatives—a reaction that is well studied in our laboratory—as a model reaction.^49−51^

We started our investigation with the electrolysis of the model substrate, *N*-allylbenzamide (**1a**) in the presence of 30 mol % of iodobenzene under conditions adopted from our recently published electrochemical synthesis of diaryliodonium salts.^52^ Under these conditions (Table 1, entry 1), the starting material was left unchanged at the end of the electrolysis. Replacing TfOH by BF_3_·Et_2_O (1 equiv) led to degradation of the starting material without observation of the desired product (Table 1, entry 2). Performing the electrolysis in the absence of acid (Table 1, entry 3) led to unreacted starting material. In view of various reports on the successful electrochemical oxidation of iodoarenes to the corresponding hypervalent iodine reagents under similar conditions, these negative outcomes (entries 1–2) could be attributed to the relative instability of the generated hypervalent iodine species under these conditions. While the unsuccessful cyclization in the absence of acid (entry 3) is in accordance with previous reports on the necessity of an acid for this transformation.^39,49,50^ Therefore, generation of a more stable species such as Koser’s reagent in acidic medium could alleviate this problem. Indeed, using two equivalents of *p*-toluenesulfonic acid monohydrate (TsOH·H_2_O) led to the formation of the desired product **2a** in 69% yield after passing 2.5 F (Table 1, entry 4). Increasing the charge from 2.5 to 3.0 F (Table 1, entry 5) led to improvement of the yield to 80%. The yield was improved further to 92% by increasing the amount of TsOH·H_2_O to 2.5 equivalents (Table 1, entry 6). Further increase of the equivalents of tosylic acid or the passed charge (Table 1, entries 7 and 8) did not lead to a significant improvement of the reaction outcome. Applying conditions of entry 6 but changing the anode material to graphite instead of glassy carbon led to a similar outcome, where **2a** was formed in 93% yield (Table 1, entry 9). On the other hand, changing the anode material from carbon to platinum led to a significant drop of the yield to 67% (Table 1, entry 10). Also, increasing the current from 5 to 10 mA, *i.e.*, cutting the reaction time in half (Table 1, entry 11), had a negative impact on the reaction outcome leading to the formation of **2a** in a lower yield (77%). Decreasing the catalyst (PhI) loading from 30 to 25 mol % (Table 1, entry 12) led to decrease of the yield from 93 to 80%. At 25 mol % of iodobenzene, the high reaction outcome could be regained by increasing the charge to 4.0 F (Table 1, entry 13). Decreasing the catalyst loading further to 20 mol % while keeping the charge at 4.0 F led to the formation of **2a** in 81% yield (Table 1, entry 14). Finally, increasing the concentration of the starting material from 0.06 to 0.1 M did not negatively impact the reaction outcome and **2a** was formed in 91% yield (Table 1, entry 15). Therefore, conditions of entry 9 were chosen as the optimum conditions.

**Table 1:**
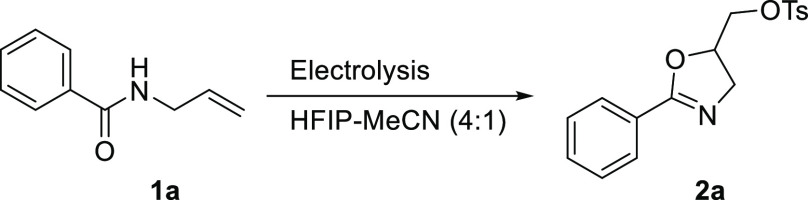
Optimization of Catalytic Electrosynthetic Oxidative Cyclization of *N*-Allyamide **1a**^a^

no	PhI (mol %)	acid (equiv)	(+), (−)	current (mA)	charge (F)	yield%^b^
1	30	TfOH (5.0)	GC, Pt	5	2.5	0
2^c^	30	BF_3_·Et_2_O (1.0)	GC, Pt	5	2.5	0
3^c^	30	none	GC, Pt	5	2.5	0
4	30	TsOH·H_2_O (2.0)	GC, Pt	5	2.5	69
5	30	TsOH·H_2_O (2.0)	GC, Pt	5	3.0	80
6	30	TsOH·H_2_O (2.5)	GC, Pt	5	3.0	92
7	30	TsOH·H_2_O (3.0)	GC, Pt	5	3.0	94
8	30	TsOH·H_2_O (2.5)	GC, Pt	5	3.5	94
9	30	TsOH·H_2_O (2.5)	C, Pt	5	3.0	93
10	30	TsOH·H_2_O (2.5)	Pt, Pt	5	3.0	67
11	30	TsOH·H_2_O (2.5)	C, Pt	10	3.0	77
12	25	TsOH·H_2_O (2.5)	C, Pt	5	3.0	80
13	25	TsOH·H_2_O (2.5)	C, Pt	5	4.0	94
14	20	TsOH·H_2_O (2.5)	C, Pt	5	4.0	81
15^d^	30	TsOH·H_2_O (2.5)	C, Pt	5	3.0	91

a Electrolyses were carried out under ambient conditions with Electrasyn 2.0, using a 5 mL glass vial equipped with two electrodes; electrode immersed area: 2.8 cm^2^; **1a** (0.3 mmol) dissolved in a mixture of HFIP and MeCN (4:1, 5 mL, 0.06 M).

b Determined by ^1^H NMR using PhNO_2_ as internal standard.

c Et_4_NBF_4_ (0.5 mmol, 0.1 M) was used as supporting electrolyte.

d **1a** (0.5 mmol, 0.1 M). GC = glassy carbon; C = graphite; HFIP = 1,1,1,3,3,3-hexafluoro-2-propanol.

**Scheme 2:**
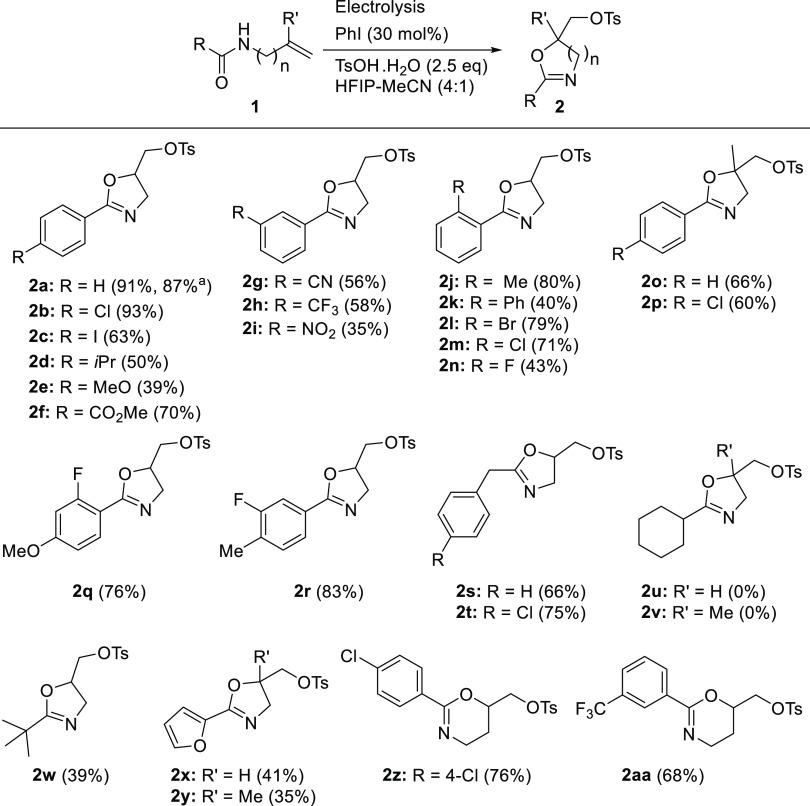
Substrate Scope of Catalytic Cyclization of Allyl and Homoallyl Amides

To study the scope of substrates, a wide range of *N*-allylamides was synthesized and electrolyzed under the optimized reaction conditions (Table 1, entry 9). The results summarized in Scheme 2 revealed that most of the studied substrates were successfully converted to the corresponding cyclized products in good to excellent yields, showing excellent functional group tolerance. Under the optimized reaction conditions, (2-phenyl-4,5-dihydrooxazol-5-yl)methyl 4-methylbenzenesulfonate (**2a**) was isolated in 91% yield starting from *N*-allylbenzamide (**1a**). The developed catalytic electrosynthetic method proved to be easily scalable, where the electrolysis of 10 mmol of **1a** under the same conditions but using a homemade beaker-type cell (see the SI for details) led to isolation of 2.9 g of **2a** (87%). Furthermore, iodobenzene catalyst was easily recovered from the gram-scale experiment.

Various *para*-substituted substrates (**1b–f**) were successfully converted to the corresponding dihydrooxazole derivatives (**2b–f**) in moderate to excellent yields. The *p*-chloro derivative **2b** was obtained in 93% yield. Noteworthy, the 4-iodo substituent was also tolerated and the corresponding iodo-substituted product **2c** was obtained in 63% yield. Electrolysis of electron-rich substrates with isopropyl and methoxy substituents was also successful and led to the formation of the desired products **2d** and **2e**, albeit in lower yields, 50 and 39%, respectively, mostly, due to the easy oxidation of the aromatic ring of electron-rich arenes.^52^ On the other hand, an ester group at the *para*-position was tolerated and led to the desired product **2f** in 70% yield.

Substrates with electron-withdrawing groups at the *meta*-position (**1g-i**) were also studied. The outcome varied, where cyano- and trifluoromethyl-substituted products **2g** and **2h** were formed in good yields, 56 and 58% yields, respectively. While the nitro-substituted product **2i** was formed in lower yield (35%), showing that under these conditions, the CN and CF_3_ groups were tolerated better than the NO_2_ group.

Five substrates with ortho substituents (**1j–n**) were also electrolyzed under the optimized conditions. The desired products (**2j–n**) were all isolated in good yields ranging from 40 to 80%. The products were formed as mixtures of diastereoisomers in all cases except the 2-fluorosubstituted product **2n**, which lacked an axis of chirality due to the small size of the ortho-substituent. The 2-methyl-substituted product **2j** was formed in high yield (80%) with a 5:1 diastereomeric ratio. The lowest yield in this subgroup of substrates was observed for the 2-phenyl-substituted substrate **1k** that led to the corresponding dihydrooxazole product **2k** in 40% yield and 2.3:1 diastereomeric ratio. The 2-halo-substituted products **2l** and **2m** were formed in very good yields, 79% (2-Br) and 71% (2-Cl) respectively, and similar dr 2.2:1 and 2.5:1, respectively.

Substrates containing an alkene moiety with a methyl substituent (**1o,p**) also performed well under the developed catalytic electrosynthetic conditions and led to the corresponding dihydrooxazole products **2o** and **2p** containing a quaternary carbon in good yields, 66 and 60%, respectively. Substrates with two substituents in the aromatic ring (**1q,r**), also cyclized smoothly giving the corresponding products (**2q,r**) in high yields, 76 and 83%, respectively. Noteworthy, the introduction of fluorine substituent in addition to electron-donating substituents in substrates **1q** and **1r** led to better performance compared to electron-rich substrates **1d** and **1e**. In addition, the easily oxidized benzylic CH_2_ position was well tolerated under the developed catalytic electrosynthetic conditions; substrates **1s** and **1t** with benzyl derivatives underwent clean cyclization leading to the corresponding product **2s** and **2t** in very good yields, 66 and 75%, respectively.

On the other hand, moving from aromatic substrate to aliphatic substrates revealed the limitations of the developed method.^50^ Substrates **1u** and **1v** containing cyclohexyl moiety attached to the amide carbonyl were totally unreactive and were recovered unchanged at the end of the reaction without observation of the desired products **2u** and **2v**. The same result was observed when the cyclohexyl moiety was changed to cyclobutyl and cyclopropyl, i.e., no reaction. But substrate **1w** with a *t*-Bu substituent was reactive under these conditions and the corresponding *t*-Bu-substituted dihydrooxazole product **2w** was isolated in 39% yield. A similar outcome was observed for substrates **1x** and **1y** containing a furan moiety,^52−54^ where the cyclized products **2x** and **2y** were obtained in 41 and 35% yields, respectively. Finally, using homoallylic *N*-amide substrates **1z** and **1aa** led to the six-membered dihydro-2*H*-pyran products **2z** and **2aa** in 76 and 68% yields, respectively.

**Scheme 3:**
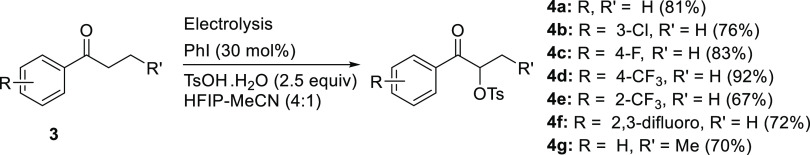
Substrate Scope of Catalytic Electrosynthetic α-Tosyloxylation of Ketones

One of the main objectives of this research was to develop a catalytic electrosynthetic method that can be successfully applied to more than one chemical transformation and has the potential for generalization in the field of oxidative transformations mediated by electrochemically generated hypervalent iodine reagents. Therefore, the same conditions (Table 1, entry 9) were applied to another well-studied hypervalent iodine-mediated transformation, the α-tosyloxylation of ketones.^22,24,55^ Delightfully, the results showed that the developed catalytic electrosynthetic conditions were feasible for this reaction as well, without any further optimization (Scheme 3). The desired products **4a–g** were formed in good to excellent yields ranging between 67 and 92% without any change in the conditions that were optimized initially for a different reaction.

**Scheme 4:**
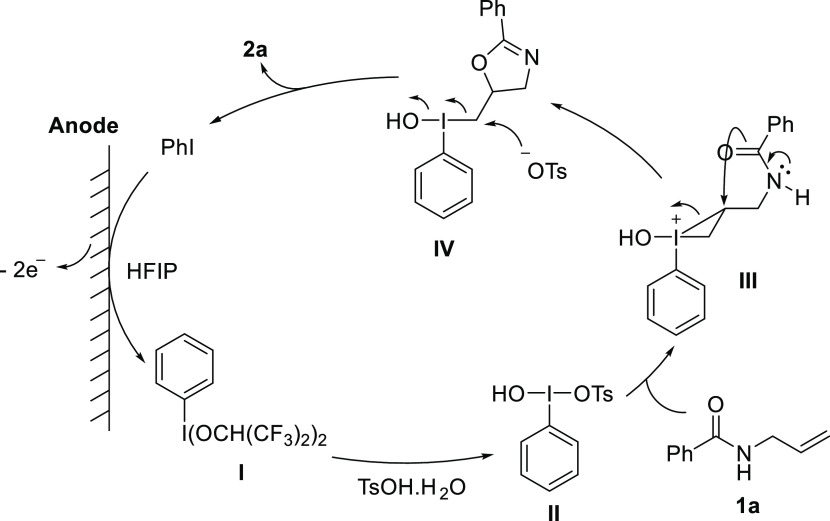
Proposed Reaction Mechanism for the Electrochemical Cyclization

Similar to the cyclization process, in the absence of acid (cf. Table 1, entry 3), the α-tosyloxylation reaction did not proceed,^49,50^ and the starting material was recovered unchanged. The same outcome, no reaction, was also observed in the absence of iodobenzene or electricity. In view of the results of these control experiments and the previously published studies on the mechanisms of hypervalent iodine-mediated *N*-allylamide cyclization and α-tosyloxylation of ketones and catalytic transformations with electrochemically generated hypervalent iodine reagents, a proposed reaction mechanism is presented for the cyclization (Scheme 4).^24,47−50,55,60^ Initially, iodobenzene is anodically oxidized in the presence of HFIP to the corresponding hypervalent iodine species **I** that undergoes ligand exchange with tosylic acid to form the more stable Koser’s reagent (**II**). This activates the double bond of substrate **1a** forming species **III** that undergoes intramolecular cyclization to form the dihydrooxazole core in species **IV**. Finally, reductive elimination leads to the desired product **2a** and regeneration of iodobenzene.

## Conclusions

In conclusion, a simple catalytic electrosynthetic protocol for hypervalent iodine-mediated oxidative transformations has been developed. In this method, no added electrolytic salts were needed, hazardous and expensive terminal chemical oxidants and their accompanying chemical waste were eliminated, and the iodine(I)/iodine(III) catalytic cycle was driven by cheap traceless electricity. The developed catalytic electrosynthetic protocol was optimized initially by studying the hypervalent iodine-mediated oxidative cyclization of *N*-allyl and *N*-homoallyl amides to the corresponding dihydrooxazole and dihydro-1,3-oxazine derivatives, respectively. Under the optimized reaction conditions, the reaction proceeded smoothly for a wide range of substrates leading to the desired product in very good yields on average and excellent functional group tolerance. The cyclization of *N*-allybenzamide to the corresponding dihydrooxazole derivative was easily scaled up to gram-scale without problems and the catalyst was easily recovered. In addition, the same catalytic electrosynthetic protocol was applied successfully to the α-tosyloxylation of ketones; another hypervalent iodine-mediated oxidative transformation. In this case, the reaction proceeded smoothly giving the desired products in very good yields without reoptimization or change of the conditions optimized initially for a different transformation. The catalytic electrosynthetic conditions reported herein could form a basis toward achieving a general catalytic protocol suitable for a range of oxidative transformations mediated by hypervalent iodine reagents. Application of the developed catalytic electrosynthetic protocol to other hypervalent iodine-mediated oxidative transformations in addition to the development of an enantioselective version is underway in our laboratory.

## Experimental Section

### General

Chemicals were purchased from Sigma-Aldrich, Alfa Aesar, and Fluorochem and were used as received without purification or drying. Solvents were used as received without drying. Thin-layer chromatography (TLC) was performed on precoated aluminum sheets of Merck silica gel 60 F254 (0.20 mm) and visualized by UV radiation (254 nm). Automated column chromatography was performed on a Biotage Isolera Four using Biotage SNAP Ultra cartridges. ^1^H NMR and ^13^C NMR spectra were measured on a Bruker Ascend 400 or 600 apparatus and were referenced to the solvent peak. Chemical shifts δ were given in ppm, and the multiplicity of the signals was reported as: s = singlet, s_br_ = broad singlet, d = doublet, t = triplet, q = quartet, sept = septet, dd = doublet of doublets, dt = doublet of triplets, dq = doublet of quartets, qd = quartet of doublets, m = multiplet. The coupling constants (*J*) in hertz. Mass spectrometric measurements were performed at Innovative Physical Organic Solutions (IPOS), University of Huddersfield on Agilent 1290 HPLC + 6530 QTOF instrument. Ions were generated by electrospray ionization (ESI), and only the mass ions are reported. Spectral data for previously reported compounds are in good agreement with literature: **1a**,^56^**1b**,^50^**1c**,^56^**1d**,^56^**1e**,^56^**1f**,^57^**1i**,^58^**1j**,^59^**1k**,^60^**1l**,^60^**1m**,^60^**1n**,^61^**1o**,^58^**1p**,^58^**1s**,^62^**1u**,^58^**1v**,^63^**1w**,^51^**1x**,^51^**1z**,^50^**4a**,^22^**4b**,^22^**4c**,^64^**4d**,^65^**4e**,^64^**4g**.^22^ Ketones **3a–g** were all purchased from Fluorochem and were used as received.

### Synthesis of *N*-Allyl/Homoallyl Amide Substrates **1**

To a 100 mL round-bottom flask were added the appropriate carboxylic acid (5 mmol, 1 equiv), dry DCM (20 mL), and a catalytic amount of DMF (2 drops), cooled to 0 °C with an ice bath, and stirred for 5 min. Oxalyl chloride (1.3 equiv) was then added dropwise at 0 °C under N_2_. Stirring was continued at RT overnight and then evaporated under vacuum. The resulting acid chloride was dissolved in dry DCM (5 mL) and added dropwise under N_2_ to a flask containing a mixture of appropriate amine derivative (5 mmol, 1 equiv) and Et_3_N (2.2 equiv) in dry DCM (10 mL). Stirring was continued overnight at RT under N_2_. After completion of the reaction, an aqueous solution of NaOH (1 M, 10 mL) was added, and the mixture was extracted with DCM (3×). The combined organic layers were washed with water (1×) and brine (1×), then dried over anhydrous magnesium sulfate, filtered, and concentrated under reduced pressure. The crude product was purified by flash chromatography using Biotage Isolera Four, applying ethyl acetate/pet. ether 4–40% gradient.

#### N-Allyl-3-cyanobenzamide (**1g**)

White solid (840 mg, 90%). Purified by flash column chromatography (ethyl acetate/hexane 4–40% gradient). ^1^H NMR (400 MHz, CDCl_3_) δ = 8.09 (s, 1H), 8.03 (d, *J* = 7.9 Hz, 1H), 7.77 (d, *J* = 7.7 Hz, 1H), 7.57 (t, *J* = 7.8 Hz, 1H), 6.53 (s, 1H), 5.92 (dq, *J* = 10.7, 5.7 Hz, 1H), 5.26 (d, *J* = 17.1 Hz, 1H), 5.20 (d, *J* = 10.2 Hz, 1H), 4.08 (t, *J* = 5.6 Hz, 2H) ppm. ^13^C{^1^H} NMR (101 MHz, CDCl_3_) δ = 165.3, 135.8, 134.8, 133.7, 131.5, 130.9, 129.7, 118.1, 117.3, 113.0, 42.8 ppm.

#### N-Allyl-3-(trifluoromethyl)benzamide (**1h**)

White solid (885 mg, 77%). Purified by flash column chromatography (ethyl acetate/hexane 4–40% gradient). ^1^H NMR (400 MHz, CDCl_3_) δ = 8.04 (s, 1H), 7.97 (d, *J* = 7.8 Hz, 1H), 7.75 (d, *J* = 7.8 Hz, 1H), 7.56 (t, *J* = 7.8 Hz, 1H), 6.47 (s, 1H), 5.93 (ddt, *J* = 16.0, 10.3, 5.7 Hz, 1H), 5.26 (dd, *J* = 17.1, 1.4 Hz, 1H), 5.19 (dd, *J* = 10.2, 1.3 Hz, 1H), 4.09 (tt, *J* = 5.8, 1.4 Hz, 2H) ppm. ^13^C{^1^H} NMR (101 MHz, CDCl_3_) δ = 166.1, 135.4, 133.9, 131.3 (q, ^2^*J*_C-F_ = 32.9 Hz), 130.4 (d, ^4^*J*_C-F_ = 1.0 Hz), 129.4, 128.23 (q, ^3^*J*_C-F_ = 3.7 Hz), 124.1 (q, ^3^*J*_C-F_ = 3.8 Hz), 123.8 (q, ^1^*J*_C-F_ = 272.6 Hz), 117.2, 42.8 ppm. ^19^F NMR (376 MHz, CDCl_3_) δ = −62.76 ppm.

#### N-Allyl-2-fluoro-4-methoxybenzamide (**1q**)

White solid (980 mg, 94%). Purified by flash column chromatography (ethyl acetate/hexane 4–40% gradient). ^1^H NMR (400 MHz, CDCl_3_) δ 8.04 (t, *J* = 9.1 Hz, 1H), 6.76 (dd, *J* = 8.8, 2.4 Hz, 1H), 6.74 (s, 1H), 6.60 (dd, *J* = 14.1, 2.4 Hz, 1H), 5.92 (ddd, *J* = 22.6, 10.7, 5.5 Hz, 1H), 5.24 (dd, *J* = 17.2, 1.3 Hz, 1H), 5.15 (dd, *J* = 10.3, 1.1 Hz, 1H), 4.11–4.05 (m, 2H), 3.82 (s, 3H) ppm. ^13^C{^1^H} NMR (101 MHz, CDCl_3_) δ 163.6 (d, ^3^*J*_C-F_ = 2.4 Hz), 163.1 (d, ^3^*J*_C-F_ = 3.6 Hz), 161.8 (d, ^1^*J*_C-F_ = 246.5 Hz), 134.3, 133.3 (d, ^3^*J*_C-F_ = 4.2 Hz), 116.4, 113.38 (d, ^2^*J*_C-F_ = 11.9 Hz), 110.7 (d, ^4^*J*_C-F_ = 2.5 Hz), 101.6 (d, ^2^*J*_C-F_ = 28.8 Hz), 55.9, 42.3 ppm. ^19^F NMR (376 MHz, CDCl_3_) δ −111.00 ppm.

#### N-Allyl-3-fluoro-4-methylbenzamide (**1r**)

White solid (890 mg, 92%). Purified by flash column chromatography (ethyl acetate/hexane 4–40% gradient). ^1^H NMR (400 MHz, CDCl_3_) δ 7.48–7.41 (m, 2H), 7.21 (t, *J* = 7.7 Hz, 1H), 6.41 (s, 1H), 5.91 (ddd, *J* = 16.0, 10.8, 5.7 Hz, 1H), 5.24 (dd, *J* = 7.1, 1.3 Hz, 1H), 5.16 (dd, *J* = 10.2, 1.2 Hz, 1H), 4.05 (t, *J* = 5.7 Hz, 2H), 2.30 (d, *J* = 1.5 Hz, 3H) ppm. ^13^C{^1^H} NMR (101 MHz, CDCl_3_) δ 166.3 (d, ^4^*J*_C-F_ = 2.3 Hz), 161.3 (d, ^1^*J*_C-F_ = 246.2 Hz), 134.1 (2C), 131.6 (d, ^3^*J*_C-F_ = 5.1 Hz), 128.9 (d, ^2^*J*_C-F_ = 17.4 Hz), 122.2 (d, ^4^*J*_C-F_ = 3.5 Hz), 116.8, 114.1 (d, ^2^*J*_C-F_ = 23.9 Hz), 42.6, 14.7 (d, ^3^*J*_C-F_ = 3.5 Hz) ppm. ^19^F NMR (376 MHz, CDCl_3_) δ −116.29 ppm.

#### N-(2-Methylallyl)furan-2-carboxamide (**1y**)

Yellow oil (460 mg, 93%). Purified by flash column chromatography (ethyl acetate/hexane 4–40% gradient). ^1^H NMR (400 MHz, CDCl_3_) δ 7.43 (d, *J* = 0.9 Hz, 1H), 7.11 (d, *J* = 3.4 Hz, 1H), 6.49 (dd, *J* = 3.4, 1.7 Hz, 1H), 6.49 (s, 1H), 4.88 (d, *J* = 11.5 Hz, 2H), 3.97 (d, *J* = 6.1 Hz, 2H), 1.77 (s, 3H) ppm. ^13^C{^1^H} NMR (101 MHz, CDCl_3_) δ 158.4, 148.1, 144.0, 141.9, 114.4, 112.3, 111.4, 44.7, 20.5 ppm.

#### N-(But-3-en-1-yl)-3-(trifluoromethyl)benzamide (**1aa**)

Colorless oil (490 mg, 40%). Purified by flash column chromatography (ethyl acetate/hexane 4–40% gradient). ^1^H NMR (400 MHz, CDCl_3_) δ = 8.00 (s, 1H), 7.91 (d, *J* = 7.8 Hz, 1H), 7.73 (d, *J* = 7.8 Hz, 1H), 7.54 (t, *J* = 7.8 Hz, 1H), 6.44 (s, 1H), 5.82 (ddt, *J* = 17.1, 10.2, 6.8 Hz, 1H), 5.20–5.07 (m, 2H), 3.53 (dd, *J* = 12.5, 6.7 Hz, 2H), 2.42–2.35 (m, 2H) ppm. ^13^C{^1^H} NMR (101 MHz, CDCl_3_) δ = 166.3, 135.7, 135.2, 131.2 (q, ^2^*J*_C-F_ = 32.8 Hz), 130.2 (d, ^4^*J*_C-F_ = 0.9 Hz), 129.3, 128.1 (q, ^3^*J*_C-F_ = 3.7 Hz), 124.0 (q, ^3^*J*_C-F_ = 3.9 Hz), 123.8 (q, ^1^*J*_C-F_ = 272.5 Hz), 117.7, 39.2, 33.8 ppm. ^19^F NMR (376 MHz, CDCl_3_) δ = −62.79 ppm.

### Catalytic Electrosynthetic Oxidative Cyclization of *N*-Allyl/Homoallyl Amides **1**

A solution of substrate **1** (0.3 mmol, 1 equiv) and tosylic acid (0.75 mmol, 2.5 equiv) in a mixture of HFIP (4 mL) and acetonitrile (1 mL) containing iodobenzene (18.4 mg, 10 μL, 0.09 mmol, 0.3 equiv) was electrolyzed using an ElectraSyn undivided cell (5 mL glass vial) equipped with graphite anode and platinum cathode under constant current of 5 mA with stirring (400 rpm) for 4.82 h (3.0 F). After electrolysis, the electrodes were rinsed with DCM, combined with the reaction mixture, then treated with sat. aq. Na_2_S_2_O_3_ solution (5 mL) and sat. aq. NaHCO_3_ solution (5 mL), and diluted with DCM (10 mL). The phases were separated, and the aqueous layer was extracted with DCM (2×). The combined organic layers were washed with water (1×) and brine (1×), then dried over anhydrous magnesium sulfate, filtered, and concentrated under reduced pressure. The crude product was purified by flash chromatography using Biotage Isolera Four, applying ethyl acetate/pet. ether 12–100% gradient.

### Gram-Scale Electrochemical Synthesis of **2a**

Using a beaker-type homemade electrolysis cell (Figure S2), a solution of amide **1a** (1.61 g, 10 mmol, 1.0 equiv) and tosylic acid (4.76 g, 25 mmol, 2.5 equiv) in a mixture of HFIP (133 mL) and acetonitrile (33 mL) containing iodobenzene (612 mg, 0.34 mL, 3 mmol, 0.3 equiv) was electrolyzed with stirring (400 rpm) under constant current of 92 mA (46 mA on each of the two anodes, *j* = 1.84 mA/cm^2^) for 8.74 h (3.0 F). After electrolysis, the electrodes were rinsed with DCM and combined with the reaction mixture, then treated with sat. aq. Na_2_S_2_O_3_ solution (50 mL) and sat. aq. NaHCO_3_ solution (50 mL), and diluted with DCM (100 mL). The phases were separated, and the aqueous layer was extracted with DCM (2×) The combined organic layers were washed with water (1×) and brine (1×), then dried over anhydrous magnesium sulfate, filtered, and concentrated under reduced pressure. The crude product was purified by flash chromatography using Biotage Isolera Four, applying ethyl acetate/pet. ether 12–100% gradient to give pure **2a** as a pale-yellow solid (2.9 g, 87% yield). In addition, 550 mg of iodobenzene was recovered (90% recovery) from the same column (early fraction at 12% EtOAc/pet. ether).

#### (2-Phenyl-4,5-dihydrooxazol-5-yl)methyl 4-methylbenzenesulfonate (**2a**)

Pale-yellow solid (90 mg, 91%). M.p. 127–128 °C. Purified by flash column chromatography (ethyl acetate/hexane 12–100% gradient). ^1^H NMR (400 MHz, CDCl_3_) δ 7.74–7.69 (m, 2H), 7.66 (d, *J* = 8.3 Hz, 2H), 7.37 (t, *J* = 7.4 Hz, 1H), 7.27 (t, *J* = 7.5 Hz, 2H), 7.16 (d, *J* = 8.6 Hz, 2H), 4.77 (tdd, *J* = 6.9, 6.1, 4.0 Hz, 1H), 4.08 (dd, *J* = 11.0, 3.9 Hz, 1H), 4.05–3.95 (m, 2H), 3.67 (dd, *J* = 15.1, 7.1 Hz, 2H), 2.29 (s, 3H) ppm. ^13^C{^1^H} NMR (101 MHz, CDCl_3_) δ 163.8, 145.2, 132.7, 131.7, 130.1, 128.4, 128.3, 128.1, 127.2, 76.3, 70.1, 56.9, 21.8 ppm. HRMS (ESI) Calcd for C_17_H_18_NO_4_S^+^ [M + H]^+^ 332.0951, found 332.0963.

#### (2-(4-Chlorophenyl)-4,5-dihydrooxazol-5-yl)methyl 4-methylbenzenesulfonate (**2b**)

Pale-yellow solid (102 mg, 93%). M.p. 112–113 °C. Purified by flash column chromatography (ethyl acetate/hexane 12–100% gradient). ^1^H NMR (400 MHz, CDCl_3_) δ 7.77 (d, *J* = 8.3 Hz, 2H), 7.76 (d, *J* = 8.6 Hz, 2H), 7.36 (d, *J* = 8.4 Hz, 2H), 7.28 (d, *J* = 8.1 Hz, 2H), 4.87 (ddd, *J* = 10.1, 8.3, 5.4 Hz, 1H), 4.21 (dd, *J* = 11.0, 3.8 Hz, 1H), 4.16–4.06 (m, 2H), 3.78 (dd, *J* = 15.1, 7.1 Hz, 1H), 2.41 (s, 3H) ppm. ^13^C{^1^H} NMR (101 MHz, CDCl_3_) δ = 163.0, 145.3, 137.9, 132.8, 130.07, 129.7, 128.8, 128.1, 125.7, 76.6, 70.0, 56.9, 21.8 ppm. HRMS (ESI) Calcd for C_17_H_17_NClO_4_S^+^ [M + H]^+^ 366.0561, found 366.0571.

#### (2-(4-Iodophenyl)-4,5-dihydrooxazol-5-yl)methyl 4-methylbenzenesulfonate (**2c**)

White solid (86 mg, 63%). M.p. 129–130 °C. Purified by flash column chromatography (ethyl acetate/hexane 12–100% gradient). ^1^H NMR (400 MHz, CDCl_3_) δ 7.79–7.69 (m, 4H), 7.57–7.48 (m, 2H), 7.28 (d, *J* = 8.0 Hz, 2H), 4.92–4.83 (m, 1H), 4.20 (dd, *J* = 11.1, 3.8 Hz, 1H), 4.13 (dd, *J* = 8.8, 3.6 Hz, 1H), 4.08 (dd, *J* = 12.9, 7.8 Hz, 1H), 3.77 (dd, *J* = 15.2, 7.1 Hz, 1H), 2.41 (s, 3H) ppm. ^13^C{^1^H} NMR (101 MHz, CDCl_3_) δ 163.2, 145.3, 137.7, 132.7, 130.1, 129.8, 128.0, 126.7, 98.7, 76.5, 70.0, 56.9, 21.8 ppm. HRMS (ESI) Calcd for C_17_H_17_INO_4_S^+^ [M + H]^+^ 457.9917, found 457.9924.

#### (2-(4-Isopropylphenyl)-4,5-dihydrooxazol-5-yl)methyl 4-methylbenzenesulfonate (**2d**)

White solid (56 mg, 50%). M.p. 112–113 °C. Purified by flash column chromatography (ethyl acetate/hexane 12–100% gradient). ^1^H NMR (400 MHz, CDCl_3_) δ 7.80–7.72 (m, 4H), 7.28 (d, *J* = 8.0 Hz, 2H), 7.24 (d, *J* = 8.2 Hz, 2H), 4.90–4.81 (m, 1H), 4.17 (dd, *J* = 10.9, 4.1 Hz, 1H), 4.12 (dd, *J* = 9.1, 5.0 Hz, 1H), 4.08 (dd, *J* = 11.5, 6.5 Hz, 1H), 3.76 (dd, *J* = 15.0, 7.0 Hz, 1H), 2.94 (sept, *J* = 6.9 Hz, 1H), 2.40 (s, 3H), 1.26 (d, *J* = 6.9 Hz, 6H) ppm. ^13^C{^1^H} NMR (101 MHz, CDCl_3_) δ 163.9, 152.9, 145.2, 132.7, 130.1, 128.4, 128.1, 126.5, 124.8, 76.2, 70.1, 56.9, 34.3, 23.9, 21.8 ppm. HRMS (ESI) Calcd for C_20_H_24_NO_4_S^+^ [M + H]^+^ 374.1421, found 374.1431.

#### (2-(4-Methoxyphenyl)-4,5-dihydrooxazol-5-yl)methyl 4-methylbenzenesulfonate (**2e**)

White solid (42 mg, 39%). M.p. 105–106 °C. Purified by flash column chromatography (ethyl acetate/hexane 12–100% gradient). ^1^H NMR (400 MHz, CDCl_3_) δ 7.77 (d, *J* = 8.1 Hz, 2H), 7.76 (d, *J* = 8.8 Hz, 2H), 7.29 (d, *J* = 8.2 Hz, 2H), 6.88 (d, *J* = 8.8 Hz, 2H), 4.84 (ddd, *J* = 10.3, 8.4, 5.3 Hz, 1H), 4.19–4.03 (m, 3H), 3.84 (s, 3H), 3.73 (dd, *J* = 14.9, 7.0 Hz, 1H), 2.41 (s, 3H) ppm. ^13^C{^1^H} NMR (101 MHz, CDCl_3_) δ 163.6, 162.3, 145.2, 132.8, 130.1 (2C), 128.1, 119.7, 113.8, 76.2, 70.2, 56.9, 55.5, 21.8 ppm. HRMS (ESI) Calcd for C_18_H_20_NO_5_S^+^ [M + H]^+^ 362.1057, found 362.1069.

#### Methyl 4-(5-((tosyloxy)methyl)-4,5-dihydrooxazol-2-yl)benzoate (**2f**)

Pale-yellow solid (82 mg, 70%). M.p. 163–164 °C. Purified by flash column chromatography (ethyl acetate/hexane 12–100% gradient). ^1^H NMR (400 MHz, CDCl_3_) δ 8.05 (d, *J* = 8.6 Hz, 2H), 7.88 (d, *J* = 8.6 Hz, 2H), 7.76 (d, *J* = 8.3 Hz, 2H), 7.27 (d, *J* = 8.0 Hz, 2H), 4.95–4.84 (m, 1H), 4.22 (dd, *J* = 11.1, 3.8 Hz, 1H), 4.18–4.07 (m, 2H), 3.93 (s, 3H), 3.82 (dd, *J* = 15.3, 7.2 Hz, 1H), 2.39 (s, 3H) ppm. ^13^C{^1^H} NMR (101 MHz, CDCl_3_) δ 166.5, 163.1, 145.3, 132.8, 132.7, 131.2, 130.1, 129.6, 128.3, 128.0, 76.6, 69.9, 57.0, 52.5, 21.8 ppm. HRMS (ESI) Calcd for C_19_H_20_NO_6_S^+^ [M + H]^+^ 390.1006, found 390.1014.

#### (2-(3-Cyanophenyl)-4,5-dihydrooxazol-5-yl)methyl 4-methylbenzenesulfonate (**2g**)

White solid (60 mg, 56%). M.p. 106–107 °C. Purified by flash column chromatography (ethyl acetate/hexane 12–100% gradient). ^1^H NMR (400 MHz, CDCl_3_) δ 8.12–8.04 (m, 2H), 7.80–7.72 (m, 3H), 7.52 (td, *J* = 7.8, 0.6 Hz, 1H), 7.31 (dd, *J* = 8.5, 0.5 Hz, 2H), 4.92 (dddd, *J* = 10.7, 7.2, 5.6, 3.6 Hz, 1H), 4.24 (dd, *J* = 11.2, 3.6 Hz, 1H), 4.18–4.10 (m, 2H), 3.84 (dd, *J* = 15.3, 7.2 Hz, 1H), 2.42 (s, 3H) ppm. ^13^C{^1^H} NMR (101 MHz, CDCl_3_) δ 161.9, 145.4, 134.8, 132.7, 132.4, 131.9, 130.1, 129.4, 128.6, 128.1, 118.1, 112.9, 76.9, 69.8, 56.9, 21.8 ppm. HRMS (ESI) Calcd for C_18_H_17_N_2_O_4_S^+^ [M + H]^+^ 357.0904, found 357.0910.

#### (2-(3-(Trifluoromethyl)phenyl)-4,5-dihydrooxazol-5-yl)methyl 4-methylbenzenesulfonate (**2h**)

White solid (69 mg, 58%). M.p. 102–103 °C. Purified by flash column chromatography (ethyl acetate/hexane 12–100% gradient). ^1^H NMR (400 MHz, CDCl_3_) δ 8.09 (s, 1H), 8.03 (d, *J* = 7.8 Hz, 1H), 7.81-7.70 (m, 3H), 7.53 (t, *J* = 7.8 Hz, 1H), 7.28 (d, *J* = 8.1 Hz, 2H), 4.97–4.85 (m, 1H), 4.23 (dd, *J* = 11.1, 3.6 Hz, 1H), 4.18–4.10 (m, 2H), 3.83 (dd, *J* = 15.2, 7.2 Hz, 1H), 2.39 (s, 3H) ppm. ^13^C{^1^H} NMR (101 MHz, CDCl_3_) δ 162.6, 145.3, 132.7, 131.6, 131.2, 130.9, 130.1, 129.1, 128.2 (q, ^4^*J*_C-F_ = 3.8 Hz), 128.0, 125.2 (q, ^4^*J*_C-F_ = 3.9 Hz), 123.8 (q, ^1^*J*_C-F_ = 270 Hz), 76.8, 69.9, 56.9, 21.7 ppm. ^19^F NMR (376 MHz, CDCl_3_) δ −62.80 ppm. HRMS (ESI) Calcd for C_18_H_17_F_3_NO_4_S^+^ [M + H]^+^ 400.0825, found 400.0834.

#### (2-(3-Nitrophenyl)-4,5-dihydrooxazol-5-yl)methyl 4-methylbenzenesulfonate (**2i**)

Thick yellow oil (40 mg, 35%). Purified by flash column chromatography (ethyl acetate/hexane 12–100% gradient). ^1^H NMR (400 MHz, CDCl_3_) δ 8.63–8.61 (m, 1H), 8.35–8.30 (m, 1H), 8.20 (d, *J* = 7.8 Hz, 1H), 7.77 (d, *J* = 8.3 Hz, 2H), 7.60 (t, *J* = 8.0 Hz, 1H), 7.30 (d, *J* = 8.2 Hz, 2H), 5.00–4.88 (m, 1H), 4.26 (dd, *J* = 11.2, 3.6 Hz, 1H), 4.21–4.07 (m, 2H), 3.87 (dd, *J* = 15.4, 7.2 Hz, 1H), 2.40 (s, 3H) ppm. ^13^C{^1^H} NMR (101 MHz, CDCl_3_) δ 161.8, 148.3, 145.4, 134.1, 132.6, 130.1, 129.6, 129.0, 128.0, 126.1, 123.3, 77.0, 69.8, 56.9, 21.8 ppm. HRMS (ESI) Calcd for C_17_H_17_N_2_O_6_S^+^ [M + H]^+^ 377.0802, found 377.0808.

#### (2-(o-Tolyl)-4,5-dihydrooxazol-5-yl)methyl 4-methylbenzenesulfonate (**2j**)

Thick pale-yellow oil (83 mg, 80%). Purified by flash column chromatography (ethyl acetate/hexane 12–100% gradient). Mixture of diastereoisomers (dr 5:1), ^1^H NMR (400 MHz, CDCl_3_) δ 7.86 (d, *J* = 8.3 Hz, 2H minor), 7.80 (d, *J* = 8.3 Hz, 2H major), 7.69 (d, *J* = 7.8 Hz, 1H major), 7.50 (d, *J* = 6.9 Hz, 1H minor), 7.40–7.33 (m, 1H major + 2H minor), 7.29 (d, *J* = 8.2 Hz, 2H major + 2H minor), 7.26–7.17 (m, 2H major + 1H minor), 4.98–4.92 (m, 1H minor), 4.85 (dtd, *J* = 6.8, 6.0, 3.9 Hz, 1H major), 4.38–4.32 (m, 2H minor), 4.22 (dd, *J* = 10.9, 3.9 Hz, 1H major), 4.16 (ddd, *J* = 10.0, 9.2, 4.2 Hz, 2H major), 3.84 (dd, *J* = 15.0, 6.9 Hz, 1H major), 3.71 (ddd, *J* = 19.2, 17.6, 2.9 Hz, 2H minor), 2.53 (s, 3H major), 2.48 (s, 3H minor), 2.43 (s, 3H major + 3H minor) ppm. ^13^C{^1^H} NMR (101 MHz, CDCl_3_) δ 164.1, 157.4, 145.4, 145.2, 139.0, 137.0, 133.9, 133.6, 132.7, 131.4, 13.0, 130.9, 130.2, 130.0, 130.0, 129.9, 128.9, 128.1, 127.9, 126.6, 125.6, 75.4, 70.6, 70.2, 65.9, 57.3, 46.9, 21.9, 21.8, 21.8, 20.8 ppm. HRMS (ESI) Calcd for C_18_H_20_NO_4_S^+^ [M + H]^+^ 346.1108, found 346.1113.

#### (2-([1,1′-Biphenyl]-2-yl)-4,5-dihydrooxazol-5-yl)methyl 4-methylbenzenesulfonate (**2k**)

Thick pale-yellow oil (49 mg, 40%). Purified by flash column chromatography (ethyl acetate/hexane 12–100% gradient). Mixture of diastereoisomers (dr 2.3:1), ^1^H NMR (400 MHz, CDCl_3_) δ 7.77 (d, *J* = 8.3 Hz, 2H minor), 7.74 (d, *J* = 8.3 Hz, 2H major), 7.67 (d, *J* = 7.8 Hz, 1H major), 7.57 (d, *J* = 7.6 Hz, 1H minor), 7.49 (td, *J* = 7.7, 1.1 Hz, 1H major), 7.44 (d, *J* = 6.5 Hz, 1H minor), 7.40–7.27 (m, 9H major + 9H minor), 4.76–4.72 (m, 1H minor), 4.58 (ddd, *J* = 11.8, 10.3, 5.2 Hz, 1H major), 3.94 (dd, *J* = 14.9, 10.0 Hz, 1H major), 3.89–3.82 (m, 1H major + 2H minor), 3.79 (dd, *J* = 10.7, 5.4 Hz, 1H major), 3.68–3.58 (m, 1H major + 1H minor), 3.55–3.47 (m, 1H minor), 2.44 (s, 3H minor), 2.43 (s, 3H major) ppm. ^13^C{^1^H} NMR (101 MHz, CDCl_3_) δ 164.9, 158.1, 145.3, 145.3, 142.2, 141.6, 141.2, 133.8, 133.3, 132.7, 130.8, 130.5, 130.2, 130.2, 130.1 (2C), 130.0, 129.7, 128.6, 128.3 (2C), 128.1 (2C), 127.9, 127.4, 127.2, 127.1, 127.0, 76.0, 70.5, 69.3, 65.7, 57.3, 47.0, 21.8 ppm. HRMS (ESI) Calcd for C_23_H_22_NO_4_S^+^ [M + H]^+^ 408.1264, found 408.1269.

#### (2-(2-Bromophenyl)-4,5-dihydrooxazol-5-yl)methyl 4-methylbenzenesulfonate (**2l**)

Thick pale-yellow oil (97 mg, 79%). Purified by flash column chromatography (ethyl acetate/hexane 12–100% gradient). Mixture of diastereoisomers (dr 2.2:1), ^1^H NMR (400 MHz, CDCl_3_) δ 7.84 (d, *J* = 8.2 Hz, 2H minor), 7.79 (d, *J* = 8.2 Hz, 2H major), 7.63–7.58 (m, 2H major), 7.55 (d, *J* = 7.9 Hz, 1H minor), 7.44 (dd, *J* = 7.6, 1.6 Hz, 1H minor), 7.38–7.27 (m, 4H major + 3H minor), 7.22 (td, *J* = 7.7, 1.6 Hz, 1H minor), 4.98–4.84 (m, 1H major + 1H minor), 4.34 (d, *J* = 1.6 Hz, 2H minor), 4.21 (dd, *J* = 10.9, 4.1 Hz, 1H major), 4.19–4.11 (m, 2H major), 3.83 (dd, *J* = 15.1, 6.9 Hz, 1H major), 3.75 (dd, *J* = 17.6, 4.1 Hz, 1H minor), 3.69–3.60 (m, 1H minor), 2.45 (s, 3H minor), 2.41 (s, 3H major) ppm. ^13^C{^1^H} NMR (101 MHz, CDCl_3_) δ 162.9, 156.5, 145.4, 145.3, 135.6, 134.0, 133.8, 133.3, 132.7, 132.0, 131.5, 131.1, 130.5, 130.2, 130.0, 129.0, 128.1, 127.9, 127.3, 127.2, 122.0, 121.2, 76.4, 70.3, 69.9, 66.0, 57.3, 47.0, 21.8 (2C) ppm. HRMS (ESI) Calcd for C_17_H_17_BrNO_4_S^+^ [M + H]^+^ 410.0056, found 410.0067.

#### (2-(2-Chlorophenyl)-4,5-dihydrooxazol-5-yl)methyl 4-methylbenzenesulfonate (**2m**)

Thick colorless oil (78 mg, 71%). Purified by flash column chromatography (ethyl acetate/hexane 12–100% gradient). Mixture of diastereoisomers (dr 2.5:1), ^1^H NMR (400 MHz, CDCl_3_) δ 7.84 (d, *J* = 8.3 Hz, 2H minor), 7.79 (d, *J* = 8.3 Hz, 2H major), 7.66 (dd, *J* = 7.8, 1.6 Hz, 1H major), 7.48 (dd, *J* = 7.6, 1.7 Hz, 1H minor), 7.45–7.38 (m, 1H major + 1H minor), 7.38–7.34 (m, 1H major + 2H minor), 7.32 (dd, *J* = 7.3, 1.8 Hz, 1H minor), 7.30–7.27 (m, 3H major), 7.24 (dd, *J* = 7.6, 1.6 Hz, 1H minor), 4.96–4.92 (m, 1H minor), 4.92–4.85 (m, 1H major), 4.36–4.32 (m, 2H minor), 4.23–4.13 (m, 3H major), 3.85 (dd, *J* = 15.2, 6.9 Hz, 1H major), 3.75 (dd, *J* = 17.7, 4.2 Hz, 1H minor), 3.69–3.61 (m, 1H minor), 2.46 (s, 3H minor), 2.41 (s, 3H minor) ppm^13^C{^1^H} NMR (101 MHz, CDCl_3_) δ 162.2, 155.8, 145.3, 133.8, 133.6, 132.7, 131.9, 131.5, 131.0, 130.9, 130.5, 130.2, 130.2, 130.1, 128.1, 127.9, 126.8, 126.7 (2C), 76.1, 70.3, 69.9, 66.1, 57.3, 47.1, 21.8, 21.8 ppm. HRMS (ESI) Calcd for C_17_H_17_ClNO_4_S^+^ [M + H]^+^ 366.0561, found 366.0566.

#### (2-(2-Fluorophenyl)-4,5-dihydrooxazol-5-yl)methyl 4-methylbenzenesulfonate (**2n**)

Thick pale-yellow oil (45 mg, 43%). Purified by flash column chromatography (ethyl acetate/hexane 12–100% gradient). ^1^H NMR (400 MHz, CDCl_3_) δ 7.77 (d, *J* = 8.3 Hz, 2H), 7.74 (dd, *J* = 7.6, 1.7 Hz, 1H), 7.45 (tdd, *J* = 8.3, 4.9, 1.8 Hz, 1H), 7.28 (d, *J* = 8.0 Hz, 2H), 7.19–7.08 (m, 2H), 4.89–4.81 (m, 1H), 4.20 (dd, *J* = 11.0, 3.9 Hz, 1H), 4.16–4.11 (m, 2H), 3.83 (dd, *J* = 15.3, 7.1 Hz, 1H), 2.39 (s, 3H) ppm. ^13^C{^1^H} NMR (101 MHz, CDCl_3_) δ 161.3 (d, ^1^*J*_C-F_ = 258.6 Hz), 160.3 (d, ^3^*J*_C-F_ = 6.0 Hz), 145.2, 133.2 (d, ^3^*J*_C-F_ = 8.8 Hz), 132.7, 131.1 (d, ^4^*J*_C-F_ = 1.3 Hz), 130.0, 128.1, 124.0 (d, ^3^*J*_C-F_ = 3.8 Hz), 116.8 (d, ^2^*J*_C-F_ = 21.9 Hz), 115.5 (d, ^2^*J*_C-F_ = 10.2 Hz), 75.7, 69.9, 57.2, 21.8 ppm. ^19^F NMR (376 MHz, CDCl_3_) δ −109.16 ppm. HRMS (ESI) Calcd for C_17_H_17_FNO_4_S^+^ [M + H]^+^ 350.0857, found 350.0860.

#### (5-Methyl-2-phenyl-4,5-dihydrooxazol-5-yl)methyl 4-methylbenzenesulfonate (**2o**)

Pale-yellow solid (68 mg, 66%). M.p. 81–82 °C. Purified by flash column chromatography (ethyl acetate/hexane 12–100% gradient). ^1^H NMR (400 MHz, CDCl_3_) δ 7.81 (d, *J* = 7.4 Hz, 2H), 7.76 (d, *J* = 8.2 Hz, 2H), 7.47 (t, *J* = 7.4 Hz, 1H), 7.38 (t, *J* = 7.6 Hz, 2H), 7.26 (d, *J* = 8.1 Hz, 2H), 4.10 (d, *J* = 10.4 Hz, 1H), 4.05 (d, *J* = 10.4 Hz, 1H), 3.92 (d, *J* = 15.0 Hz, 1H), 3.71 (d, *J* = 15.0 Hz, 1H), 2.40 (s, 3H), 1.48 (s, 3H) ppm. ^13^C{^1^H} NMR (101 MHz, CDCl_3_) δ 163.1, 145.1, 132.7, 131.5, 130.0, 128.4, 128.3, 128.0, 127.6, 83.1, 72.8, 63.2, 22.8, 21.8 ppm. HRMS (ESI) Calcd for C_18_H_20_NO_4_S^+^ [M + H]^+^ 346.1108, found 346.1116.

#### (2-(4-Chlorophenyl)-5-methyl-4,5-dihydrooxazol-5-yl)methyl 4-methylbenzenesulfonate (**2p**)

White solid (68 mg, 60%). M.p. 110–111 °C. Purified by flash column chromatography (ethyl acetate/hexane 12–100% gradient). ^1^H NMR (400 MHz, CDCl_3_) δ 7.76–7.71 (m, 4H), 7.38–7.32 (m, 2H), 7.26 (d, *J* = 8.0 Hz, 2H), 4.10 (d, *J* = 10.5 Hz, 1H), 4.03 (d, *J* = 10.5 Hz, 1H), 3.91 (d, *J* = 15.1 Hz, 1H), 3.70 (d, *J* = 15.1 Hz, 1H), 2.40 (s, 3H), 1.46 (s, 3H) ppm. ^13^C{^1^H} NMR (101 MHz, CDCl_3_) δ 162.3, 145.2, 137.8, 132.7, 130.0, 129.6, 128.7, 128.0, 126.1, 83.5, 72.8, 63.1, 22.8, 21.8 ppm. HRMS (ESI) Calcd for C_18_H_19_ClNO_4_S^+^ [M + H]^+^ 380.0718, found 380.0726.

#### (2-(2-Fluoro-4-methoxyphenyl)-4,5-dihydrooxazol-5-yl)methyl 4-methylbenzenesulfonate (**2q**)

Pale-yellow solid (87 mg, 76%). M.p. 91–92 °C. Purified by flash column chromatography (ethyl acetate/hexane 12–100% gradient). ^1^H NMR (400 MHz, CDCl_3_) δ 7.78 (d, *J* = 8.3 Hz, 2H), 7.67 (t, *J* = 8.5 Hz, 1H), 7.29 (d, *J* = 8.1 Hz, 2H), 6.67 (ddd, *J* = 15.0, 10.7, 2.5 Hz, 2H), 4.86–4.76 (m, 1H), 4.20–4.08 (m, 3H), 3.83 (s, 3H), 3.79 (dd, *J* = 15.1, 7.0 Hz, 1H), 2.41 (s, 3H) ppm. ^13^C{^1^H} NMR (101 MHz, CDCl_3_) δ 163.5 (d, ^3^*J*_C-F_ = 11.4 Hz), 162.5 (d, ^1^*J*_C-F_ = 258.6 Hz), 160.2 (d, ^3^*J*_C-F_ = 6.5 Hz), 145.2, 132.7, 132.0 (d, ^3^*J*_C-F_ = 3.4 Hz), 130.1, 128.1, 110.2 (d, ^4^*J*_C-F_ = 2.9 Hz), 107.9 (d, ^2^*J*_C-F_ = 10.5 Hz), 102.4 (d, ^2^*J*_C-F_ = 25.4 Hz), 75.4, 70.0, 57.2, 55.9, 21.8 ppm. ^19^F NMR (376 MHz, CDCl_3_) δ −106.34 ppm. HRMS (ESI) Calcd for C_18_H_19_FNO_5_S^+^ [M + H]^+^ 380.0962, found 380.0972.

#### (2-(3-Fluoro-4-methylphenyl)-4,5-dihydrooxazol-5-yl)methyl 4-methylbenzenesulfonate (**2r**)

Pale-yellow solid (91 mg, 83%). M.p. 85–86 °C. Purified by flash column chromatography (ethyl acetate/hexane 12–100% gradient). ^1^H NMR (400 MHz, CDCl_3_) δ 7.77 (d, *J* = 8.3 Hz, 2H), 7.52 (dd, *J* = 7.9, 1.5 Hz, 1H), 7.40 (dd, *J* = 10.3, 1.5 Hz, 1H), 7.29 (d, *J* = 8.0 Hz, 2H), 7.19 (t, *J* = 7.8 Hz, 1H), 5.08–4.57 (m, 1H), 4.19 (dd, *J* = 11.0, 3.8 Hz, 1H), 4.16–4.12 (m, 1H), 4.09 (dd, *J* = 12.5, 7.4 Hz, 1H), 3.77 (dd, *J* = 15.1, 7.1 Hz, 1H), 2.40 (s, 3H), 2.31 (d, *J* = 1.8 Hz, 3H) ppm. ^13^C{^1^H} NMR (101 MHz, CDCl_3_) δ 162.9 (d, ^4^*J*_C-F_ = 3.0 Hz), 160.9 (d, ^1^*J*_C-F_ = 245.0 Hz), 145.3, 132.7, 131.5 (d, ^3^*J*_C-F_ = 5.2 Hz), 130.1, 129.0 (d, ^2^*J*_C-F_ = 17.3 Hz), 128.1, 126.6 (d, ^3^*J*_C-F_ = 8.4 Hz), 123.8 (d, ^4^*J*_C-F_ = 3.5 Hz), 114.9 (d, ^2^*J*_C-F_ = 24.7 Hz), 76.5, 70.1, 56.8, 21.7, 14.9 (d, ^3^*J*_C-F_ = 3.5 Hz) ppm. ^19^F NMR (376 MHz, CDCl_3_) δ −116.88 ppm. HRMS (ESI) Calcd for C_18_H_19_FNO_4_S^+^ [M + H]^+^ 364.1013, found 364.1023.

#### (2-Benzyl-4,5-dihydrooxazol-5-yl)methyl 4-methylbenzenesulfonate (**2s**)

Pale-yellow thick oil (68 mg, 66%). Purified by flash column chromatography (ethyl acetate/hexane 12–100% gradient). ^1^H NMR (400 MHz, CDCl_3_) δ 7.74 (d, *J* = 8.3 Hz, 2H), 7.33 (d, *J* = 8.0 Hz, 2H), 7.31–7.26 (m, 3H), 7.24 (dd, *J* = 6.3, 1.6 Hz, 2H), 4.72–4.60 (m, 1H), 4.05 (dd, *J* = 10.8, 4.0 Hz, 1H), 4.00 (dd, *J* = 10.8, 5.5 Hz, 1H), 3.93–3.84 (m, 1H), 3.61–3.51 (m, 3H), 2.45 (s, 3H) ppm. ^13^C{^1^H} NMR (101 MHz, CDCl_3_) δ 166.3, 145.3, 134.8, 132.7, 130.1, 129.1, 128.7, 128.1, 127.2, 126.0, 76.3, 69.9, 56.5, 34.7, 21.8 ppm. HRMS (ESI) Calcd for C_18_H_20_NO_4_S^+^ [M + H]^+^ 346.1108, found 346.1113.

#### (2-(4-Chlorobenzyl)-4,5-dihydrooxazol-5-yl)methyl 4-methylbenzenesulfonate (**2t**)

Pale-yellow solid (86 mg, 75%). M.p. 110–111 °C. Purified by flash column chromatography (ethyl acetate/hexane 12–100% gradient). ^1^H NMR (400 MHz, CDCl_3_) δ 7.76 (d, *J* = 8.3 Hz, 2H), 7.36 (d, *J* = 8.0 Hz, 2H), 7.30–7.26 (m, 2H), 7.20 (d, *J* = 8.6 Hz, 2H), 4.76–4.66 (m, 1H), 4.09 (dd, *J* = 10.9, 3.7 Hz, 1H), 4.02 (dd, *J* = 10.9, 5.5 Hz, 1H), 3.91 (ddt, *J* = 14.5, 10.1, 1.1 Hz, 1H), 3.60 (dd, *J* = 14.6, 6.9 Hz, 1H), 3.53 (s, 2H), 2.48 (s, 3H) ppm. ^13^C{^1^H} NMR (101 MHz, CDCl_3_) δ 165.9, 145.3, 133.3, 133.1, 132.7, 130.5, 130.1, 128.9, 128.1, 76.4, 69.8, 56.5, 34.0, 21.8 ppm. HRMS (ESI) Calcd for C_18_H_19_ClNO_4_S^+^ [M + H]^+^ 380.0718, found 380.0723.

#### (2-(tert-Butyl)-4,5-dihydrooxazol-5-yl)methyl 4-methylbenzenesulfonate (**2w**)

Colorless thick oil (36 mg, 39%). Purified by flash column chromatography (ethyl acetate/hexane 12–100% gradient). ^1^H NMR (400 MHz, CDCl_3_) δ 7.78 (d, *J* = 8.3 Hz, 2H), 7.34 (d, *J* = 8.1 Hz, 2H), 4.67 (dtd, *J* = 10.1, 6.2, 4.0 Hz, 1H), 4.03 (dd, *J* = 10.6, 3.9 Hz, 1H), 3.96 (dd, *J* = 10.6, 5.9 Hz, 1H), 3.85 (dd, *J* = 14.5, 10.0 Hz, 1H), 3.51 (dd, *J* = 14.5, 6.6 Hz, 1H), 2.44 (s, 3H), 1.14 (s, 9H) ppm. ^13^C{^1^H} NMR (101 MHz, CDCl_3_) δ 174.1, 145.3, 132.7, 130.1, 128.1, 75.8, 70.2, 56.4, 33.3, 27.6, 21.8 ppm. HRMS (ESI) Calcd for C_15_H_22_NO_4_S^+^ [M + H]^+^ 312.1264, found 312.1268.

#### (2-(Furan-2-yl)-4,5-dihydrooxazol-5-yl)methyl 4-methylbenzenesulfonate (**2**×)

Light brown solid (40 mg, 41%). M.p. 90–91 °C. Purified by flash column chromatography (ethyl acetate/hexane 12–100% gradient). ^1^H NMR (400 MHz, CDCl_3_) δ 7.78 (d, *J* = 8.3 Hz, 2H), 7.53–7.52 (m, 1H), 7.31 (d, *J* = 8.0 Hz, 2H), 6.87 (d, *J* = 3.4 Hz, 1H), 6.47 (dd, *J* = 3.5, 1.8 Hz, 1H), 4.94–4.74 (m, 1H), 4.17 (dd, J = 11.0, 4.1 Hz, 1H), 4.15–4.06 (m, 2H), 3.77 (dd, *J* = 15.1, 7.1 Hz, 1H), 2.43 (s, 3H) ppm. ^13^C{^1^H} NMR (101 MHz, CDCl_3_) δ 156.1, 145.6, 145.3, 142.5, 132.7, 130.1, 128.1, 114.9, 111.7, 76.6, 69.7, 56.8, 21.8 ppm. HRMS (ESI) Calcd for C_15_H_16_NO_5_S^+^ [M + H]^+^ 322.0744, found 322.0749.

#### (2-(Furan-2-yl)-5-methyl-4,5-dihydrooxazol-5-yl)methyl 4-methylbenzenesulfonate (**2y**)

Pale-yellow thick oil (35 mg, 35%). Purified by flash column chromatography (ethyl acetate/hexane 12–100% gradient). ^1^H NMR (400 MHz, CDCl_3_) δ 7.76 (d, *J* = 8.3 Hz, 2H), 7.51 (d, *J* = 1.0 Hz, 1H), 7.30 (d, *J* = 8.0 Hz, 2H), 6.84 (d, *J* = 3.4 Hz, 1H), 6.47 (dd, *J* = 3.5, 1.8 Hz, 1H), 4.08 (d, *J* = 10.4 Hz, 1H), 4.02 (d, *J* = 10.4 Hz, 1H), 3.90 (d, *J* = 15.0 Hz, 1H), 3.69 (d, *J* = 15.0 Hz, 1H), 2.42 (s, 3H), 1.46 (s, 3H) ppm. ^13^C{^1^H} NMR (101 MHz, CDCl_3_) δ 155.4, 145.4, 145.2, 142.7, 132.6, 130.1, 128.1, 114.6, 111.6, 83.6, 72.5, 63.0, 22.7, 21.8 ppm. HRMS (ESI) Calcd for C_16_H_18_NO_5_S^+^ [M + H]^+^ 336.0900, found 336.0905.

#### (2-(4-Chlorophenyl)-5,6-dihydro-4H-1,3-oxazin-6-yl)methyl 4-methylbenzenesulfonate (**2z**)

Pale-yellow solid (87 mg, 76%). M.p. 114–115 °C. Purified by flash column chromatography (ethyl acetate/hexane 12–100% gradient). ^1^H NMR (400 MHz, CDCl_3_) δ = 7.81 (d, *J* = 8.3 Hz, 2H), 7.72 (d, *J* = 8.6 Hz, 2H), 7.33 (d, *J* = 8.2 Hz, 2H), 7.30 (d, *J* = 8.6 Hz, 2H), 4.45 (ddd, *J* = 9.8, 6.4, 3.8 Hz, 1H), 4.26–4.15 (m, 2H), 3.65 (ddd, *J* = 16.9, 5.4, 2.9 Hz, 1H), 3.59–3.49 (m, 1H), 2.44 (s, 3H), 1.97–1.89 (m, 1H), 1.82–1.70 (m, 1H) ppm. ^13^C{^1^H} NMR (101 MHz, CDCl_3_) δ = 153.9, 145.3, 136.8, 132.8, 131.9, 130.1, 128.5, 128.3, 128.1, 72.0, 70.8, 42.2, 23.1, 21.8 ppm. HRMS (ESI) Calcd for C_18_H_19_ClNO_4_S^+^ [M + H]^+^ 380.0718, found 380.0728.

#### (2-(3-(Trifluoromethyl)phenyl)-5,6-dihydro-4H-1,3-oxazin-6-yl)methyl 4-methylbenzenesulfonate (**2aa**)

Pale-yellow solid (84 mg, 68%). M.p. 130–131 °C. Purified by flash column chromatography (ethyl acetate/hexane 12–100% gradient). ^1^H NMR (400 MHz, CDCl_3_) δ 8.08 (s, 1H), 7.99 (d, *J* = 7.9 Hz, 1H), 7.82 (d, *J* = 7.7 Hz, 2H), 7.66 (d, *J* = 7.7 Hz, 1H), 7.46 (t, *J* = 7.8 Hz, 1H), 7.33 (d, *J* = 7.9 Hz, 2H), 4.49 (td, *J* = 8.9, 3.9 Hz, 1H), 4.27–4.17 (m, 2H), 3.76–3.64 (m, 1H), 3.62–3.52 (m, 1H), 2.42 (s, 3H), 2.01–1.91 (m, 1H), 1.86–1.74 (m, 1H) ppm. ^13^C{^1^H} NMR (101 MHz, CDCl_3_) δ 153.6, 145.4, 140.8, 134.2, 132.7, 130.4, 130.1, 128.7, 128.1, 127.5 (q, ^1^*J*_C-F_ = 331.2 Hz), 127.2 (q, ^3^*J*_C-F_ = 3.7 Hz), 124.1 (q, ^3^*J*_C-F_ = 3.9 Hz), 72.2, 70.7, 42.2, 23.1, 21.8 ppm. ^19^F NMR (376 MHz, CDCl_3_) δ −62.62 ppm. HRMS (ESI) Calcd for C_19_H_19_F_3_NO_4_S^+^ [M + H]^+^ 414.0981, found 414.0992.

### Catalytic Electrosynthetic α-Tosyloxylation of Ketones

A solution of ketone substrate **3** (0.3 mmol, 1 equiv) and tosylic acid (0.75 mmol, 2.5 equiv) in a mixture of HFIP (4 mL) and acetonitrile (1 mL) containing iodobenzene (18.4 mg, 10 μL, 0.09 mmol, 0.3 equiv) was electrolyzed using an ElectraSyn undivided cell (5 mL glass vial) equipped with graphite anode and platinum cathode under constant current of 5 mA with stirring (400 rpm) for 4.82 h (3.0 F). After electrolysis, the electrodes were rinsed with DCM and combined with the reaction mixture, then treated with sat. aq. Na_2_S_2_O_3_ solution (5 mL) and sat. aq. NaHCO_3_ solution (5 mL), and diluted with DCM (10 mL). The phases were separated, and the aqueous layer was extracted with DCM (2×) The combined organic layers were washed with water (1×) and brine (1×), then dried over anhydrous magnesium sulfate, filtered, and concentrated under reduced pressure. The crude product was purified by flash chromatography using Biotage Isolera Four, applying ethyl acetate/pet. ether 4–40% gradient.

#### 1-Oxo-1-phenylpropan-2-yl 4-methylbenzenesulfonate (**4a**)^22^

Pale-yellow solid (74 mg, 81%). Purified by flash column chromatography (ethyl acetate/hexane 4–40% gradient). ^1^H NMR (500 MHz, CDCl_3_) δ = 7.91–7.84 (m, 2H), 7.75 (d, *J* = 8.3 Hz, 2H), 7.63–7.55 (m, 1H), 7.49–7.42 (m, 2H), 7.26 (d, *J* = 7.9 Hz, 2H), 5.78 (q, *J* = 6.9 Hz, 1H), 2.41 (s, 3H), 1.60 (d, *J* = 6.9 Hz, 3H) ppm. ^13^C{^1^H} NMR (101 MHz, CDCl_3_) δ = 195.0, 145.2, 134.0, 133.9, 133.7, 129.9, 128.9, 128.1, 77.5, 21.8, 18.9 ppm.

#### 1-(3-Chlorophenyl)-1-oxopropan-2-yl 4-methylbenzenesulfonate (**4b**)^22^

White solid (77 mg, 76%). Purified by flash column chromatography (ethyl acetate/hexane 4–40% gradient). ^1^H NMR (400 MHz, CDCl_3_) δ 7.83–7.76 (m, 2H), 7.73 (d, *J* = 8.3 Hz, 2H), 7.58–7.53 (m, 1H), 7.40 (t, *J* = 7.8 Hz, 1H), 7.27 (d, *J* = 8.7 Hz, 2H), 5.68 (q, *J* = 6.9 Hz, 1H), 2.42 (s, 3H), 1.60 (d, *J* = 6.9 Hz, 3H) ppm. ^13^C{^1^H} NMR (101 MHz, CDCl_3_) δ 194.1, 145.4, 135.4, 135.3, 133.9, 133.4, 130.2, 130.0, 128.9, 128.1, 127.0, 77.6, 21.8, 18.8 ppm.

#### 1-(4-Fluorophenyl)-1-oxopropan-2-yl 4-methylbenzenesulfonate (**4c**)^64^

White solid (80 mg, 83%). Purified by flash column chromatography (ethyl acetate/hexane 4–40% gradient). ^1^H NMR (400 MHz, CDCl_3_) δ = 8.04–7.82 (m, 2H), 7.74 (d, *J* = 8.3 Hz, 2H), 7.27 (d, *J* = 8.5 Hz, 2H), 7.12 (t, *J* = 8.6 Hz, 2H), 5.70 (q, *J* = 6.9 Hz, 1H), 2.41 (s, 3H), 1.58 (d, *J* = 6.9 Hz, 3H) ppm. ^13^C{^1^H} NMR (101 MHz, CDCl_3_) δ = 193.5, 166.2 (d, ^1^*J*_C-F_ = 256.7 Hz), 145.3, 133.50, 131.7 (d, ^3^*J*_C-F_ = 9.5 Hz), 130.2 (d, ^4^*J*_C-F_ = 3.1 Hz), 129.9, 128.1, 116.1 (d, ^2^*J*_C-F_ = 22.0 Hz), 77.6, 21.8, 18.8 ppm. ^19^F NMR (376 MHz, CDCl_3_) δ = −103.23 ppm.

#### 1-Oxo-1-(4-(trifluoromethyl)phenyl)propan-2-yl 4-methylbenzenesulfonate (**4d**)^65^

White solid (103 mg, 92%). Purified by flash column chromatography (ethyl acetate/hexane 4–40% gradient). ^1^H NMR (400 MHz, CDCl_3_) δ = 7.99 (d, *J* = 8.1 Hz, 2H), 7.76–7.66 (m, 4H), 7.27 (d, *J* = 7.3 Hz, 2H), 5.70 (q, *J* = 6.9 Hz, 1H), 2.42 (s, 3H), 1.60 (d, *J* = 7.0 Hz, 3H) ppm. ^13^C{^1^H} NMR (101 MHz, CDCl_3_) δ = 194.6, 145.5, 136.7 (q, ^4^*J*_C-F_ = 1.0 Hz), 135.1 (q, ^2^*J*_C-F_ = 32.9 Hz), 133.4, 130.0, 129.3, 128.1, 125.9 (q, ^3^*J*_C-F_ = 3.8 Hz), 123.5 (d, ^1^*J*_C-F_ = 272.9 Hz), 77.8, 21.8, 18.6 ppm. ^19^F NMR (376 MHz, CDCl_3_) δ = −63.30 ppm.

#### 1-Oxo-1-(2-(trifluoromethyl)phenyl)propan-2-yl 4-methylbenzenesulfonate (**4e**)^64^

Pale-yellow thick oil (75 mg, 67%). Purified by flash column chromatography (ethyl acetate/hexane 4–40% gradient). ^1^H NMR (400 MHz, CDCl_3_) δ = 7.71–7.67 (m, 1H), 7.65 (d, *J* = 8.4 Hz, 2H), 7.61–7.55 (m, 2H), 7.49–7.42 (m, 1H), 7.26 (d, *J* = 8.0 Hz, 2H), 5.50 (q, *J* = 6.9 Hz, 1H), 2.42 (s, 3H), 1.55 (d, *J* = 6.9 Hz, 3H) ppm. ^13^C{^1^H} NMR (101 MHz, CDCl_3_) δ = 199.0, 145.2, 136.1 (q, ^4^*J*_C-F_ = 2.0 Hz), 133.5, 131.7, 130.9, 129.9, 128.1 (q, ^2^*J*_C-F_ = 32.6 Hz), 127.9, 127.7, 127.1 (q, ^3^*J*_C-F_ = 4.8 Hz), 123.3 (q, ^1^*J*_C-F_ = 273.8 Hz), 79.4, 21.8, 17.7 ppm. ^19^F NMR (376 MHz, CDCl_3_) δ = −58.22 ppm.

#### 1-(2,3-Difluorophenyl)-1-oxopropan-2-yl 4-methylbenzenesulfonate (**4f**)

White solid (74 mg, 72%). Purified by flash column chromatography (ethyl acetate/hexane 4–40% gradient). ^1^H NMR (400 MHz, CDCl_3_) δ = 7.77 (d, *J* = 8.3 Hz, 2H), 7.51 (ddt, *J* = 7.5, 5.8, 1.6 Hz, 1H), 7.37 (dtd, *J* = 9.6, 8.1, 1.7 Hz, 1H), 7.30 (d, *J* = 8.1 Hz, 2H), 7.18 (tdd, *J* = 8.1, 4.5, 1.4 Hz, 1H), 5.66 (q, *J* = 6.9, 0.5 Hz, 1H), 2.43 (s, 3H), 1.56 (dd, *J* = 6.9, 1.1 Hz, 3H) ppm. ^13^C{^1^H} NMR (101 MHz, CDCl_3_) δ = 192.8 (dd, ^3,4^*J*_C-F_ = 3.5, 2.8 Hz), 151.4 (dd, ^1,2^*J*_C-F_ = 116.8, 13.8 Hz), 148.9 (dd, ^1,2^*J*_C-F_ = 122.0, 13.9 Hz), 145.2, 133.6, 130.0, 128.0, 125.8 (dd, *J*_C-F_ = 3.7, 1.4 Hz), 125.1, 125.0 (dd, *J*_C-F_ = 6.2, 4.5 Hz), 122.3 (dd, *J*_C-F_ = 17.3, 1.1 Hz), 79.5 (d, *J*_C-F_ = 7.1 Hz), 21.8, 17.7 (d, *J*_C-F_ = 1.9 Hz) ppm. ^19^F NMR (376 MHz, CDCl_3_) δ = −134.98 (d, *J* = 21.8 Hz), −136.55 (d, *J* = 21.8 Hz) ppm.

#### 1-Oxo-1-phenylbutan-2-yl 4-methylbenzenesulfonate (**4g**)^22^

White solid (67 mg, 70%). Purified by flash column chromatography (ethyl acetate/hexane 4–40% gradient). ^1^H NMR (400 MHz, CDCl_3_) δ = 7.85 (d, *J* = 7.4 Hz, 2H), 7.74 (d, *J* = 8.3 Hz, 2H), 7.58 (t, *J* = 7.4 Hz, 1H), 7.44 (t, *J* = 7.8 Hz, 2H), 7.24 (d, *J* = 8.2 Hz, 2H), 5.55 (dd, *J* = 7.8, 5.0 Hz, 1H), 2.39 (s, 3H), 2.04–1.79 (m, 2H), 0.98 (t, *J* = 7.4 Hz, 3H) ppm. ^13^C{^1^H} NMR (101 MHz, CDCl_3_) δ = 195.0, 145.1, 134.3, 133.9, 133.4, 129.8, 128.9, 128.8, 128.2, 82.7, 26.4, 21.8, 9.7 ppm.

## Data Availability

The data underlying this study are available in the published article and its Supporting Information.

## Supporting Information Available

Details of electrochemical cells and copies of ^1^H, ^13^C, and ^19^F NMR spectra (PDF)
