# Supplementary material for: Toward a General Protocol for Catalytic Oxidative Transformations Using Electrochemically Generated Hypervalent Iodine Species
Source: J Org Chem. 2023 Jan 23;88(3):1424–33. doi: 10.1021/acs.joc.2c02309 (PMC9903329; doi:10.1021/acs.joc.2c02309)

Supporting Information

Towards a general electrocatalytic method for in-situ hypervalent iodine species generation and reaction

Mohamed Elsherbini and Wesley J. Moran*

Department of Chemical Sciences, University of Huddersfield, Queensgate, Huddersfield HD1 3DH (UK)

E-mail: w.j.moran@hud.ac.uk

Contents

Electrolysis cells	S3
Copies of NMR spectra	S5

Electrolysis cells

Electrasyn 2.0: Screening of reaction conditions and application of the optimized conditions (scope of substrates) were performed using the ElectraSyn 2.0 with 5 mL glass vials equipped with a carbon or Pt anode and a Pt cathode.

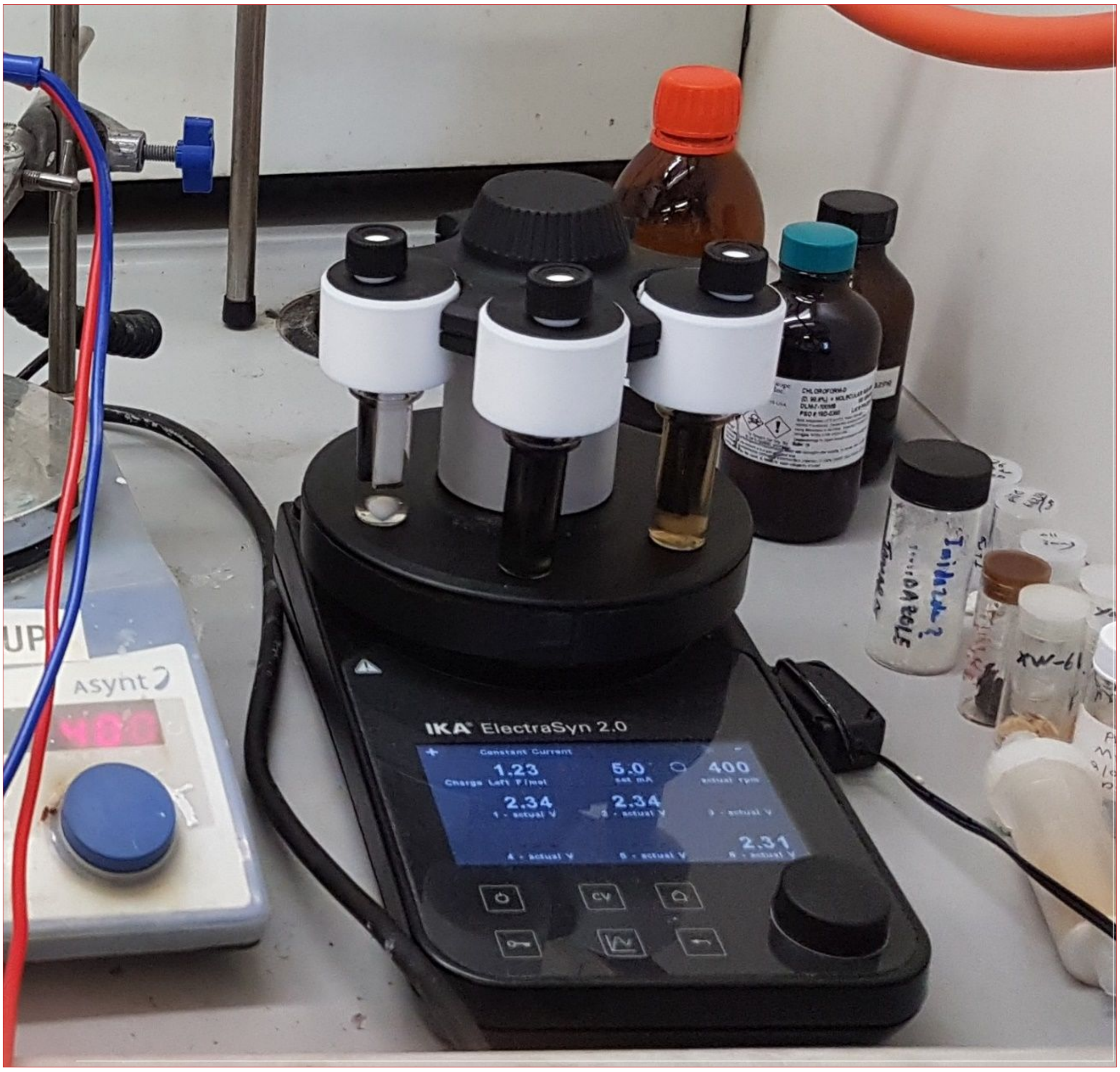

Figure S1. ElectraSyn with Carousel.

Beaker-type cell: Scale-up / catalyst recovery experiment was performed using a homemade beaker-type cell. The cell is made using a 250 mL beaker and equipped with three parallel square (5 x 5 cm) electrodes, with 0.7 cm interelectrode gap between each pair. The three parallel electrodes are in order: graphite (anode), platinum (cathode), and graphite (anode). Each graphite anode was connected to one positive (+) output of the power supply (TT*i* Ex354RT triple, 300 W), two negative outputs (-) of the power supply were connected to the common Pt cathode.

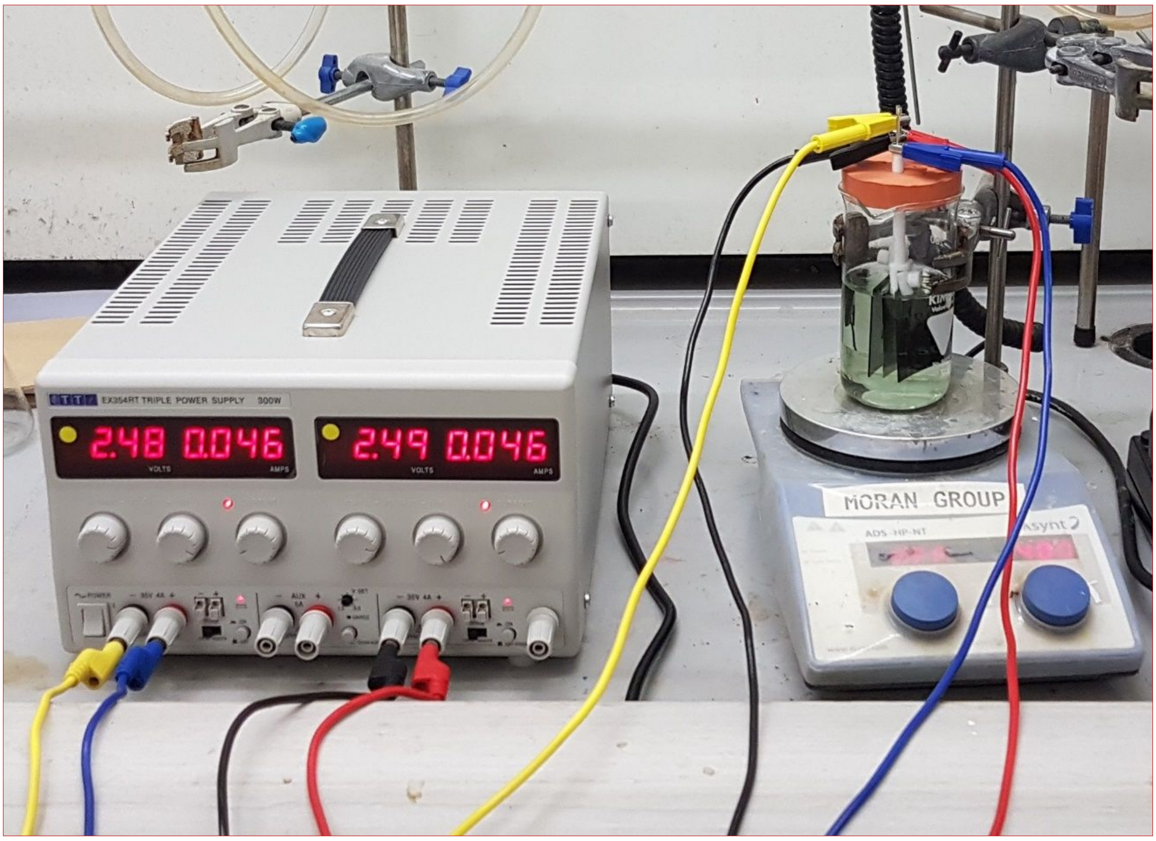

Figure S2. Actual setup of the scale-up experiment using a homemade beaker-type electrolysis cell.

N-Allylbenzamide (1a), ^{1}H NMR (400 MHz, $CDCl_3$)

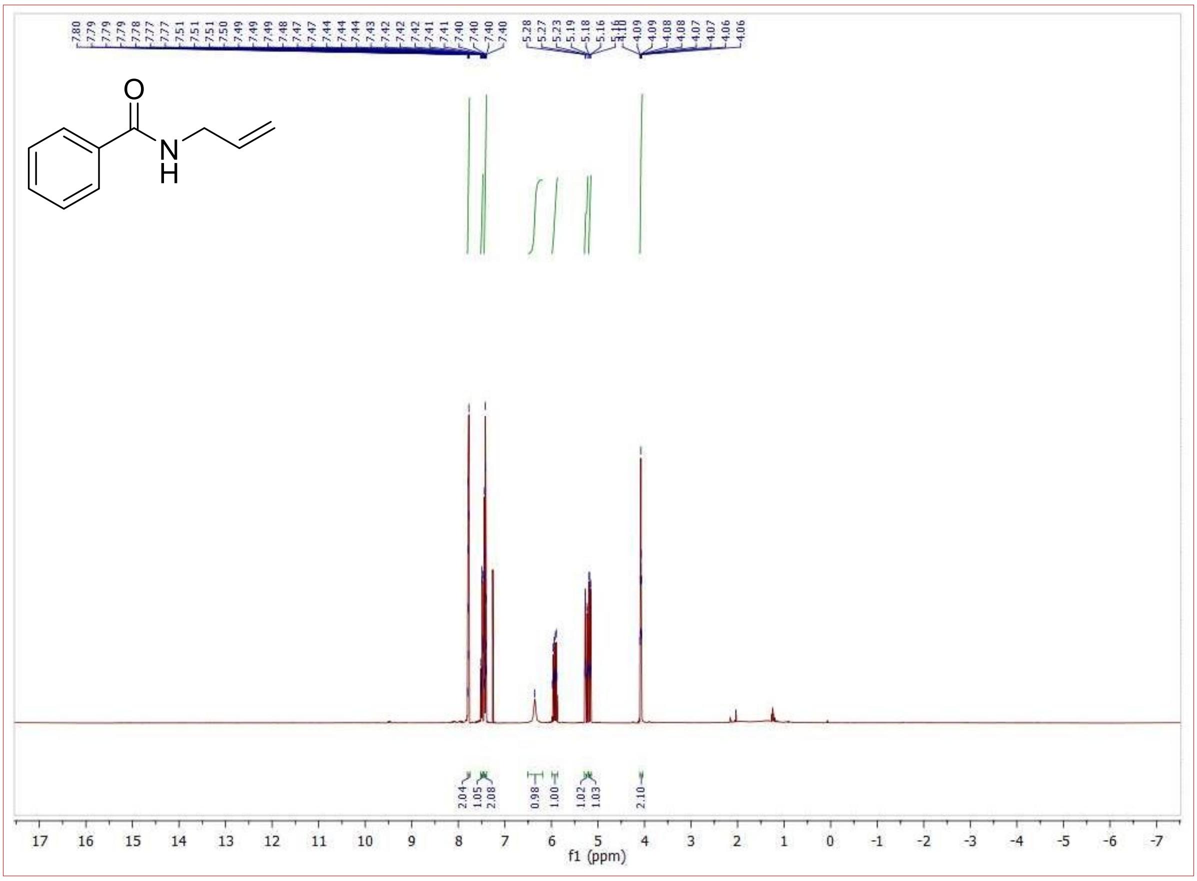

N-Allylbenzamide (1a), $^{13}C\{^{1}H\}$ NMR (101 MHz, $CDCl_3$)

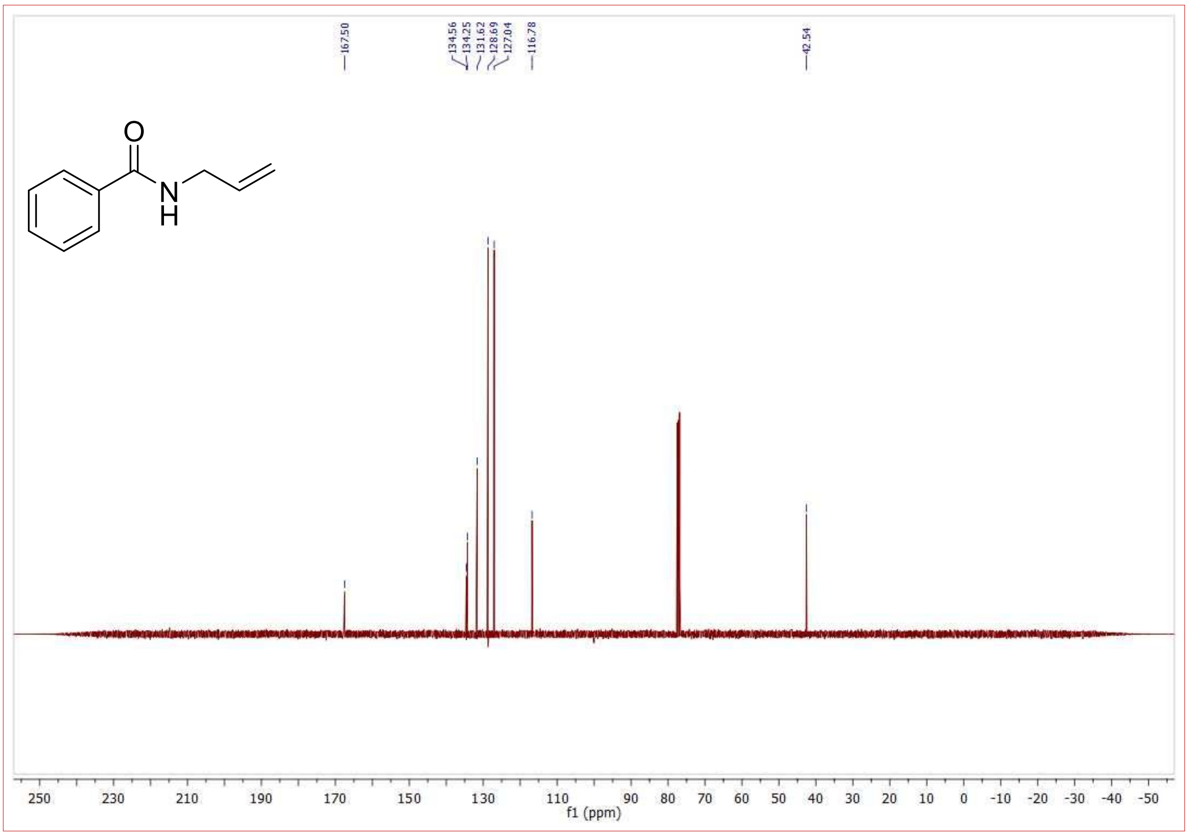

N-Allyl-4-chlorobenzamide (1b), ^{1}H NMR (400 MHz, CDCl$_3$)

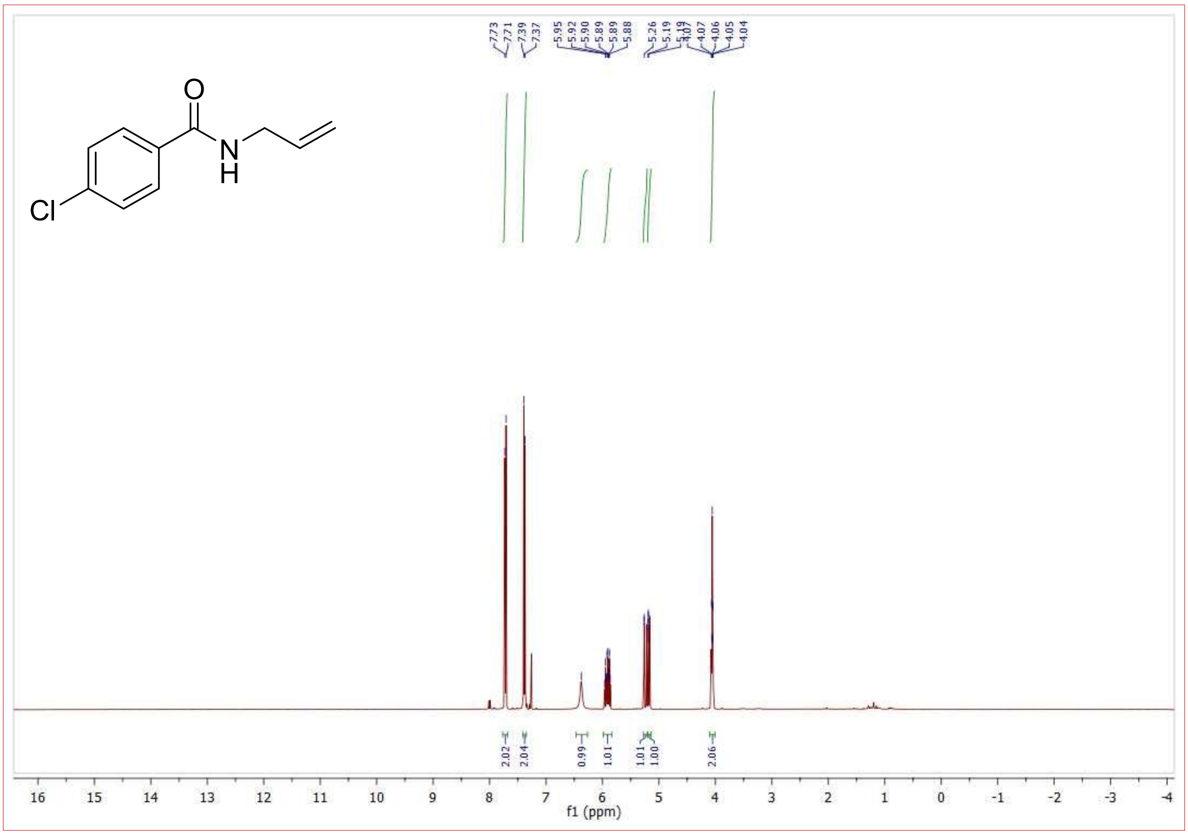

N-Allyl-4-chlorobenzamide (1b), ^{13}C{^{1}H} NMR (101 MHz, CDCl$_3$)

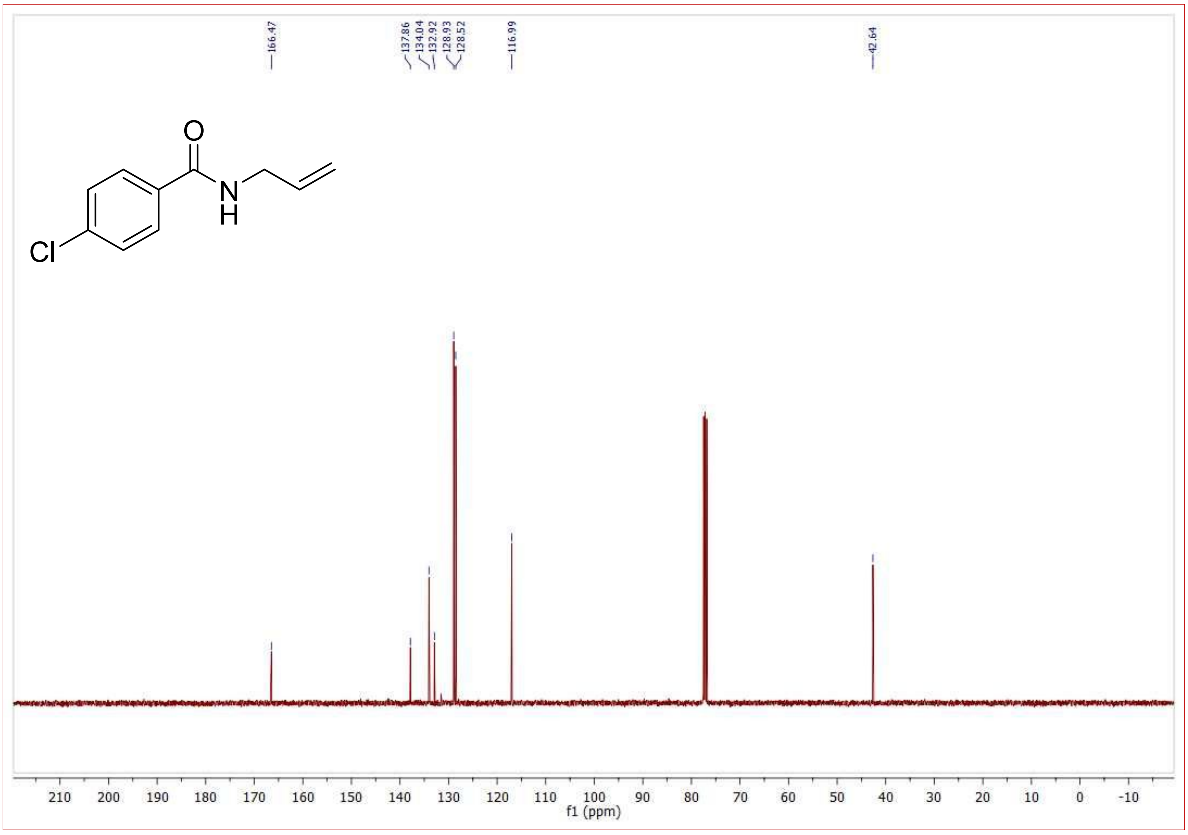

***N*-Allyl-4-iodobenzamide (1c), ^{1}H NMR (400 MHz, $CDCl_3$)**

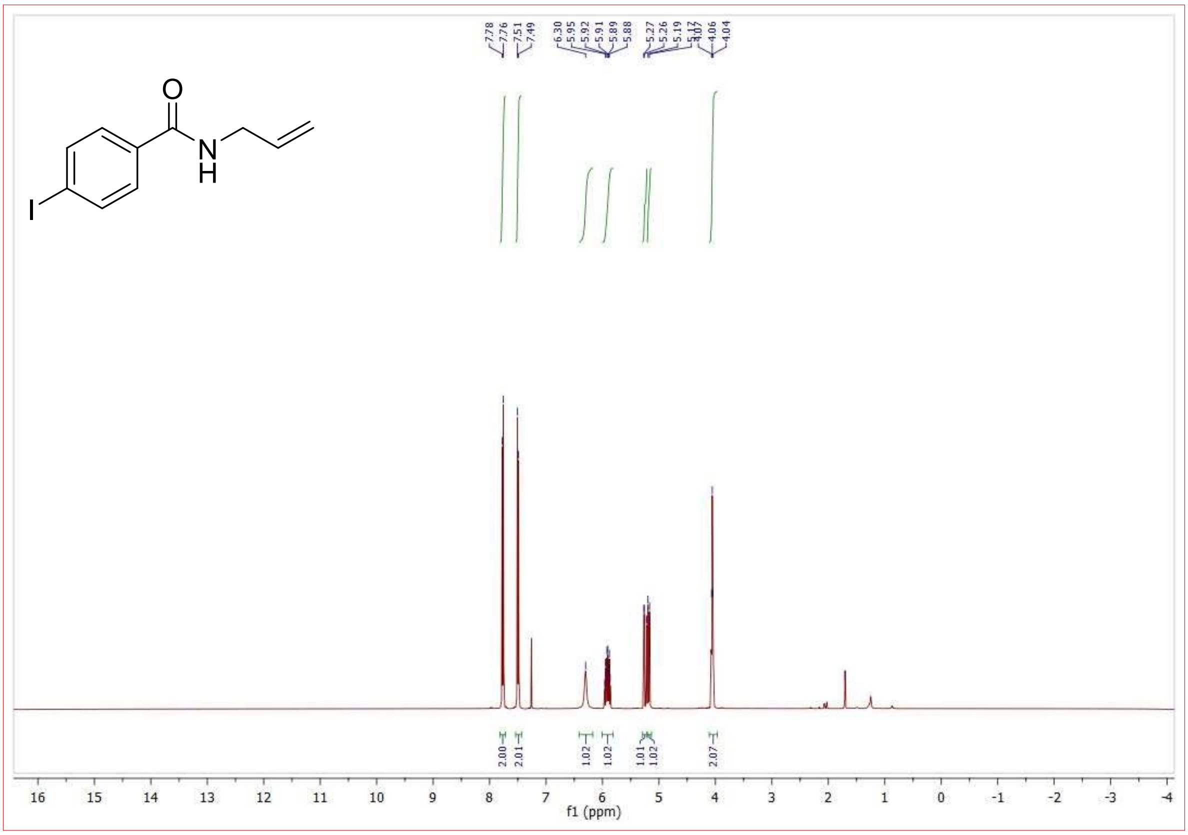

***N*-Allyl-4-iodobenzamide (1c), $^{13}C\{^{1}H\}$ NMR (101 MHz, $CDCl_3$)**

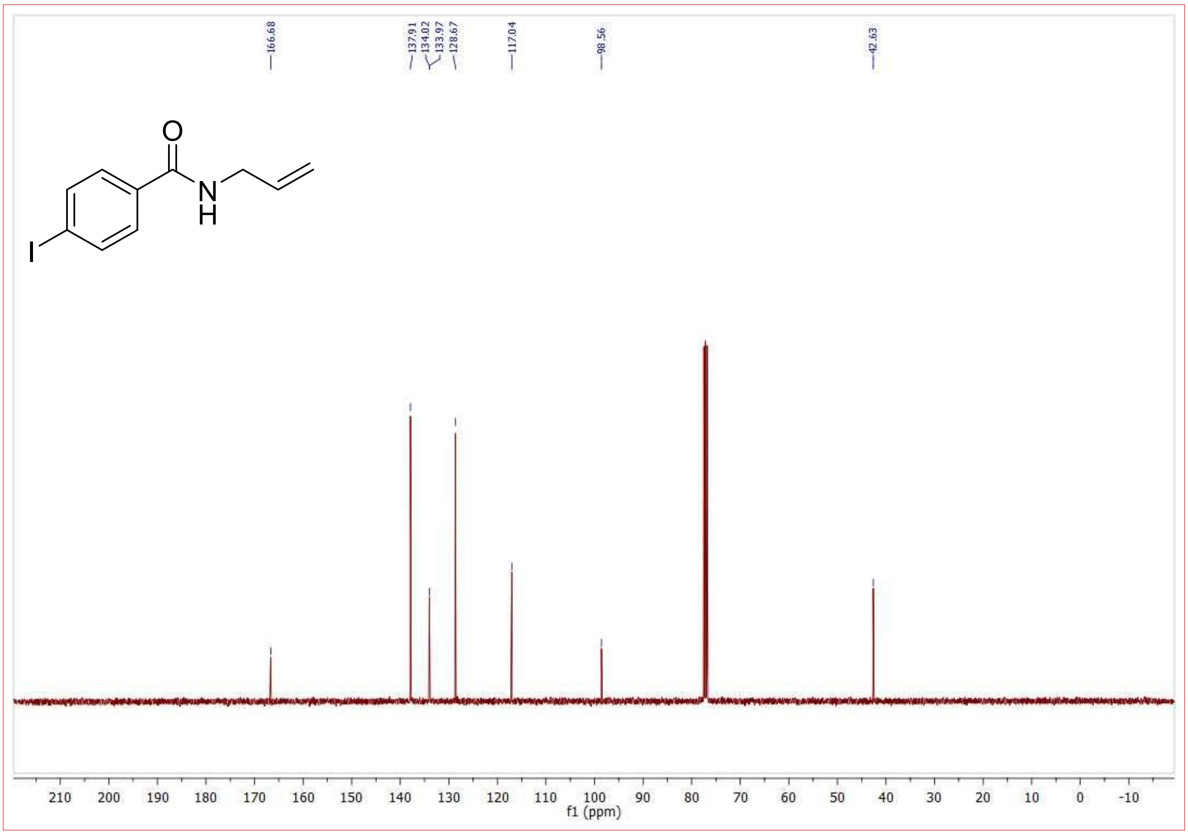

***N*-Allyl-4-isopropylbenzamide (1d), ^{1}H NMR (400 MHz, $CDCl_3$)**

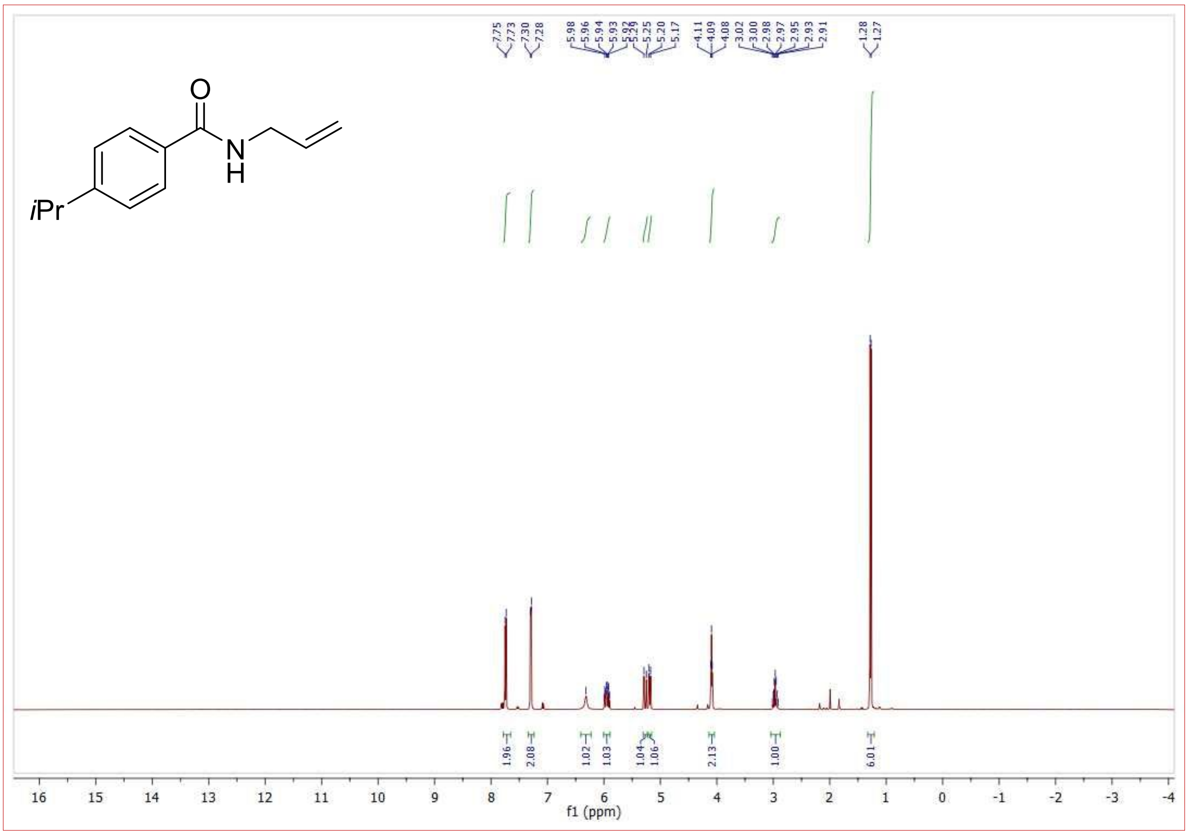

***N*-Allyl-4-isopropylbenzamide (1d), $^{13}C\{^{1}H\}$ NMR (101 MHz, $CDCl_3$)**

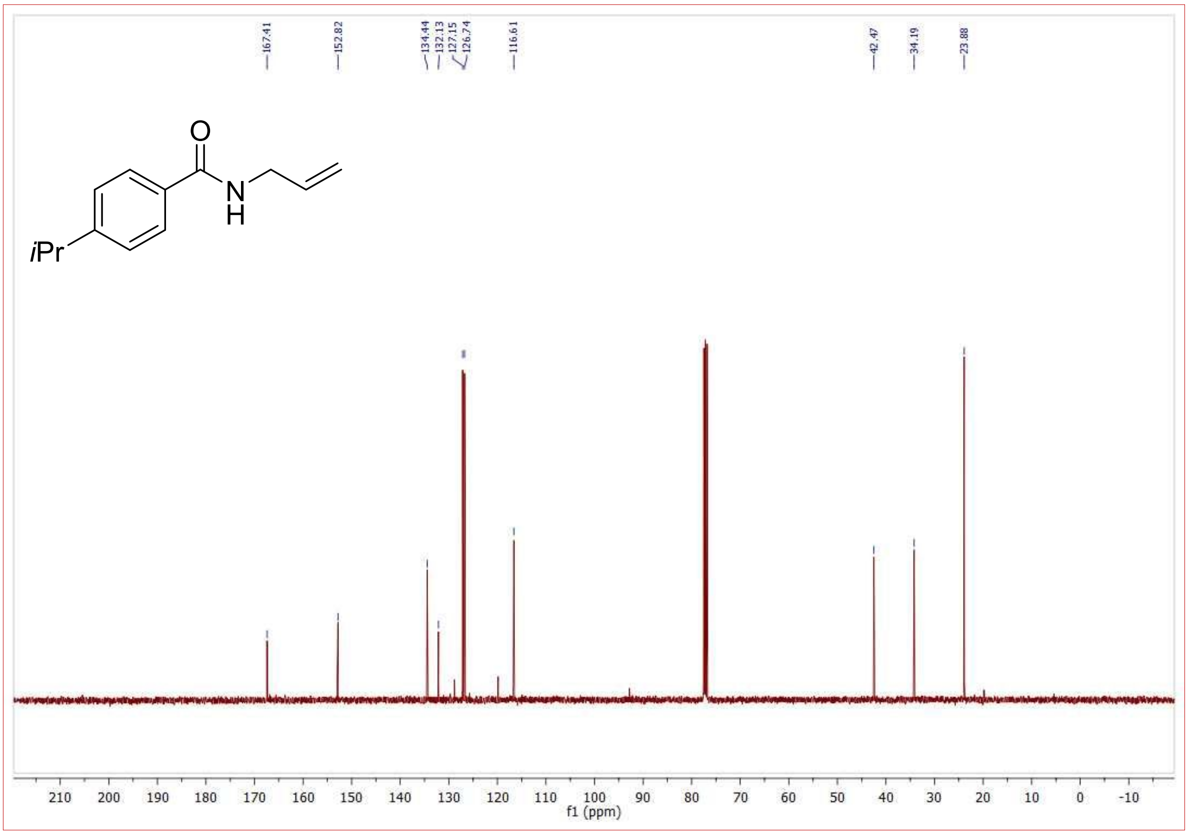

***N*-Allyl-4-isopropylbenzamide (1e), ^{1}H NMR (400 MHz, CDCl$_3$)**

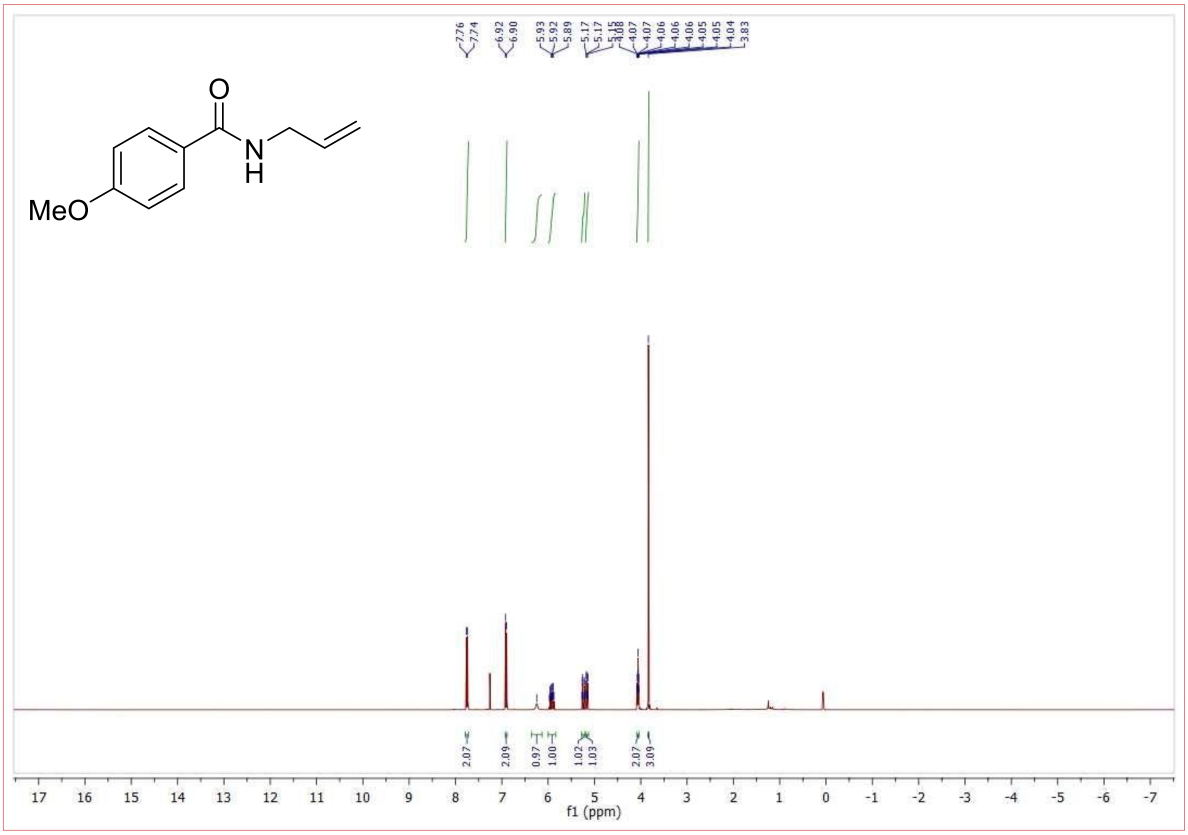

***N*-Allyl-4-isopropylbenzamide (1e), ^{13}C{^{1}H} NMR (101 MHz, CDCl$_3$)**

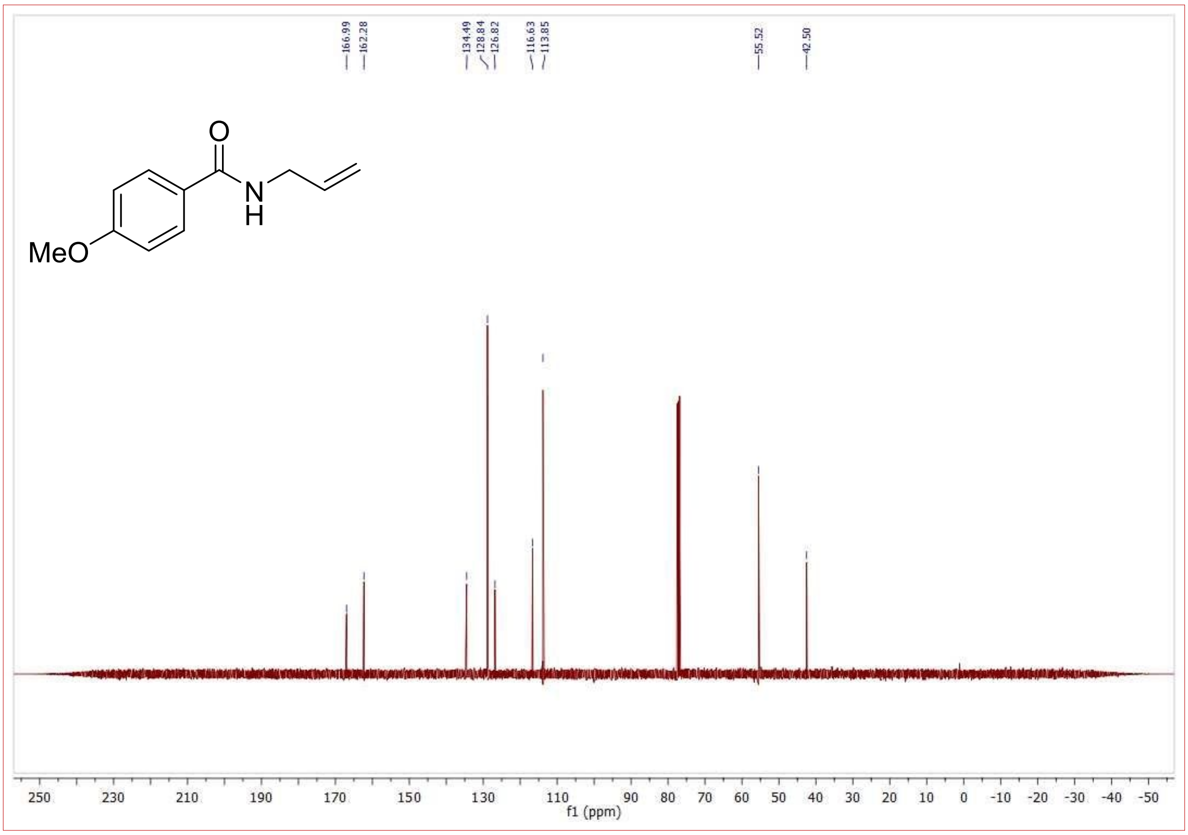

Methyl 4-(allylcarbamoyl)benzoate (1f), ^{1}H NMR (400 MHz, $CDCl_3$)

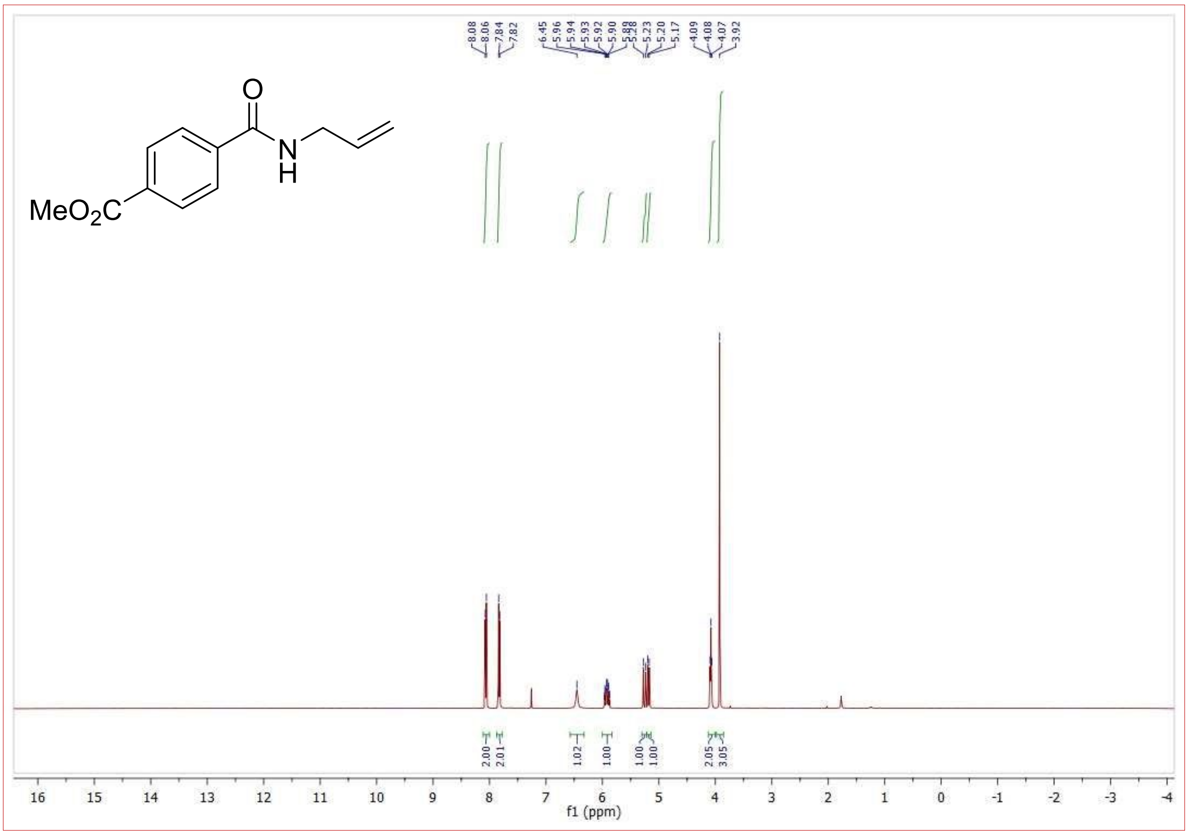

Methyl 4-(allylcarbamoyl)benzoate (1f), $^{13}C\{^{1}H\}$ NMR (101 MHz, $CDCl_3$)

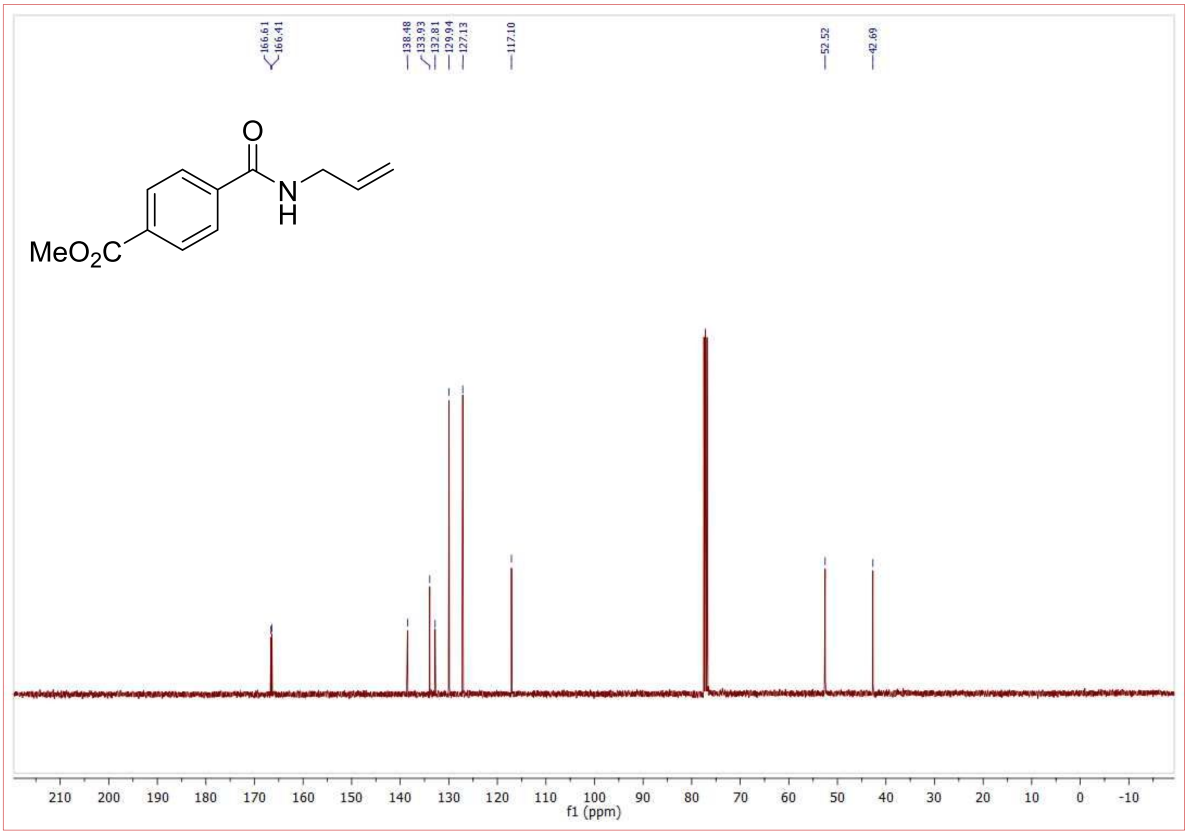

***N*-Allyl-3-cyanobenzamide (1g), ^{1}H NMR (400 MHz, $CDCl_3$)**

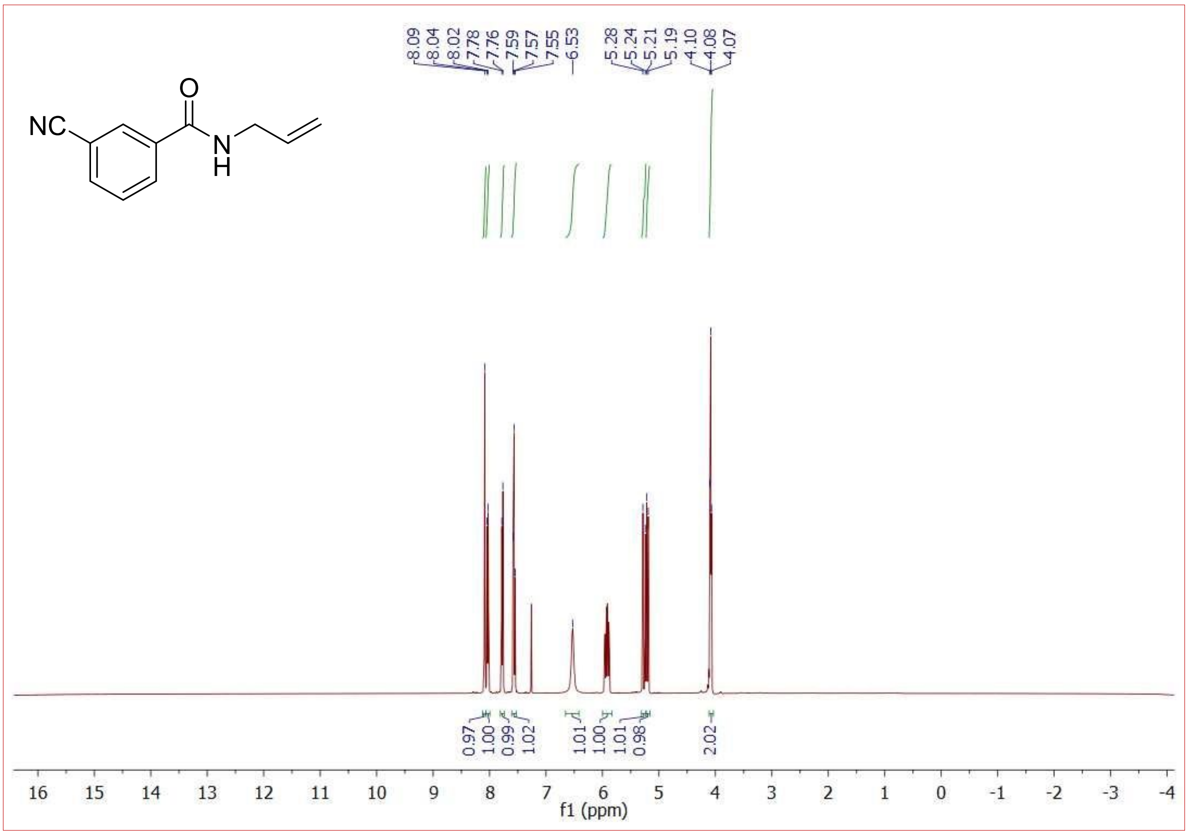

***N*-Allyl-3-cyanobenzamide (1g), ^{13}C{^{1}H} NMR (101 MHz, $CDCl_3$)**

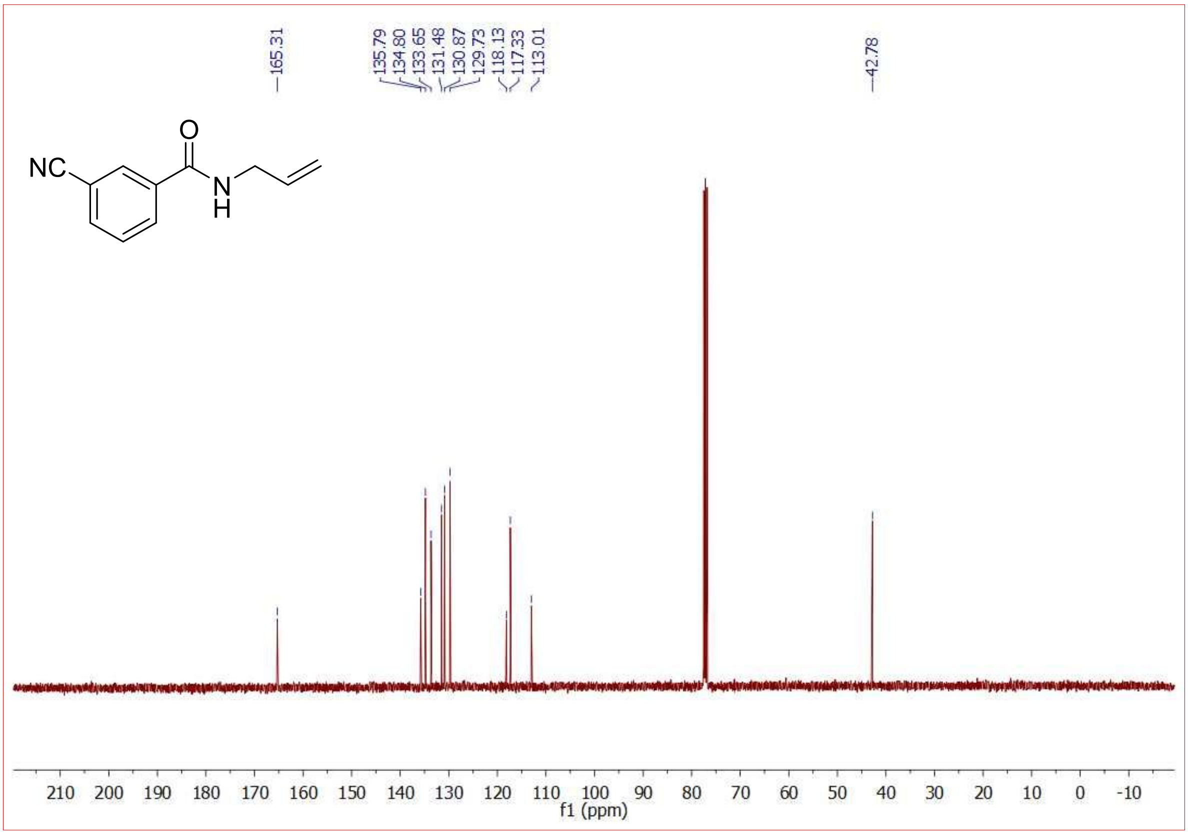

N-Allyl-3-(trifluoromethyl)benzamide (1h), ^{1}H NMR (400 MHz, $CDCl_3$)

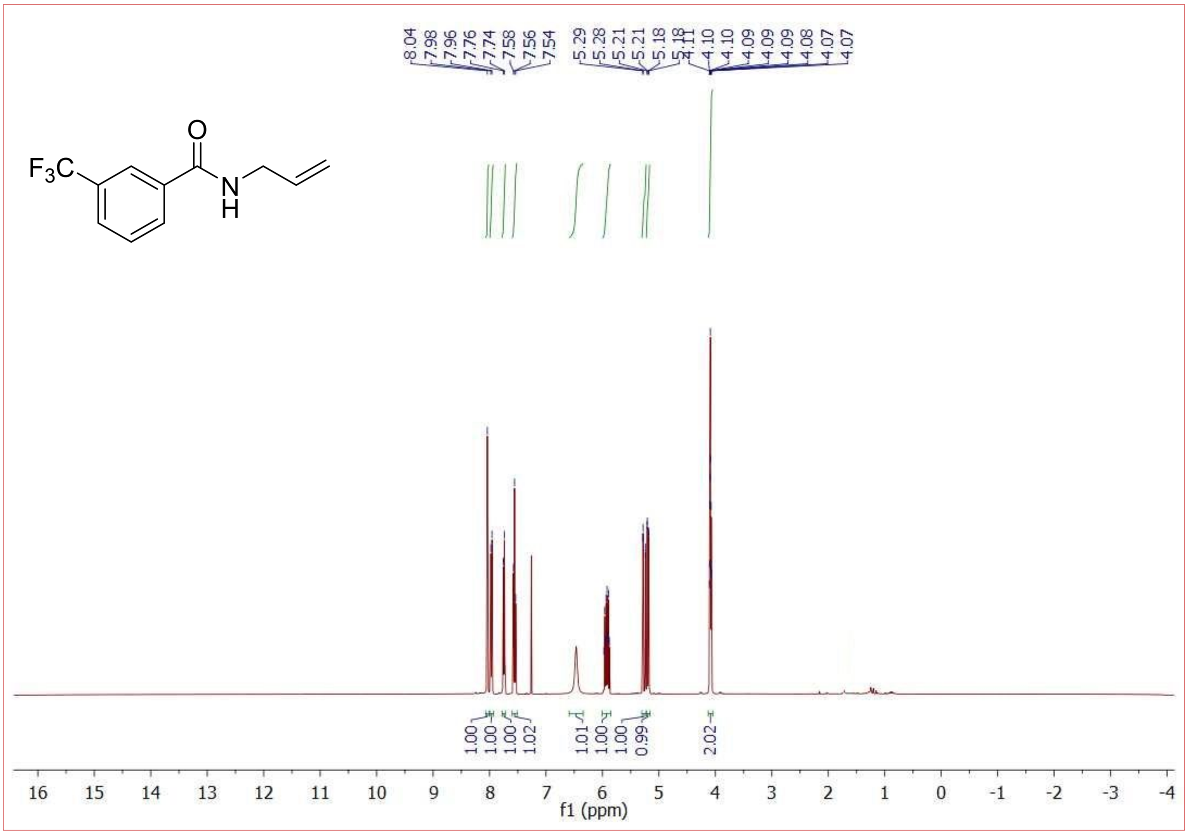

N-Allyl-3-(trifluoromethyl)benzamide (1h), $^{13}C\{^{1}H\}$ NMR (101 MHz, $CDCl_3$)

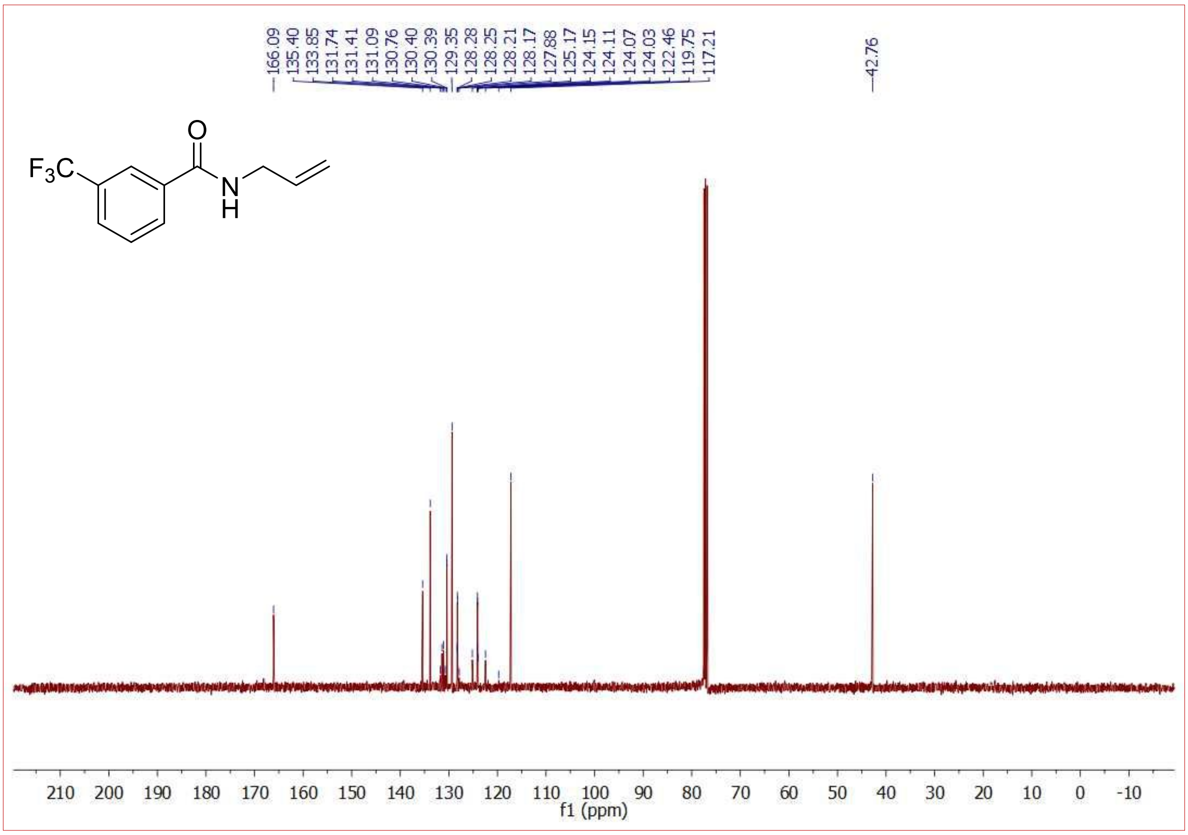

N-Allyl-3-nitrobenzamide (1i), ^{1}H NMR (400 MHz, $CDCl_3$)

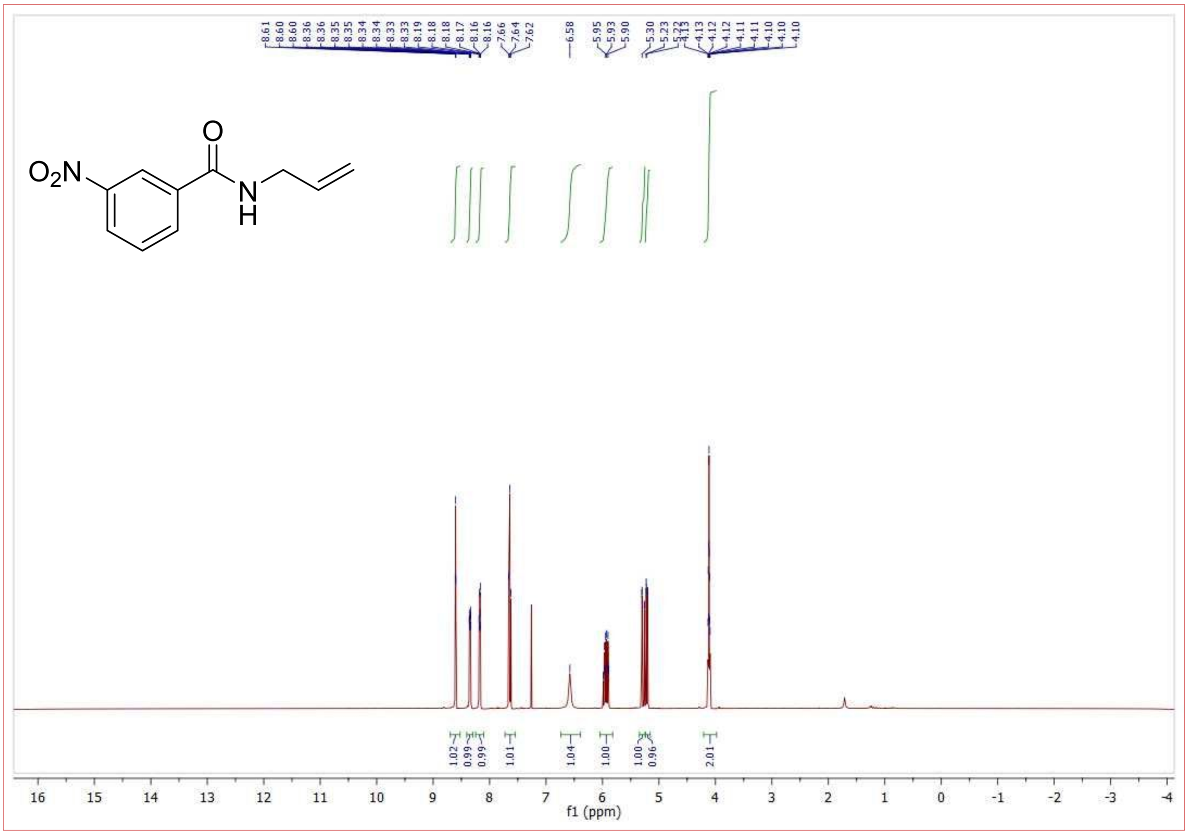

N-Allyl-3-nitrobenzamide (1i), ^{13}C{^{1}H} NMR (101 MHz, $CDCl_3$)

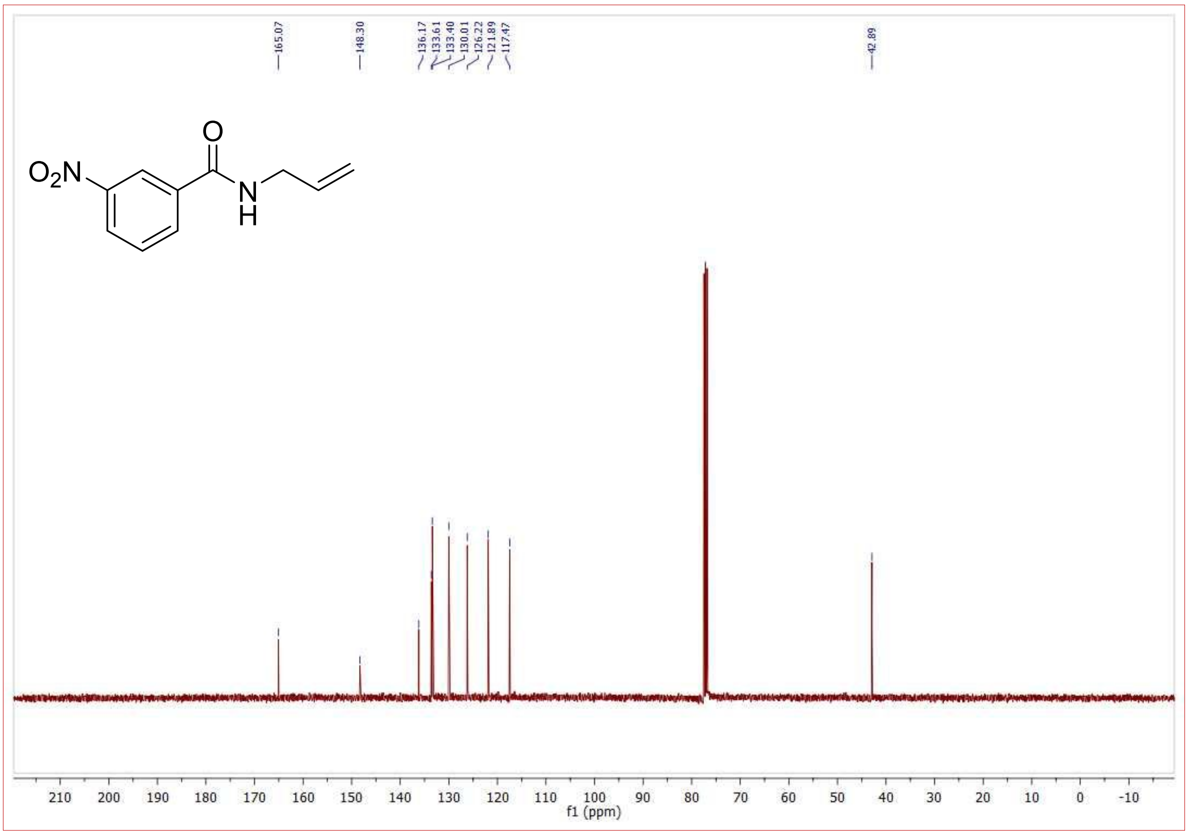

N-Allyl-2-methylbenzamide (1j), ^{1}H NMR (400 MHz, $CDCl_3$)

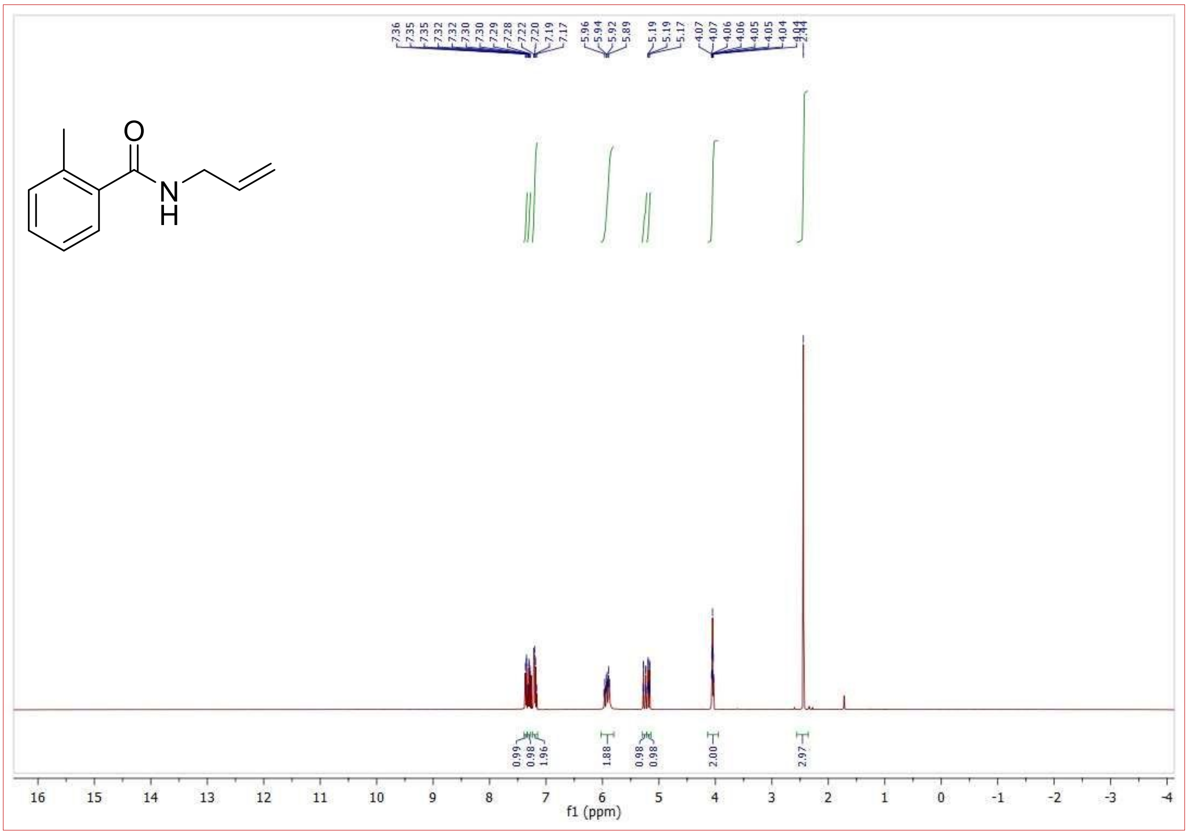

N-Allyl-2-methylbenzamide (1j), $^{13}C\{^{1}H\}$ NMR (101 MHz, $CDCl_3$)

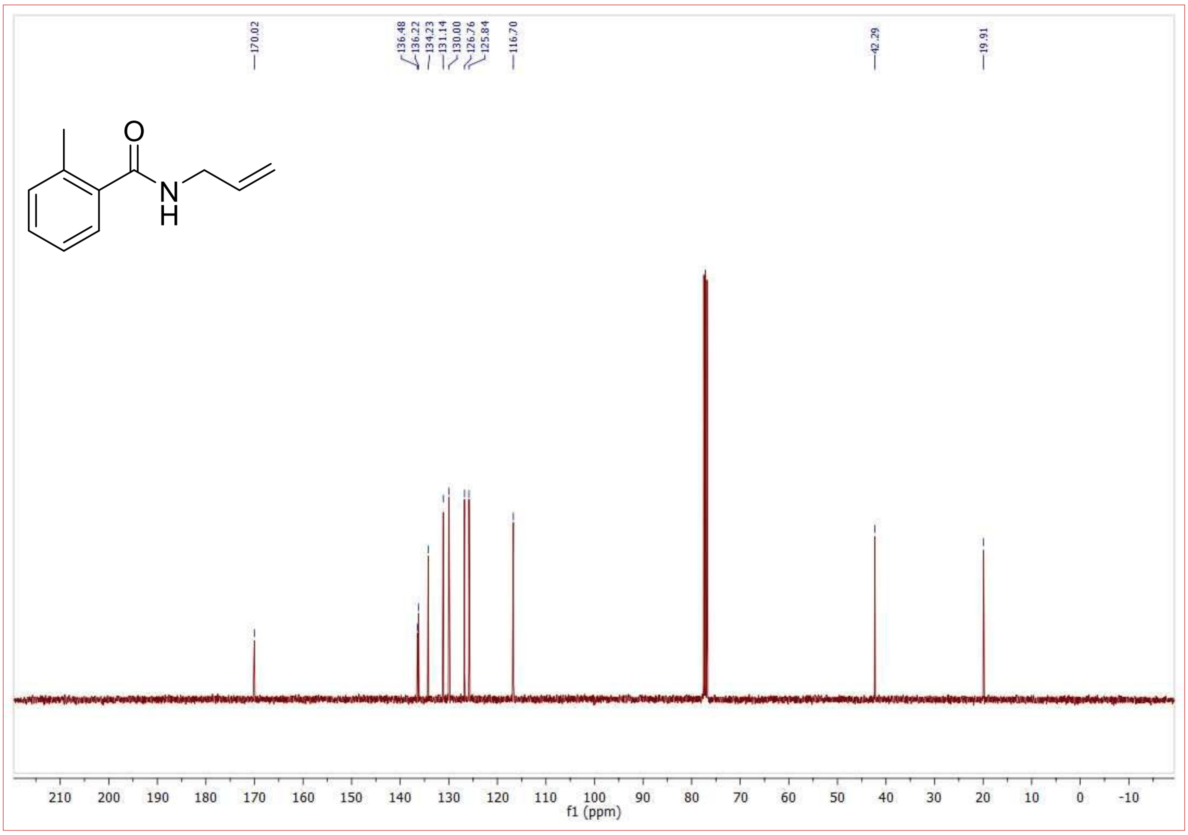

N-Allyl-[1,1'-biphenyl]-2-carboxamide (1k), ^{1}H NMR (400 MHz, $CDCl_3$)

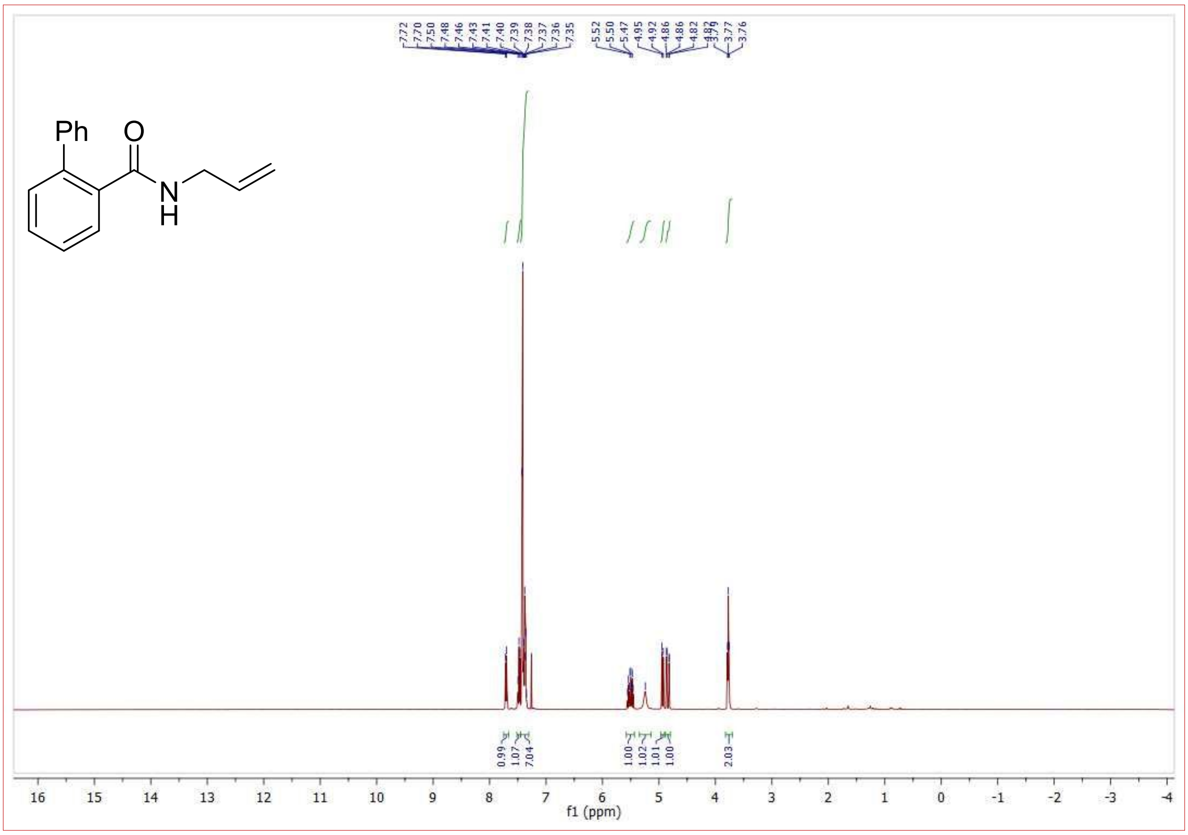

N-Allyl-[1,1'-biphenyl]-2-carboxamide (1k), $^{13}C\{^{1}H\}$ NMR (101 MHz, $CDCl_3$)

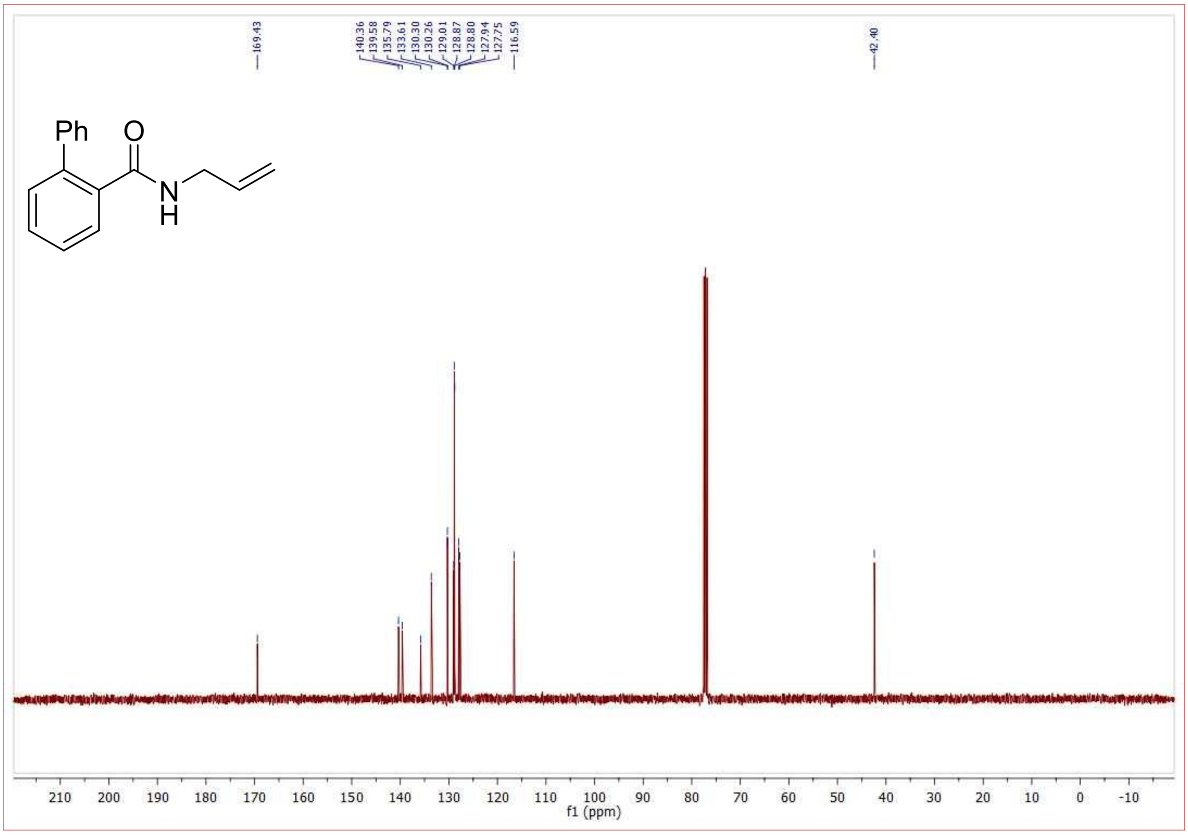

***N*-Allyl-2-bromobenzamide (1l), ^{1}H NMR (400 MHz, $CDCl_3$)**

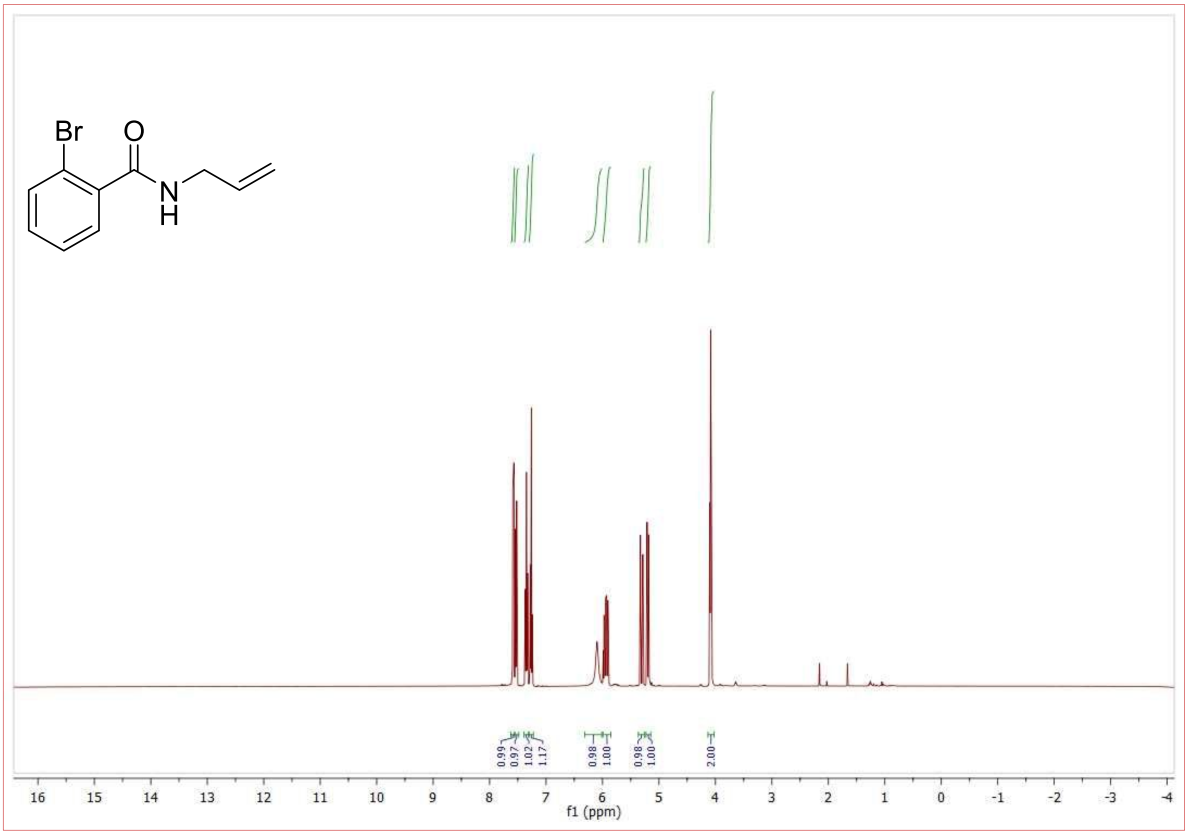

***N*-Allyl-2-bromobenzamide (1l), ^{13}C{^{1}H} NMR (101 MHz, $CDCl_3$)**

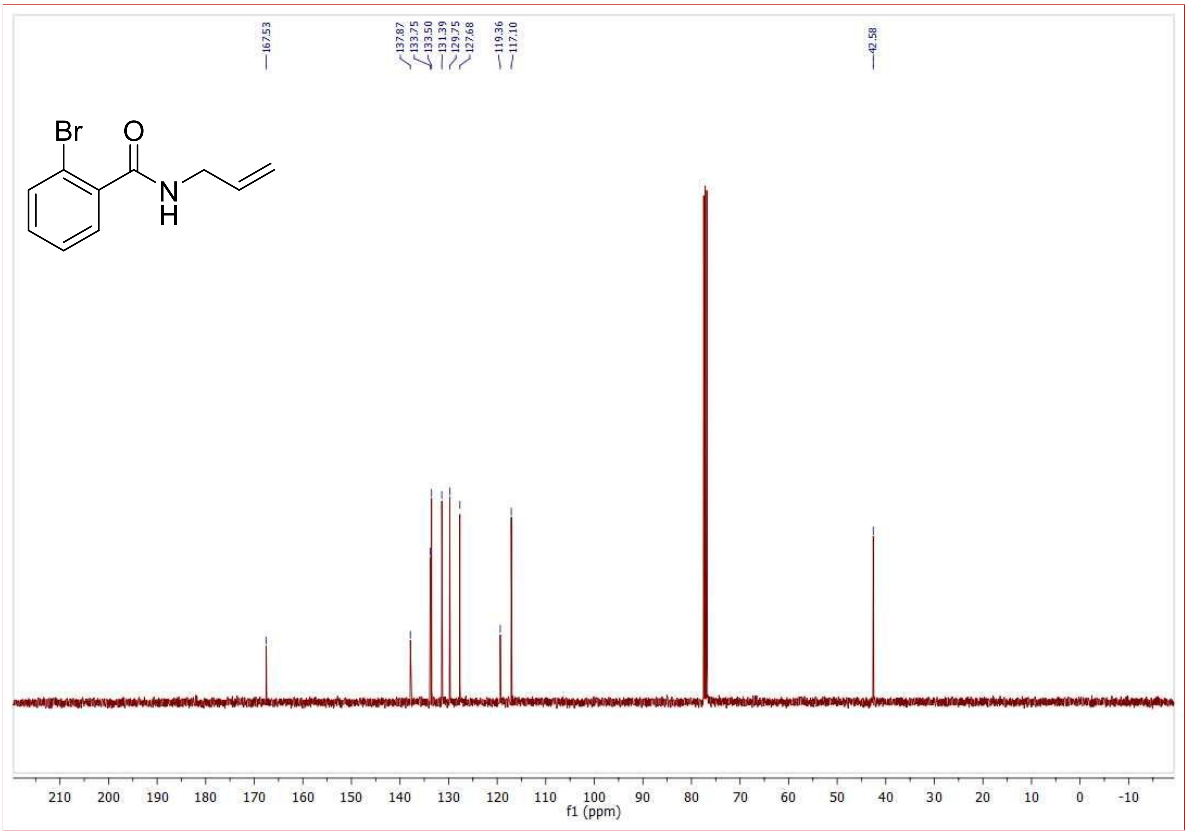

N-Allyl-2-chlorobenzamide (1m), ^{1}H NMR (400 MHz, $CDCl_3$)

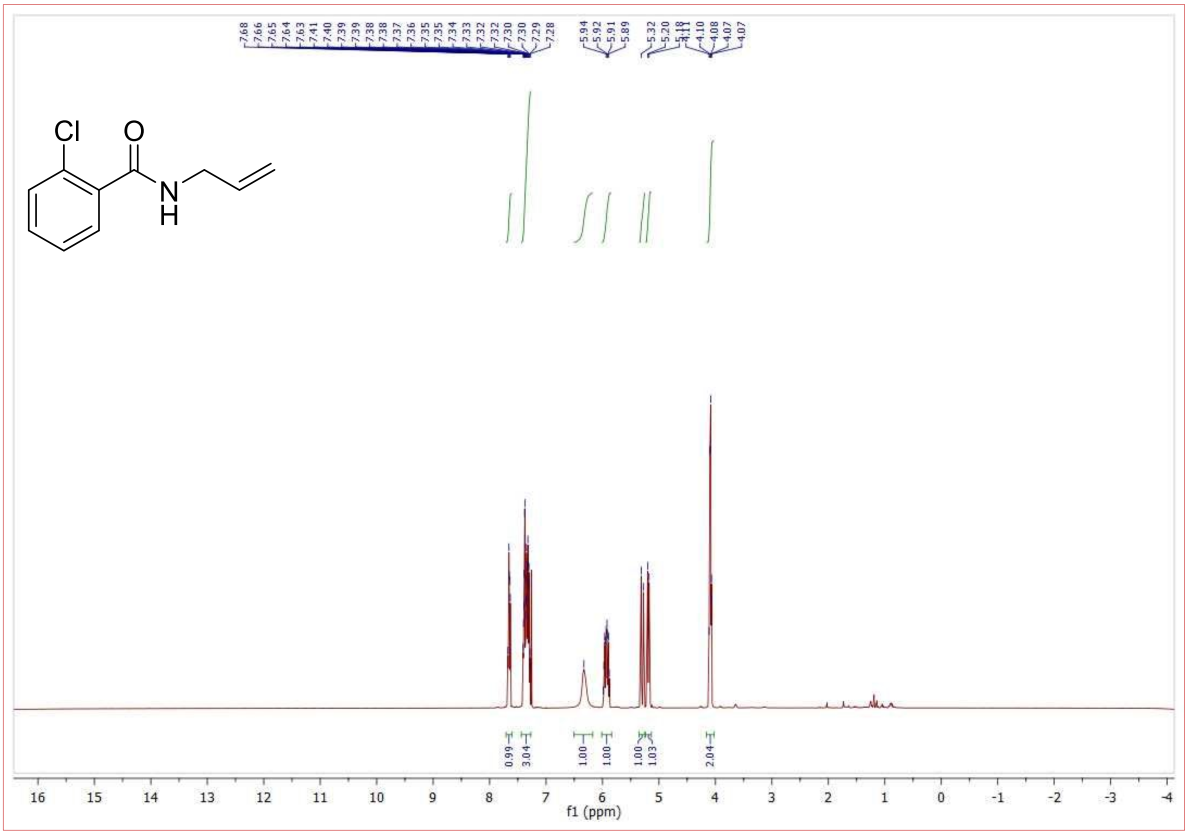

N-Allyl-2-chlorobenzamide (1m), $^{13}C\{^{1}H\}$ NMR (101 MHz, $CDCl_3$)

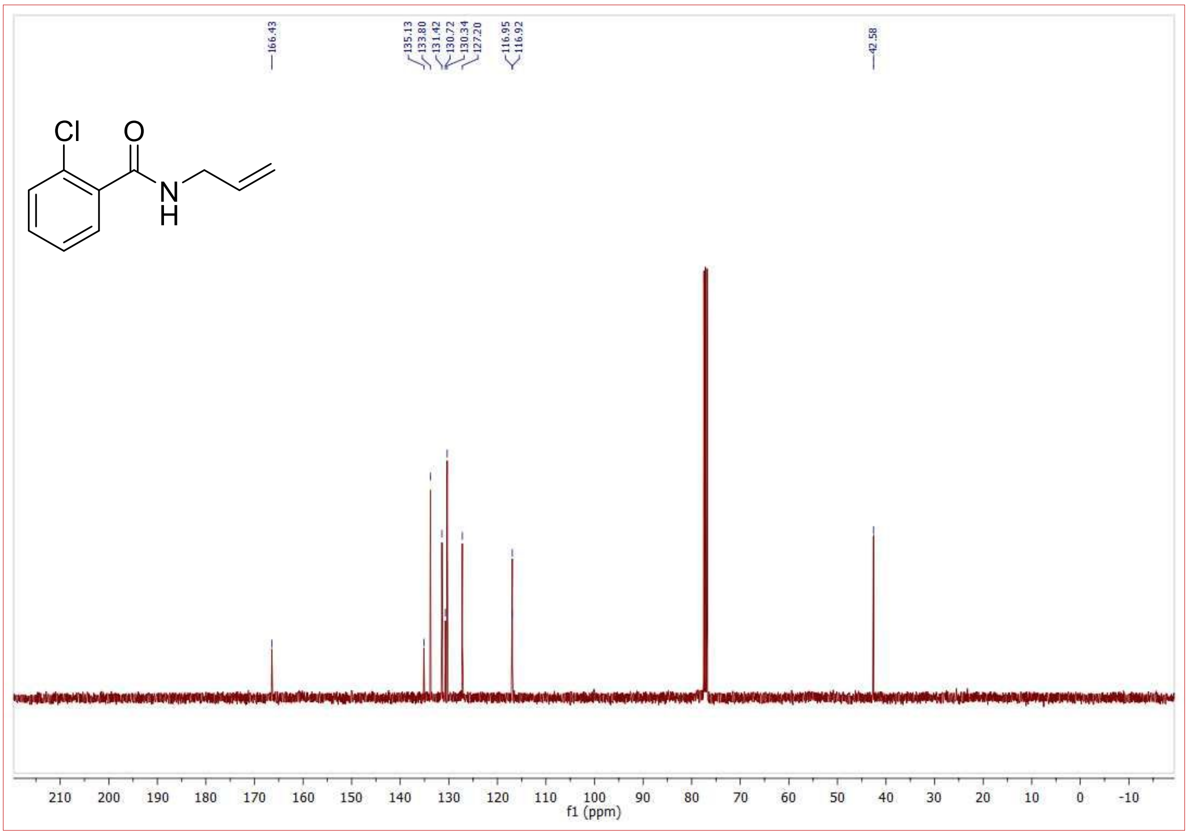

N-Allyl-2-fluorobenzamide (1n), ^{1}H NMR (400 MHz, $CDCl_3$)

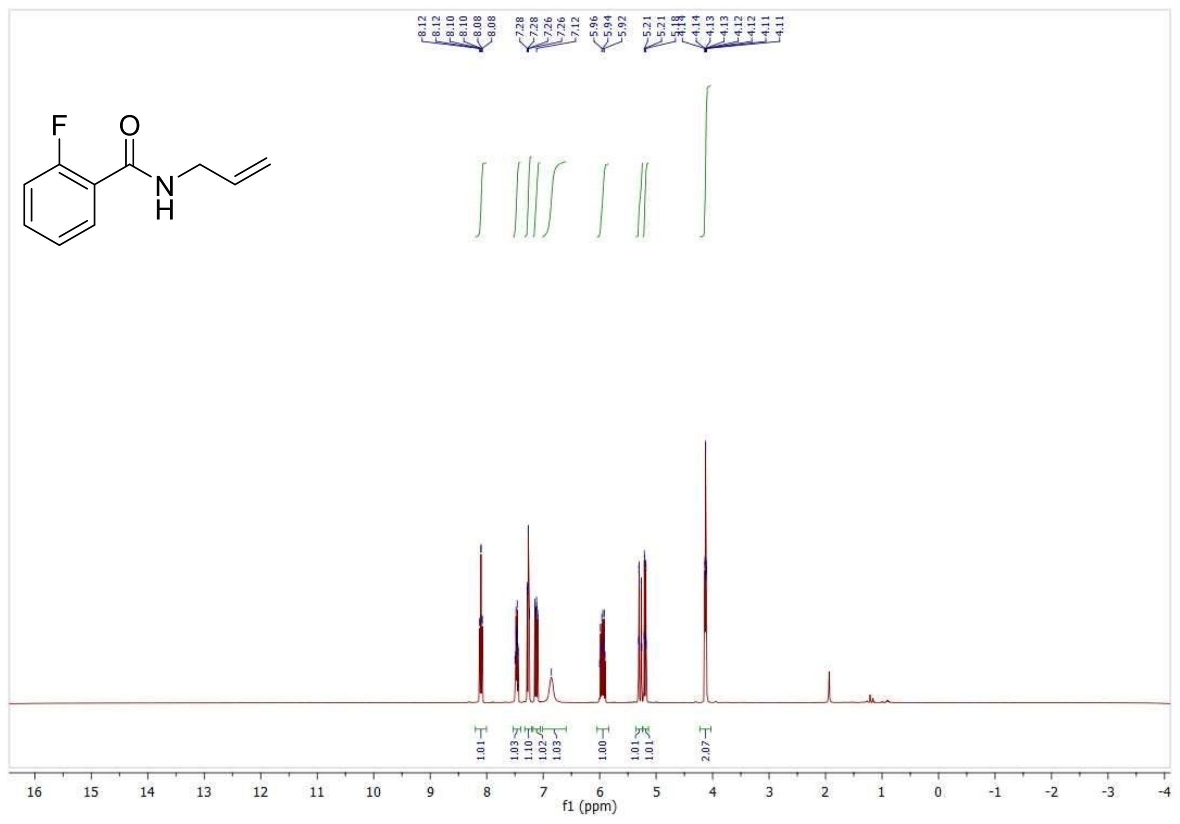

N-Allyl-2-fluorobenzamide (1n), $^{13}C\{^{1}H\}$ NMR (101 MHz, $CDCl_3$)

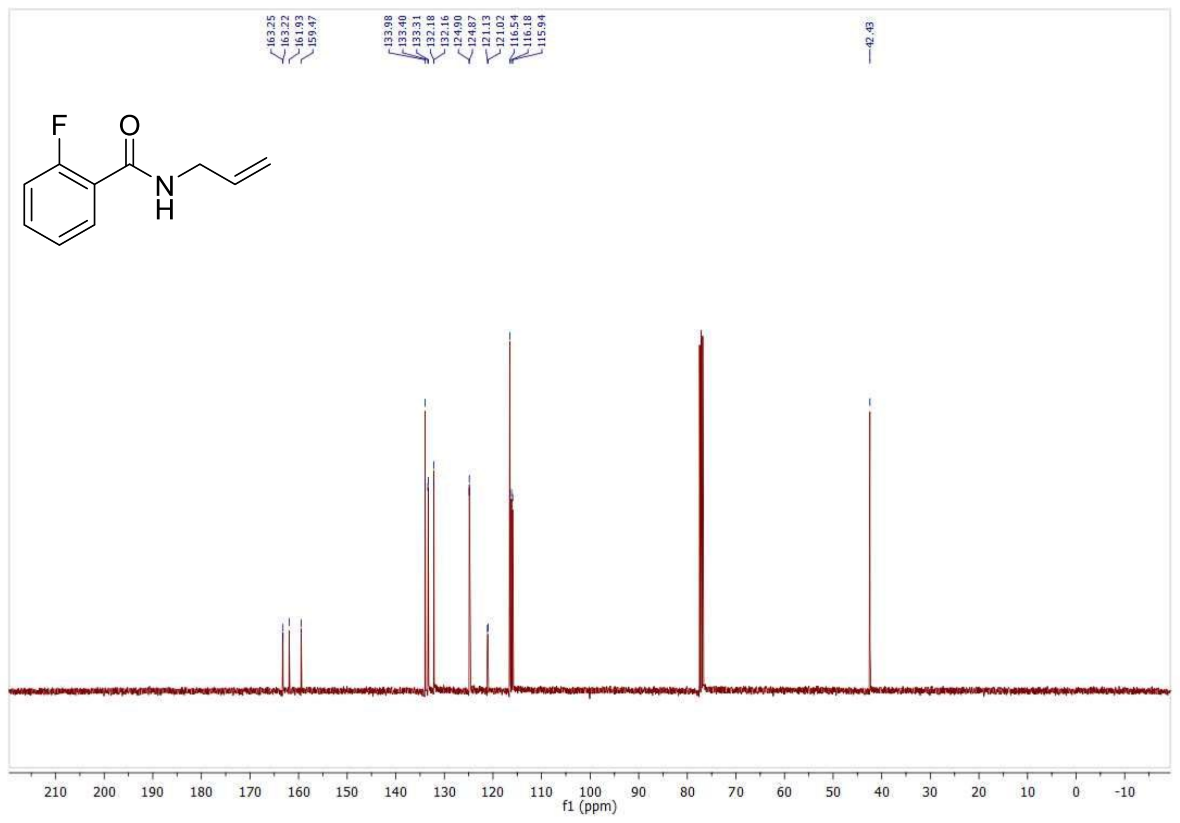

N-(2-Methylallyl)benzamide (1o), ^{1}H NMR (400 MHz, $CDCl_3$)

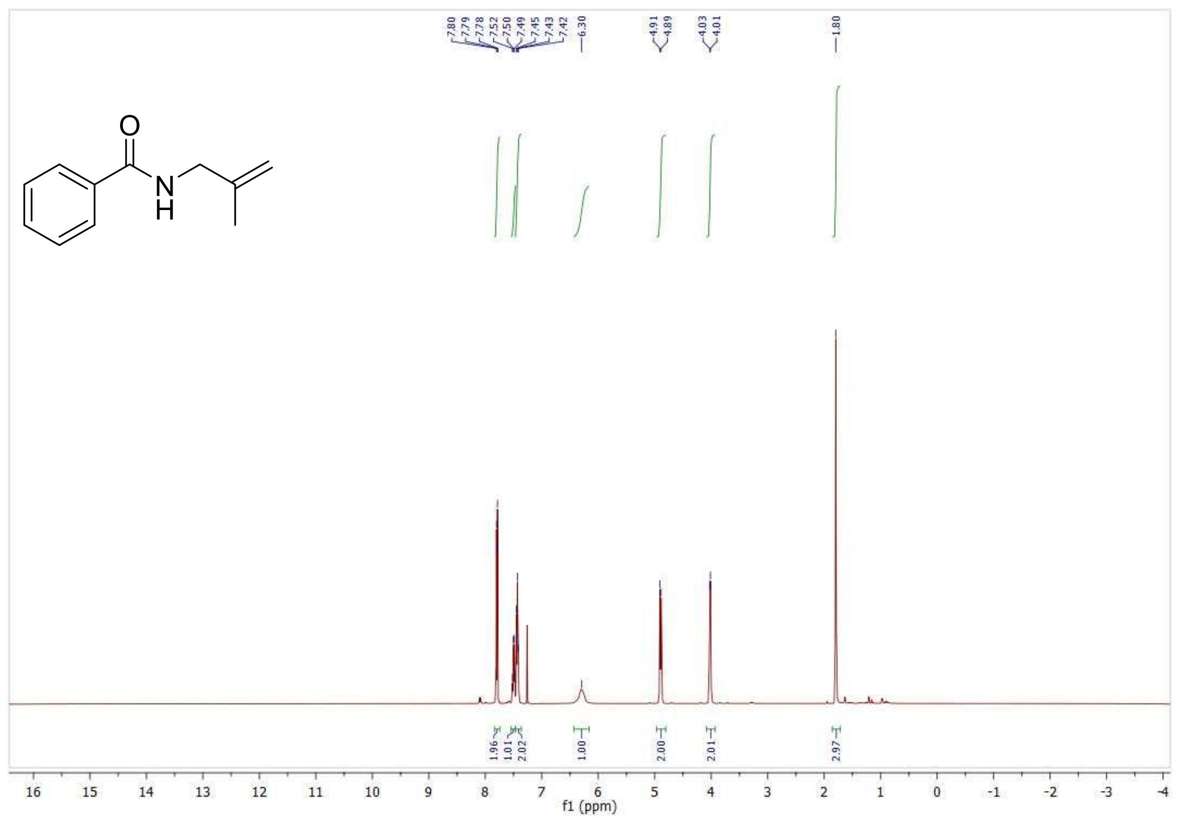

N-(2-Methylallyl)benzamide (1o), $^{13}C\{^{1}H\}$ NMR (101 MHz, $CDCl_3$)

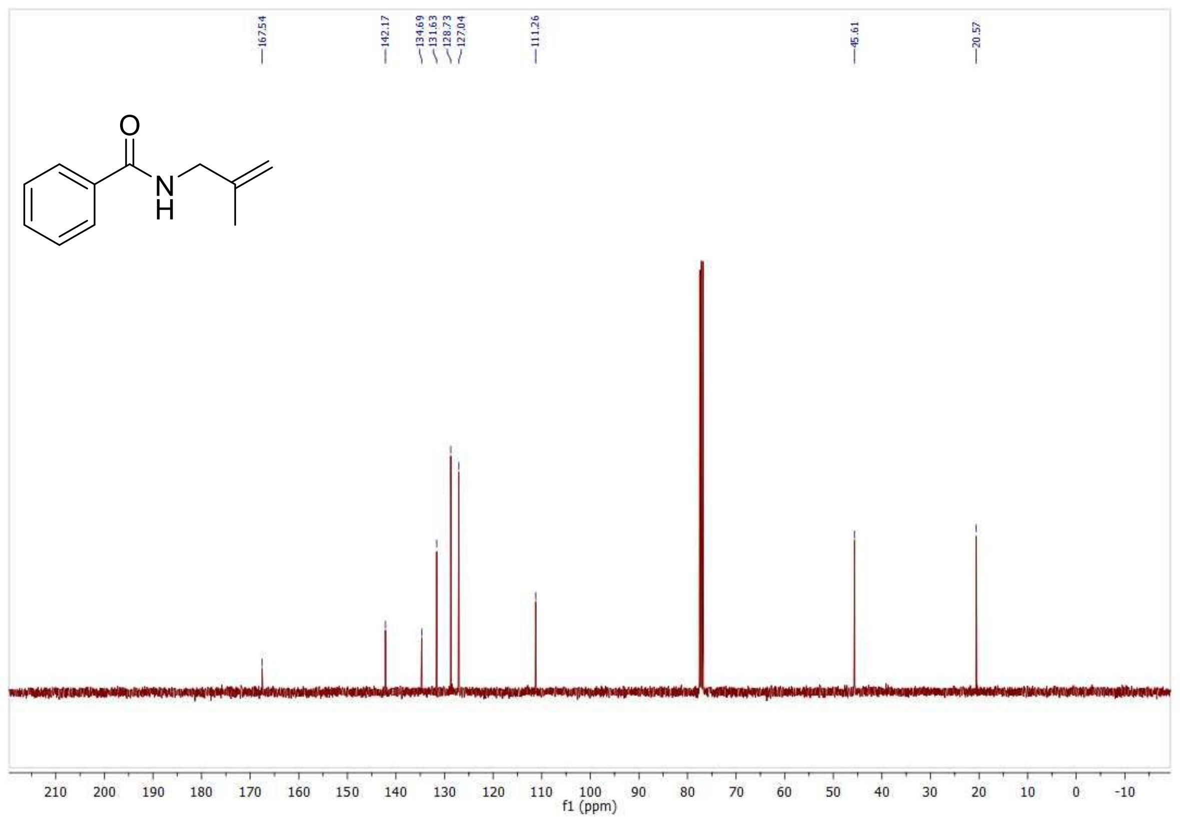

4-Chloro-*N*-(2-methylallyl)benzamide (1p), ^{1}H NMR (400 MHz, $CDCl_3$)

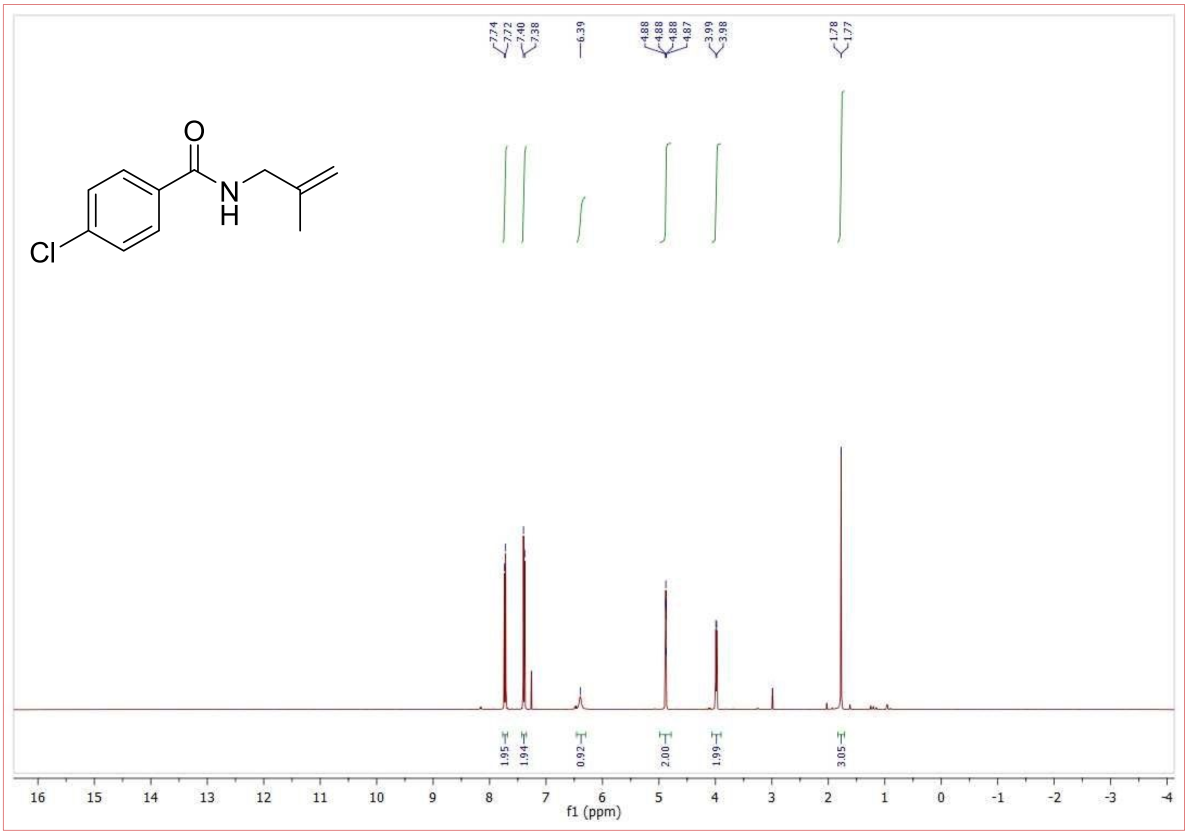

4-Chloro-*N*-(2-methylallyl)benzamide (1p), $^{13}C\{^{1}H\}$ NMR (101 MHz, $CDCl_3$)

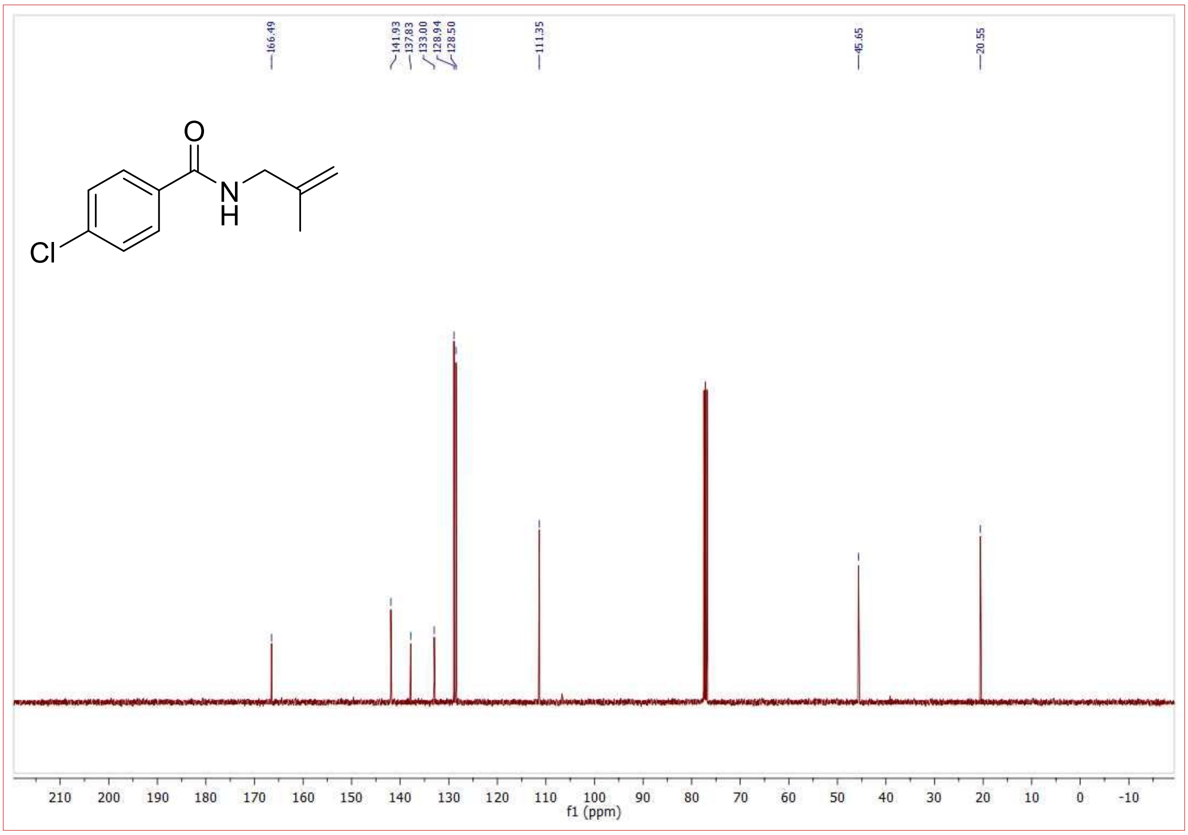

N-Allyl-2-fluoro-4-methoxybenzamide (1q), ^{1}H NMR (400 MHz, $CDCl_3$)

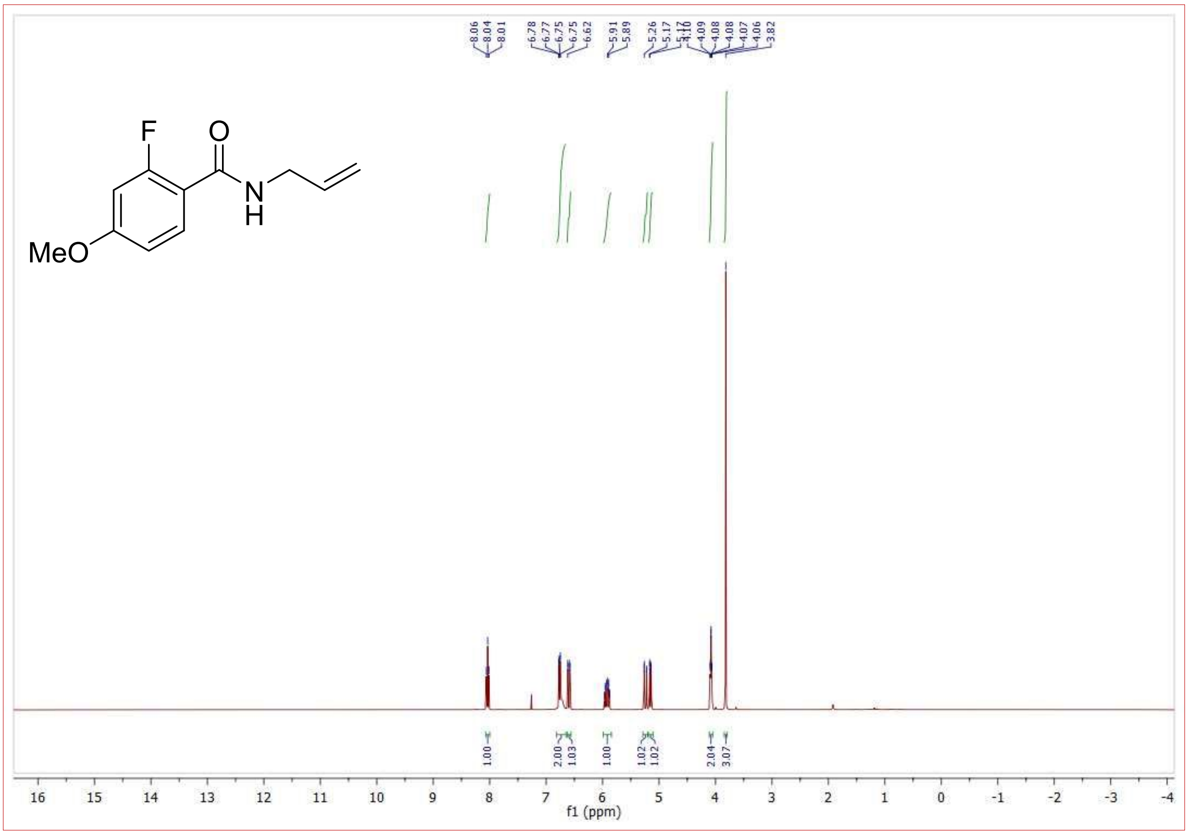

N-Allyl-2-fluoro-4-methoxybenzamide (1q), $^{13}C\{^{1}H\}$ NMR (101 MHz, $CDCl_3$)

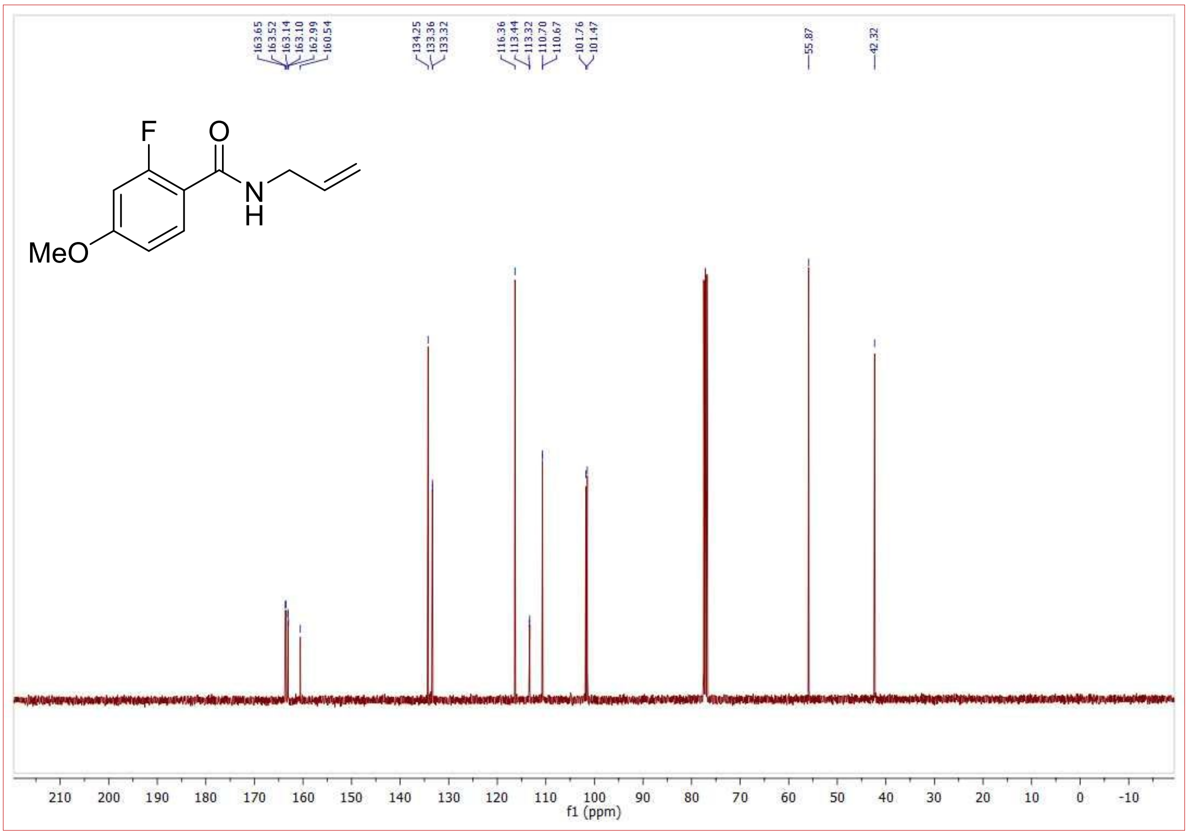

N-Allyl-2-fluoro-4-methoxybenzamide (1q), ^{19}F NMR (376 MHz, $CDCl_3$)

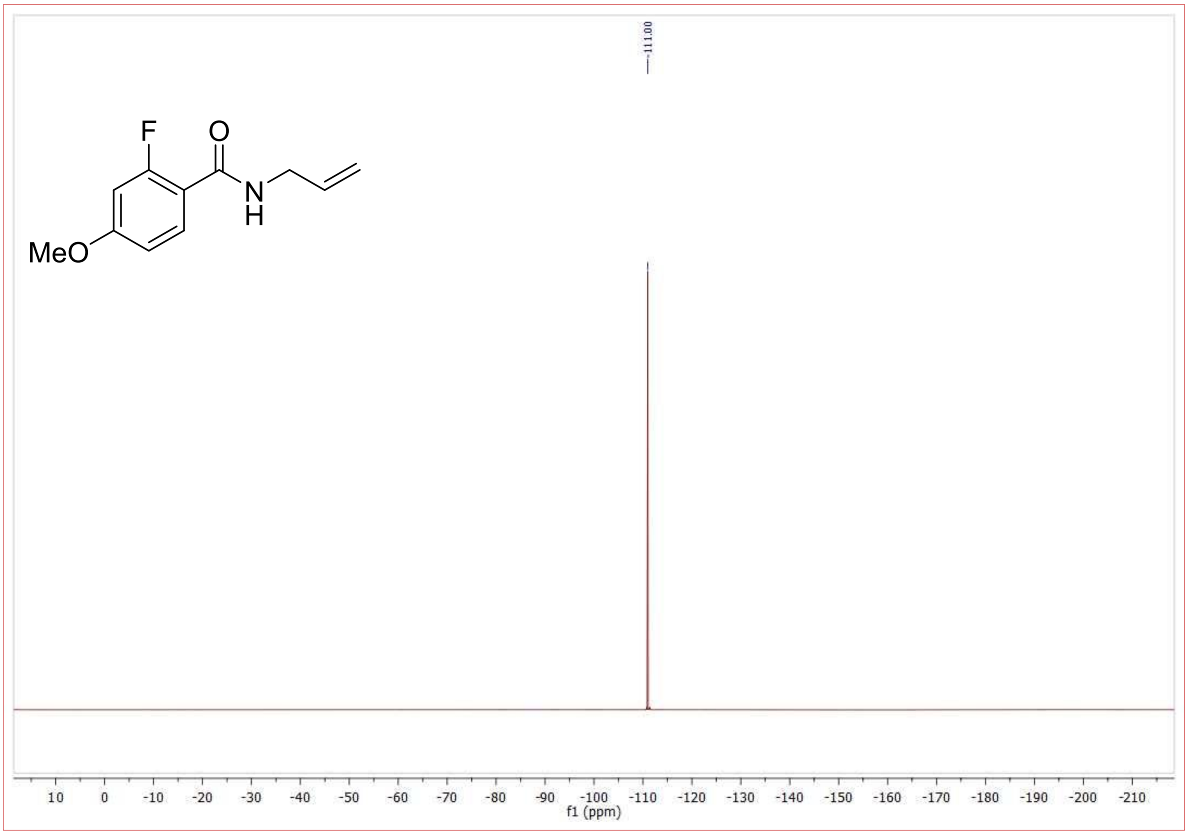

N-Allyl-3-fluoro-4-methylbenzamide (1r), ^{1}H NMR (400 MHz, $CDCl_3$)

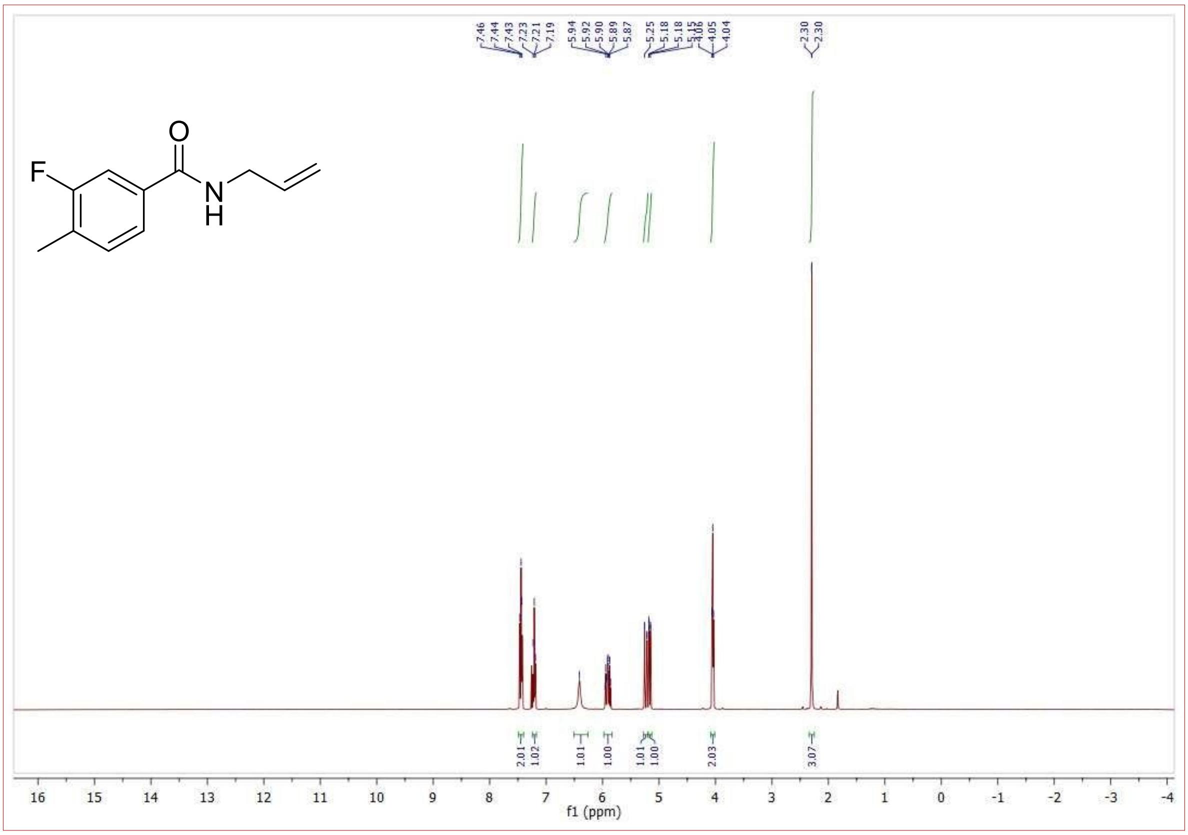

N-Allyl-3-fluoro-4-methylbenzamide (1r), $^{13}C\{^{1}H\}$ NMR (101 MHz, $CDCl_3$)

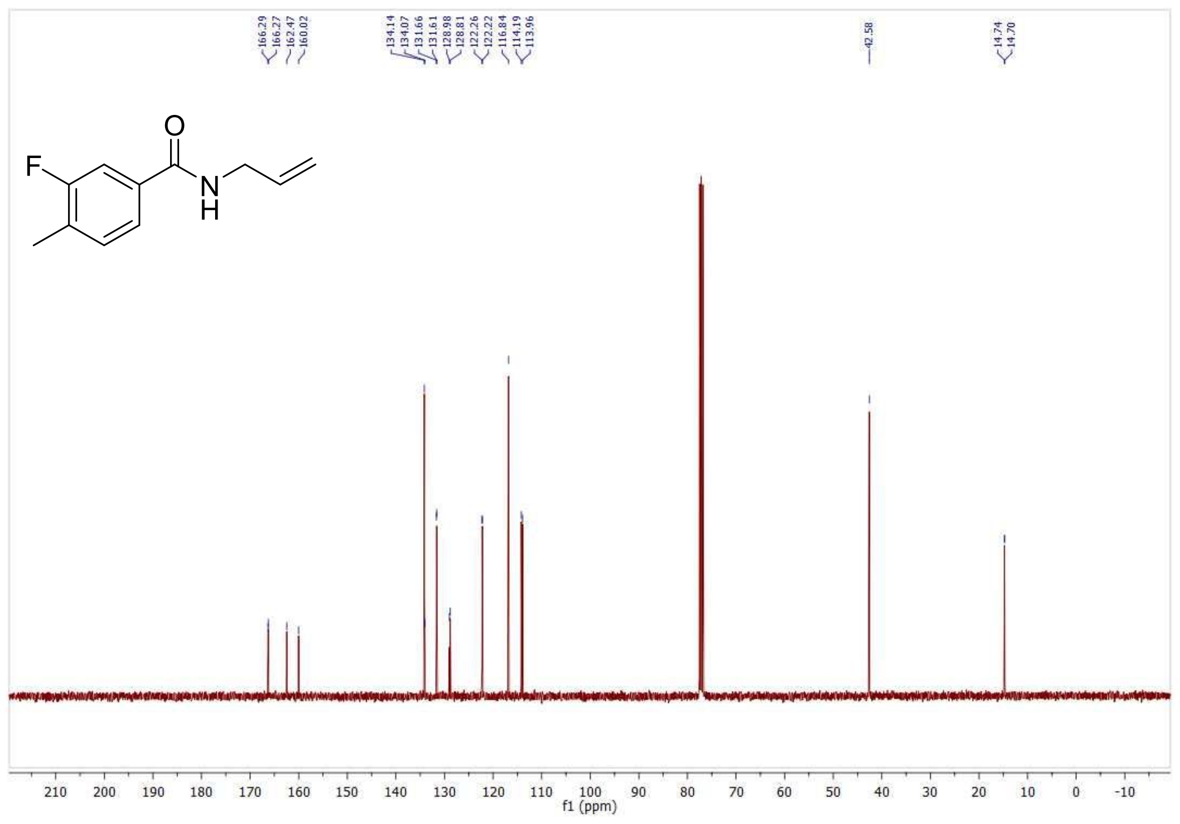

N-Allyl-3-fluoro-4-methylbenzamide (1r), ^{19}F NMR (376 MHz, $CDCl_3$)

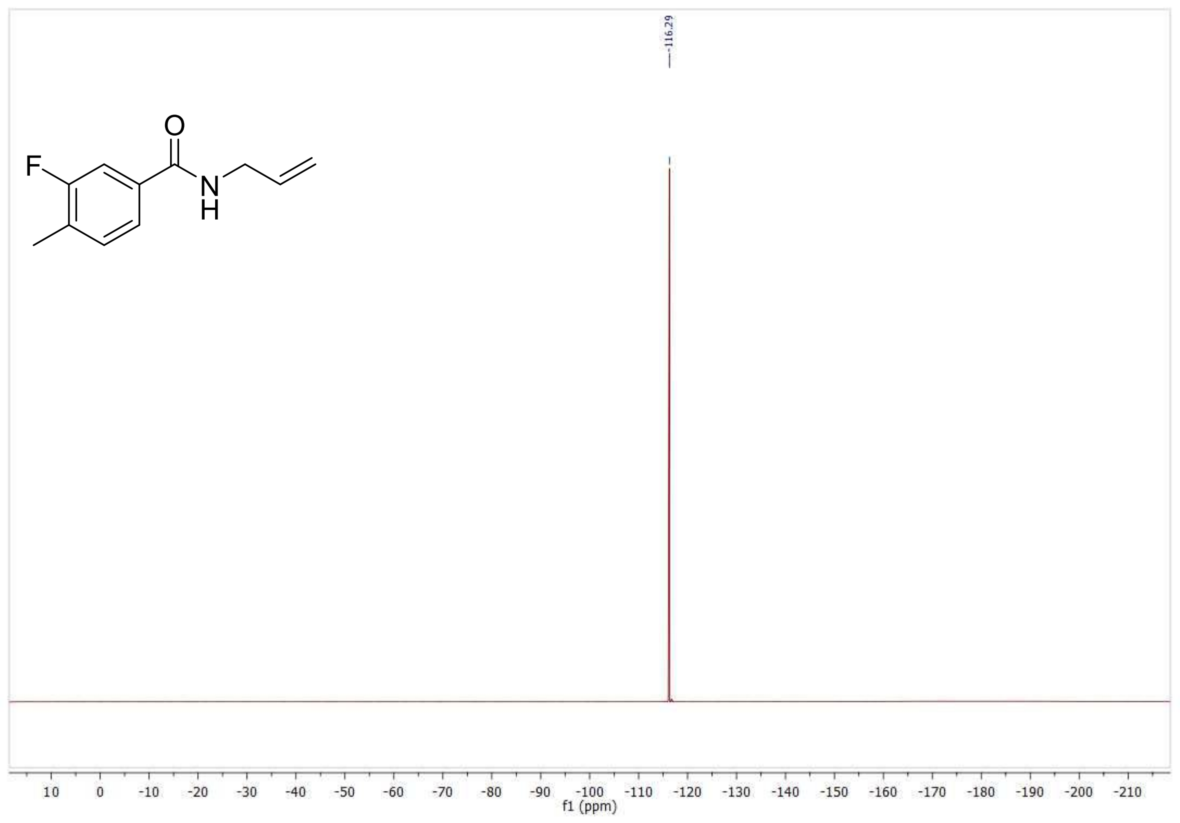

N-Allyl-2-phenylacetamide (1s), ^{1}H NMR (400 MHz, $CDCl_3$)

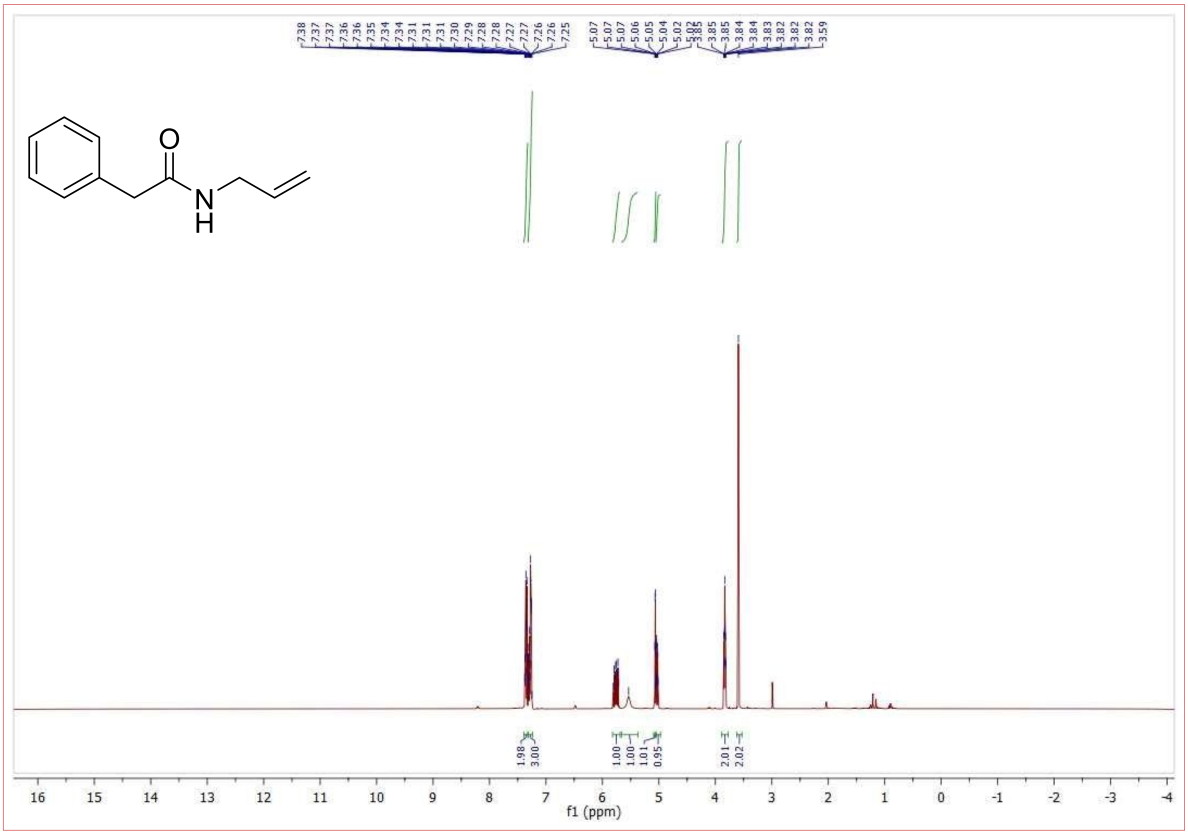

N-Allyl-2-phenylacetamide (1s), $^{13}C\{^{1}H\}$ NMR (101 MHz, $CDCl_3$)

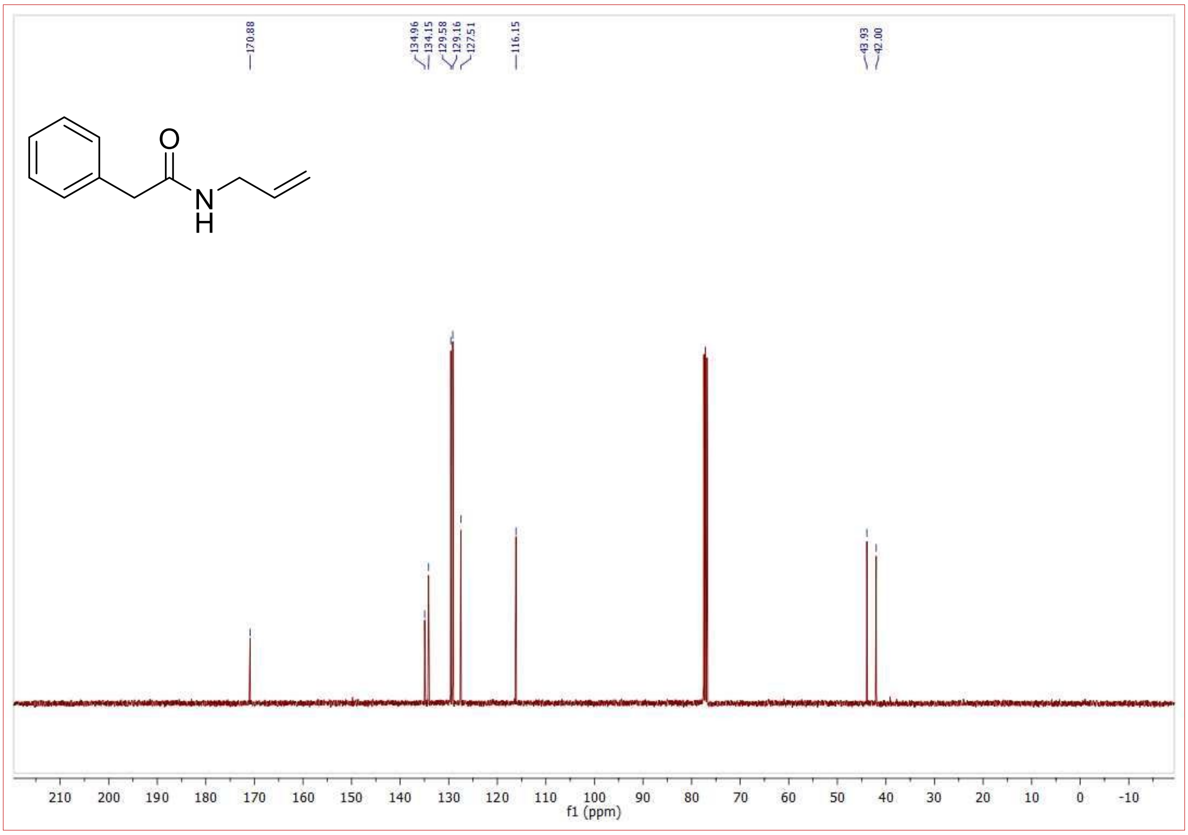

N-Allyl-2-(4-chlorophenyl)acetamide (1t), ^{1}H NMR (400 MHz, $CDCl_3$)

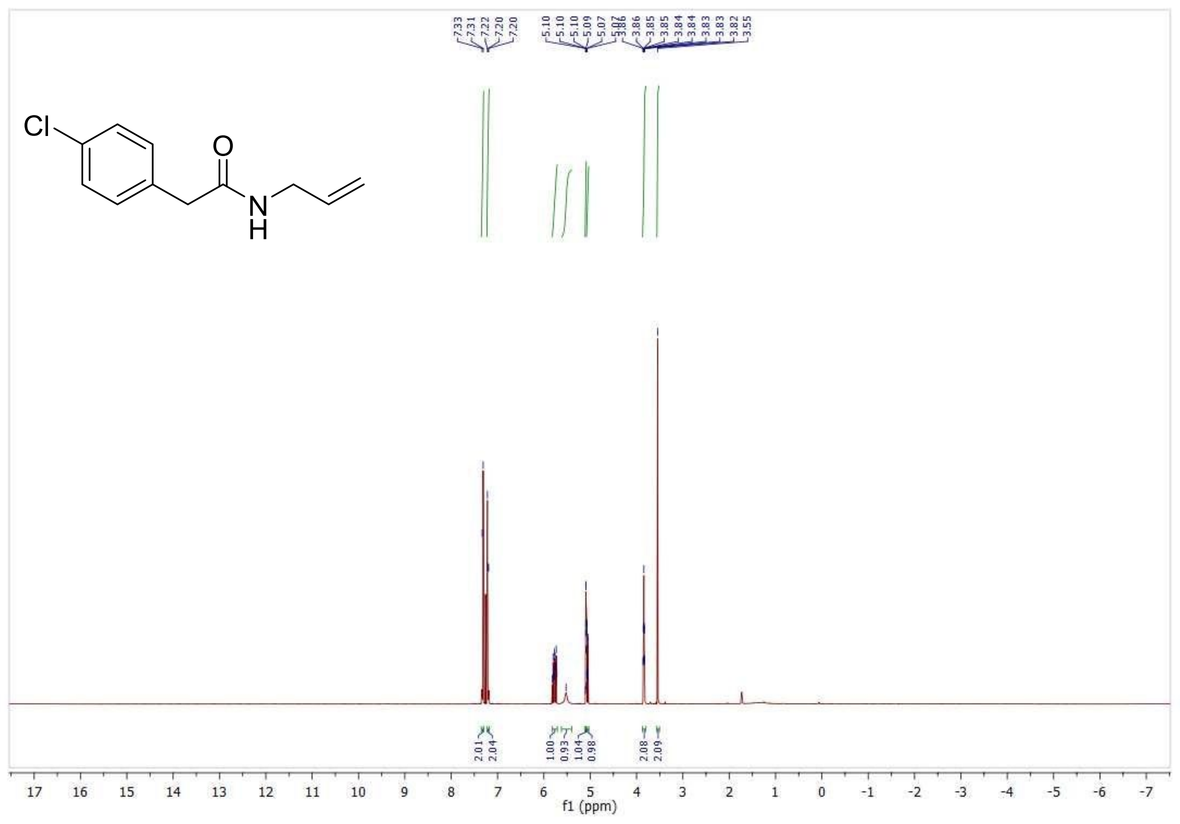

N-Allyl-2-(4-chlorophenyl)acetamide (1t), $^{13}C\{^1H\}$ NMR (101 MHz, $CDCl_3$)

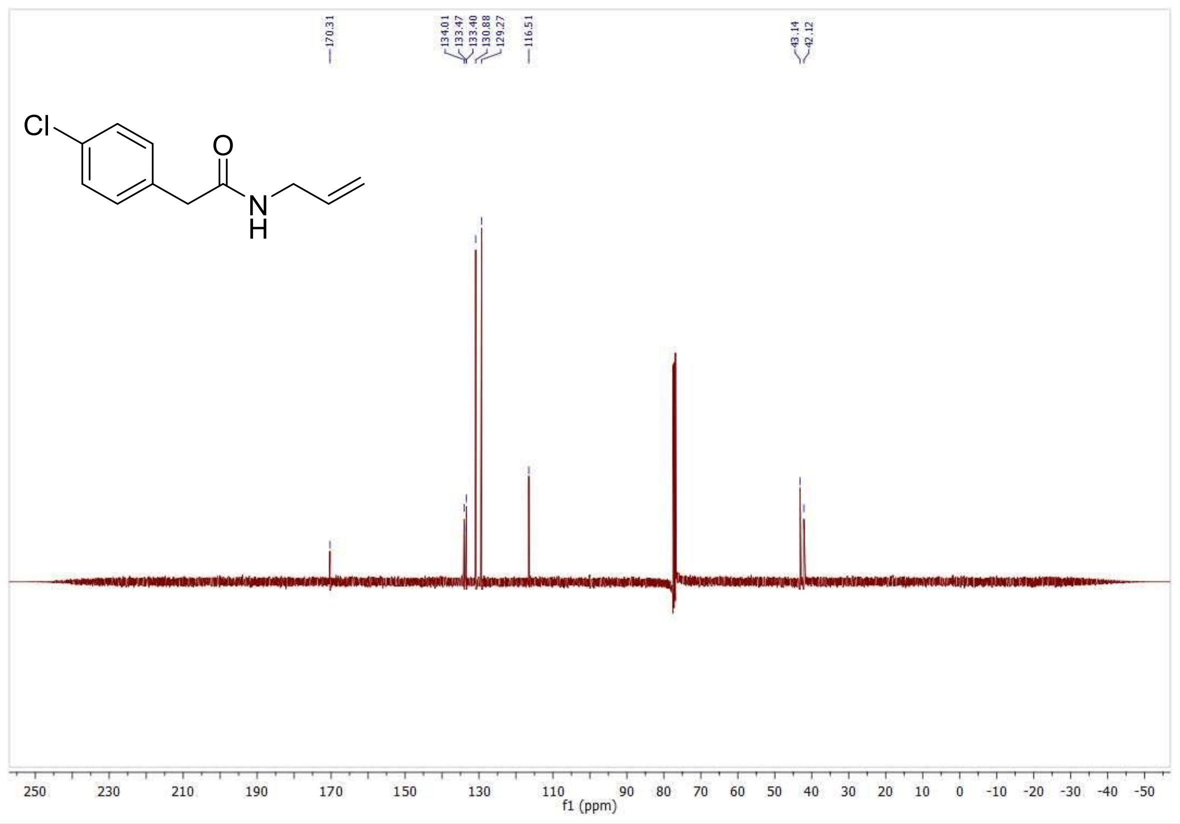

N-Allylcyclohexanecarboxamide (1u), ^{1}H NMR (400 MHz, CDCl$_3$)

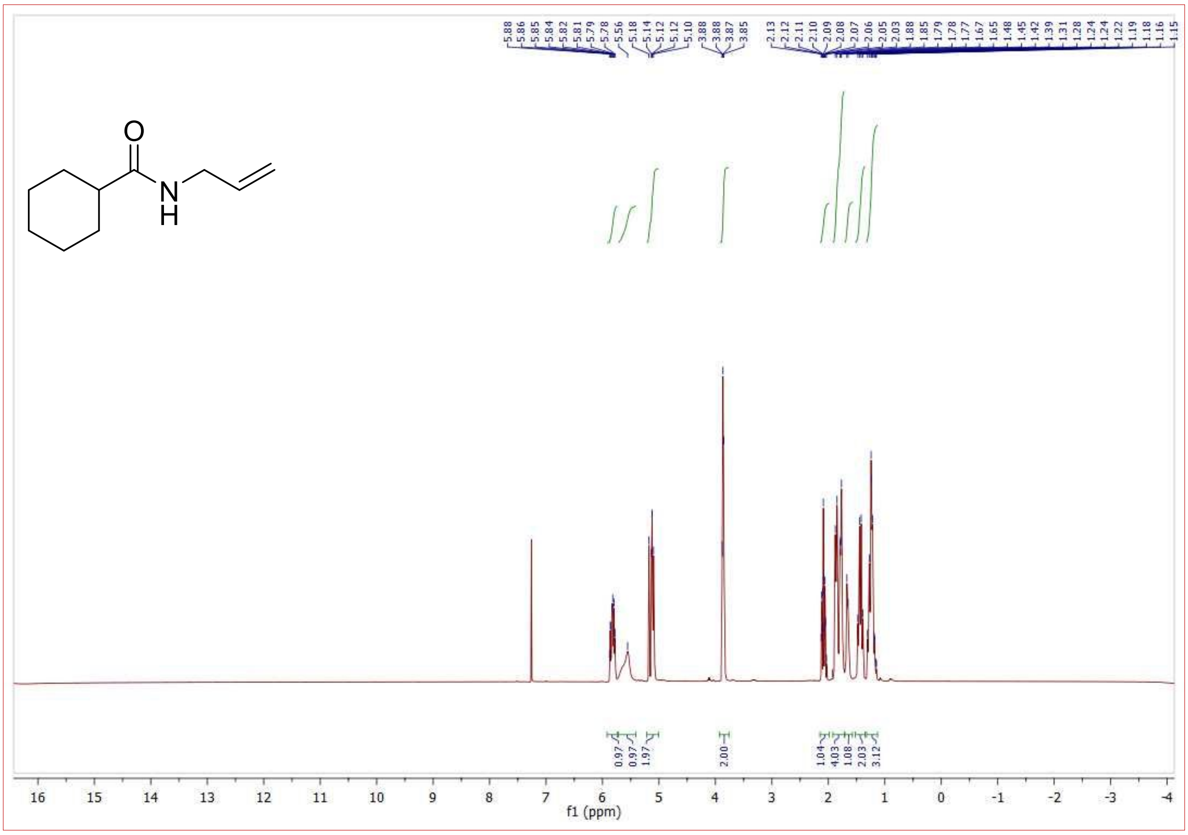

N-Allylcyclohexanecarboxamide (1u), ^{13}C{^{1}H} NMR (101 MHz, CDCl$_3$)

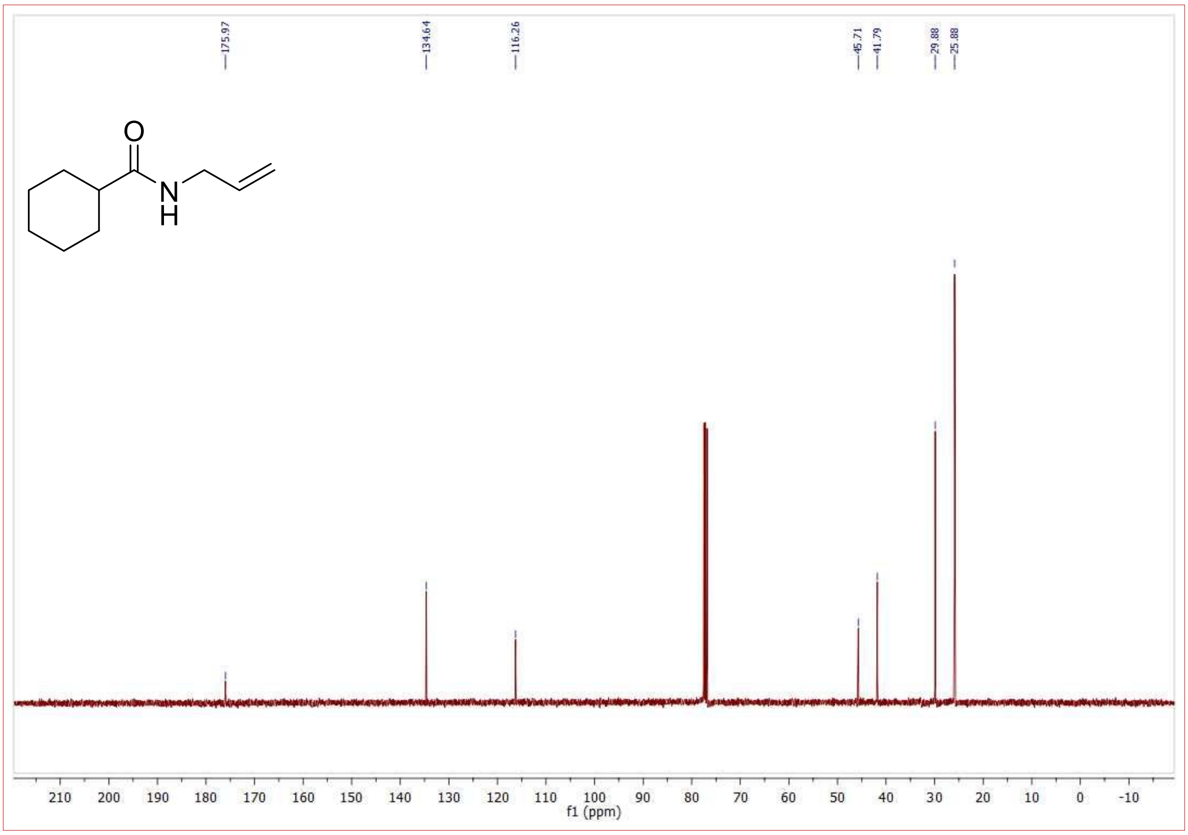

***N*-(2-Methylallyl)cyclohexanecarboxamide (1v), ^{1}H NMR (400 MHz, $CDCl_3$)**

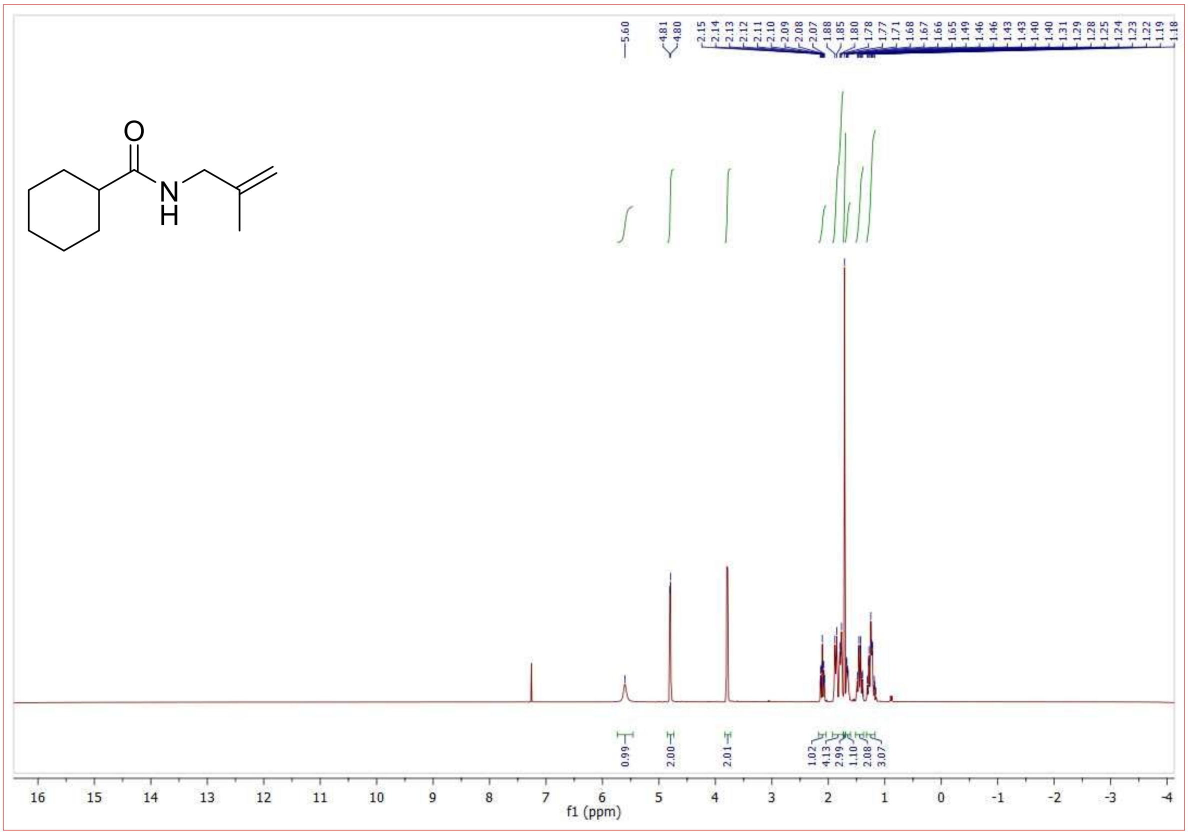

***N*-(2-Methylallyl)cyclohexanecarboxamide (1v), $^{13}C\{^{1}H\}$ NMR (101 MHz, $CDCl_3$)**

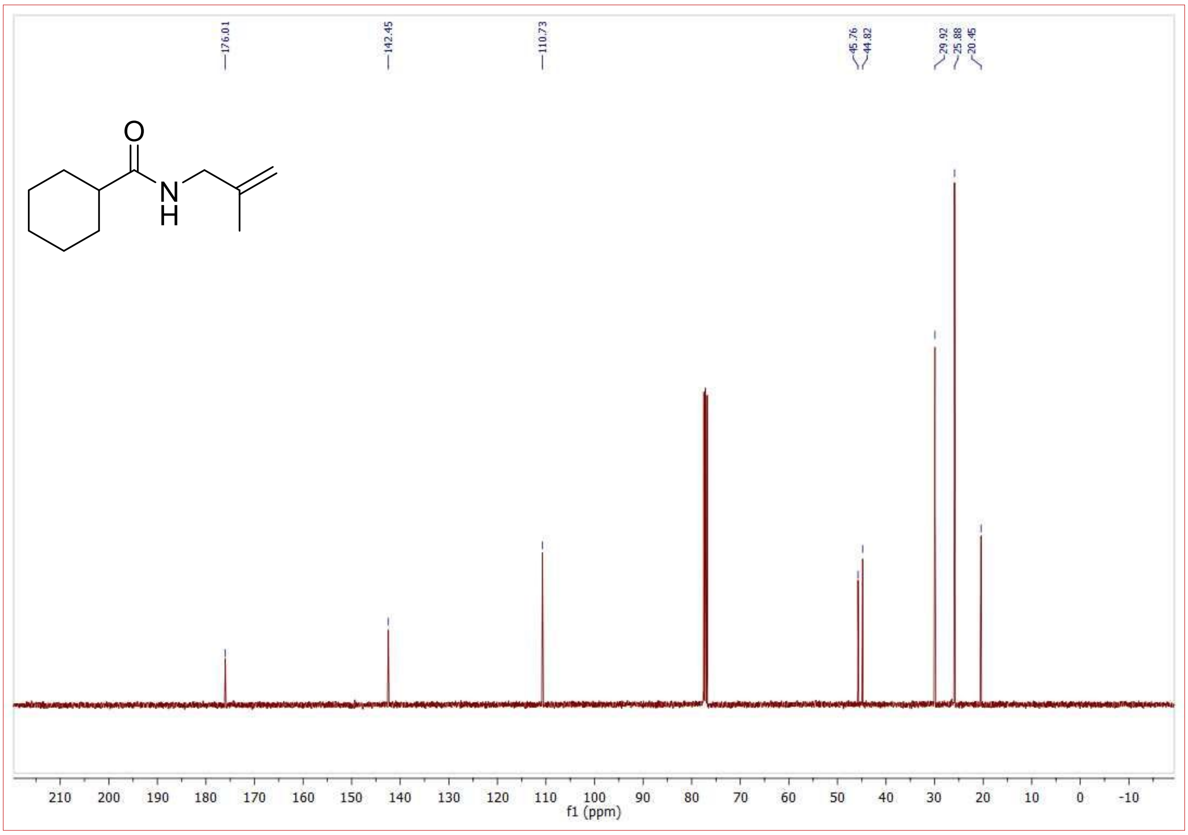

***N*-Allylpivalamide (1w), ^{1}H NMR (400 MHz, CDCl$_3$)**

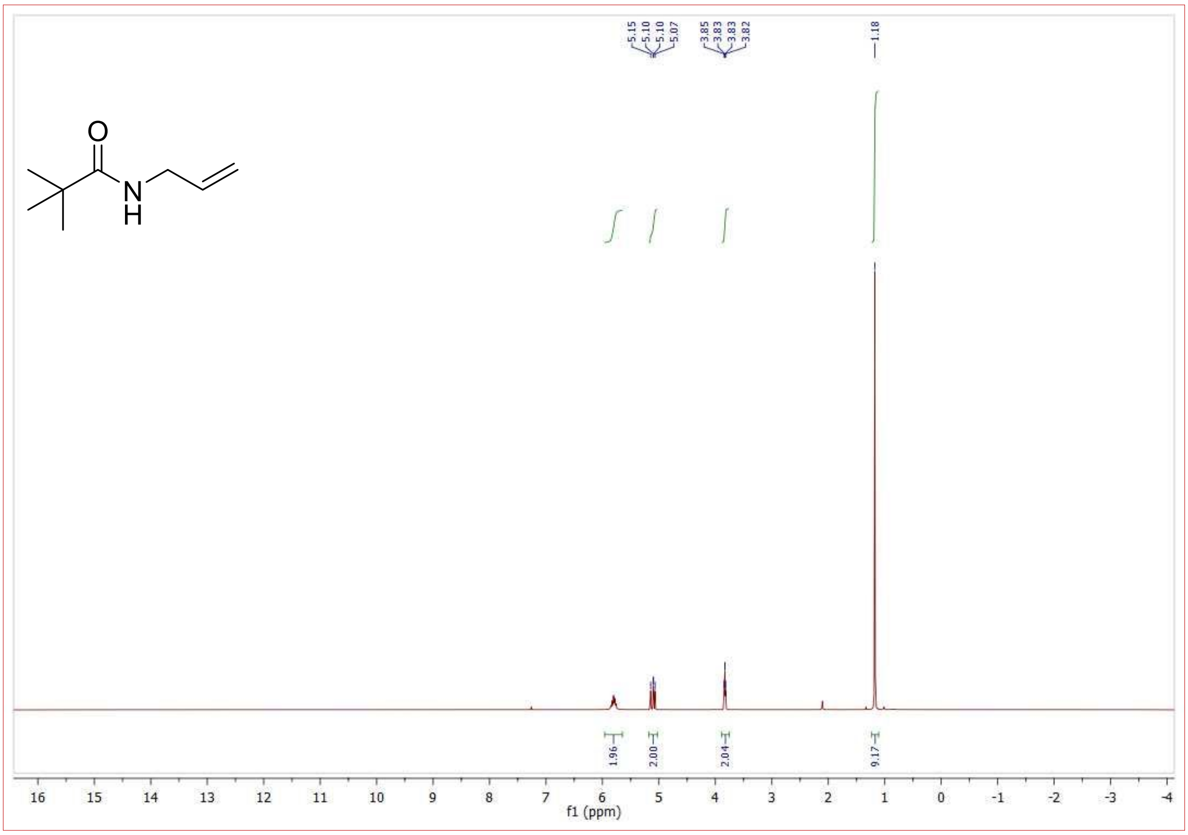

***N*-Allylpivalamide (1w), ^{13}C{^{1}H} NMR (101 MHz, CDCl$_3$)**

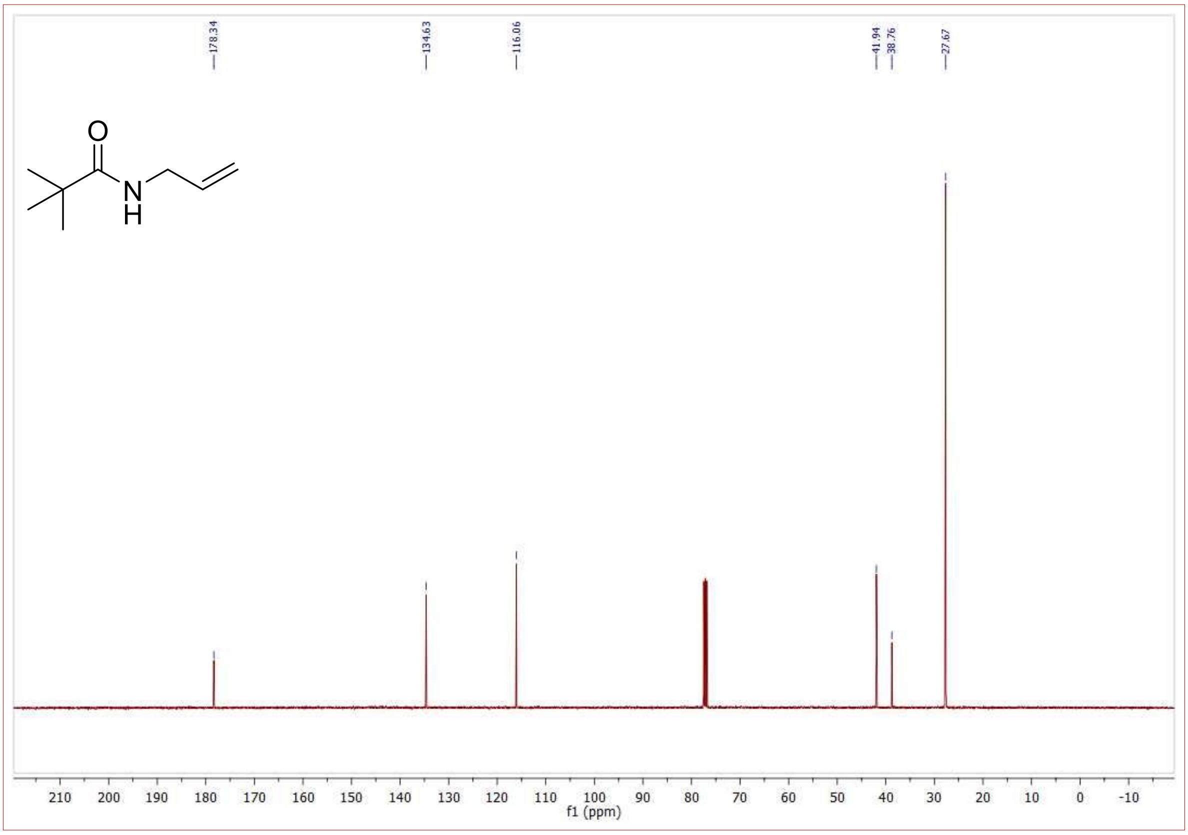

***N*-Allylfuran-2-carboxamide (1x), ^{1}H NMR (400 MHz, $CDCl_3$)**

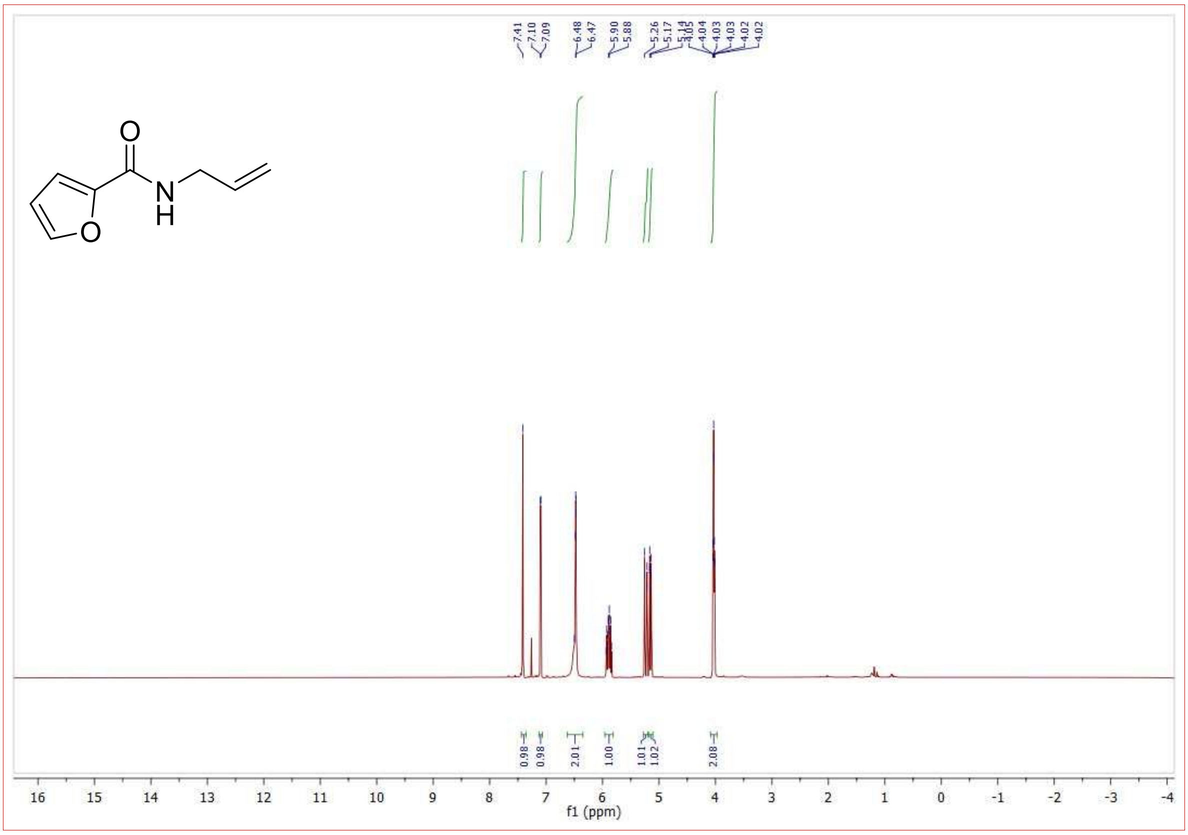

***N*-Allylfuran-2-carboxamide (1x), $^{13}C\{^{1}H\}$ NMR (101 MHz, $CDCl_3$)**

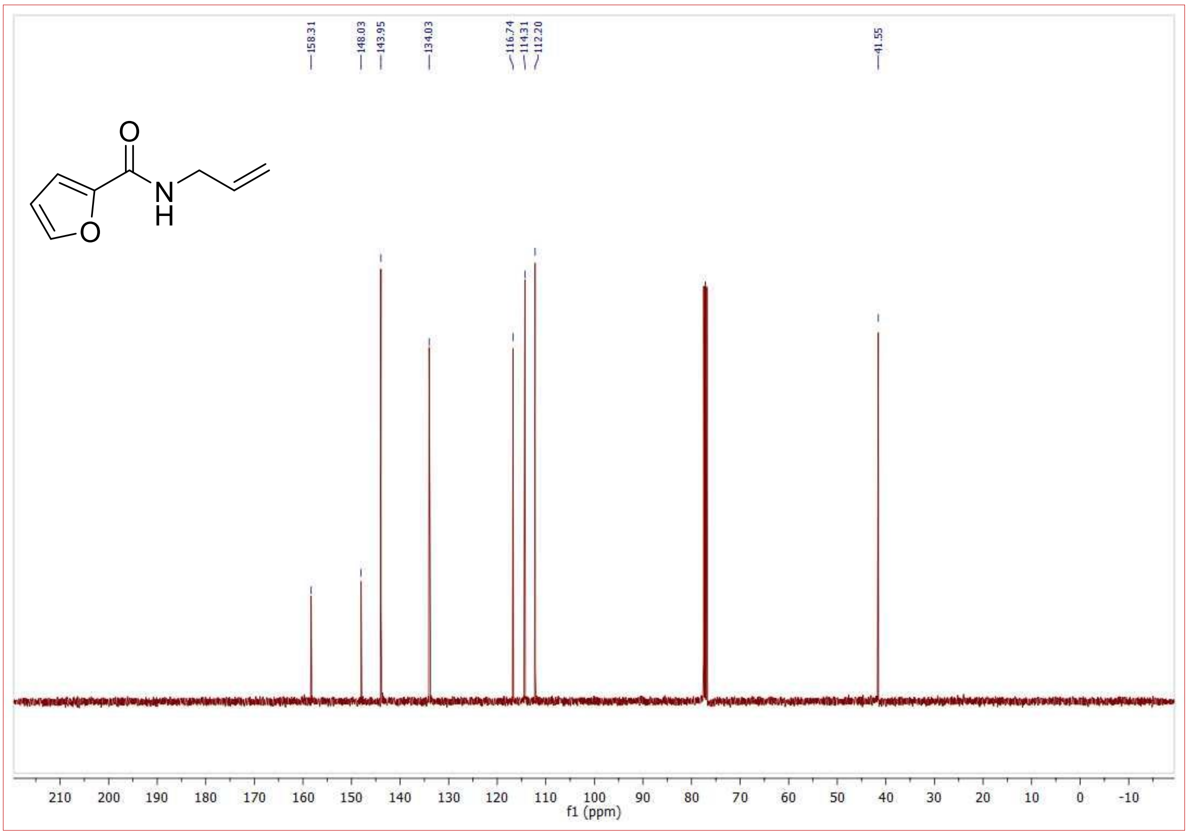

N-(2-Methylallyl)furan-2-carboxamide (1y), ^{1}H NMR (400 MHz, $CDCl_3$)

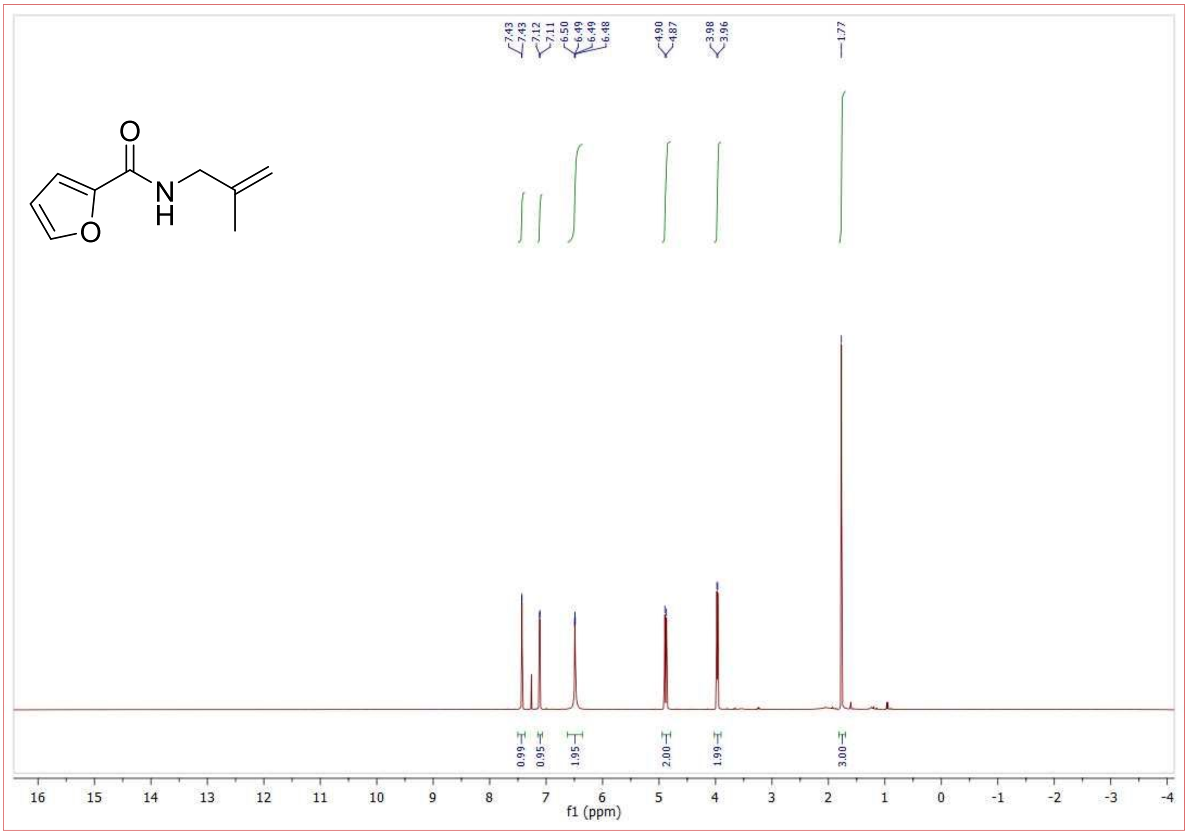

N-(2-Methylallyl)furan-2-carboxamide (1y), $^{13}C\{^1H\}$ NMR (101 MHz, $CDCl_3$)

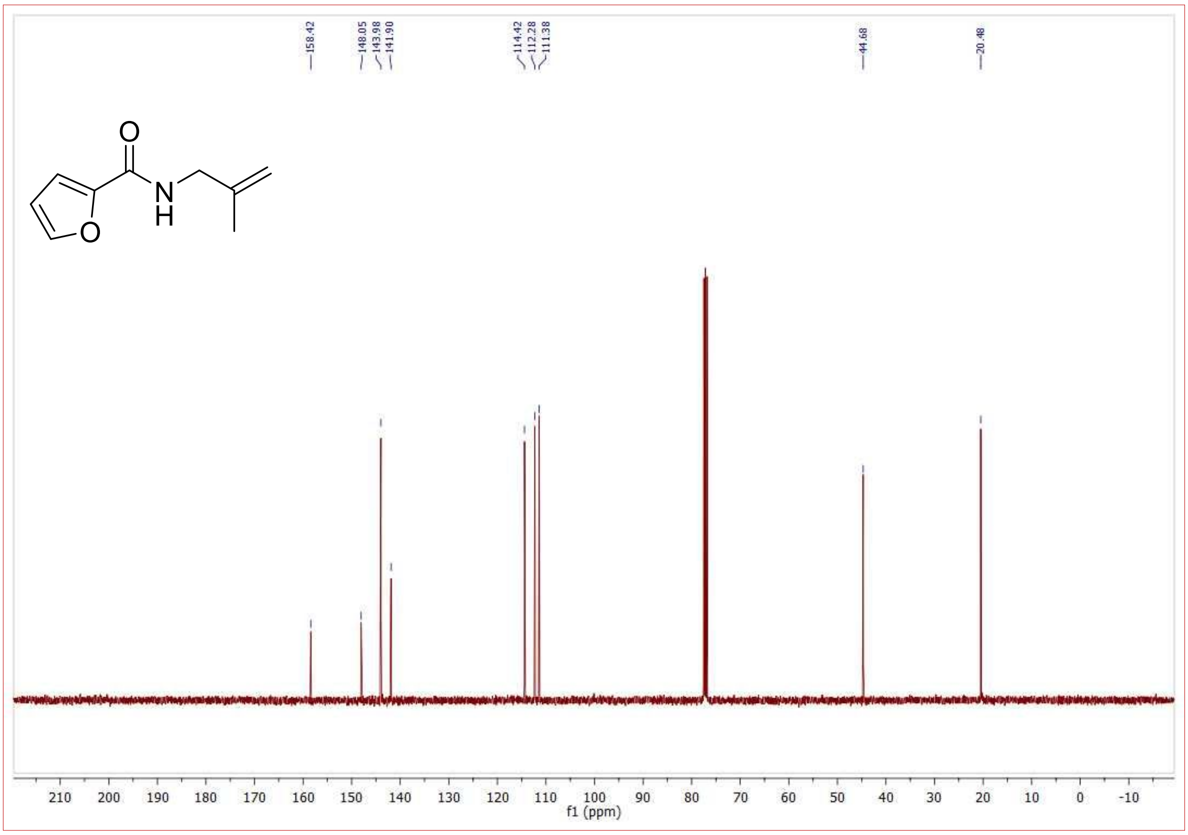

***N*-(But-3-en-1-yl)-4-chlorobenzamide (1z), ^{1}H NMR (400 MHz, $CDCl_3$)**

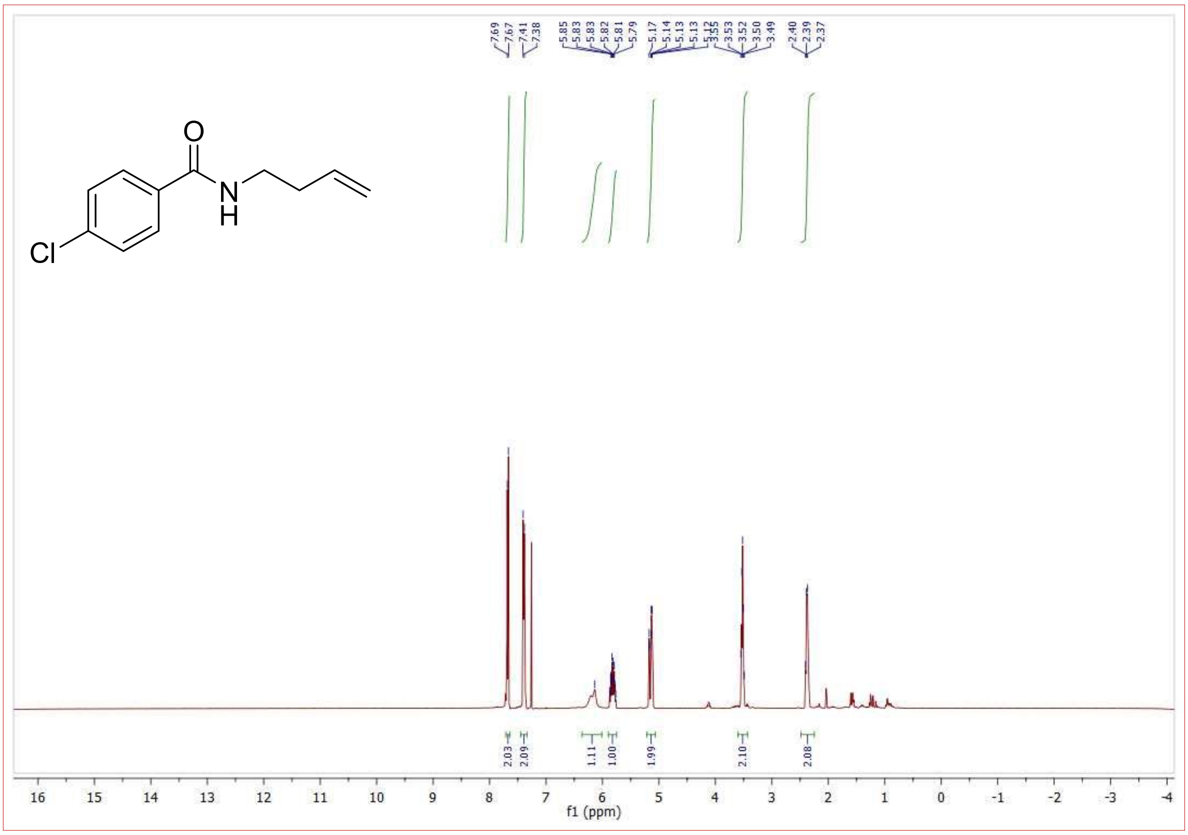

***N*-(But-3-en-1-yl)-4-chlorobenzamide (1z), ^{13}C{^{1}H} NMR (101 MHz, $CDCl_3$)**

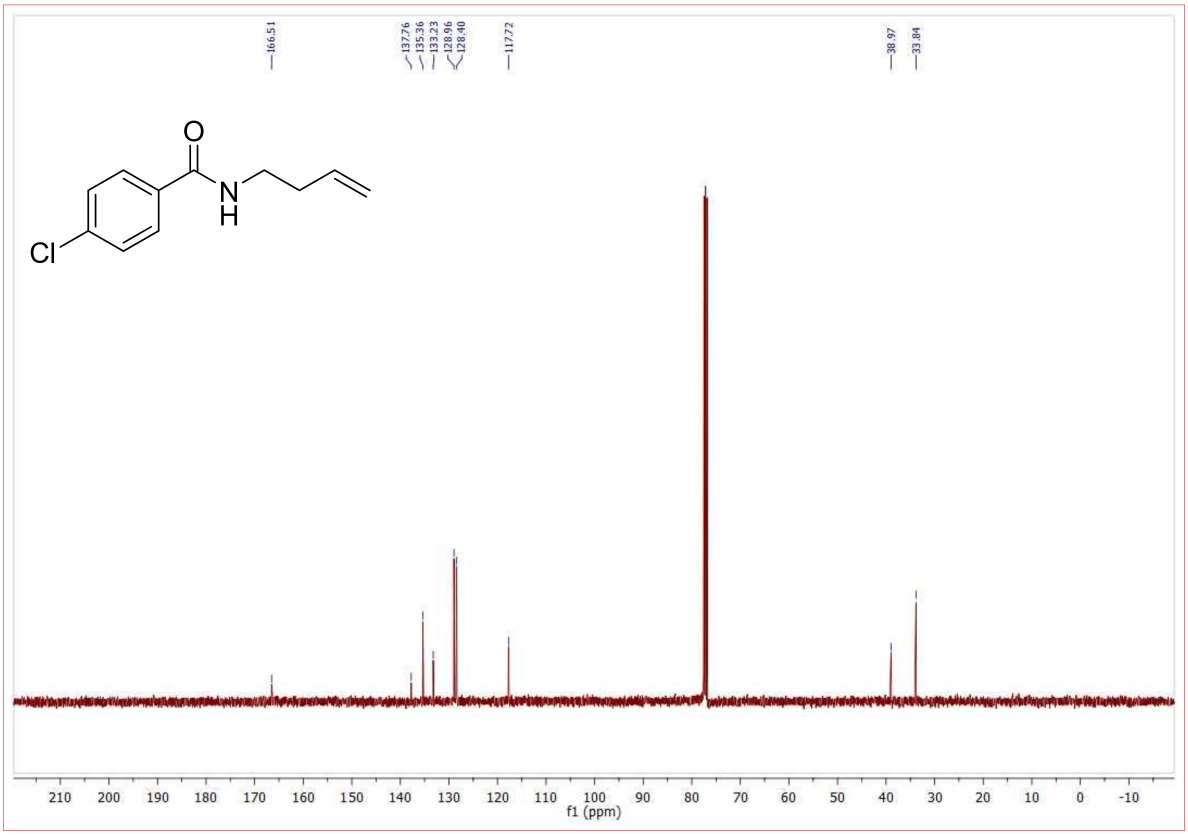

N-(But-3-en-1-yl)-3-(trifluoromethyl)benzamide (1aa), ^{1}H NMR (400 MHz, $CDCl_3$)

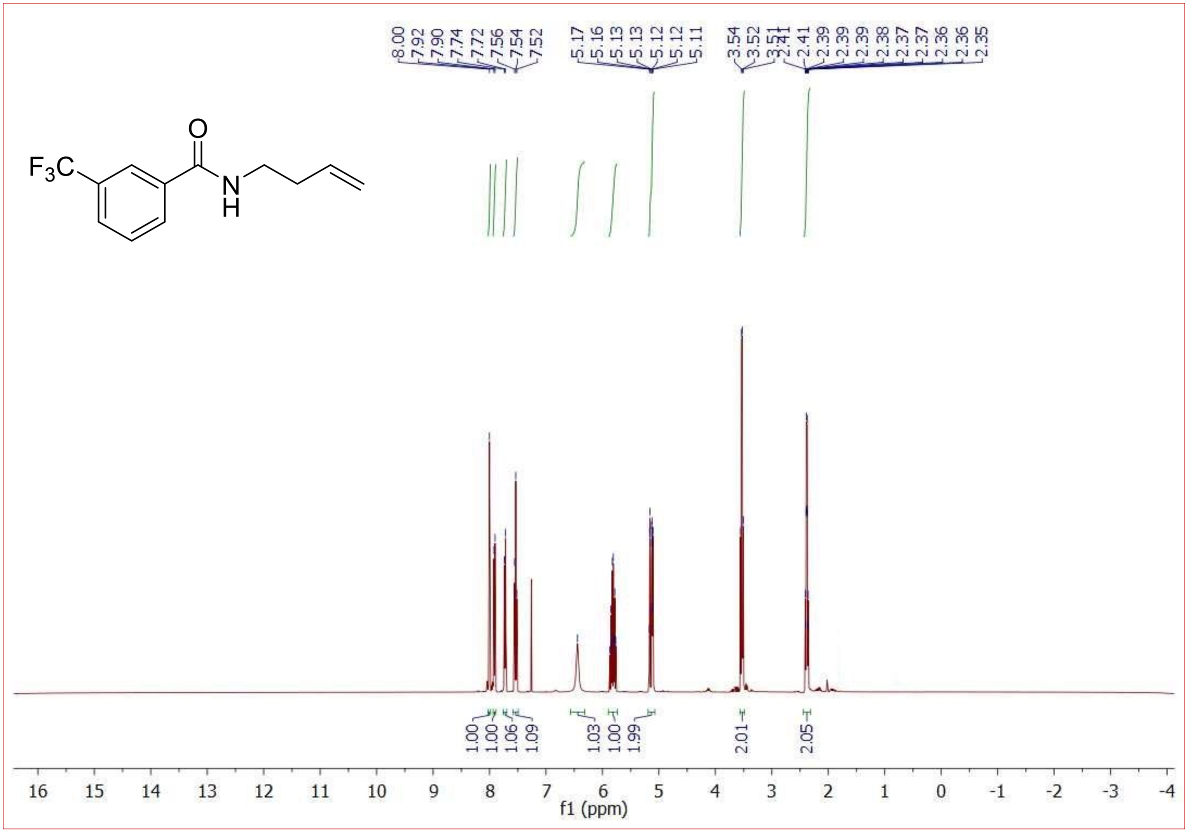

N-(But-3-en-1-yl)-3-(trifluoromethyl)benzamide (1aa), $^{13}C\{^1H\}$ NMR (101 MHz, $CDCl_3$)

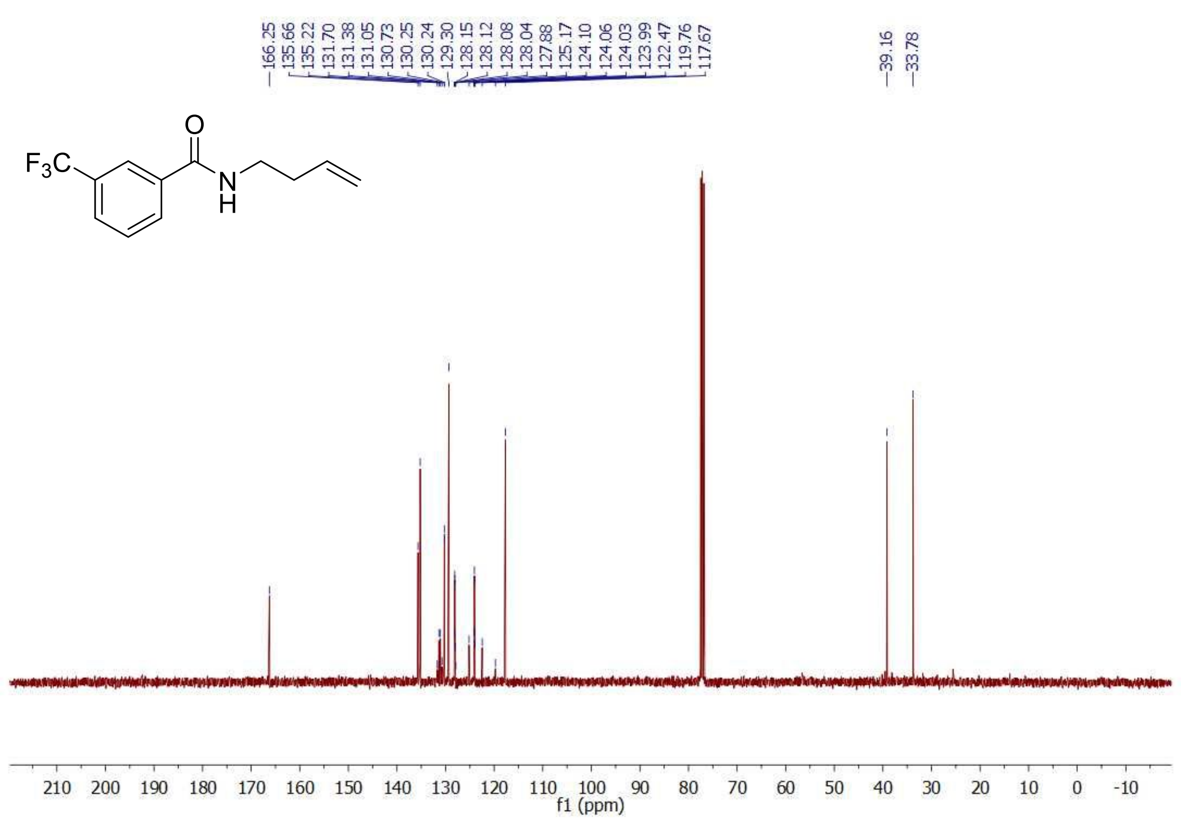

(2-Phenyl-4,5-dihydrooxazol-5-yl)methyl 4-methylbenzenesulfonate (2a), ^{1}H NMR (400 MHz, $CDCl_3$)

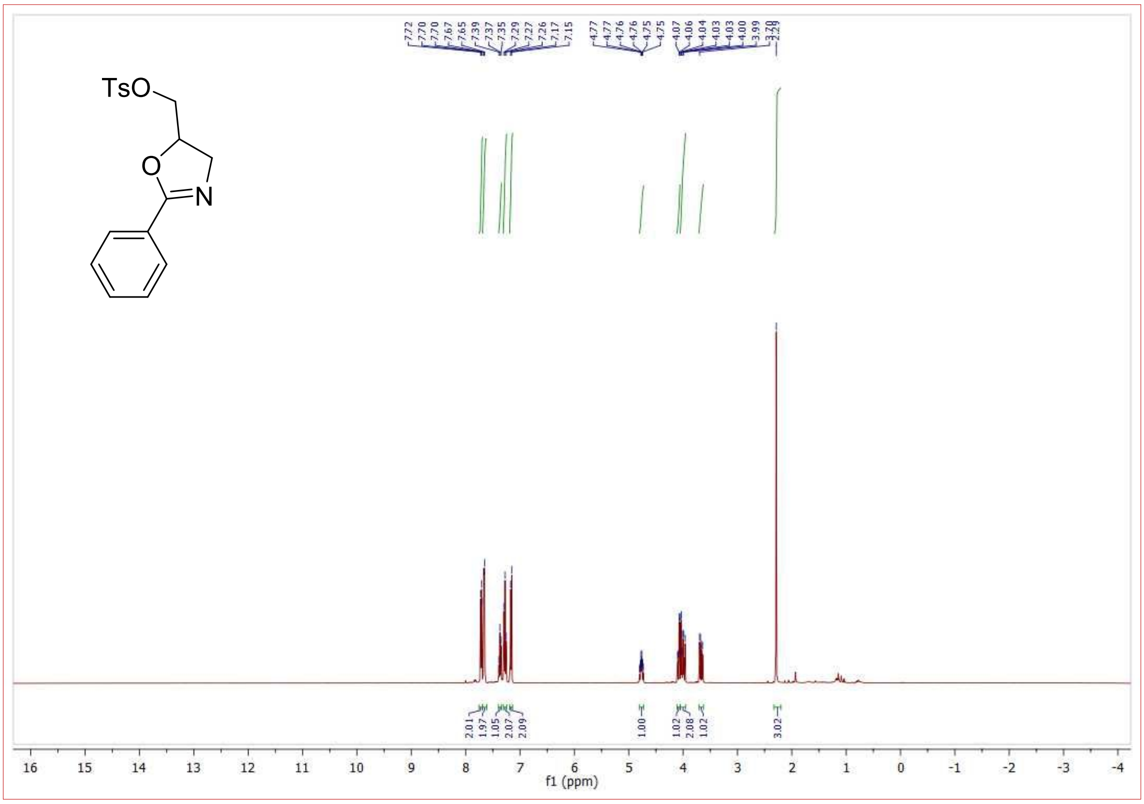

(2-Phenyl-4,5-dihydrooxazol-5-yl)methyl 4-methylbenzenesulfonate (2a), $^{13}C\{^{1}H\}$ NMR (101 MHz, $CDCl_3$)

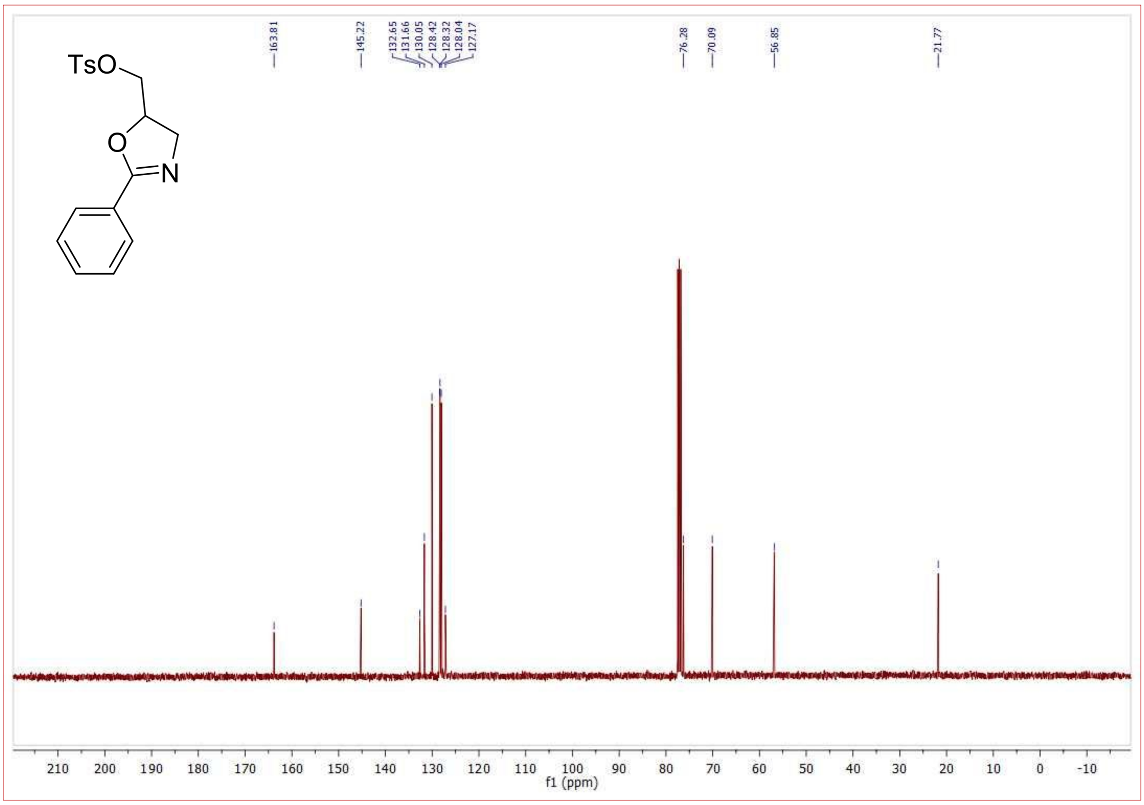

(2-(4-Chlorophenyl)-4,5-dihydrooxazol-5-yl)methyl 4-methylbenzenesulfonate (2b), ^{1}H NMR (400 MHz, $CDCl_3$)

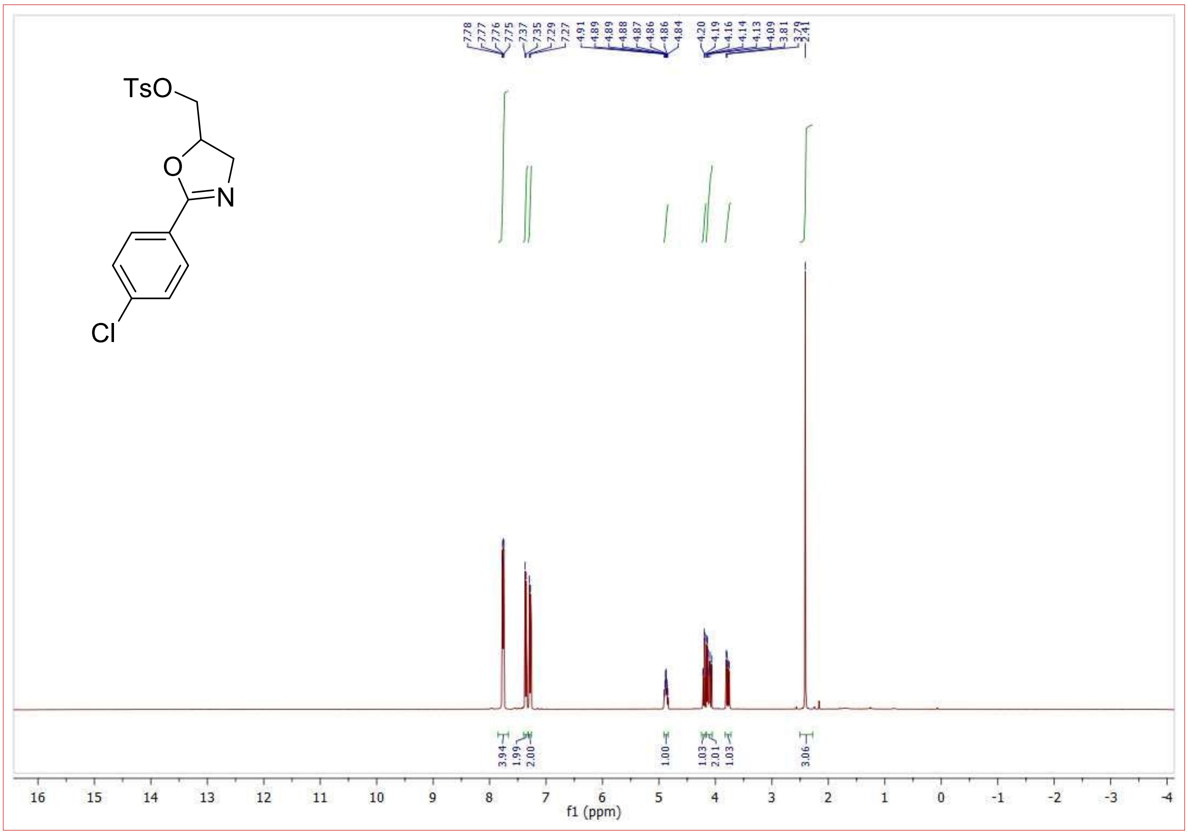

(2-(4-Chlorophenyl)-4,5-dihydrooxazol-5-yl)methyl 4-methylbenzenesulfonate (2b), $^{13}C\{^{1}H\}$ NMR (101 MHz, $CDCl_3$)

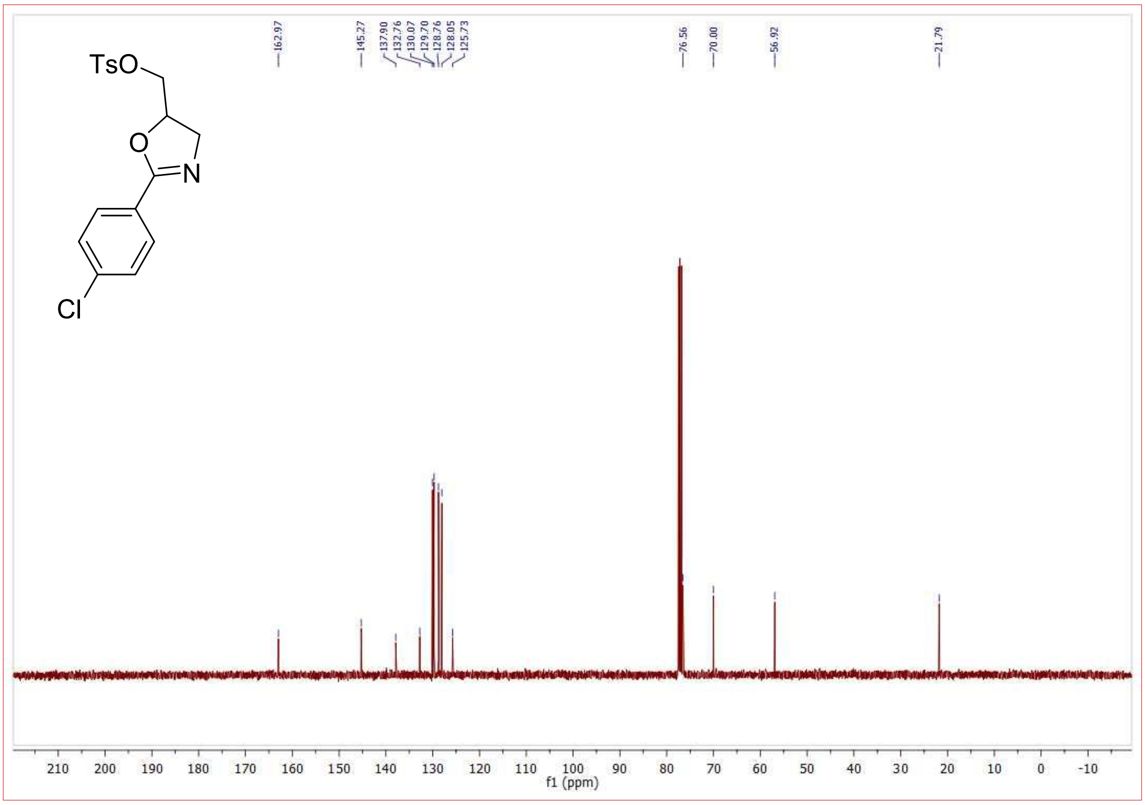

(2-(4-Iodophenyl)-4,5-dihydrooxazol-5-yl)methyl 4-methylbenzenesulfonate (2c), ^{1}H NMR (400 MHz, $CDCl_3$)

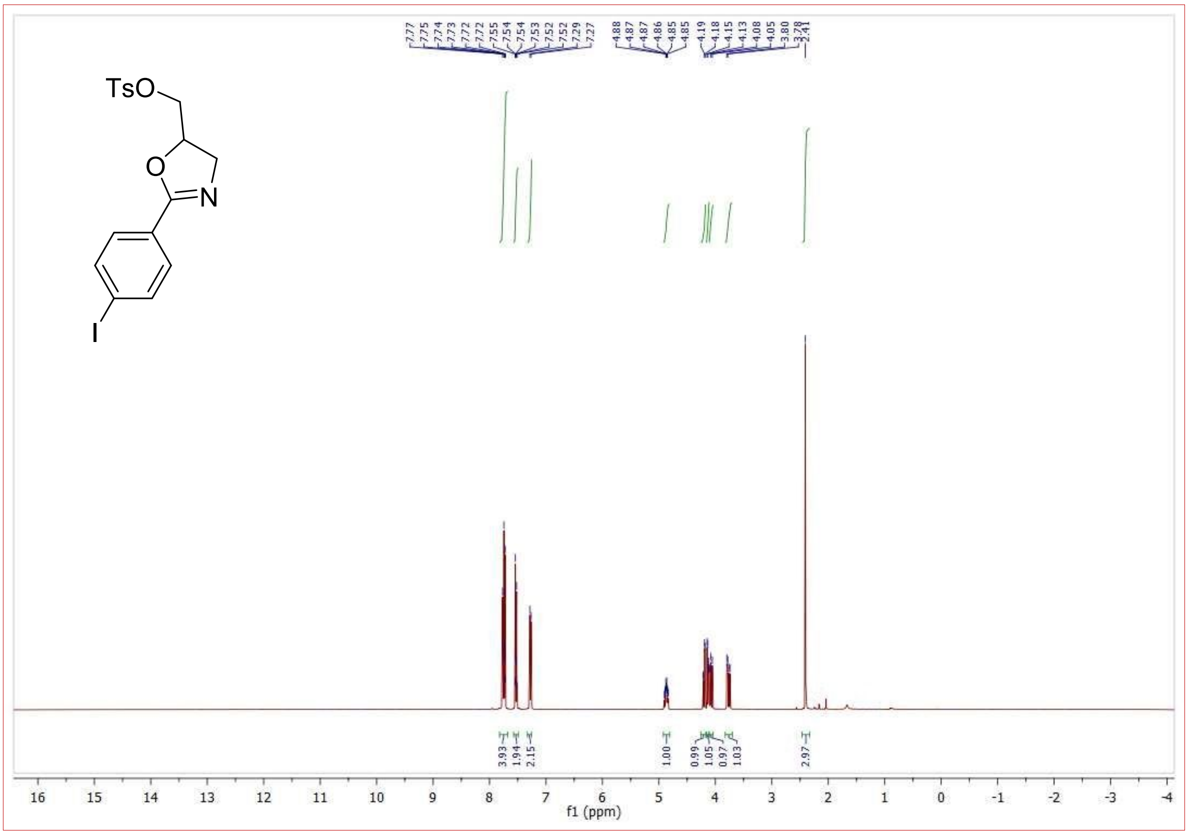

(2-(4-Iodophenyl)-4,5-dihydrooxazol-5-yl)methyl 4-methylbenzenesulfonate (2c), $^{13}C\{^{1}H\}$ NMR (101 MHz, $CDCl_3$)

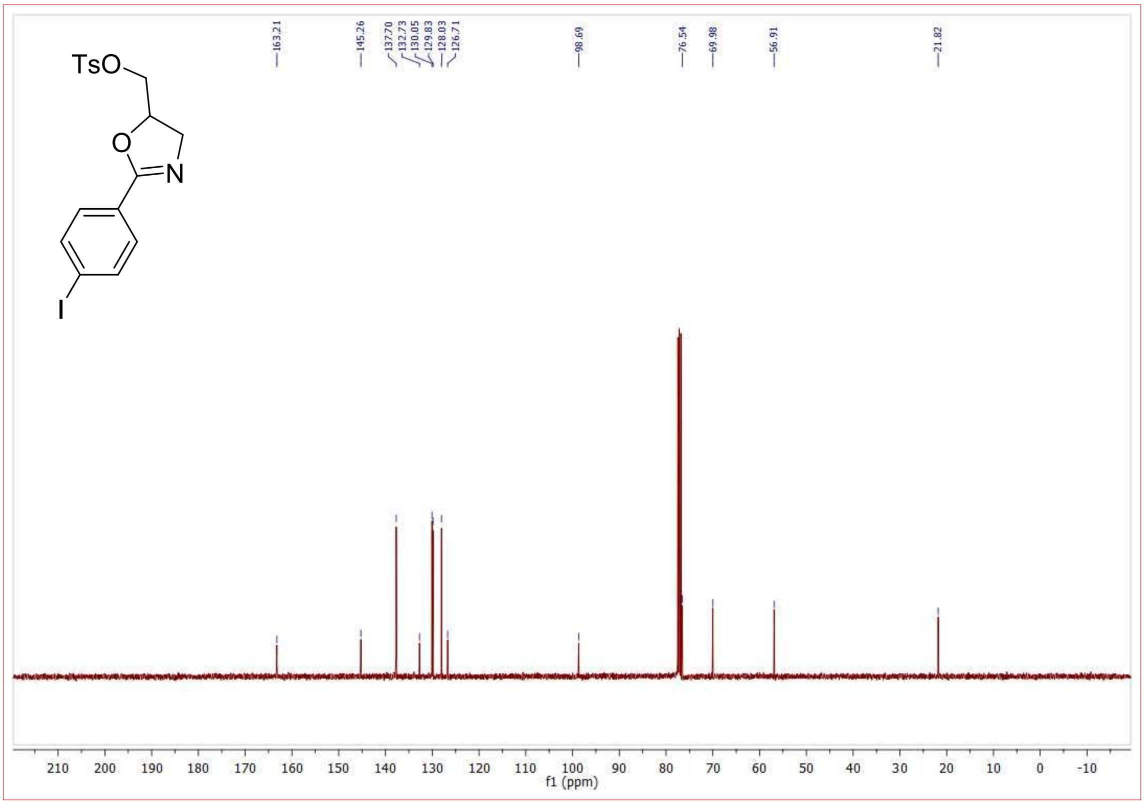

(2-(4-Isopropylphenyl)-4,5-dihydrooxazol-5-yl)methyl 4-methylbenzenesulfonate (2d), ^{1}H NMR (400 MHz, $CDCl_3$)

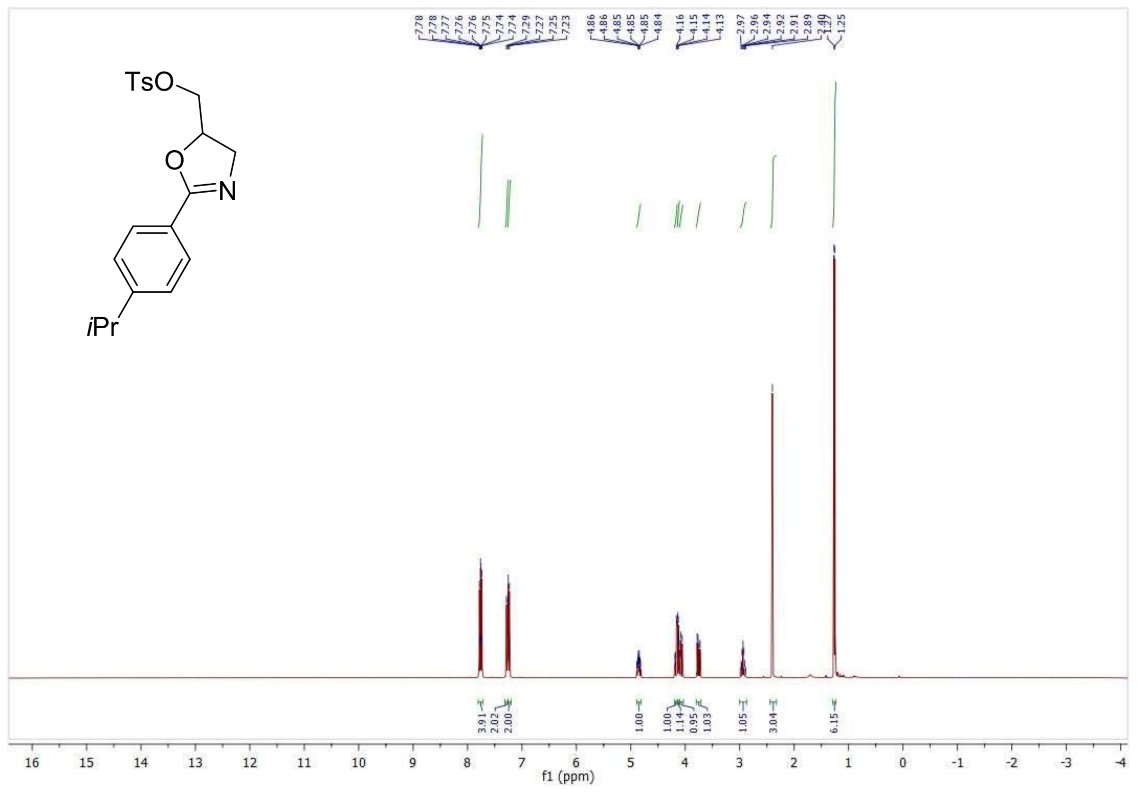

(2-(4-Isopropylphenyl)-4,5-dihydrooxazol-5-yl)methyl 4-methylbenzenesulfonate (2c), $^{13}C\{^{1}H\}$ NMR (101 MHz, $CDCl_3$)

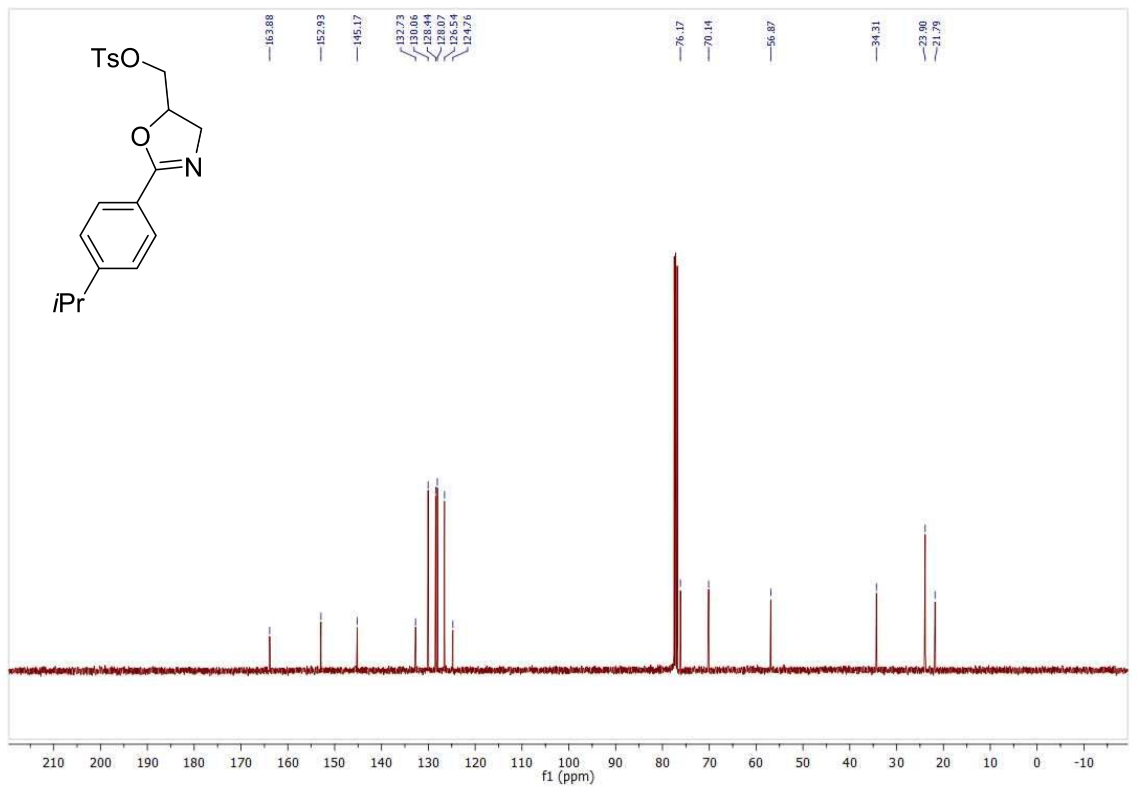

(2-(4-Methoxypylphenyl)-4,5-dihydrooxazol-5-yl)methyl 4-methylbenzenesulfonate (2e), ^{1}H NMR (400 MHz, $CDCl_3$)

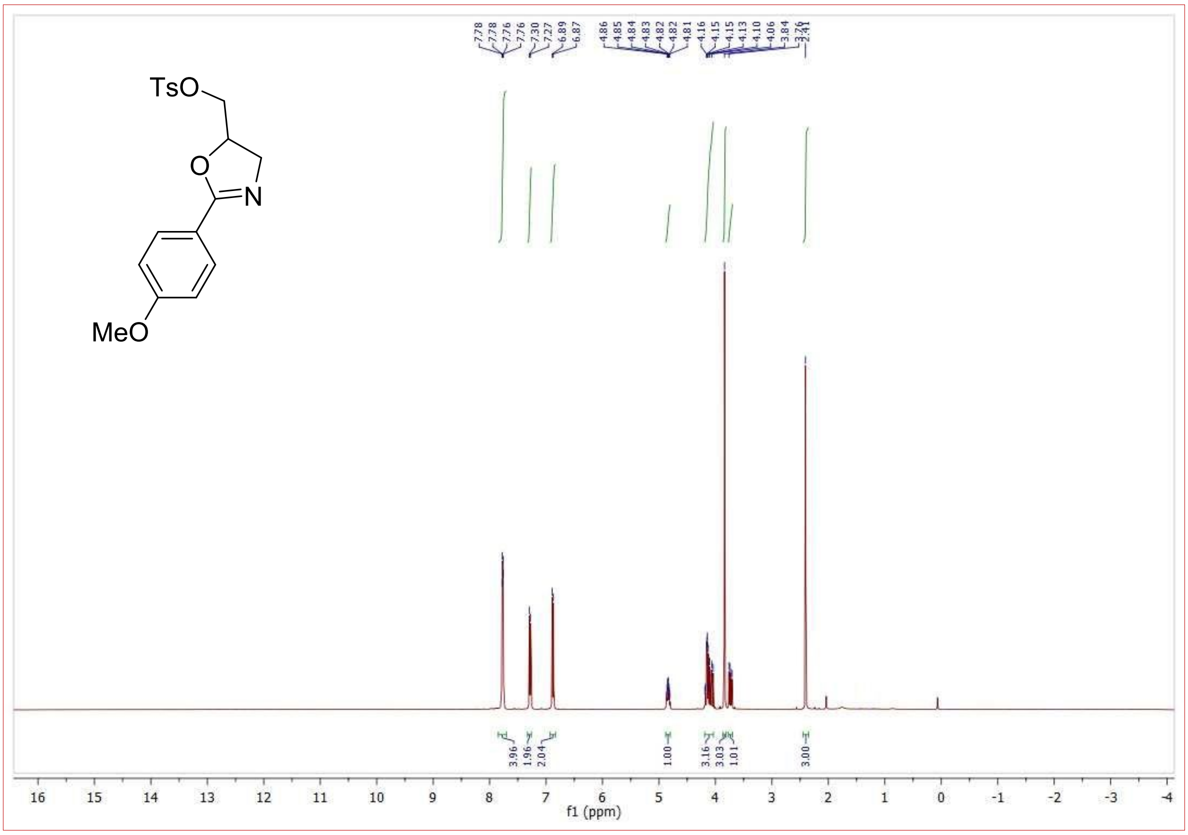

(2-(4-Methoxyphenyl)-4,5-dihydrooxazol-5-yl)methyl 4-methylbenzenesulfonate (2e), $^{13}C\{^1H\}$ NMR (101 MHz, $CDCl_3$)

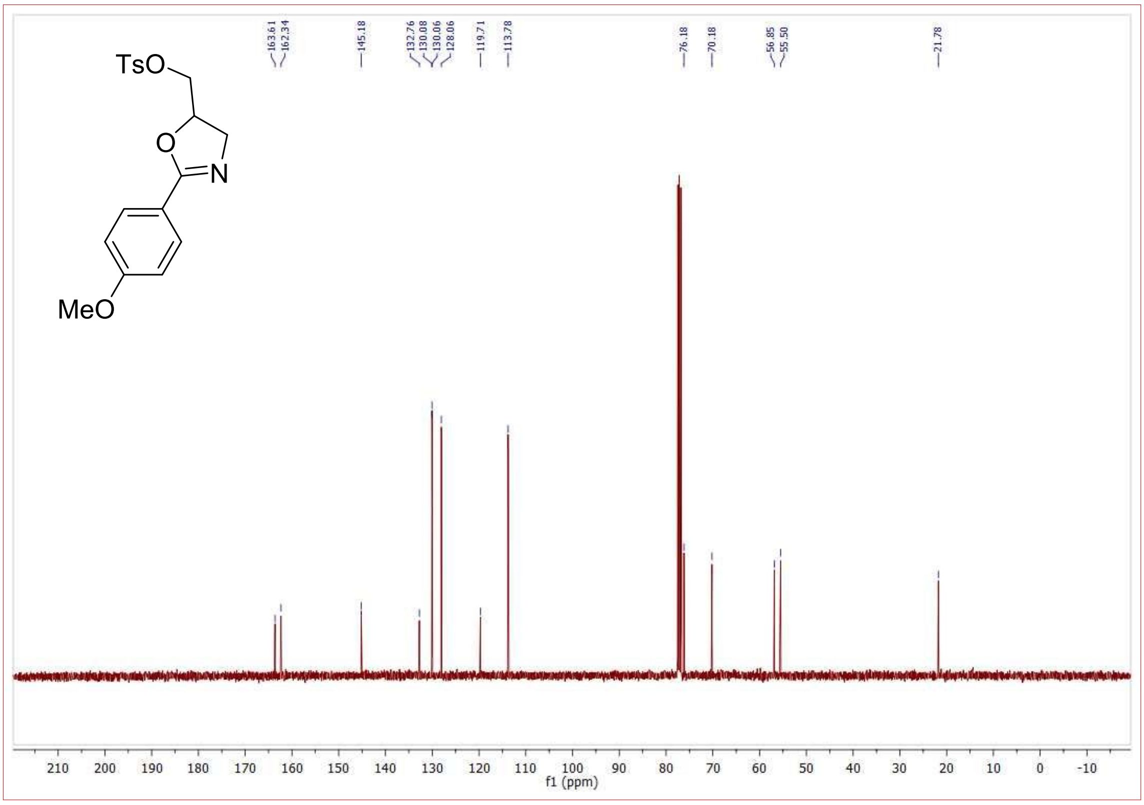

Methyl 4-(5-((tosyloxy)methyl)-4,5-dihydrooxazol-2-yl)benzoate (2f), ^{1}H NMR (400 MHz, $CDCl_3$)

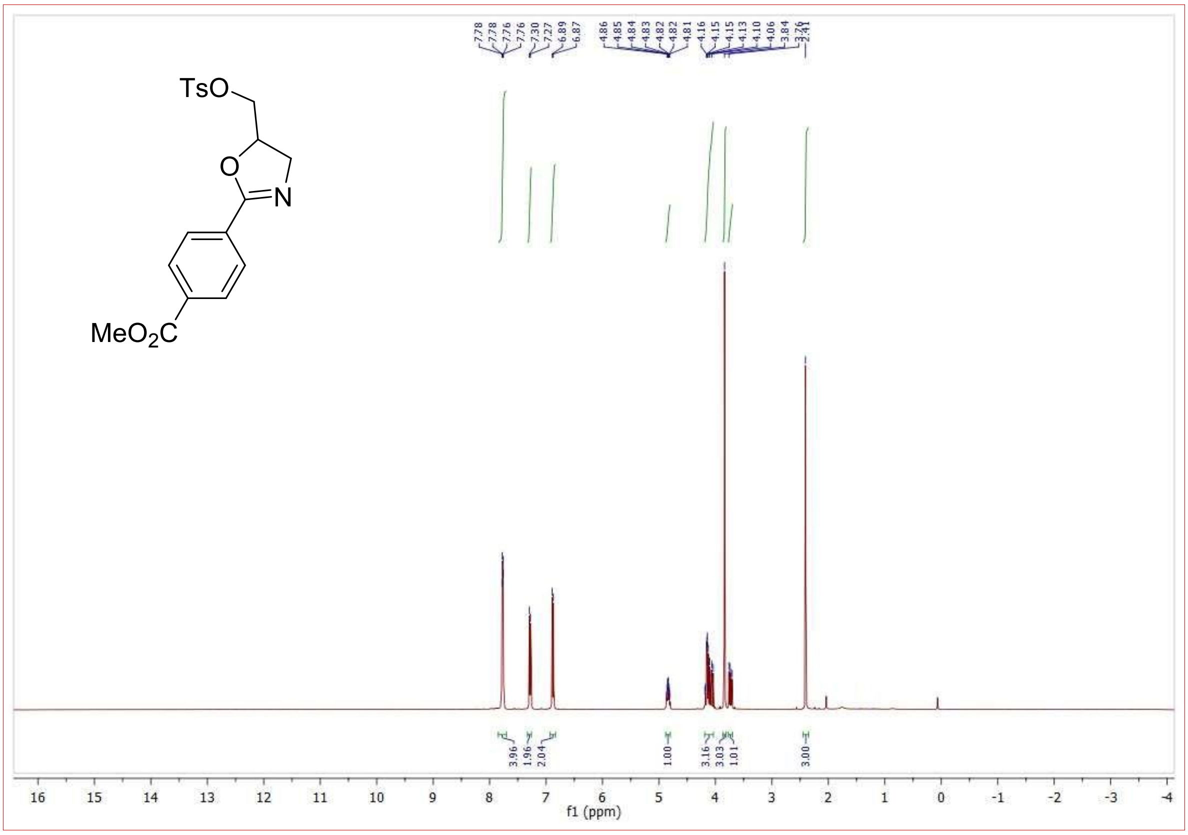

Methyl 4-(5-((tosyloxy)methyl)-4,5-dihydrooxazol-2-yl)benzoate (2f), $^{13}C\{^{1}H\}$ NMR (101 MHz, $CDCl_3$)

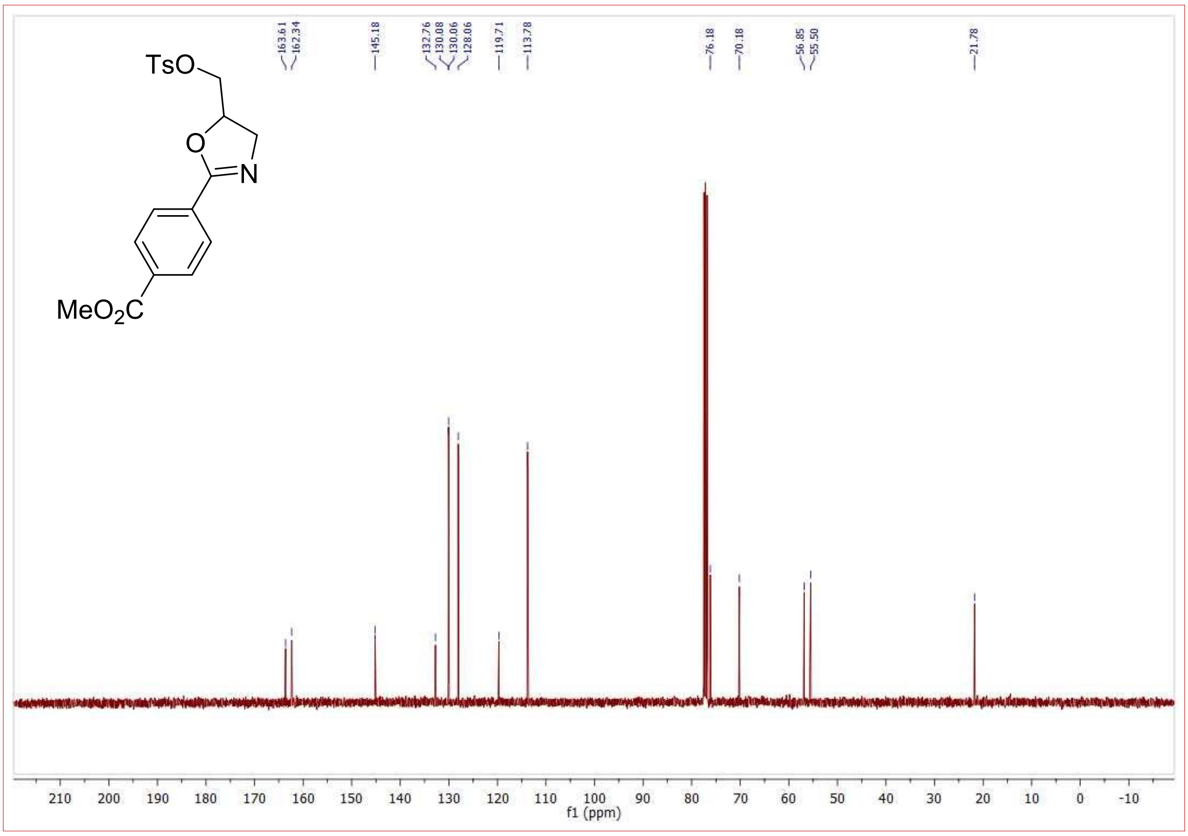

(2-(3-Cyanophenyl)-4,5-dihydrooxazol-5-yl)methyl 4-methylbenzenesulfonate (2g), ^{1}H NMR (400 MHz, $CDCl_3$)

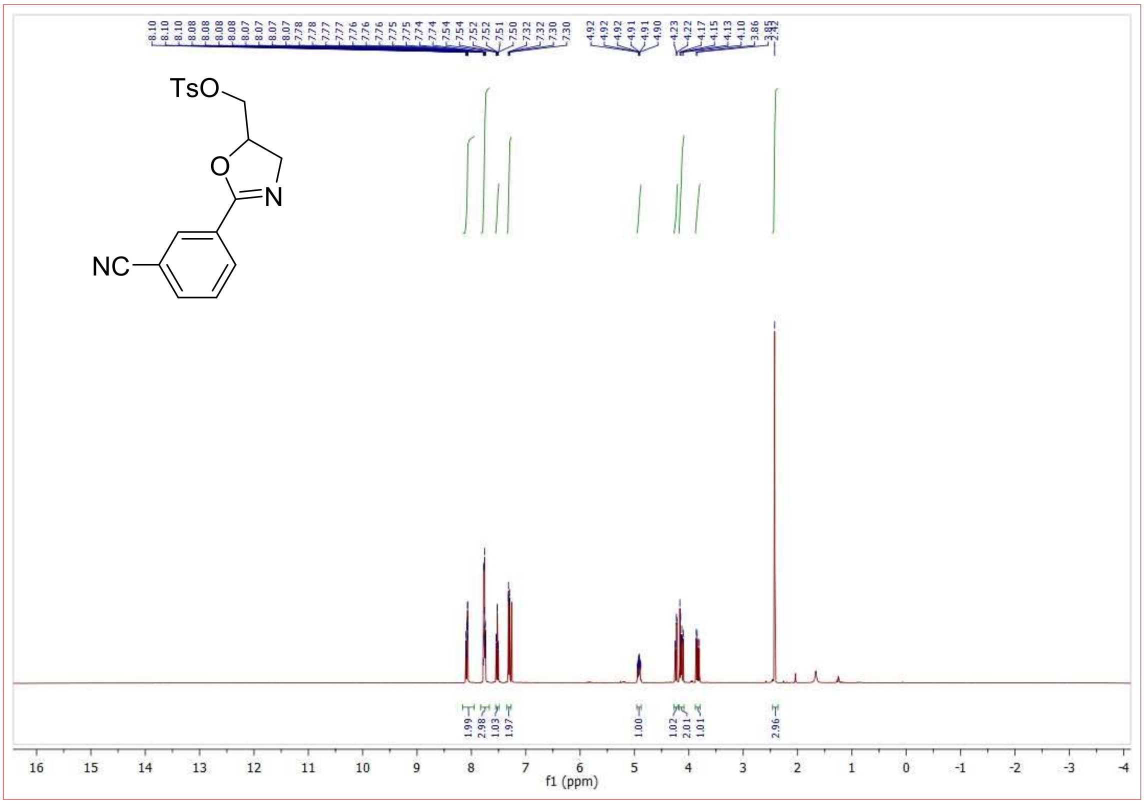

(2-(3-Cyanophenyl)-4,5-dihydrooxazol-5-yl)methyl 4-methylbenzenesulfonate (2g), $^{13}C\{^{1}H\}$ NMR (101 MHz, $CDCl_3$)

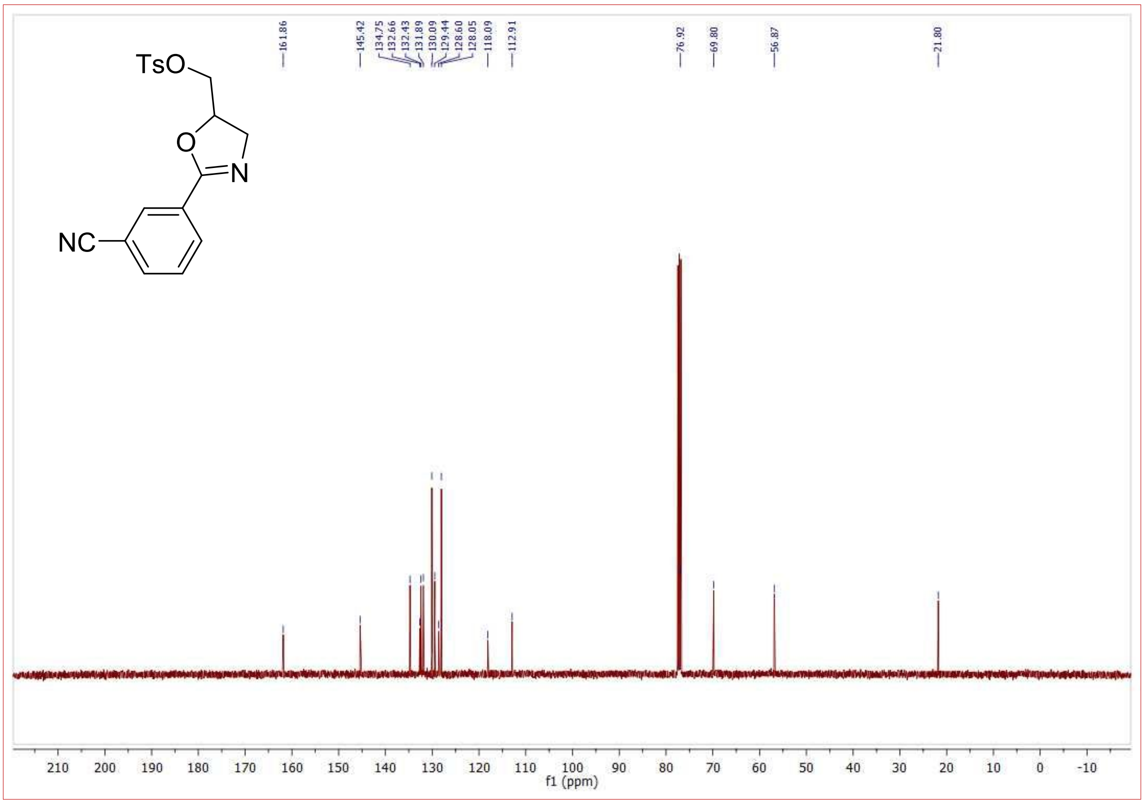

(2-(3-(Trifluoromethyl)phenyl)-4,5-dihydrooxazol-5-yl)methyl 4-methylbenzenesulfonate (2h), ^{1}H NMR (400 MHz, $CDCl_3$)

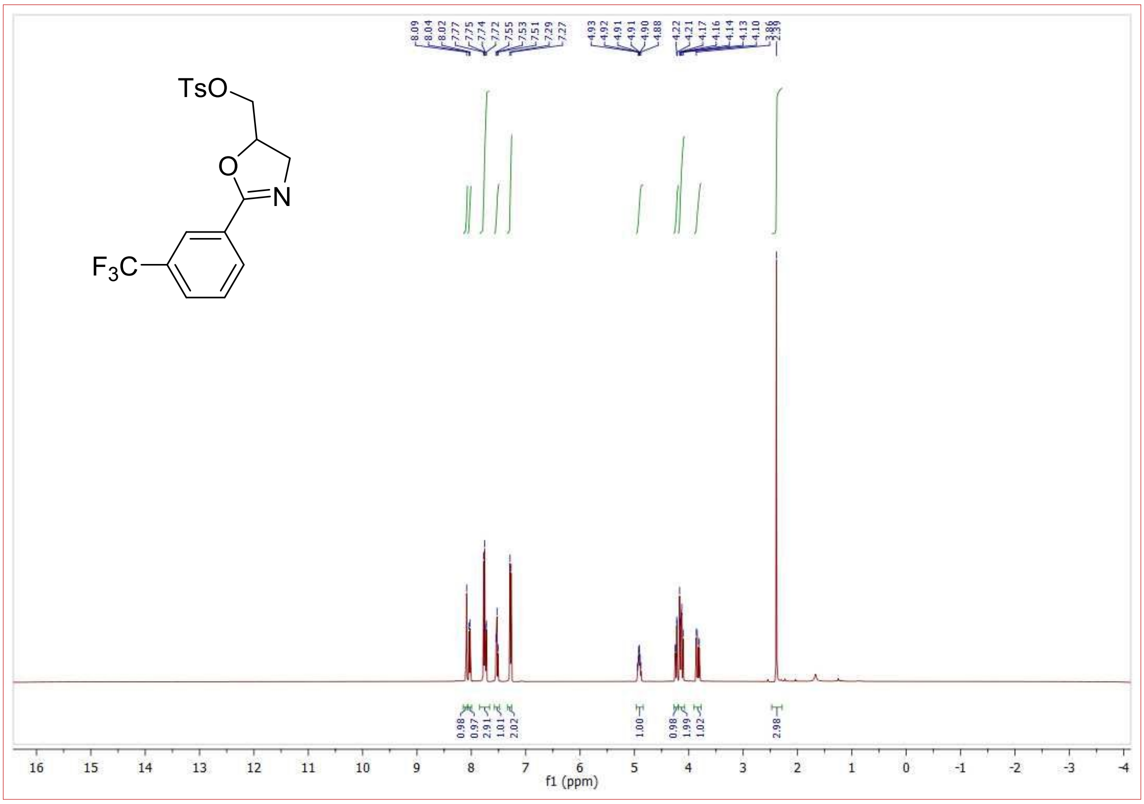

(2-(3-(Trifluoromethyl)phenyl)-4,5-dihydrooxazol-5-yl)methyl 4-methylbenzenesulfonate (2h), ^{13}C{^{1}H} NMR (101 MHz, $CDCl_3$)

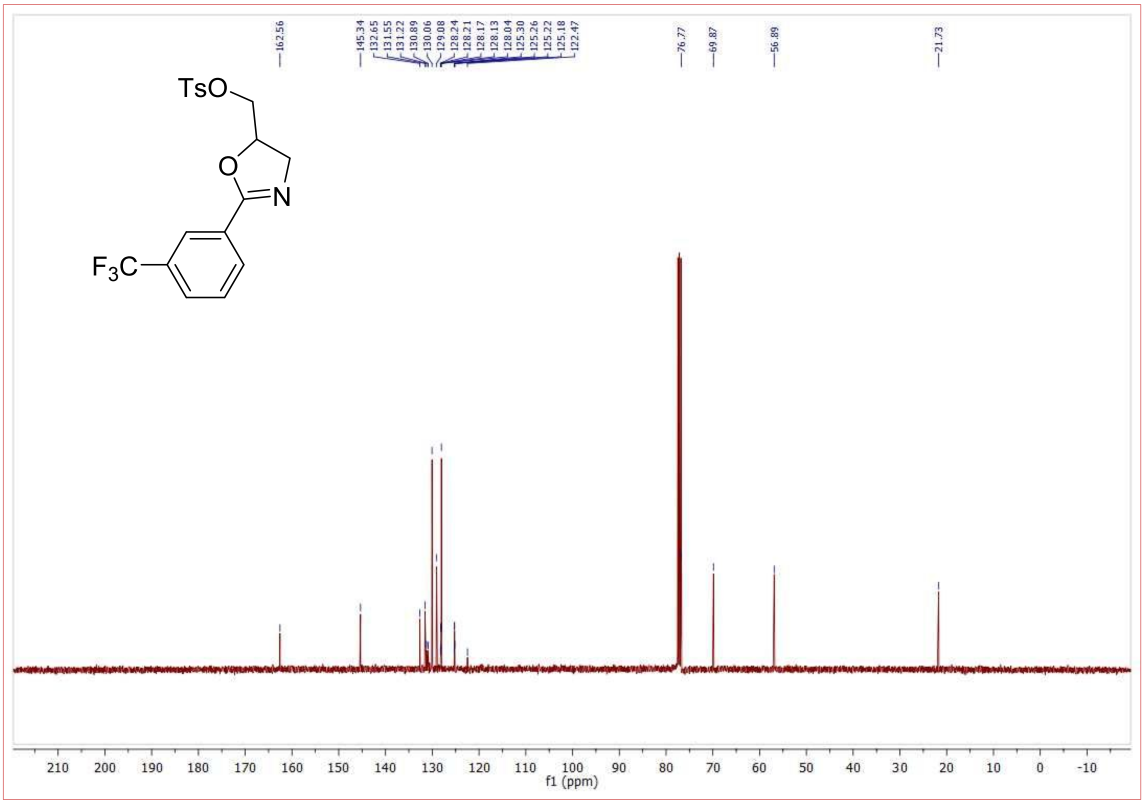

2-(3-(Trifluoromethyl)phenyl)-4,5-dihydrooxazol-5-yl)methyl 4-methylbenzenesulfonate (2h), ^{19}F NMR

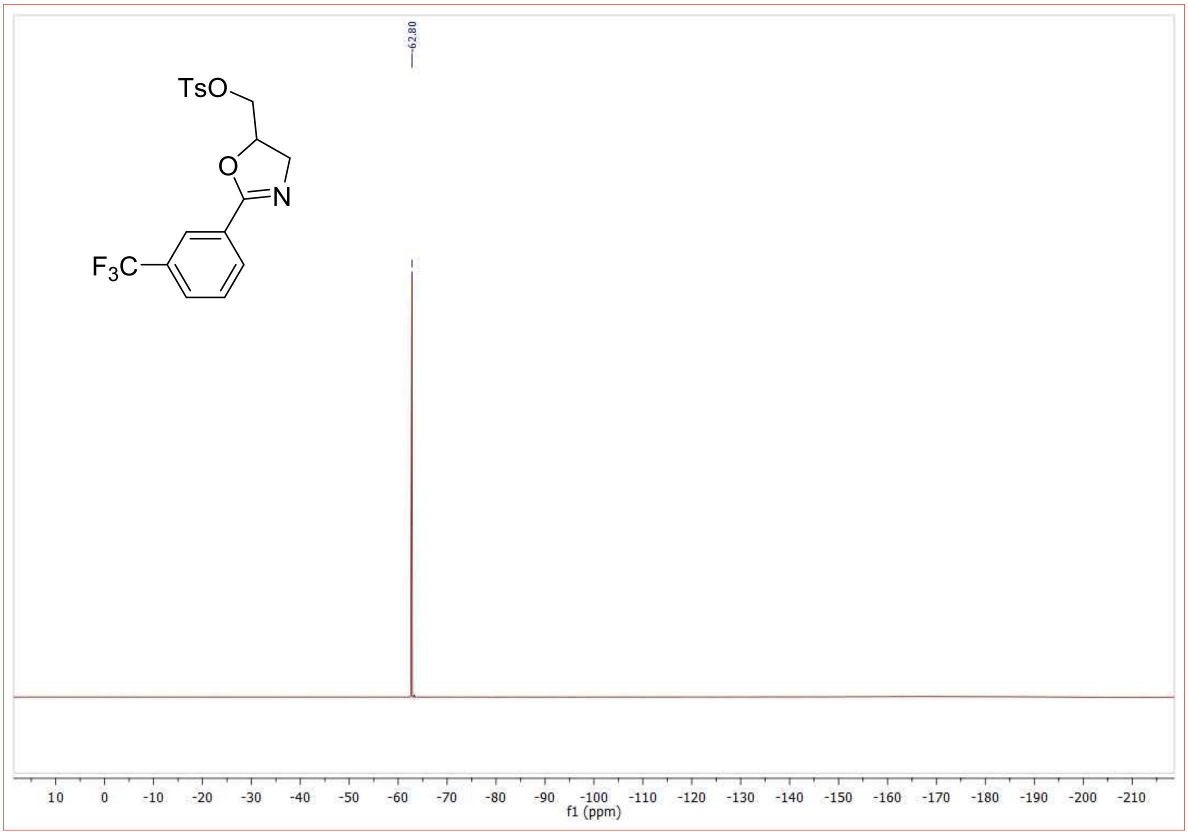

(2-(3-Nitrophenyl)-4,5-dihydrooxazol-5-yl)methyl 4-methylbenzenesulfonate (2i), ^{1}H NMR (400 MHz, $CDCl_3$)

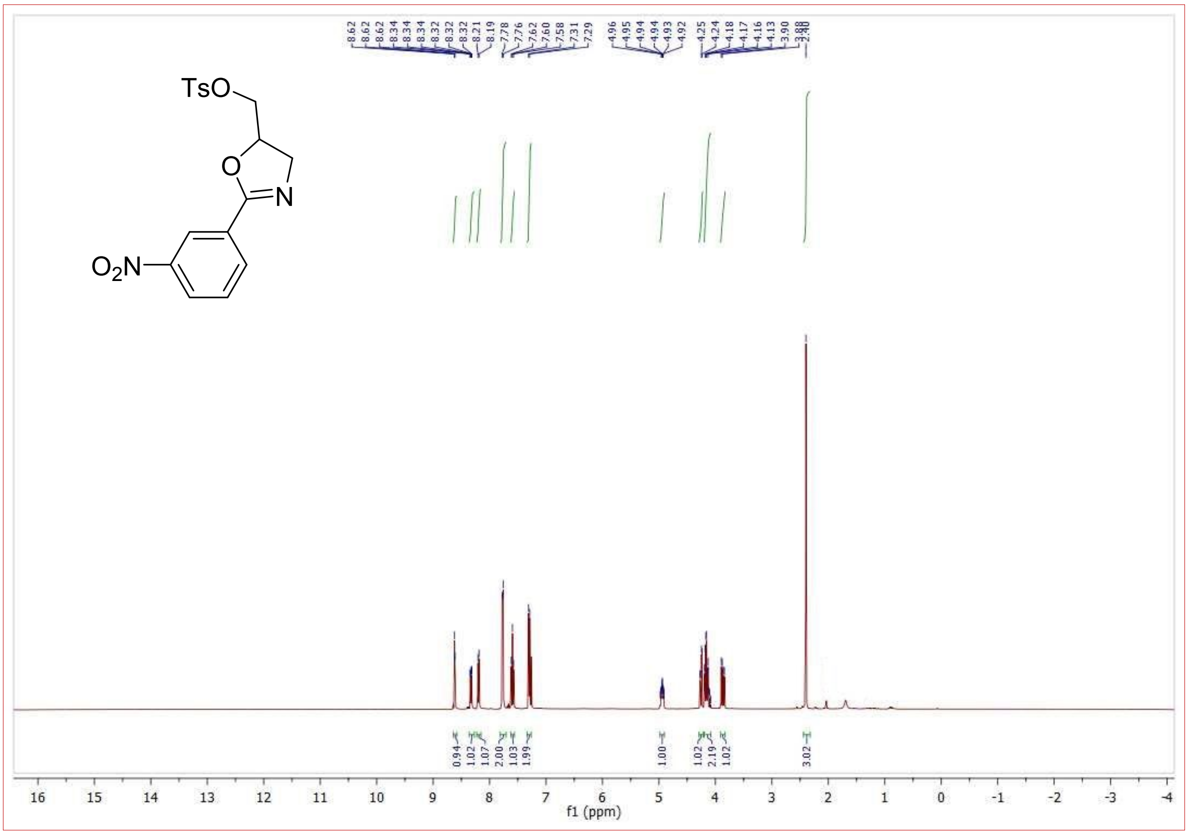

(2-(3-Nitrophenyl)-4,5-dihydrooxazol-5-yl)methyl 4-methylbenzenesulfonate (2i), $^{13}C\{^{1}H\}$ NMR (101 MHz, $CDCl_3$)

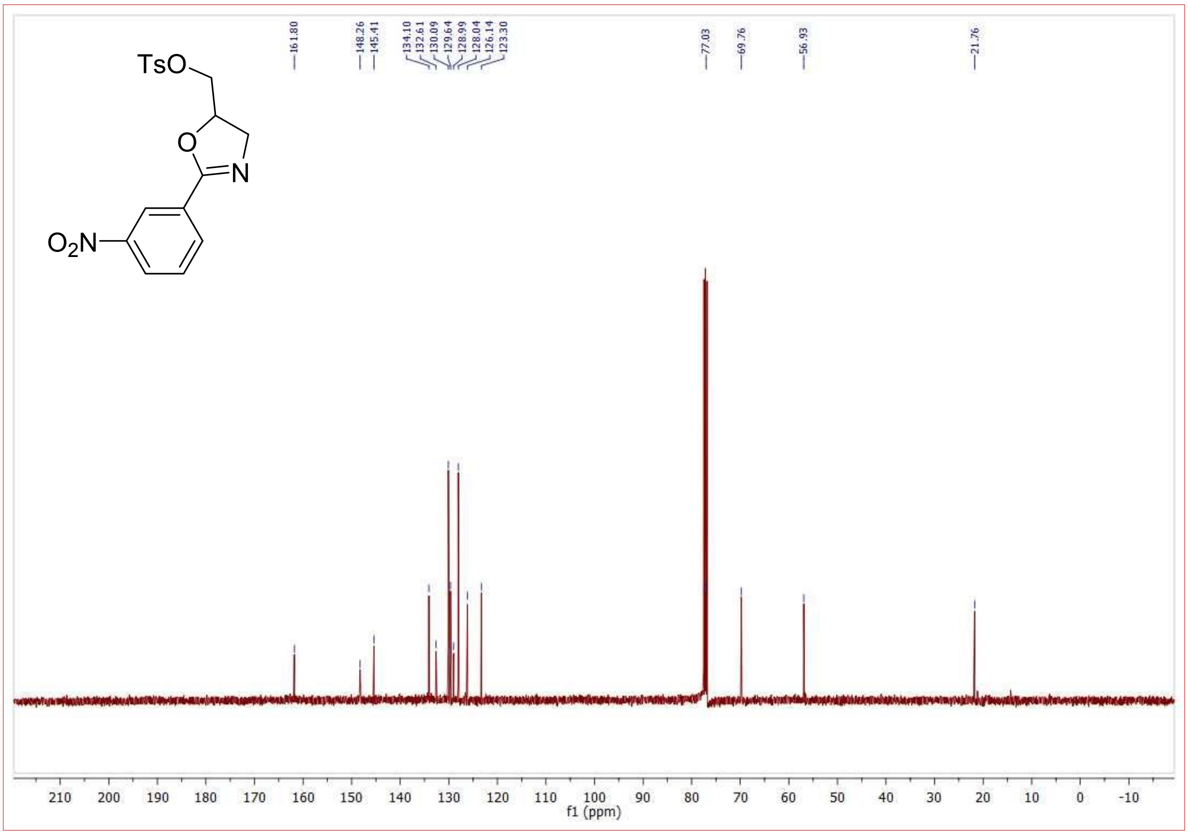

(2-(*o*-Tolyl)-4,5-dihydrooxazol-5-yl)methyl 4-methylbenzenesulfonate (2j), ^{1}H NMR (400 MHz, $CDCl_3$)

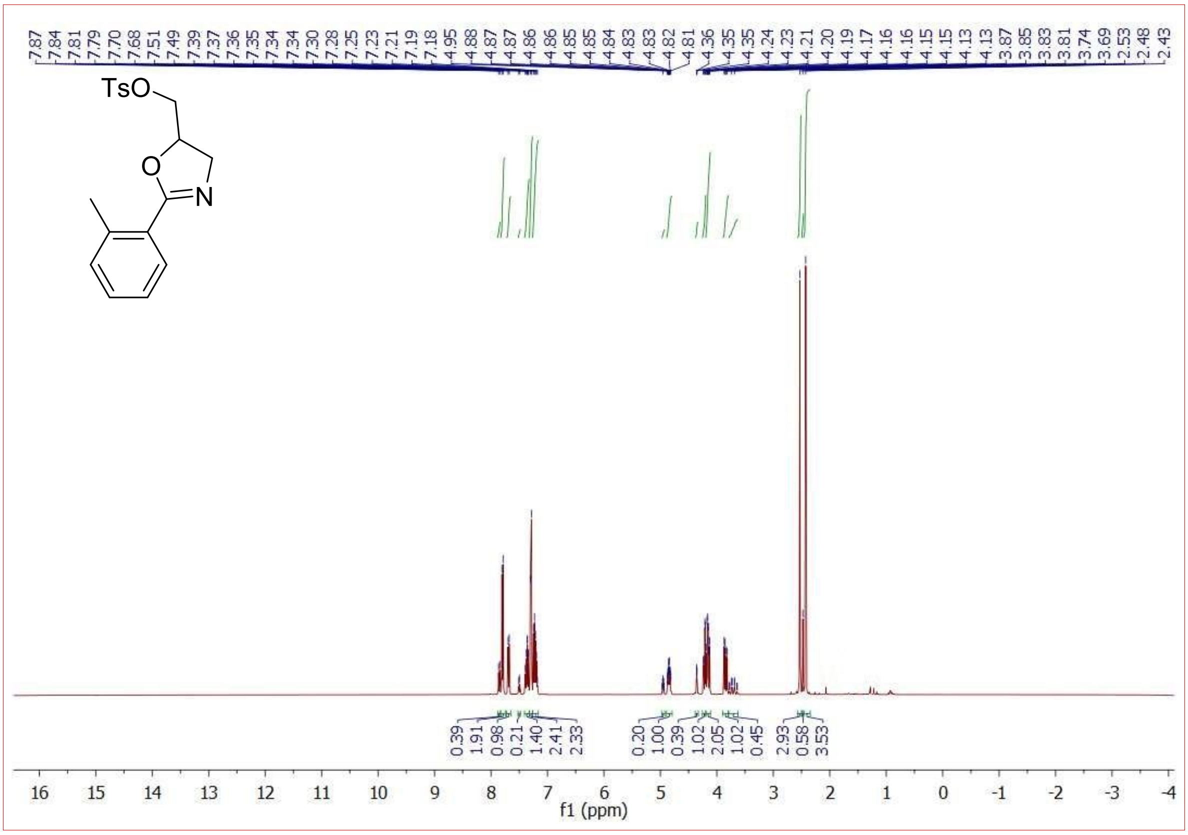

(2-(*o*-Tolyl)-4,5-dihydrooxazol-5-yl)methyl 4-methylbenzenesulfonate (2j), $^{13}C\{^{1}H\}$ NMR (101 MHz, $CDCl_3$)

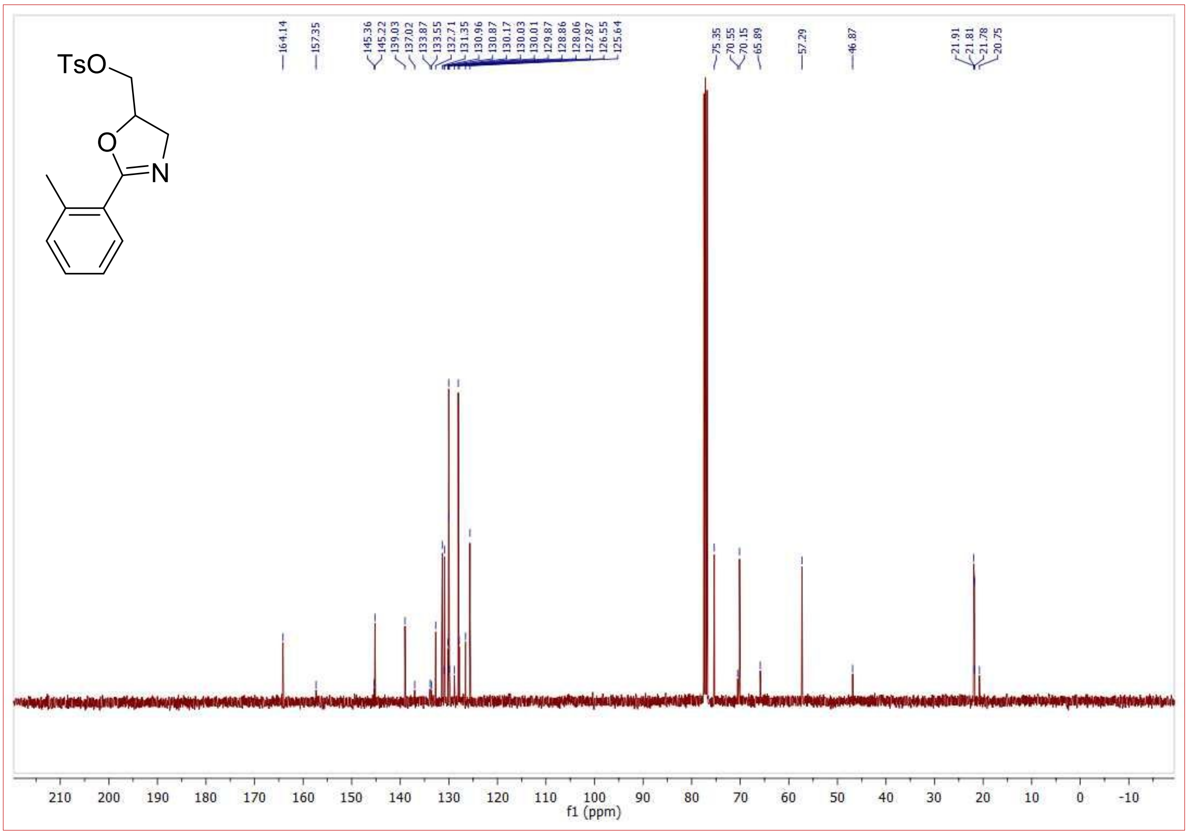

(2-([1,1'-Biphenyl]-2-yl)-4,5-dihydrooxazol-5-yl)methyl 4-methylbenzenesulfonate (2k), ^{1}H NMR (400 MHz, $CDCl_3$)

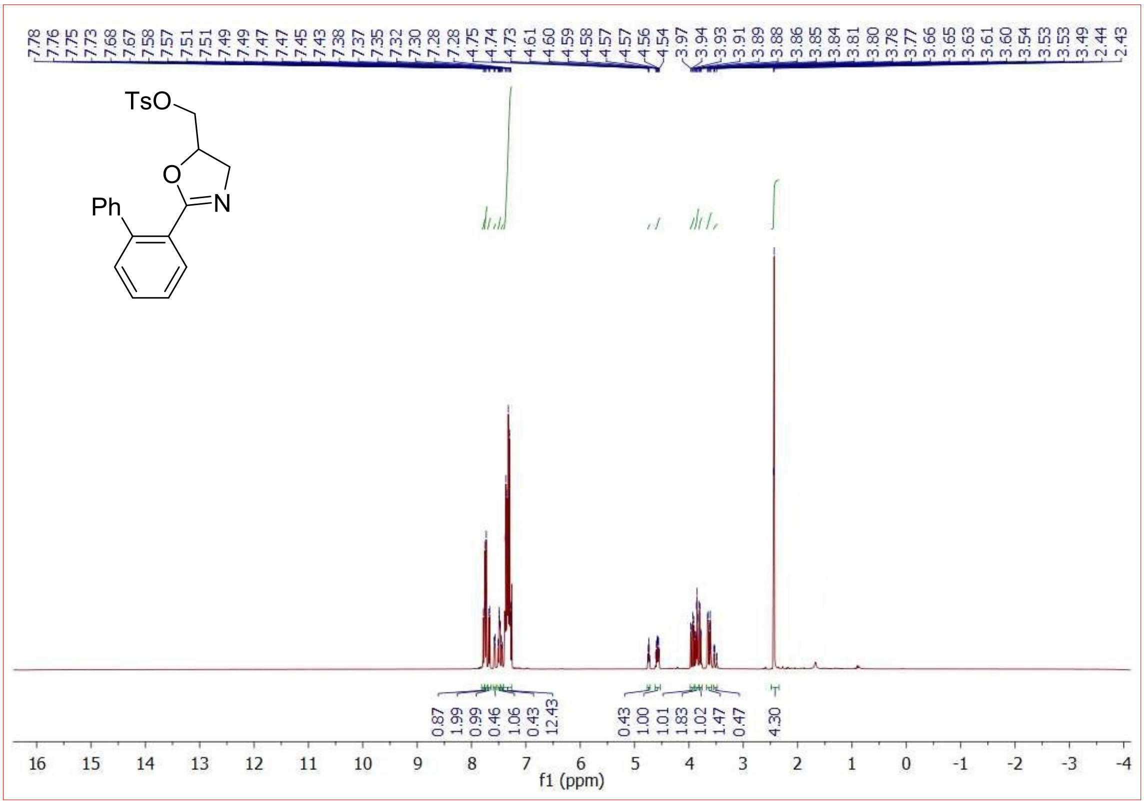

(2-([1,1'-Biphenyl]-2-yl)-4,5-dihydrooxazol-5-yl)methyl 4-methylbenzenesulfonate (2k), $^{13}C\{^1H\}$ NMR (101 MHz, CDCl$_3$)

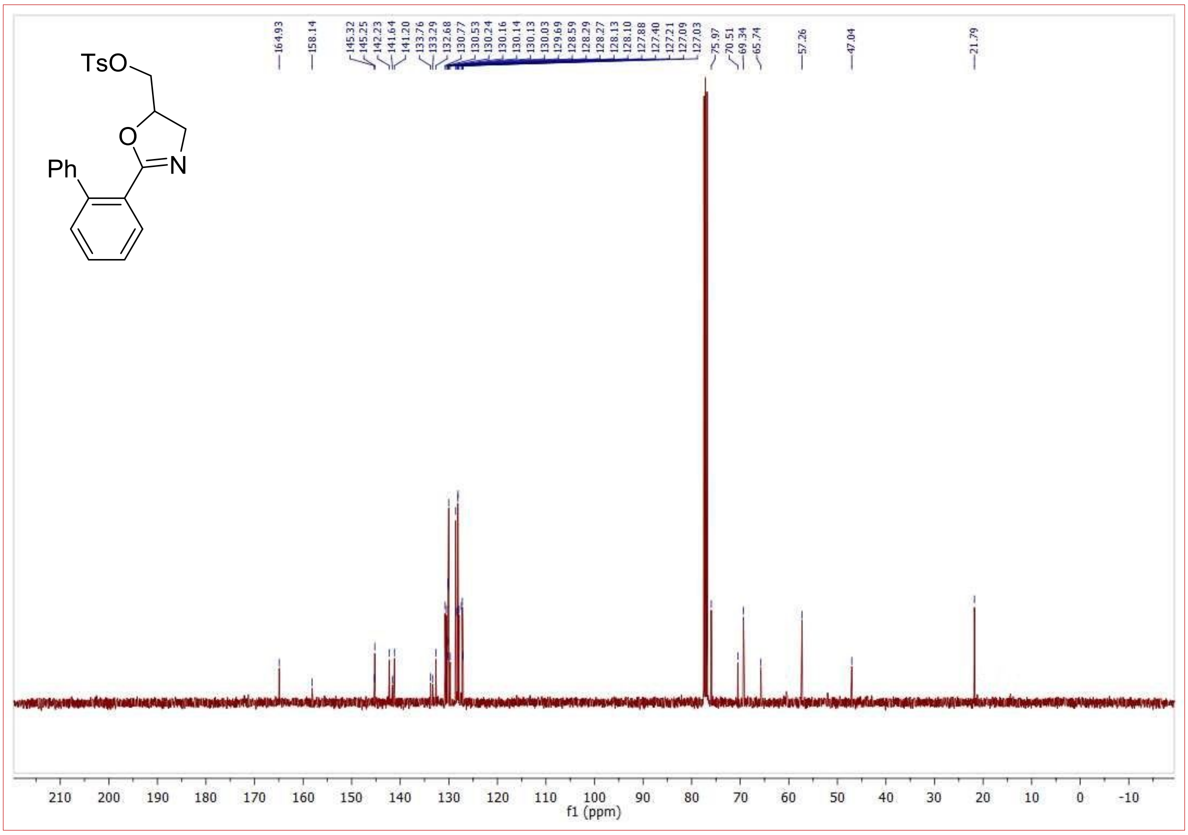

(2-(2-Bromophenyl)-4,5-dihydrooxazol-5-yl)methyl 4-methylbenzenesulfonate (2l), ^1H NMR (400 MHz, CDCl$_3$)

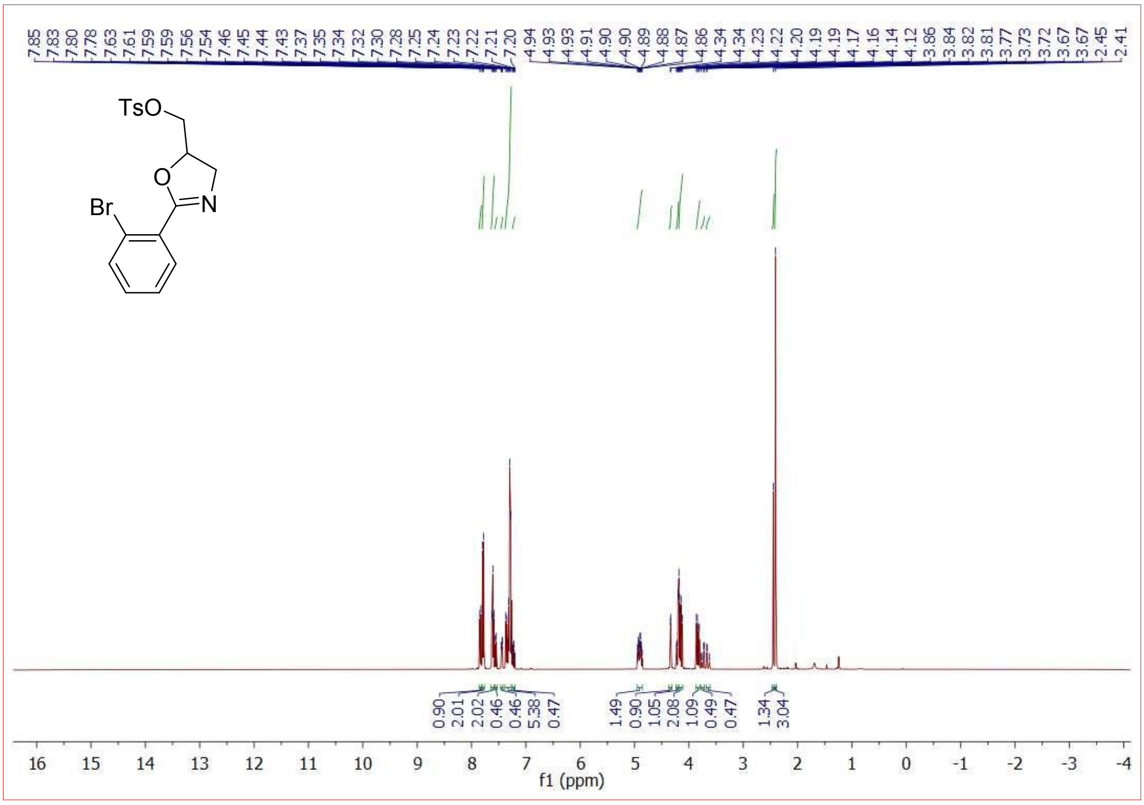

(2-(2-Bromophenyl)-4,5-dihydrooxazol-5-yl)methyl 4-methylbenzenesulfonate (2l), $^{13}C\{^{1}H\}$ NMR (101 MHz, $CDCl_3$)

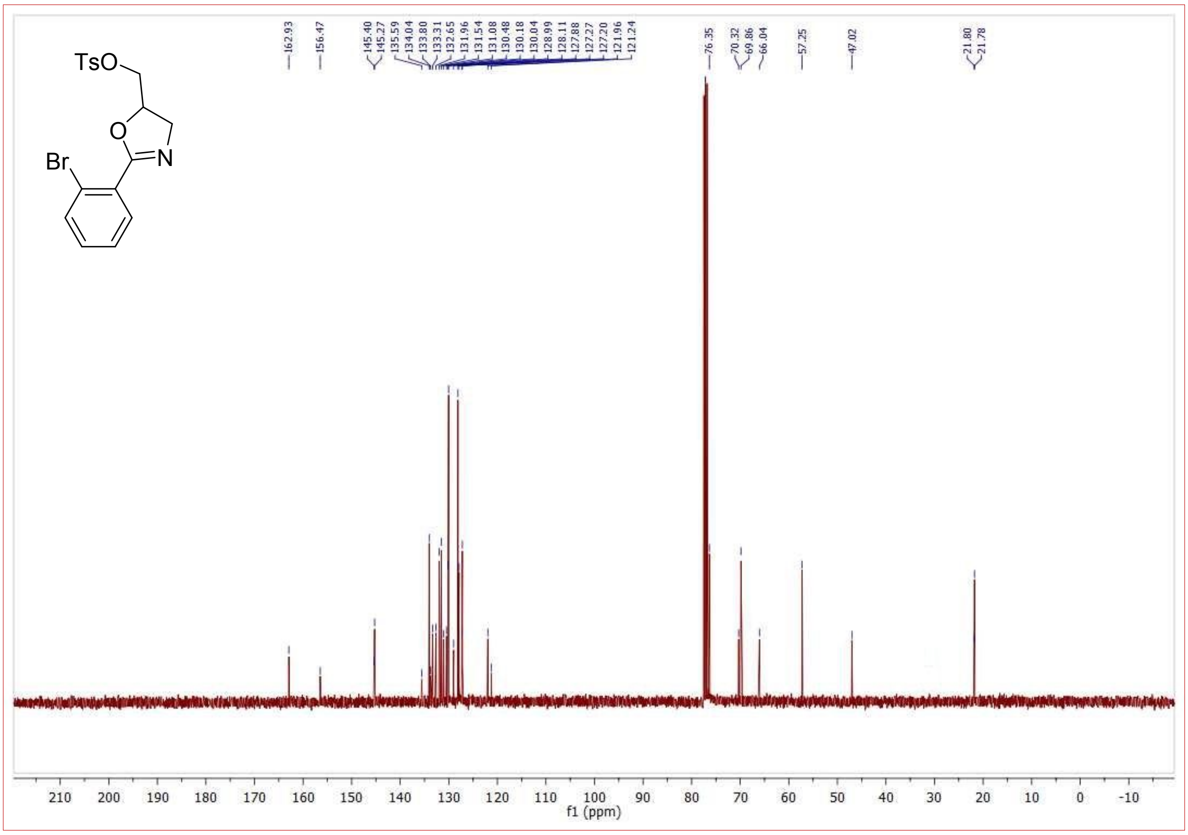

2-(2-Chlorophenyl)-4,5-dihydrooxazol-5-yl)methyl 4-methylbenzenesulfonate (2m), ^{1}H NMR (400 MHz, $CDCl_3$)

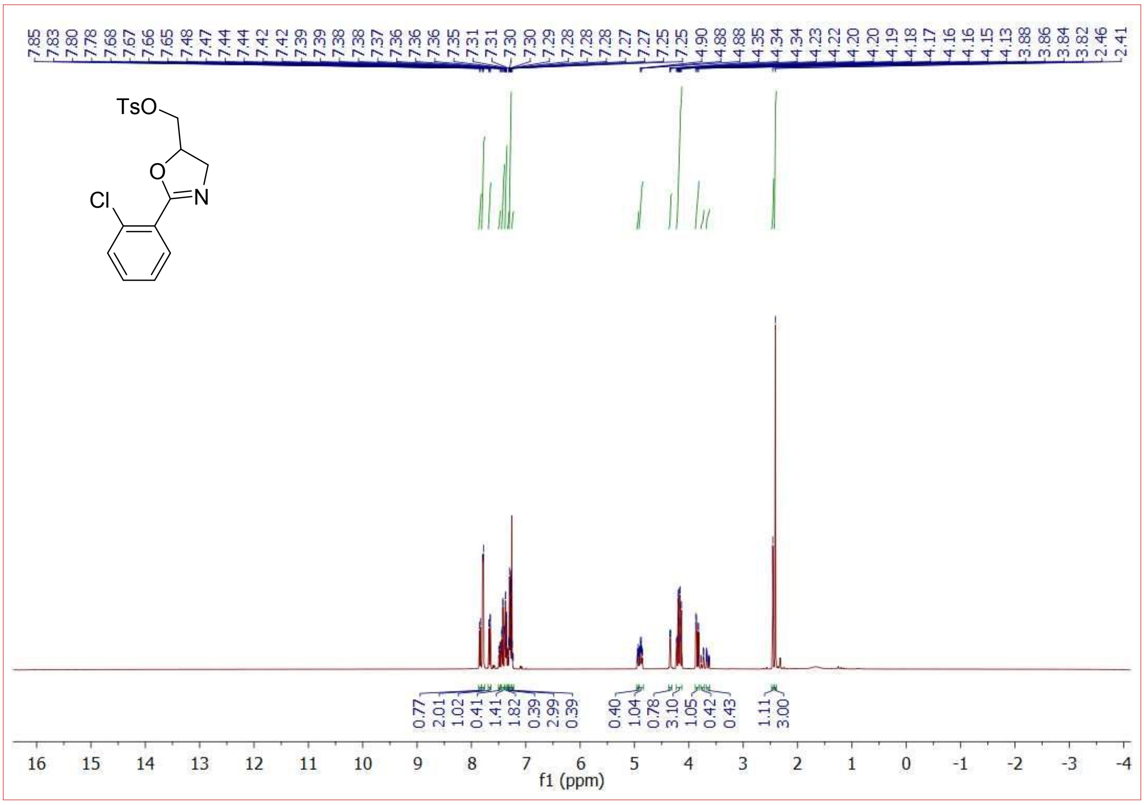

(2-(2-Chlorophenyl)-4,5-dihydrooxazol-5-yl)methyl 4-methylbenzenesulfonate (2m), $^{13}C\{^{1}H\}$ NMR (101 MHz, $CDCl_3$)

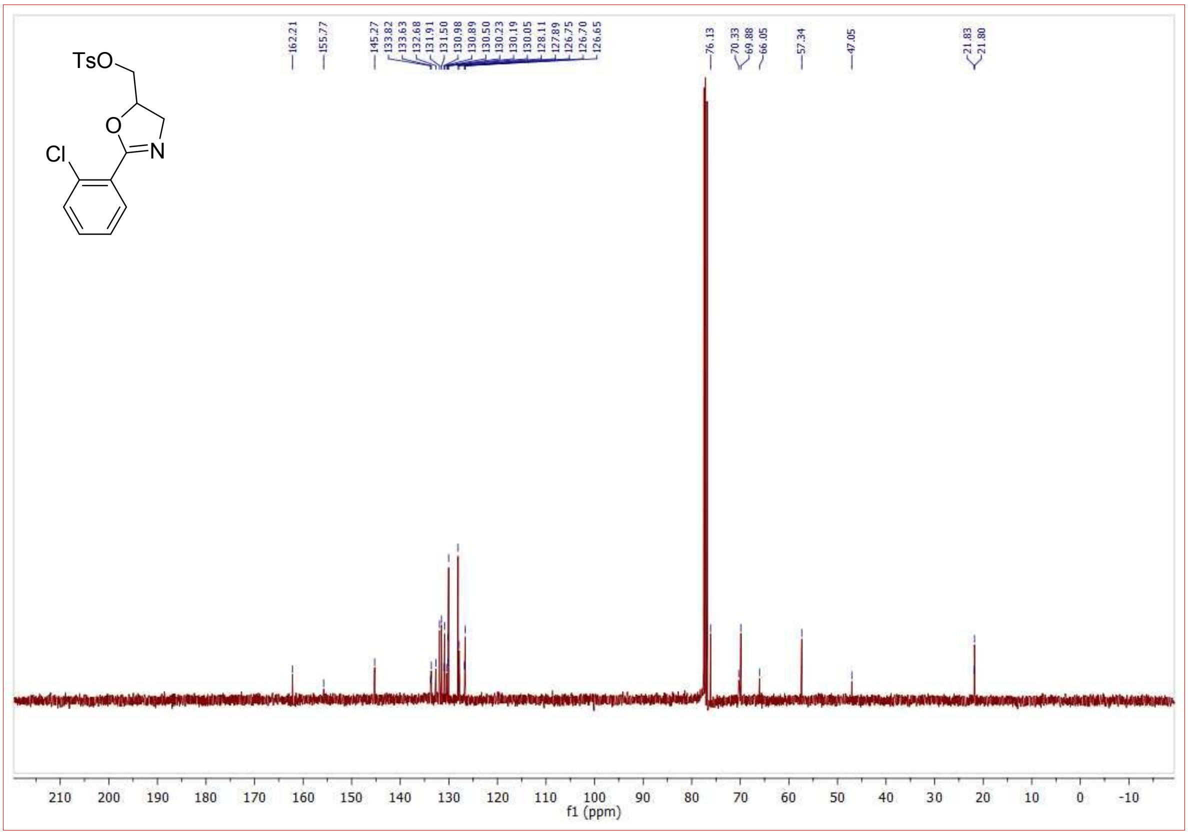

2-(2-Fluorophenyl)-4,5-dihydrooxazol-5-yl)methyl 4-methylbenzenesulfonate (2n), ^{1}H NMR (400 MHz, $CDCl_3$)

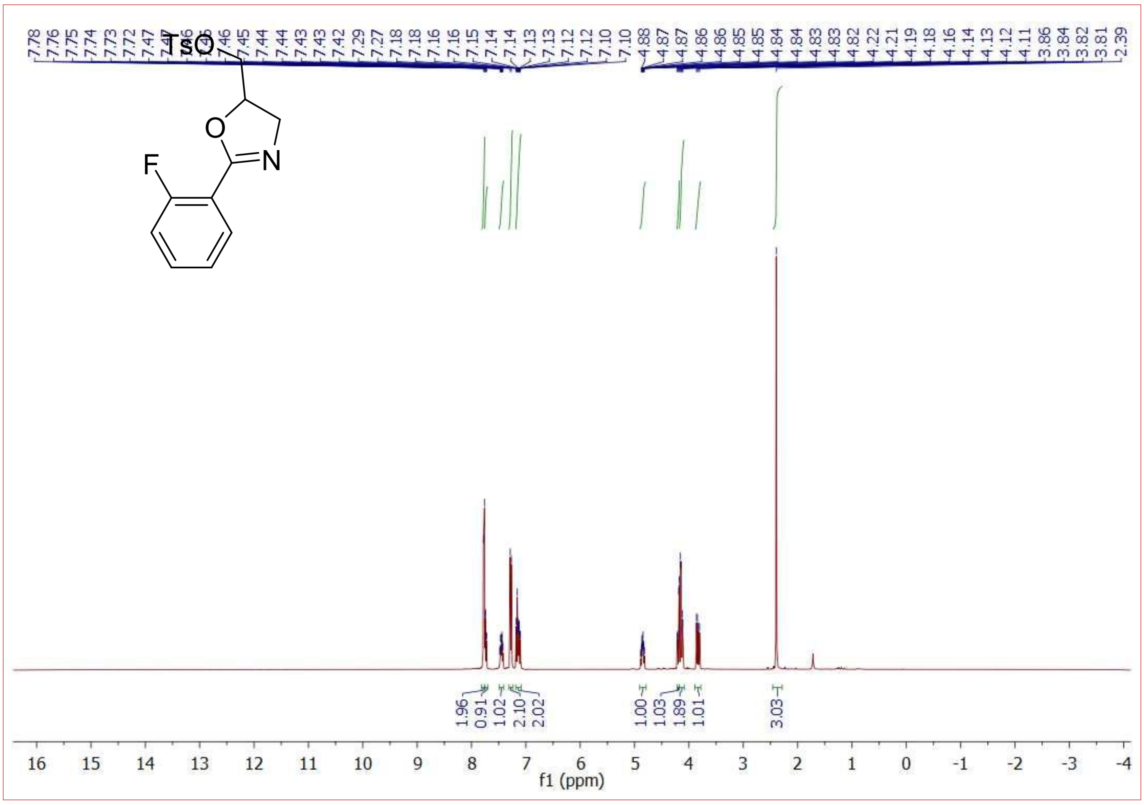

(2-(2-Fluorophenyl)-4,5-dihydrooxazol-5-yl)methyl 4-methylbenzenesulfonate (2n), $^{13}C\{^{1}H\}$ NMR (101 MHz, $CDCl_3$)

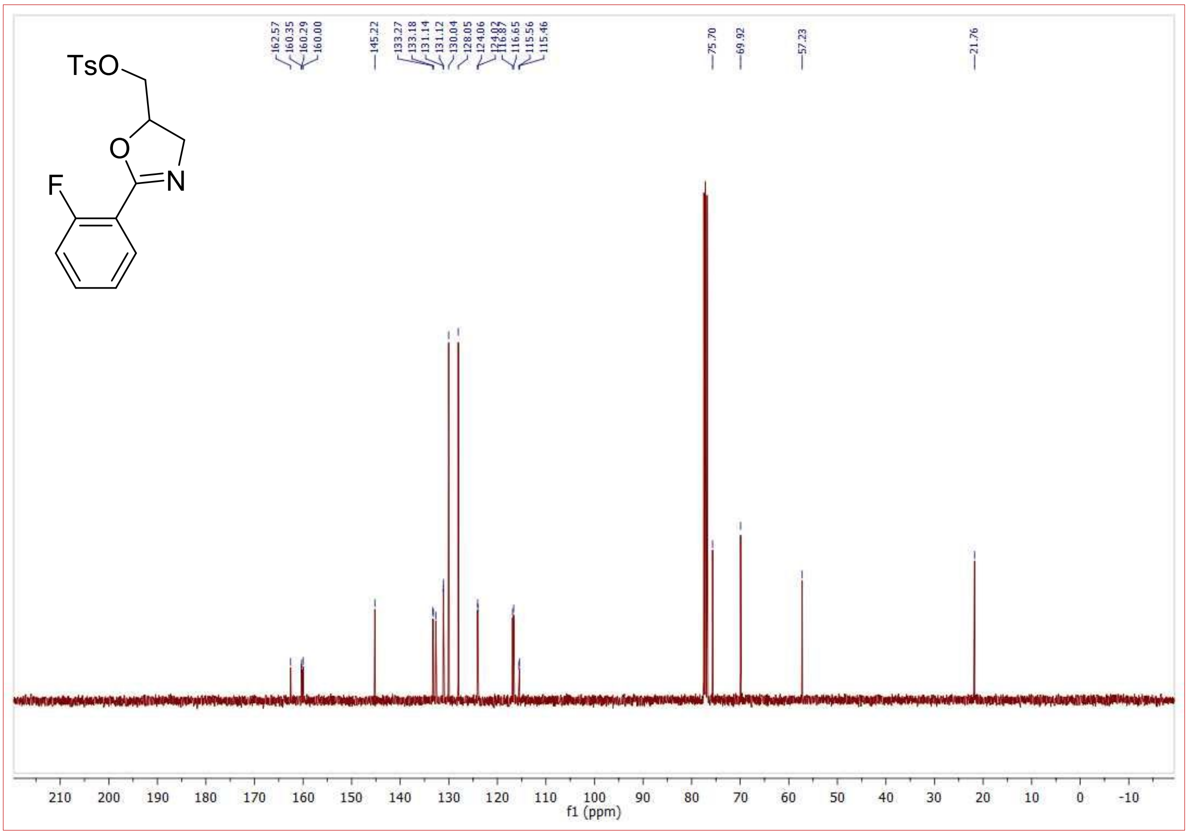

(2-(2-Fluorophenyl)-4,5-dihydrooxazol-5-yl)methyl 4-methylbenzenesulfonate (2n), ^{19}F NMR (376 MHz, $CDCl_3$)

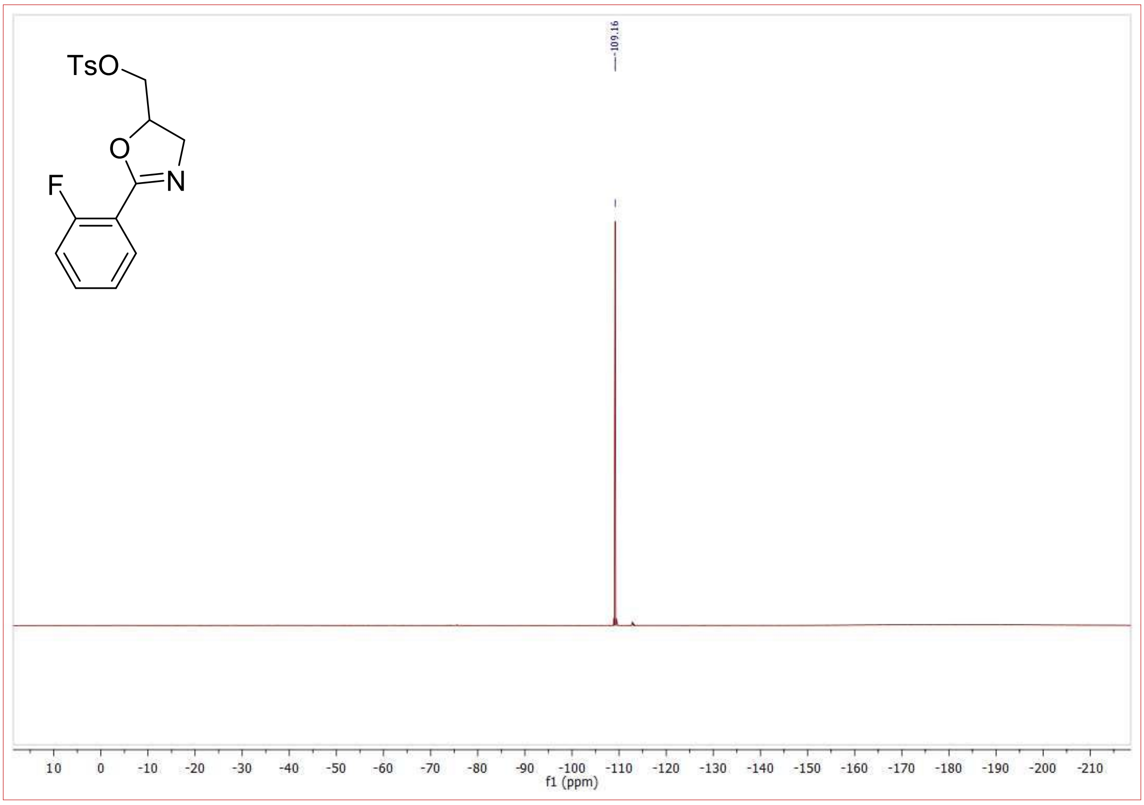

(5-Methyl-2-phenyl-4,5-dihydrooxazol-5-yl)methyl 4-methylbenzenesulfonate (2o), ^{1}H NMR (400 MHz, $CDCl_3$)

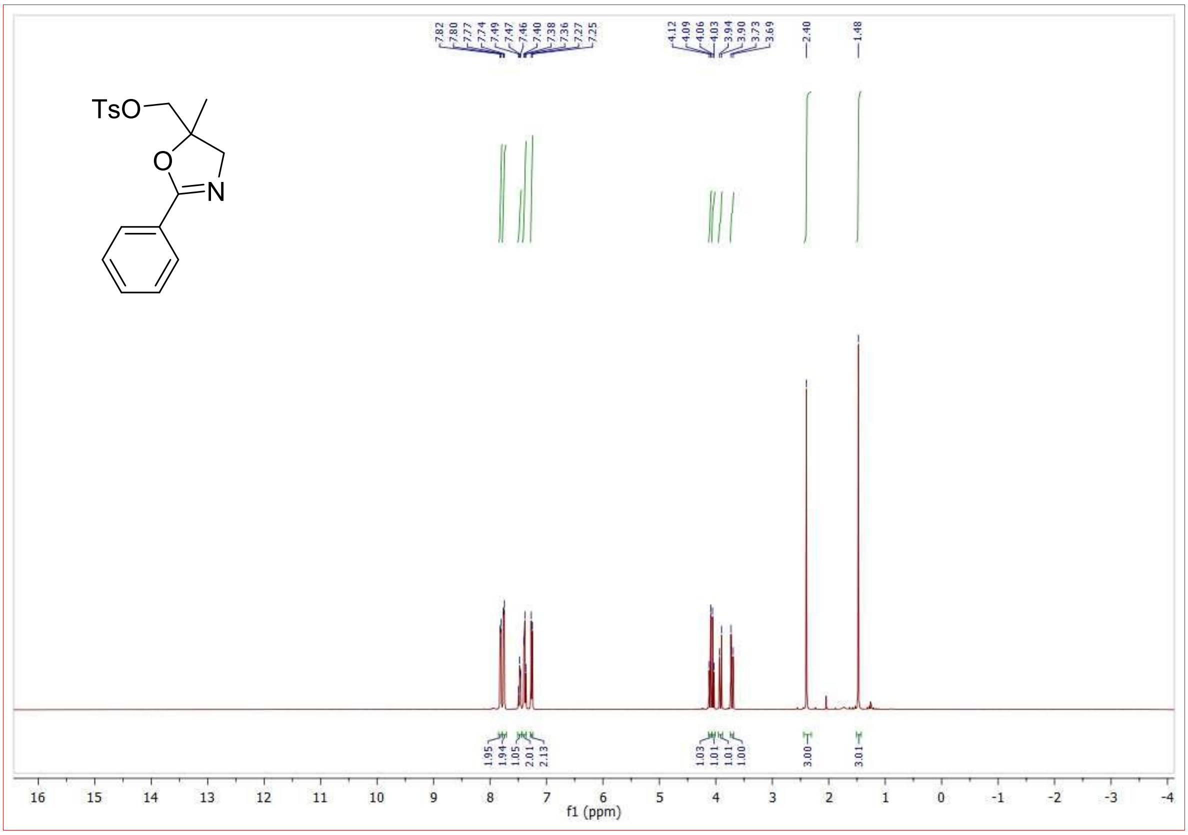

5-Methyl-2-phenyl-4,5-dihydrooxazol-5-yl)methyl 4-methylbenzenesulfonate (2o), $^{13}C\{^{1}H\}$ NMR (101 MHz, $CDCl_3$)

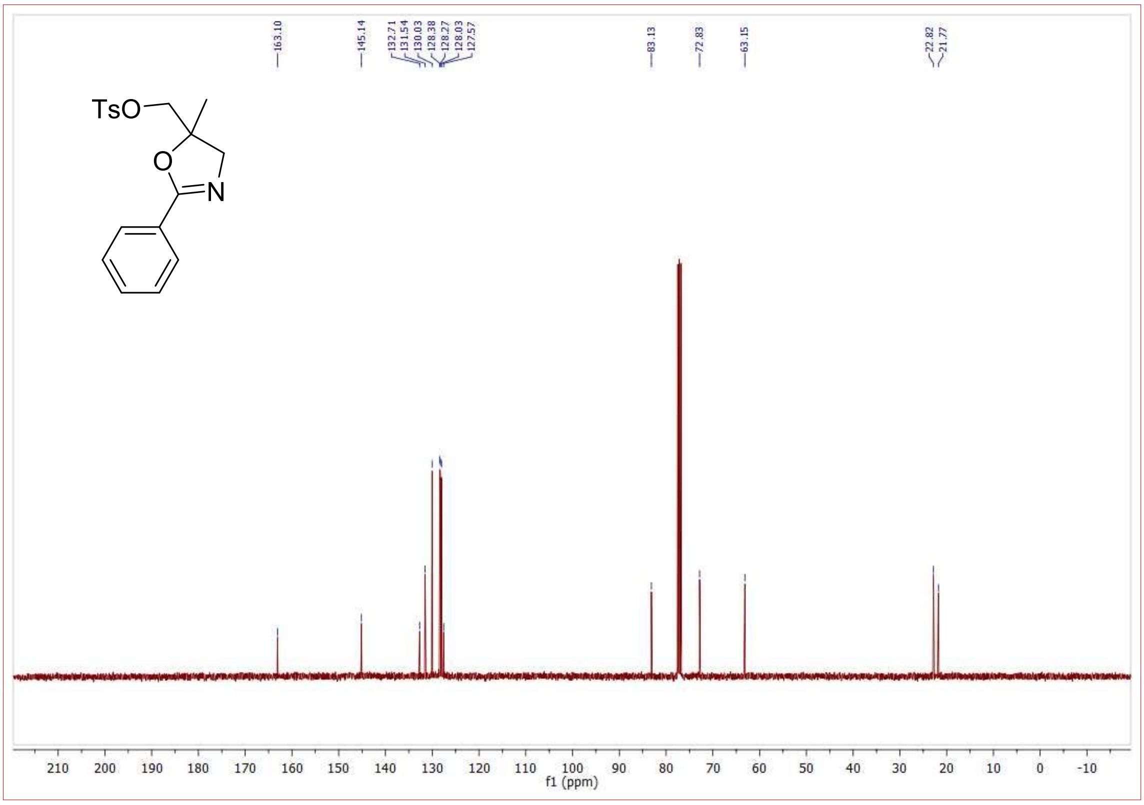

(2-(4-Chlorophenyl)-5-methyl-4,5-dihydrooxazol-5-yl)methyl 4-methylbenzenesulfonate (2P), ^{1}H NMR (400 MHz, $CDCl_3$)

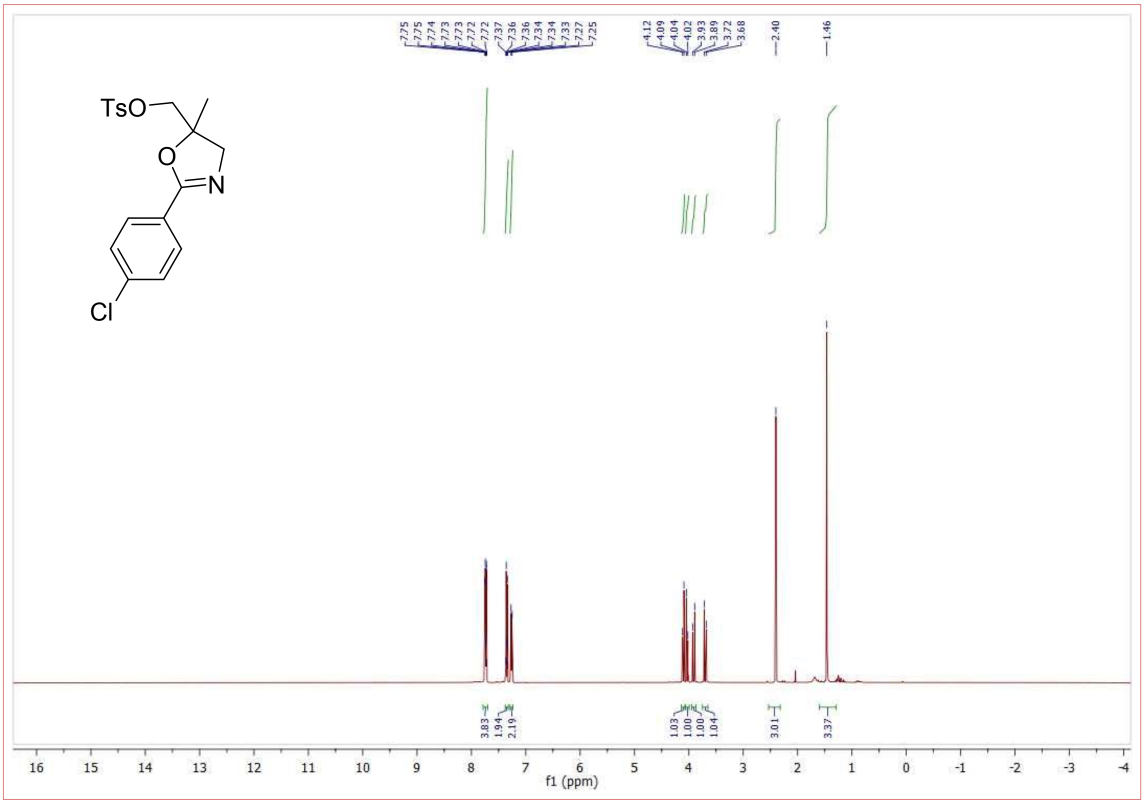

(2-(4-Chlorophenyl)-5-methyl-4,5-dihydrooxazol-5-yl)methyl 4-methylbenzenesulfonate (2P), $^{13}C\{^{1}H\}$ NMR (101 MHz, $CDCl_3$)

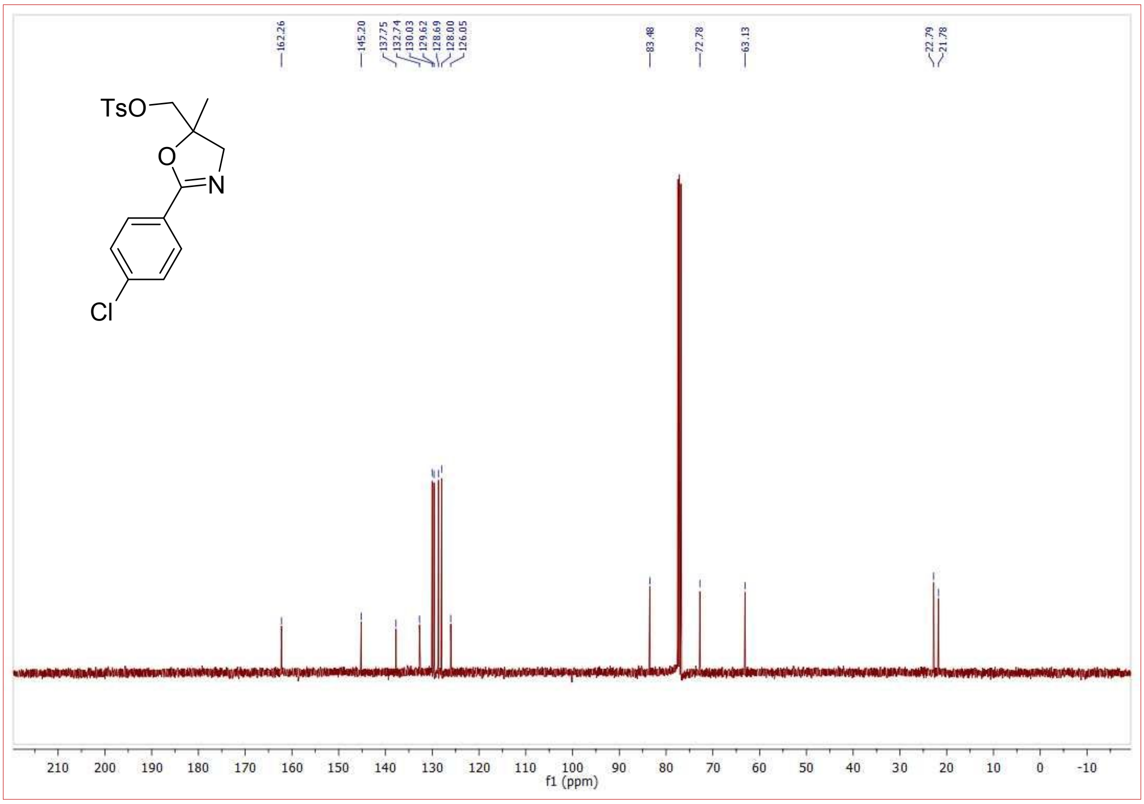

(2-(2-Fluoro-4-methoxyphenyl)-4,5-dihydrooxazol-5-yl)methyl 4-methylbenzenesulfonate (2q), ^{1}H NMR (400 MHz, $CDCl_3$)

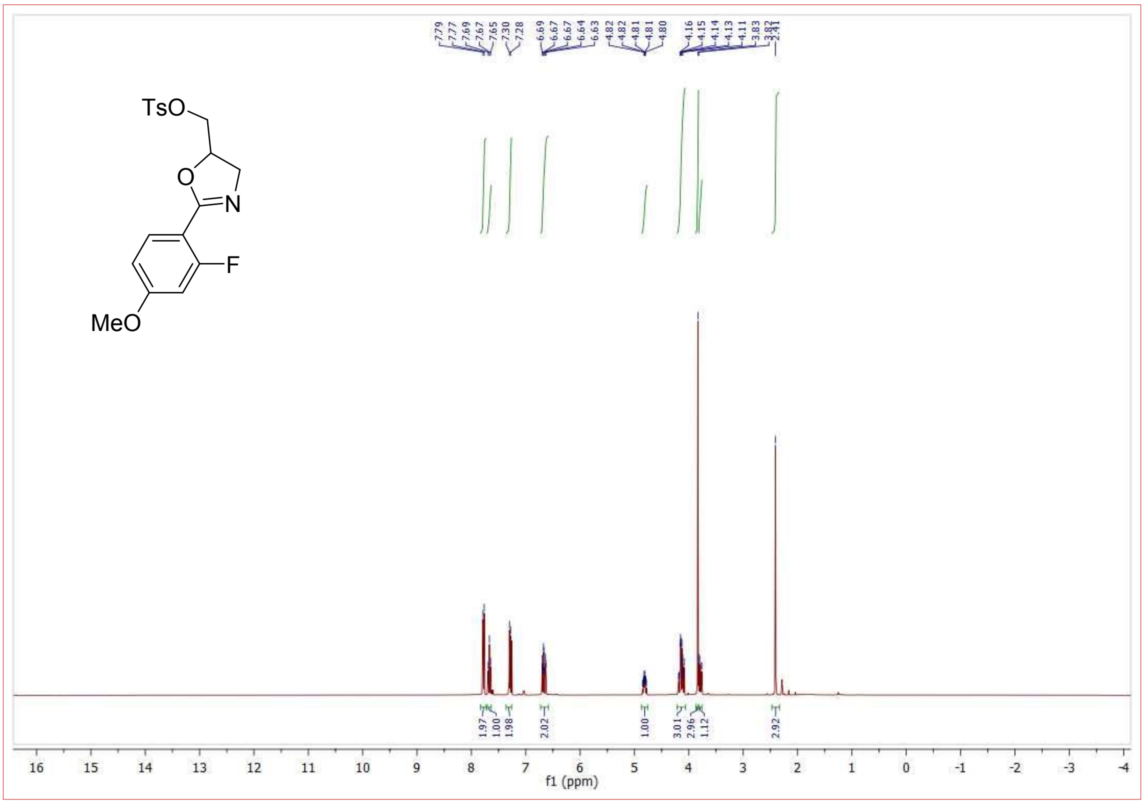

(2-(2-Fluoro-4-methoxyphenyl)-4,5-dihydrooxazol-5-yl)methyl 4-methylbenzenesulfonate (2q), $^{13}C\{^1H\}$ NMR (101 MHz, $CDCl_3$)

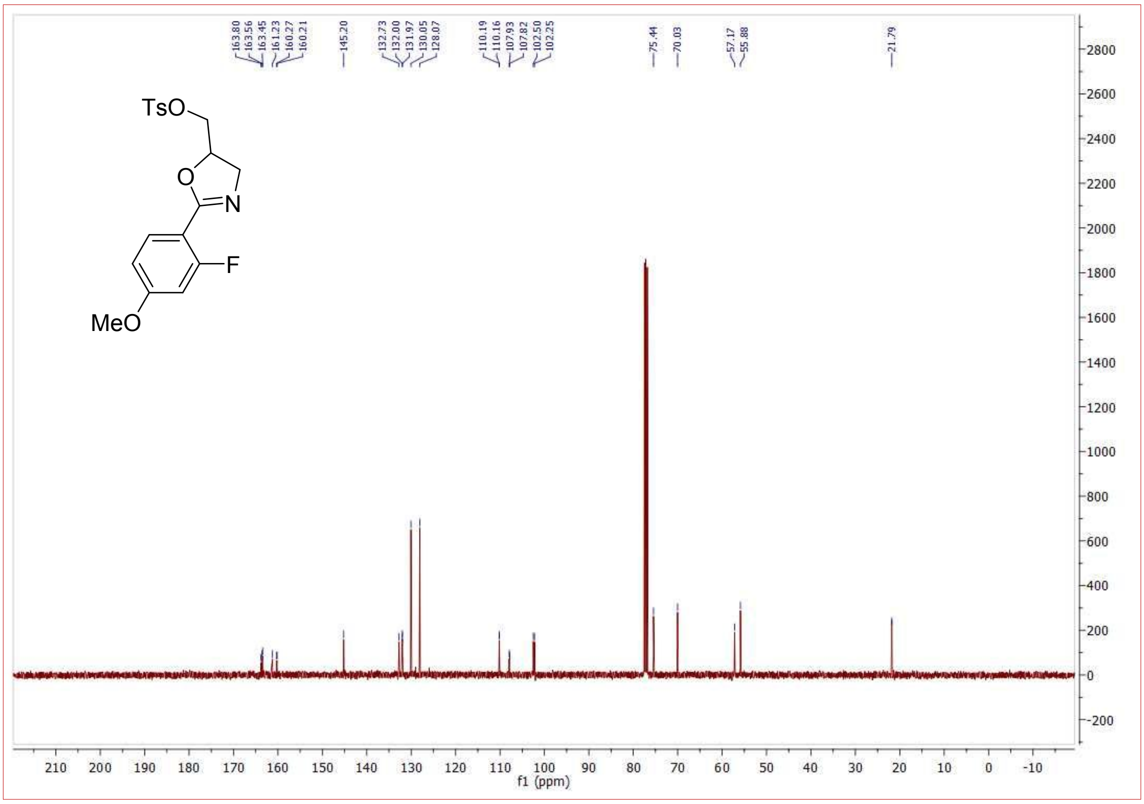

(2-(2-Fluoro-4-methoxyphenyl)-4,5-dihydrooxazol-5-yl)methyl 4-methylbenzenesulfonate (2q), ^{19}F NMR (376 MHz, $CDCl_3$)

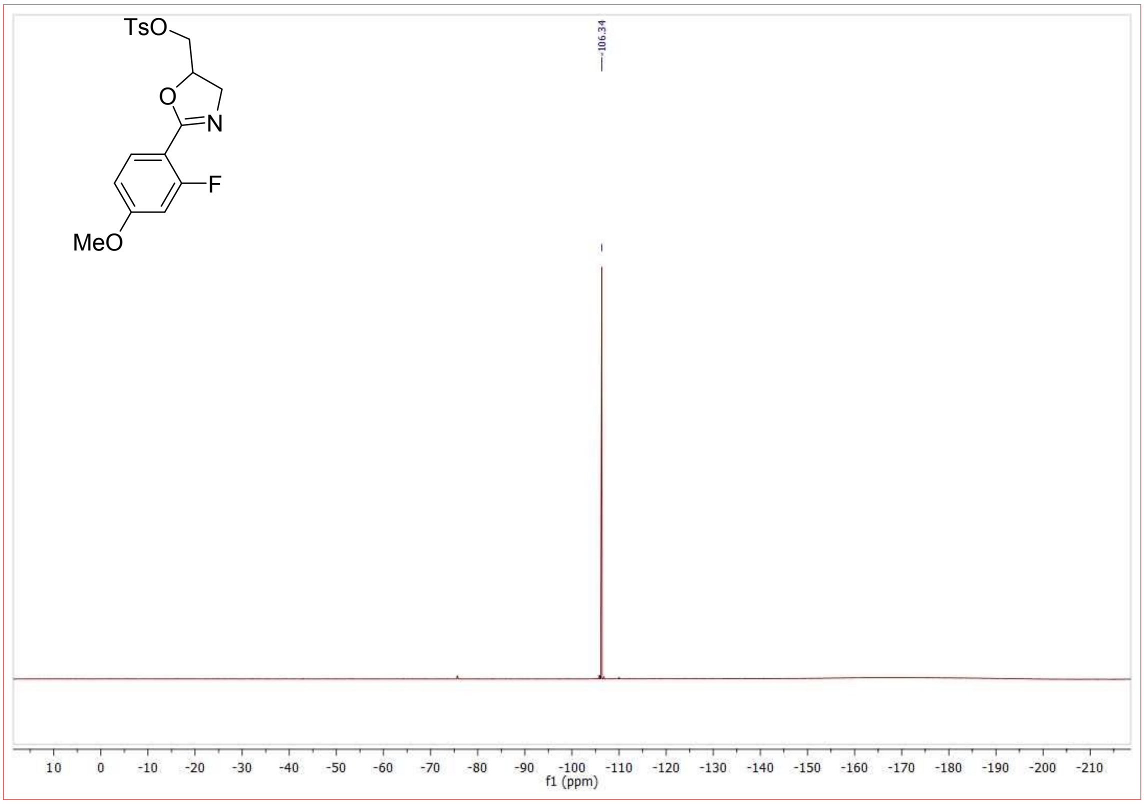

2-(3-Fluoro-4-methylphenyl)-4,5-dihydrooxazol-5-yl)methyl 4-methylbenzenesulfonate (2r), ^{1}H NMR (400 MHz, $CDCl_3$)

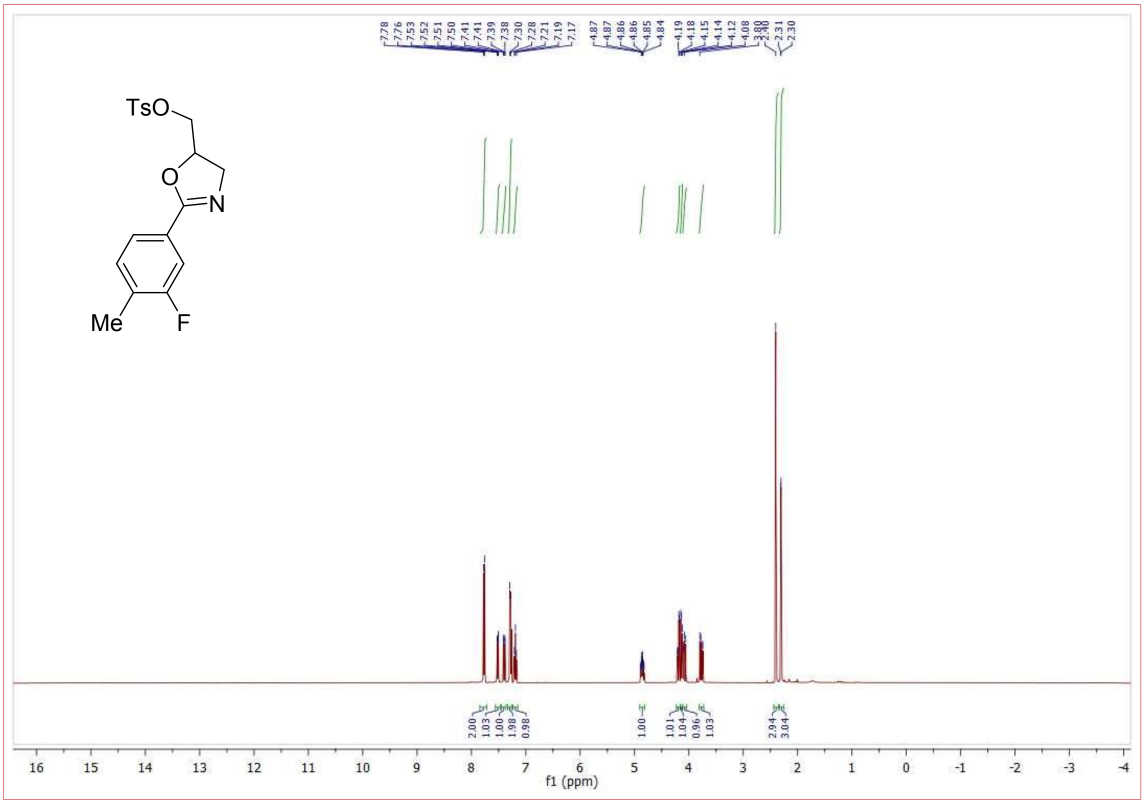

2-(3-Fluoro-4-methylphenyl)-4,5-dihydrooxazol-5-yl)methyl 4-methylbenzenesulfonate (2r), $^{13}C\{^1H\}$ NMR (101 MHz, $CDCl_3$)

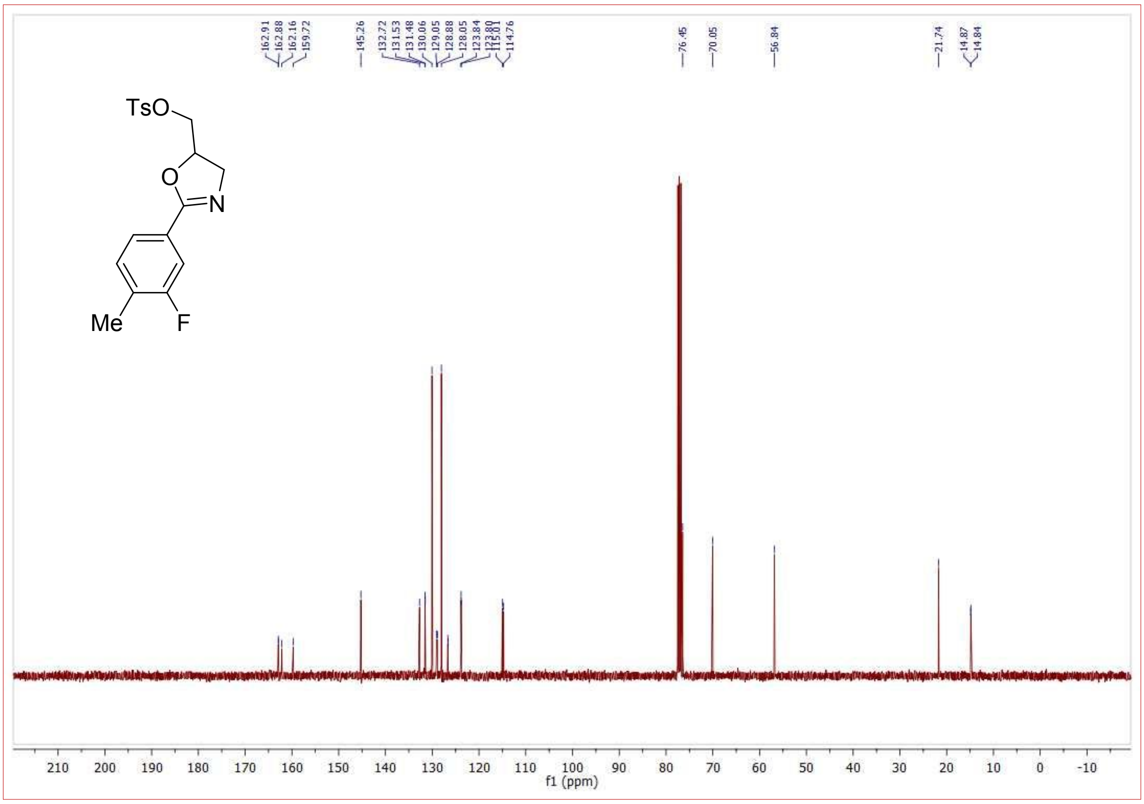

2-(3-Fluoro-4-methylphenyl)-4,5-dihydrooxazol-5-yl)methyl 4-methylbenzenesulfonate (2r), ^{19}F NMR (376 MHz, $CDCl_3$)

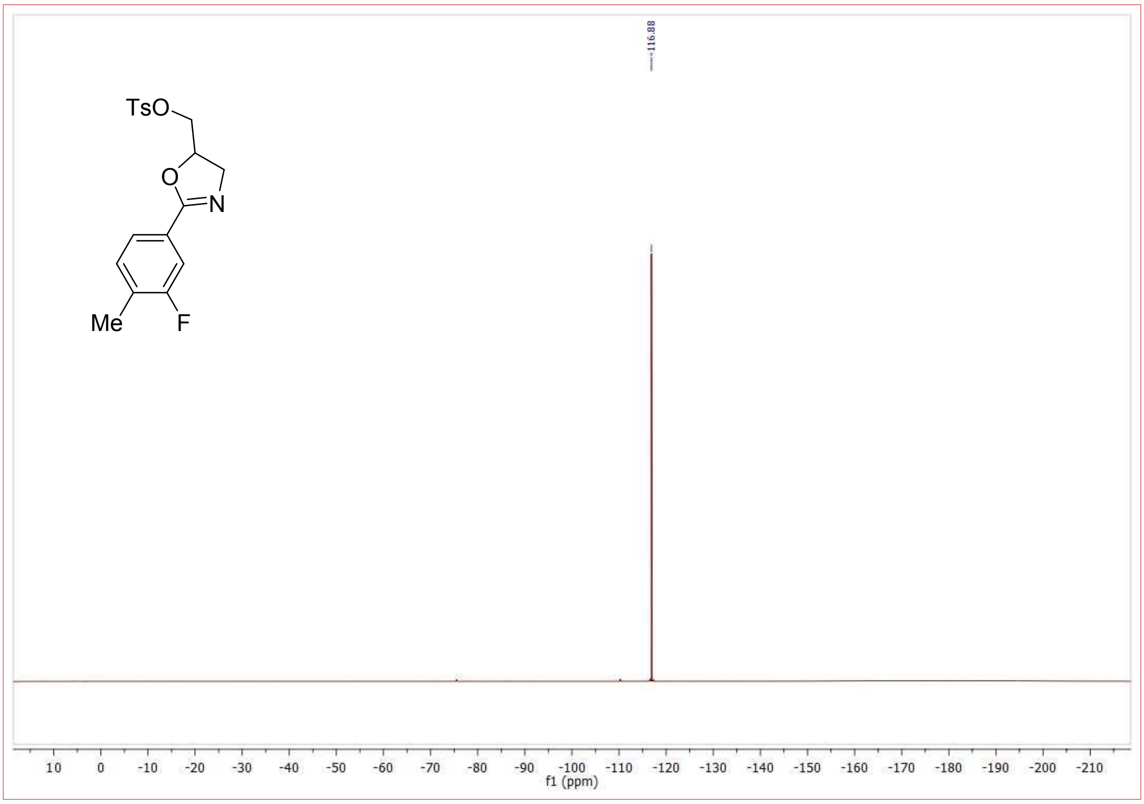

(2-Benzyl-4,5-dihydrooxazol-5-yl)methyl 4-methylbenzenesulfonate (2s), ^{1}H NMR (400 MHz, CDCl$_3$)

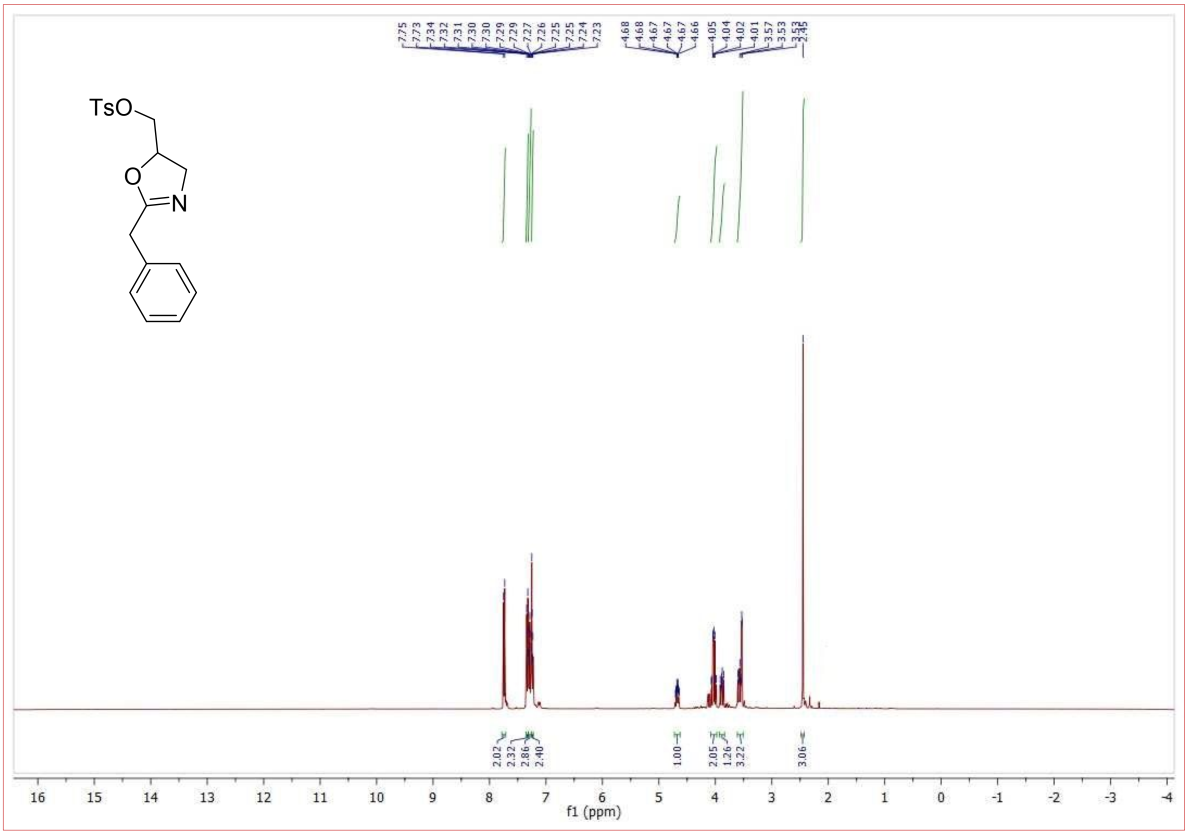

(2-Benzyl-4,5-dihydrooxazol-5-yl)methyl 4-methylbenzenesulfonate (2s), ^{13}C{^{1}H} NMR (101 MHz, CDCl$_3$)

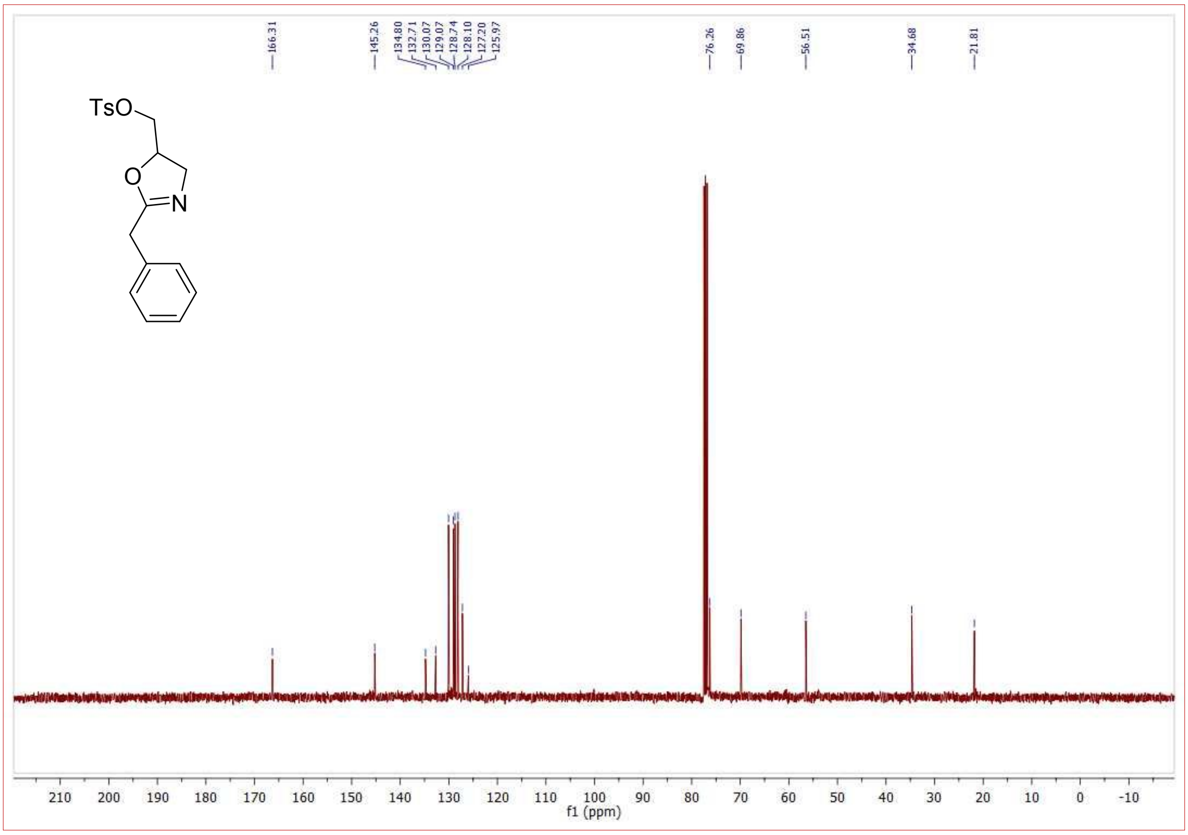

(2-(4-Chlorobenzyl)-4,5-dihydrooxazol-5-yl)methyl 4-methylbenzenesulfonate (2t), ^{1}H NMR (400 MHz, CDCl$_3$)

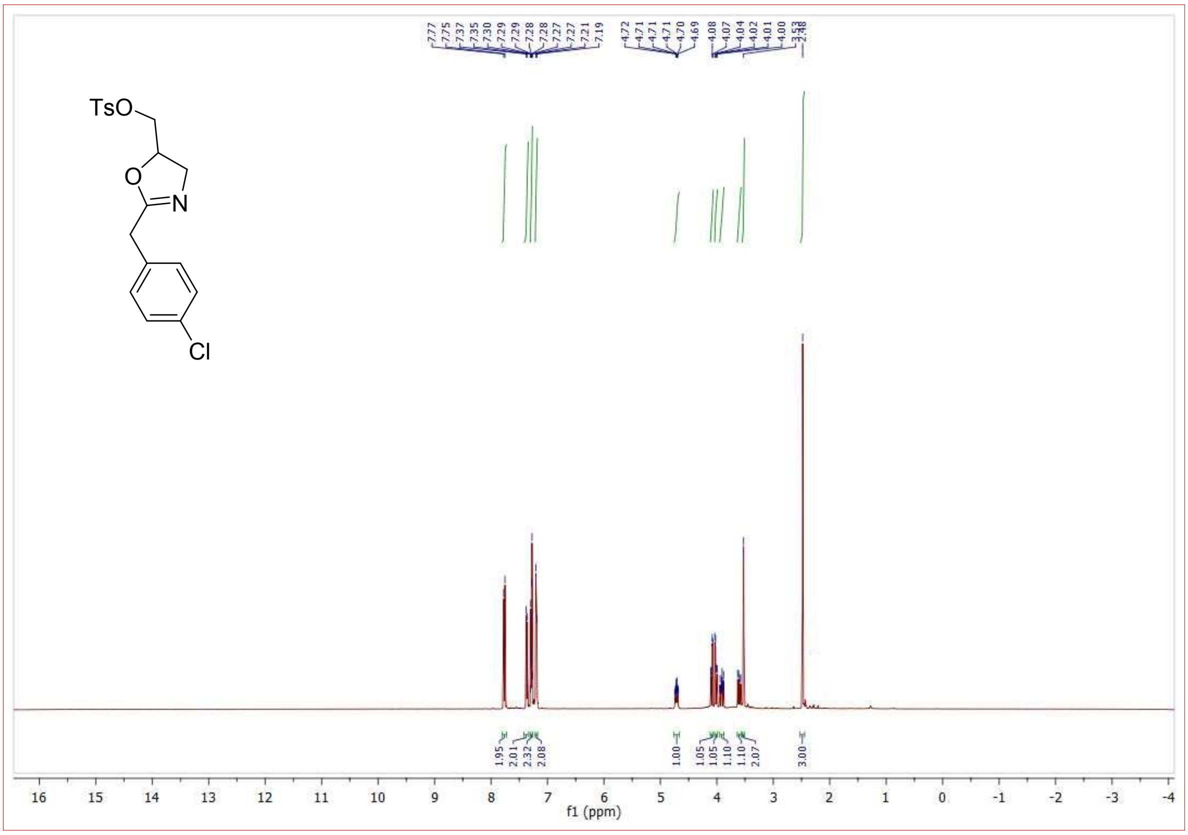

(2-(4-Chlorobenzyl)-4,5-dihydrooxazol-5-yl)methyl 4-methylbenzenesulfonate (2t), ^{13}C{^{1}H} NMR (101 MHz, CDCl$_3$)

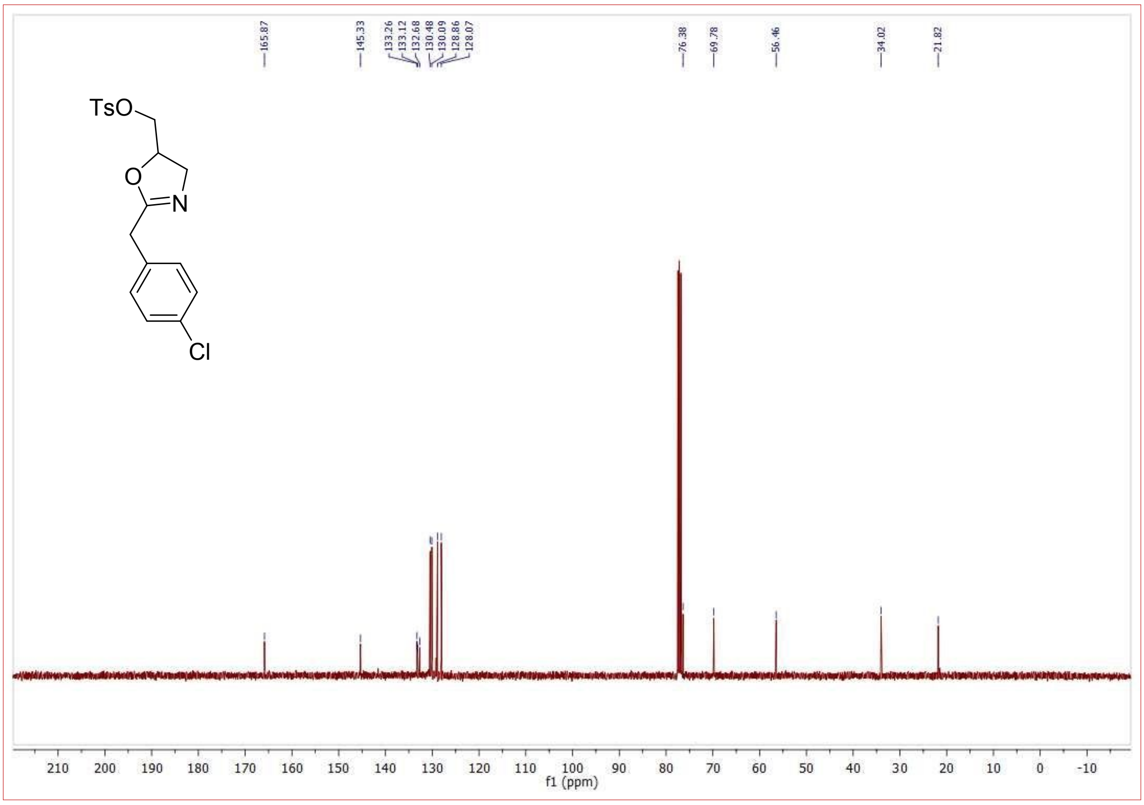

(2-(*tert*-Butyl)-4,5-dihydrooxazol-5-yl)methyl 4-methylbenzenesulfonate (2w), ^{1}H NMR (400 MHz, CDCl$_3$)

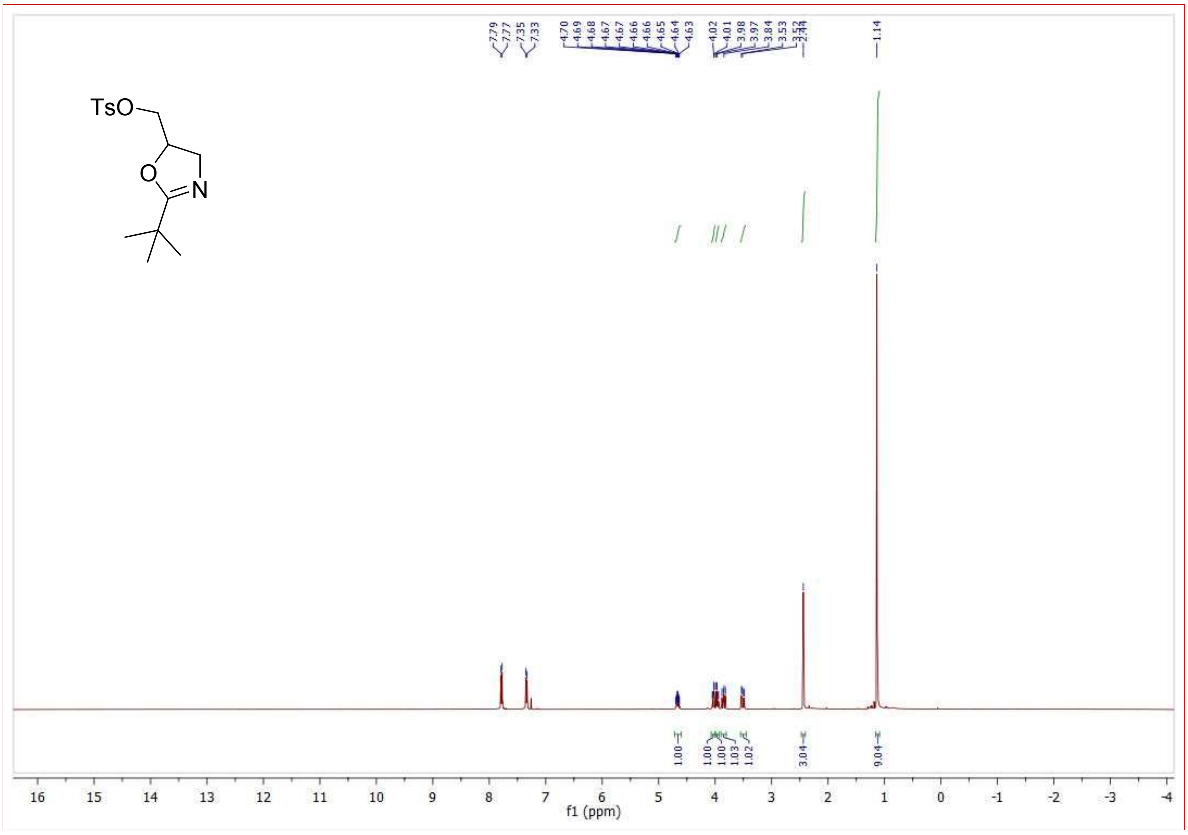

(2-(*tert*-Butyl)-4,5-dihydrooxazol-5-yl)methyl 4-methylbenzenesulfonate (2w), ^{13}C{^{1}H} NMR (101 MHz, CDCl$_3$)

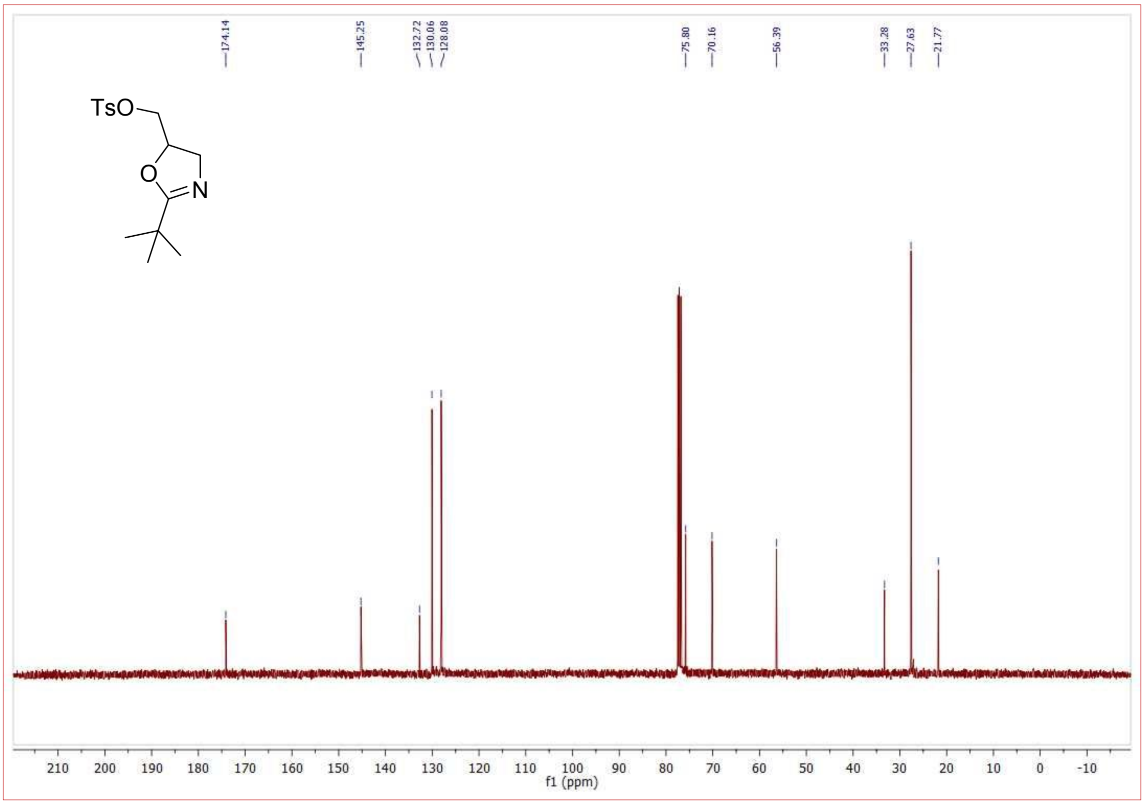

(2-(Furan-2-yl)-4,5-dihydrooxazol-5-yl)methyl 4-methylbenzenesulfonate (2x), ^{1}H NMR (400 MHz, $CDCl_3$)

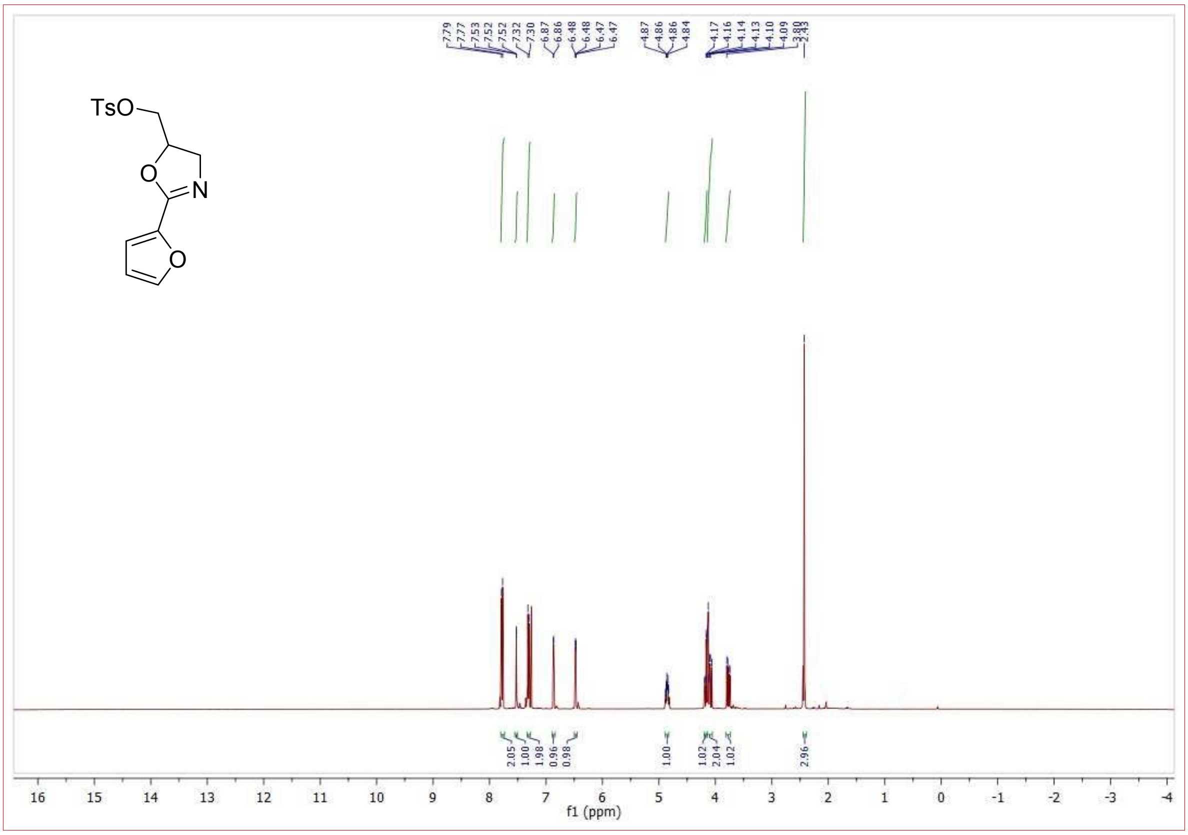

(2-(Furan-2-yl)-4,5-dihydrooxazol-5-yl)methyl 4-methylbenzenesulfonate (2x), ^{13}C{^{1}H} NMR (101 MHz, $CDCl_3$)

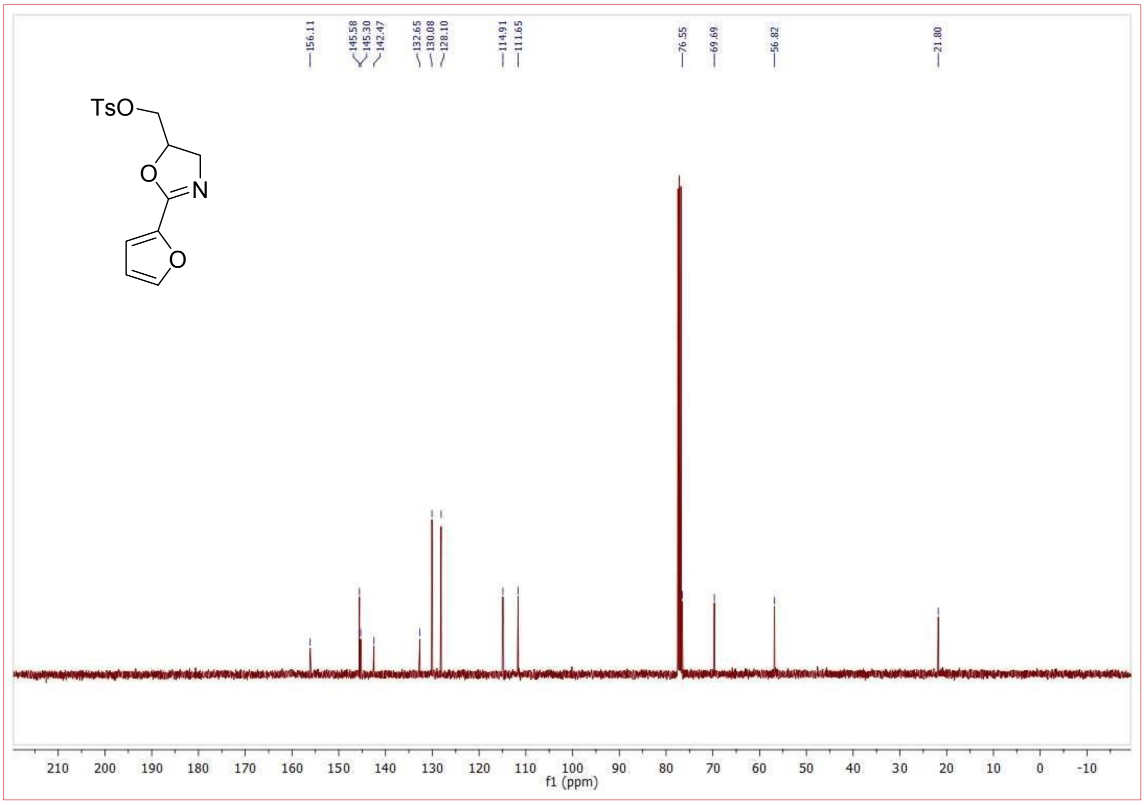

(2-(Furan-2-yl)-5-methyl-4,5-dihydrooxazol-5-yl)methyl 4-methylbenzenesulfonate (2y), ^{1}H NMR (400 MHz, $CDCl_3$)

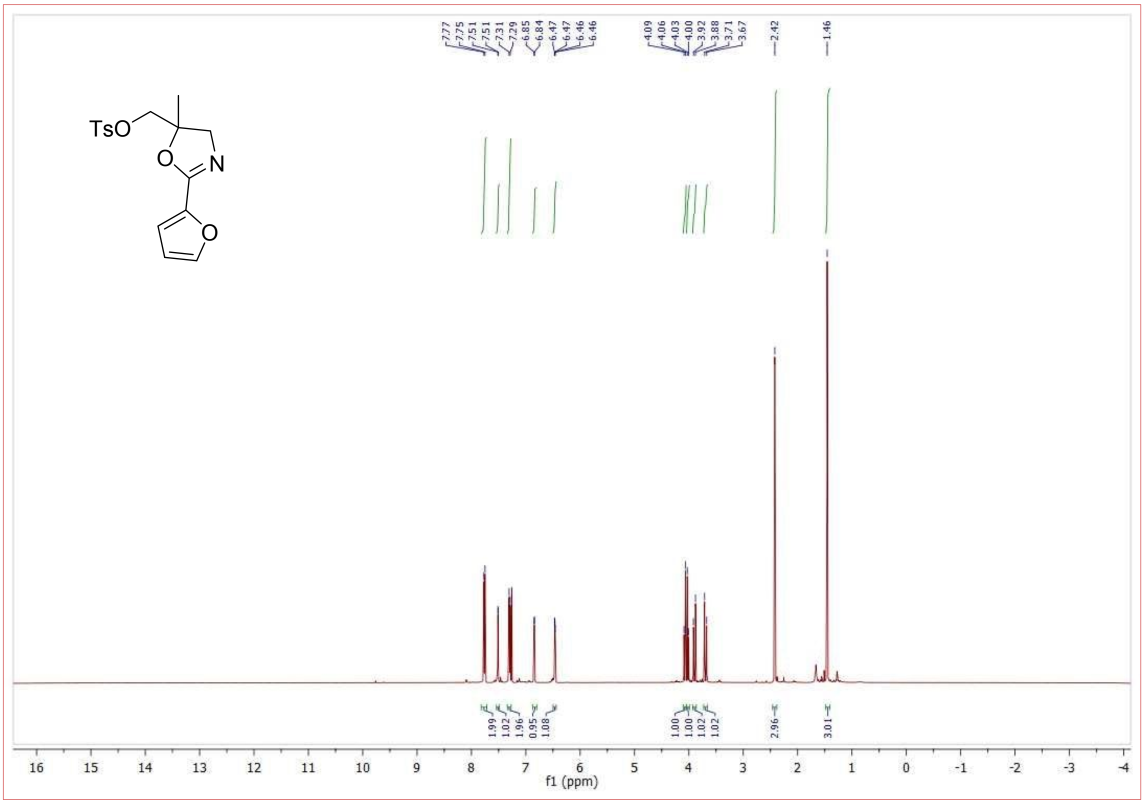

2-(Furan-2-yl)-5-methyl-4,5-dihydrooxazol-5-yl)methyl 4-methylbenzenesulfonate (2y), $^{13}C\{^{1}H\}$ NMR (101 MHz, $CDCl_3$)

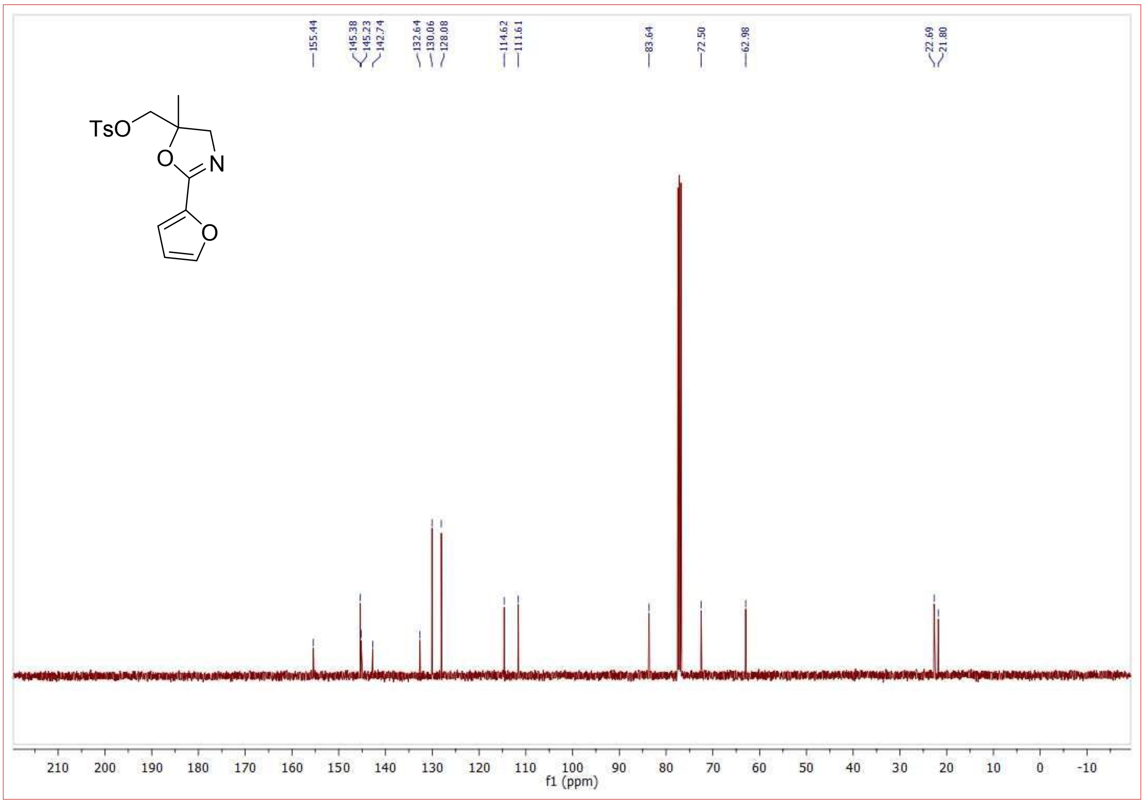

(2-(4-Chlorophenyl)-5,6-dihydro-4H-1,3-oxazin-6-yl)methyl 4-methylbenzenesulfonate (2z), ^{1}H NMR (400 MHz, CDCl$_3$)

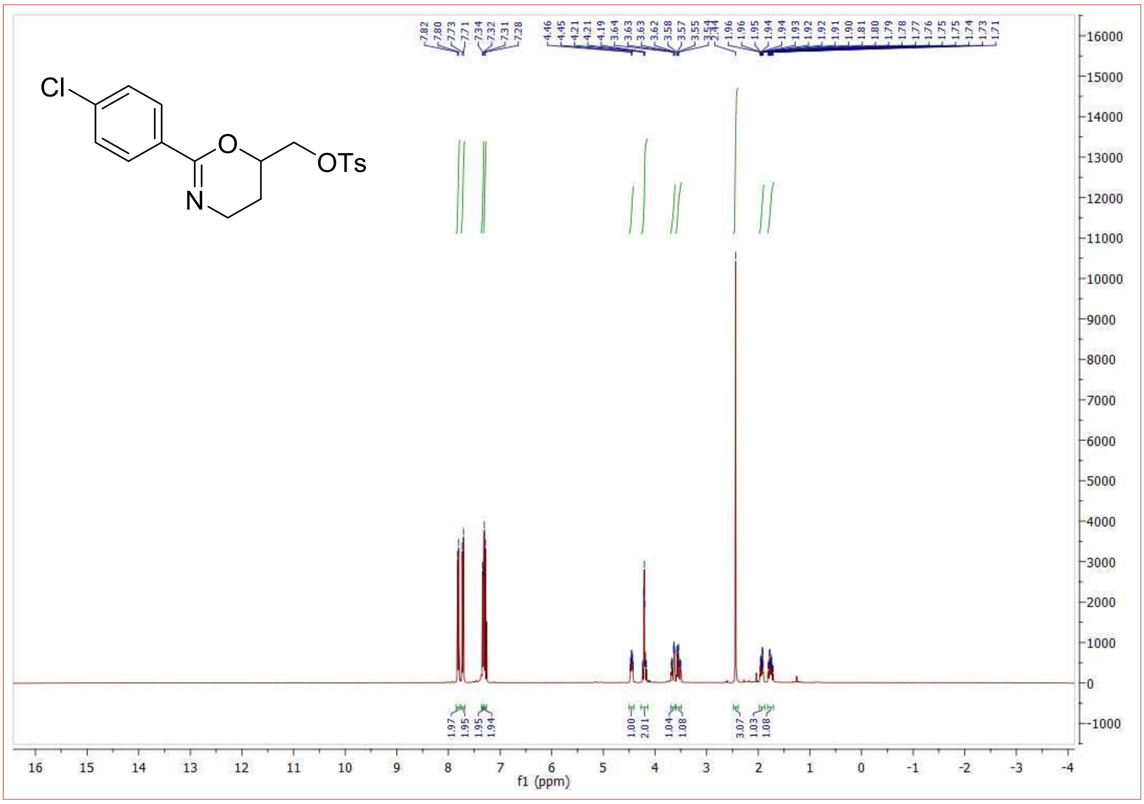

(2-(4-Chlorophenyl)-5,6-dihydro-4H-1,3-oxazin-6-yl)methyl 4-methylbenzenesulfonate (2z), ^{13}C{^{1}H} NMR (101 MHz, CDCl$_3$)

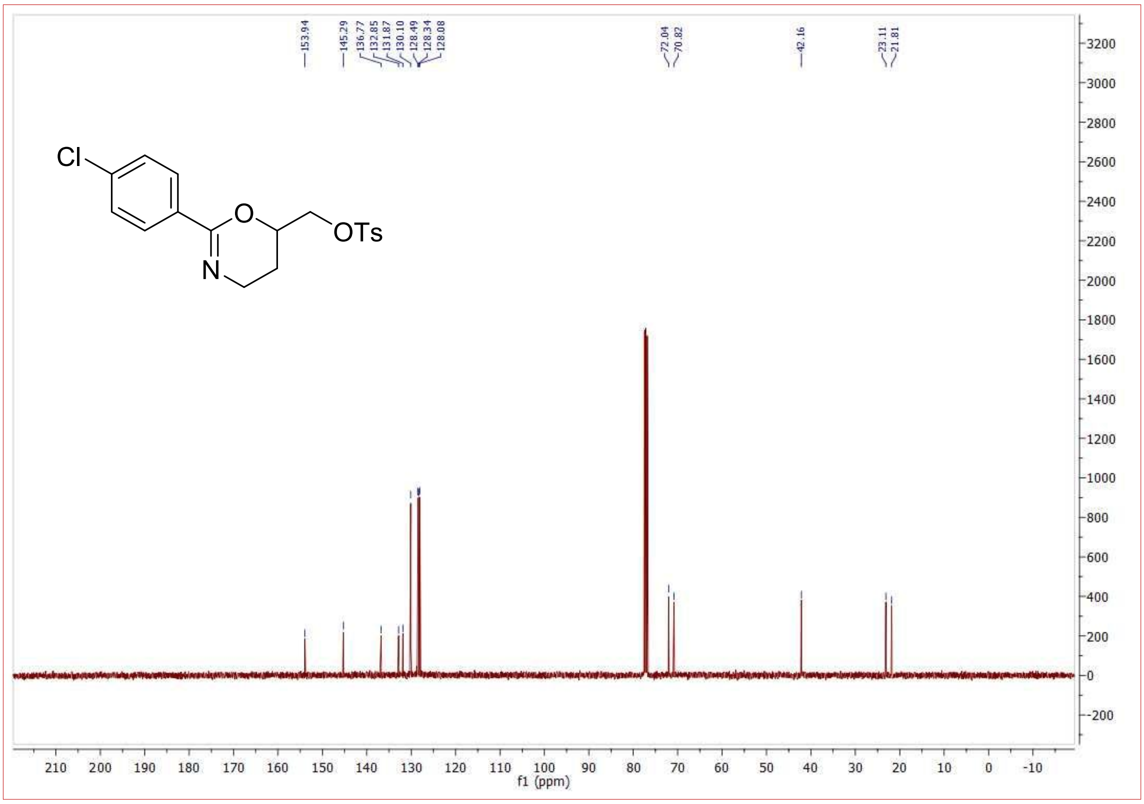

(2-(3-(Trifluoromethyl)phenyl)-5,6-dihydro-4H-1,3-oxazin-6-yl)methyl 4-methylbenzenesulfonate (2aa), ^{1}H NMR (400 MHz, $CDCl_3$)

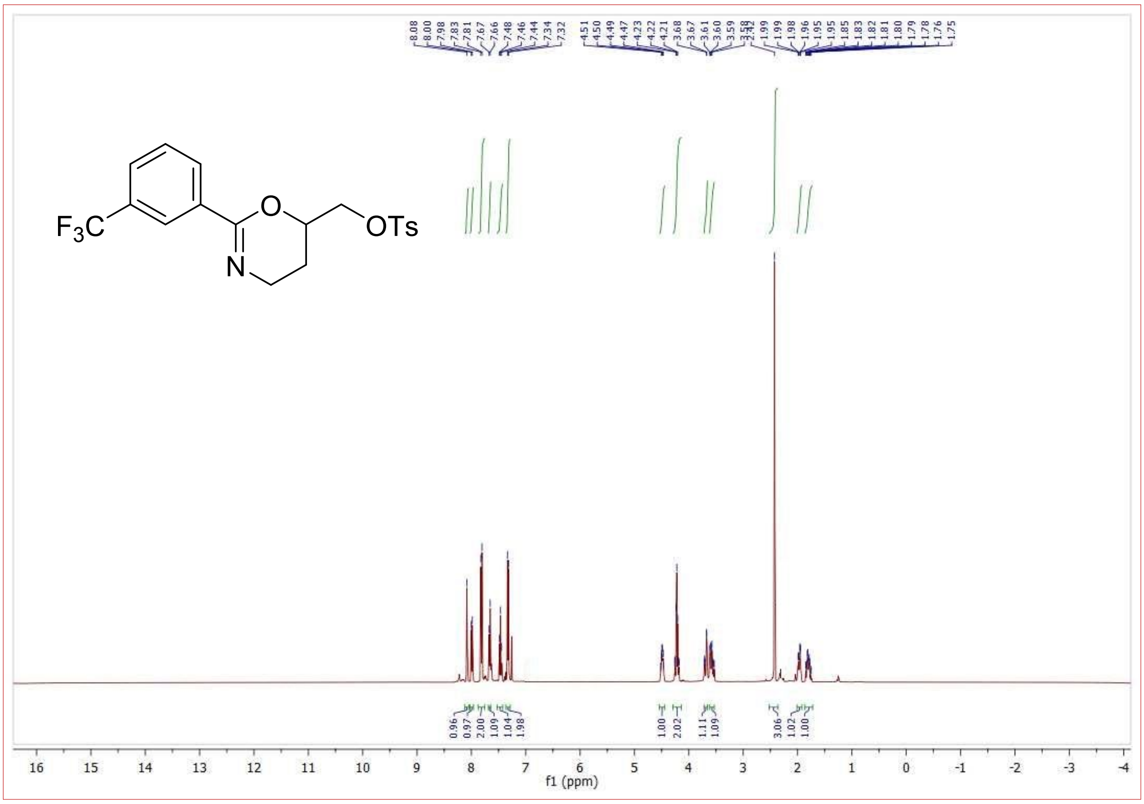

2-(3-(Trifluoromethyl)phenyl)-5,6-dihydro-4H-1,3-oxazin-6-yl)methyl 4-methylbenzenesulfonate (2aa), $^{13}C\{^{1}H\}$ NMR (101 MHz, $CDCl_3$)

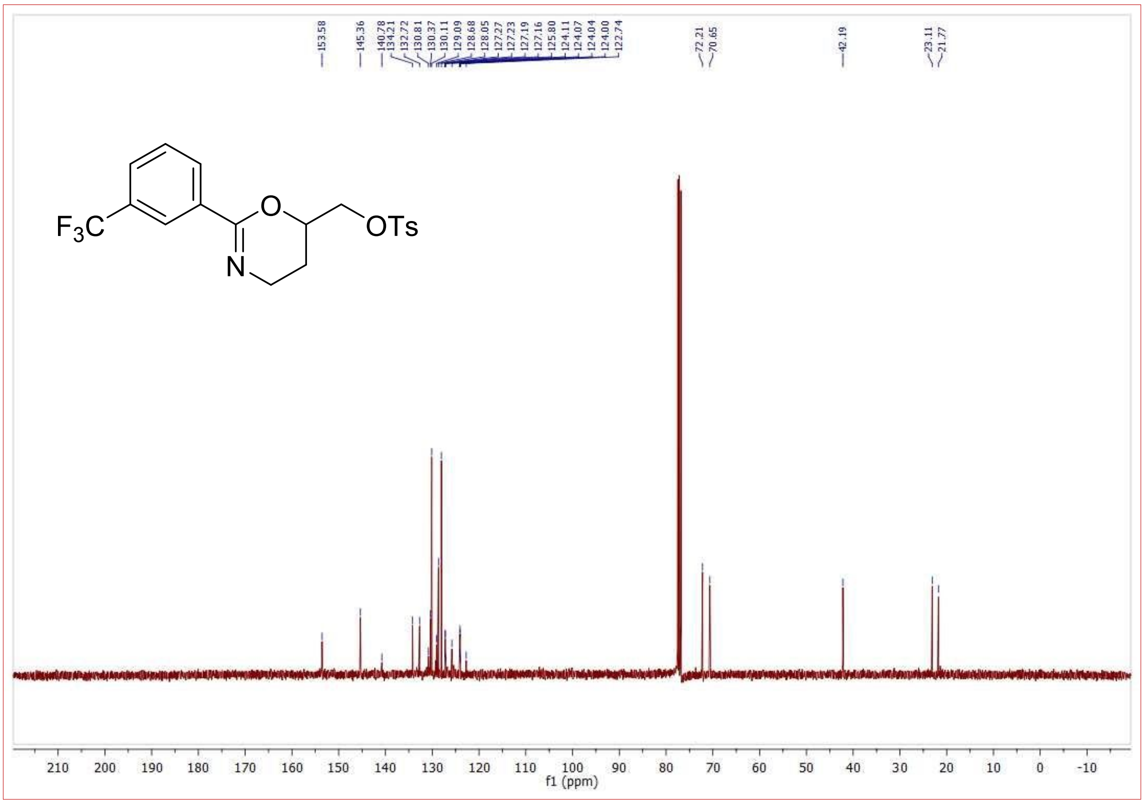

2-(3-(Trifluoromethyl)phenyl)-5,6-dihydro-4H-1,3-oxazin-6-yl)methyl 4-methylbenzenesulfonate (2aa), ^{19}F NMR (376 MHz, CDCl$_3$)

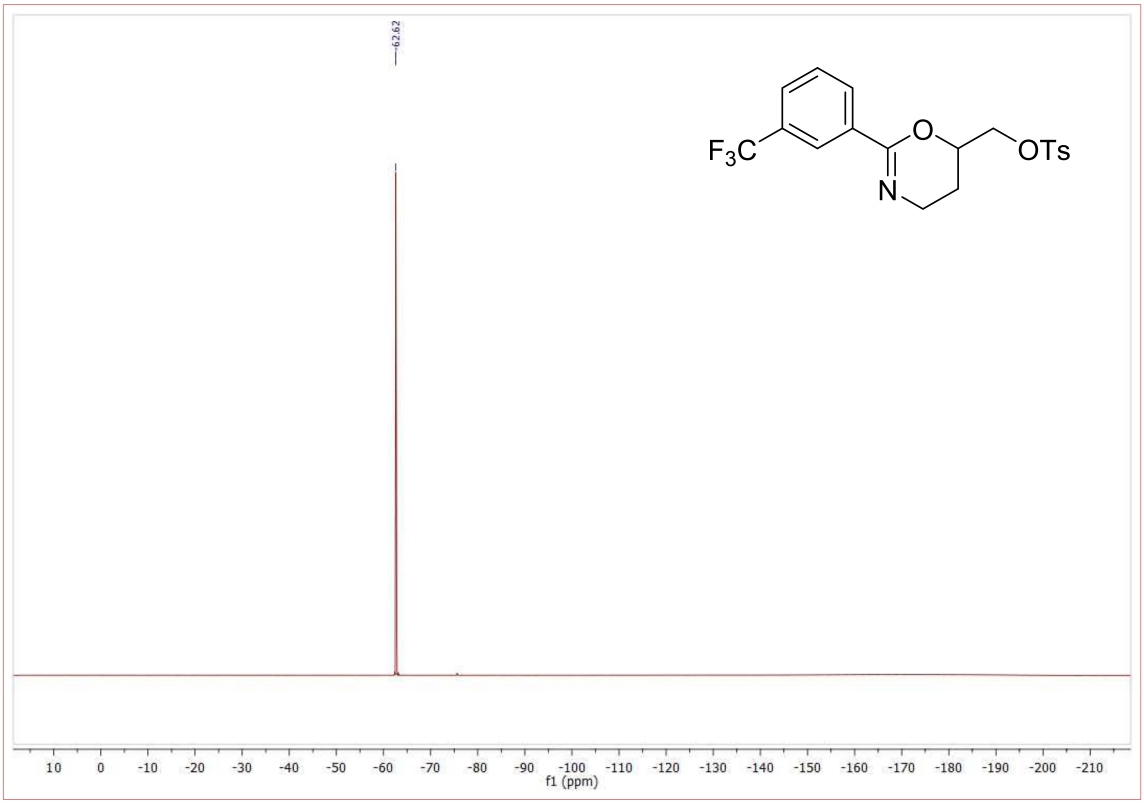

1-Oxo-1-phenylpropan-2-yl 4-methylbenzenesulfonate (4a), ^{1}H NMR (400 MHz, CDCl$_3$)

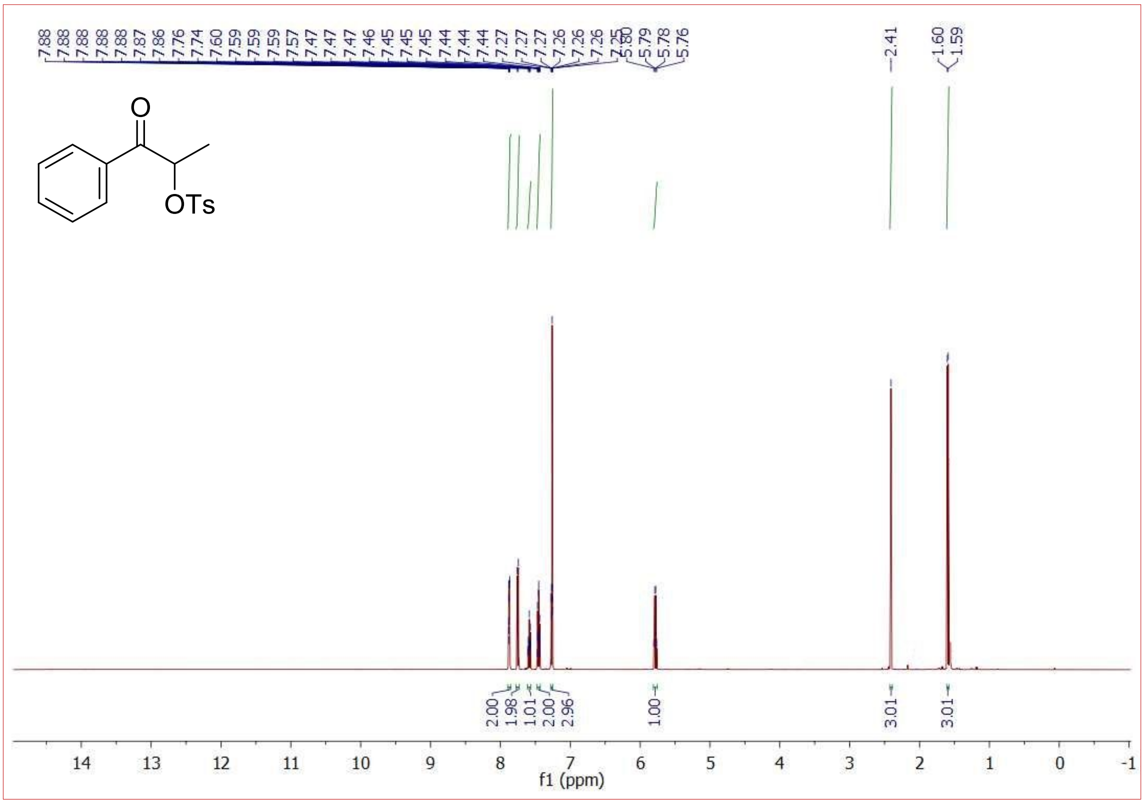

1-Oxo-1-phenylpropan-2-yl 4-methylbenzenesulfonate (4a), $^{13}C\{^{1}H\}$ NMR (101 MHz, $CDCl_3$)

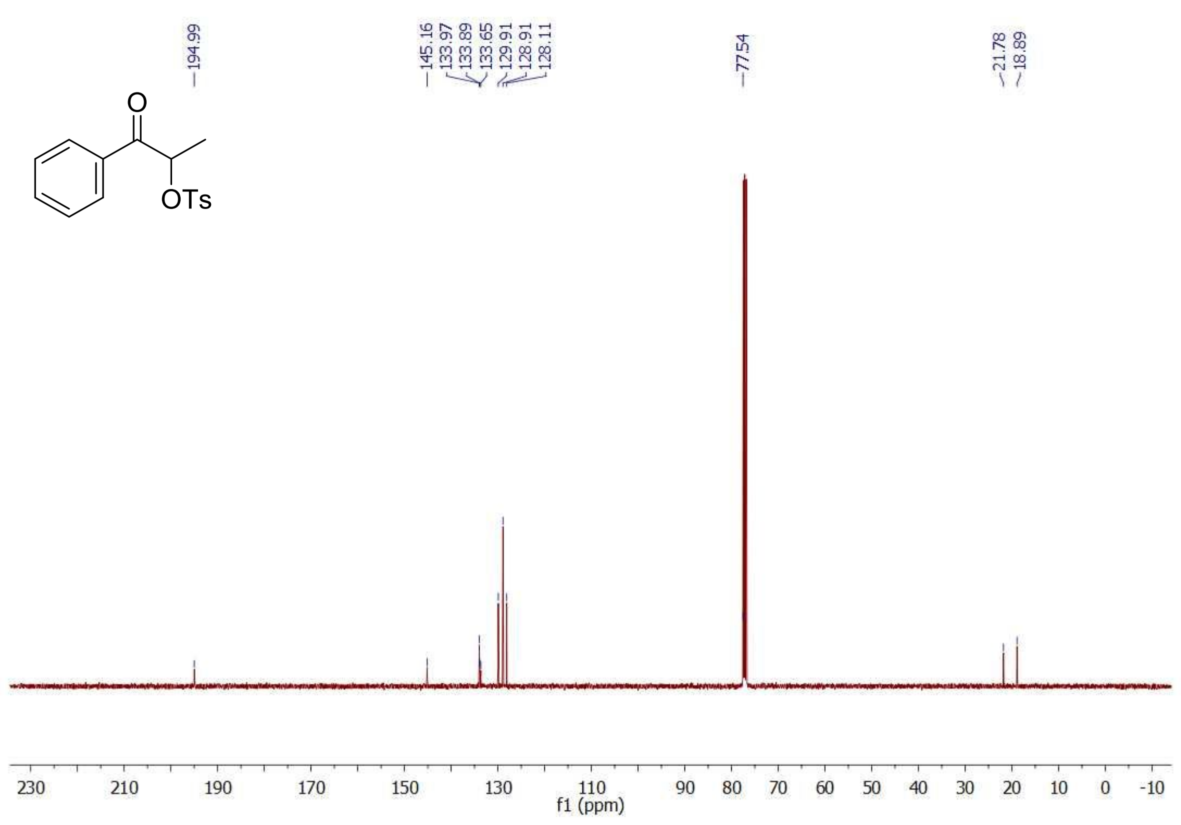

1-(3-Chlorophenyl)-1-oxopropan-2-yl 4-methylbenzenesulfonate (4b), ^{1}H NMR (400 MHz, $CDCl_3$)

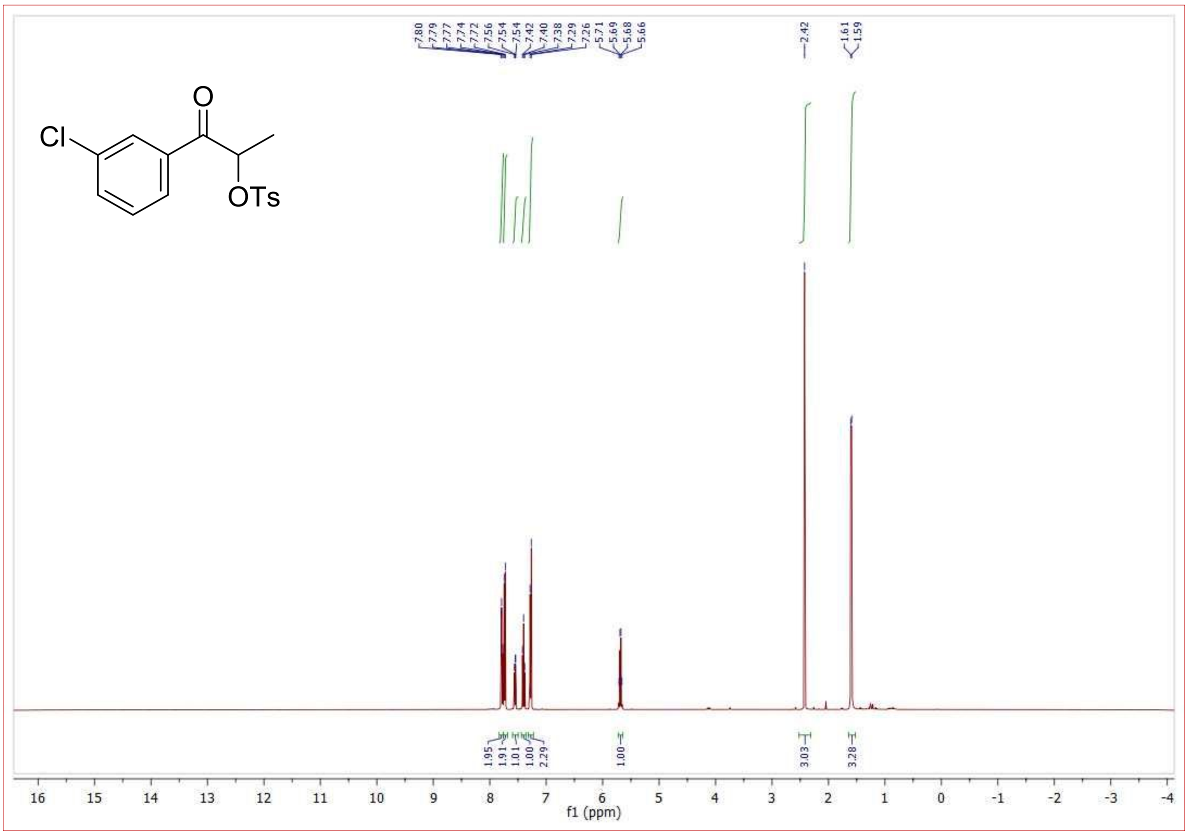

1-(3-Chlorophenyl)-1-oxopropan-2-yl 4-methylbenzenesulfonate (4b), $^{13}C\{^{1}H\}$ NMR (101 MHz, $CDCl_3$)

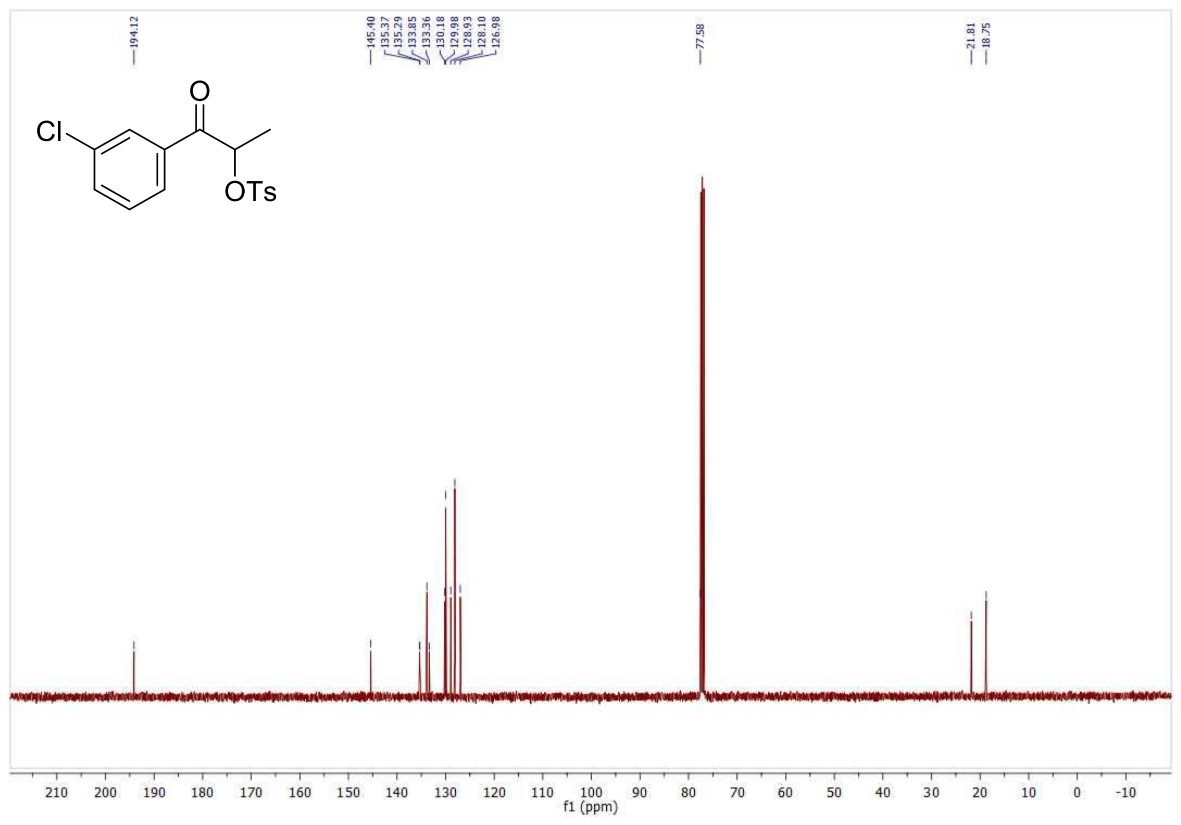

1-(4-Fluorophenyl)-1-oxopropan-2-yl 4-methylbenzenesulfonate (4c), ^{1}H NMR (400 MHz, $CDCl_3$)

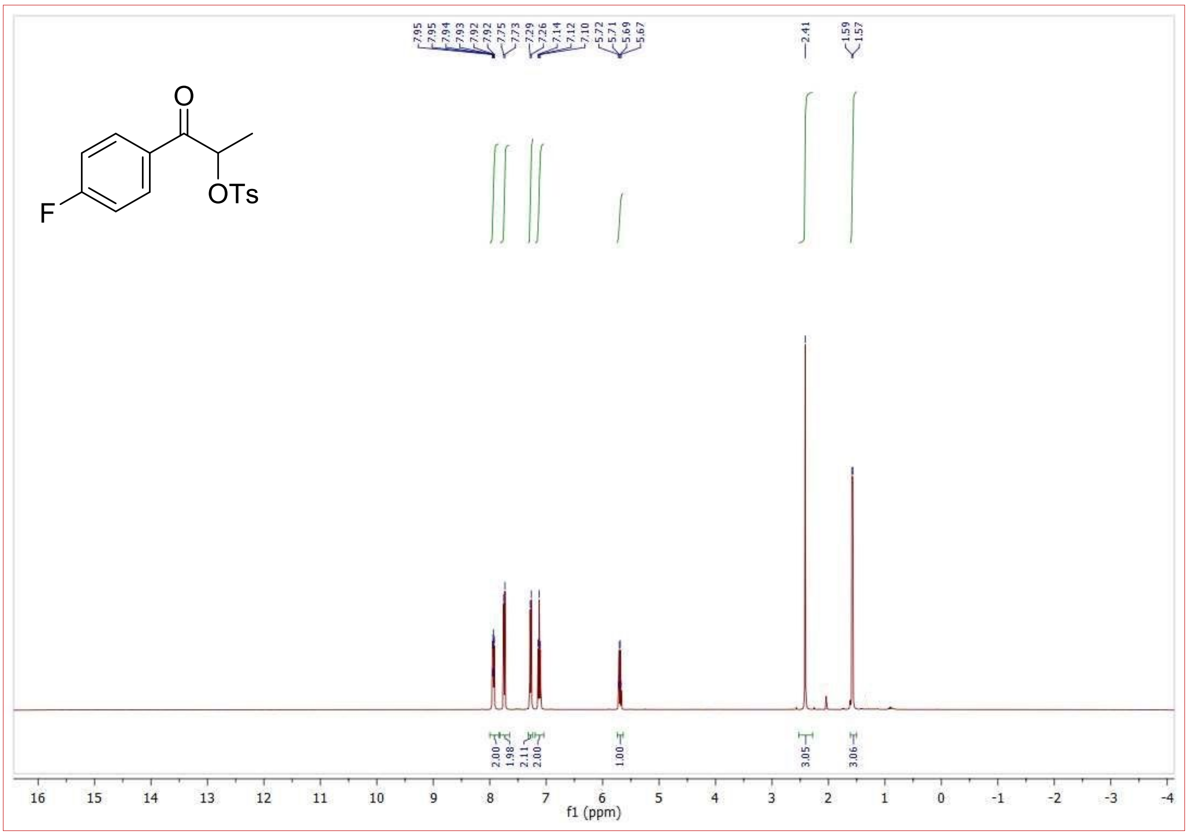

1-(4-Fluorophenyl)-1-oxopropan-2-yl 4-methylbenzenesulfonate (4c), $^{13}C\{^{1}H\}$ NMR (101 MHz, $CDCl_3$)

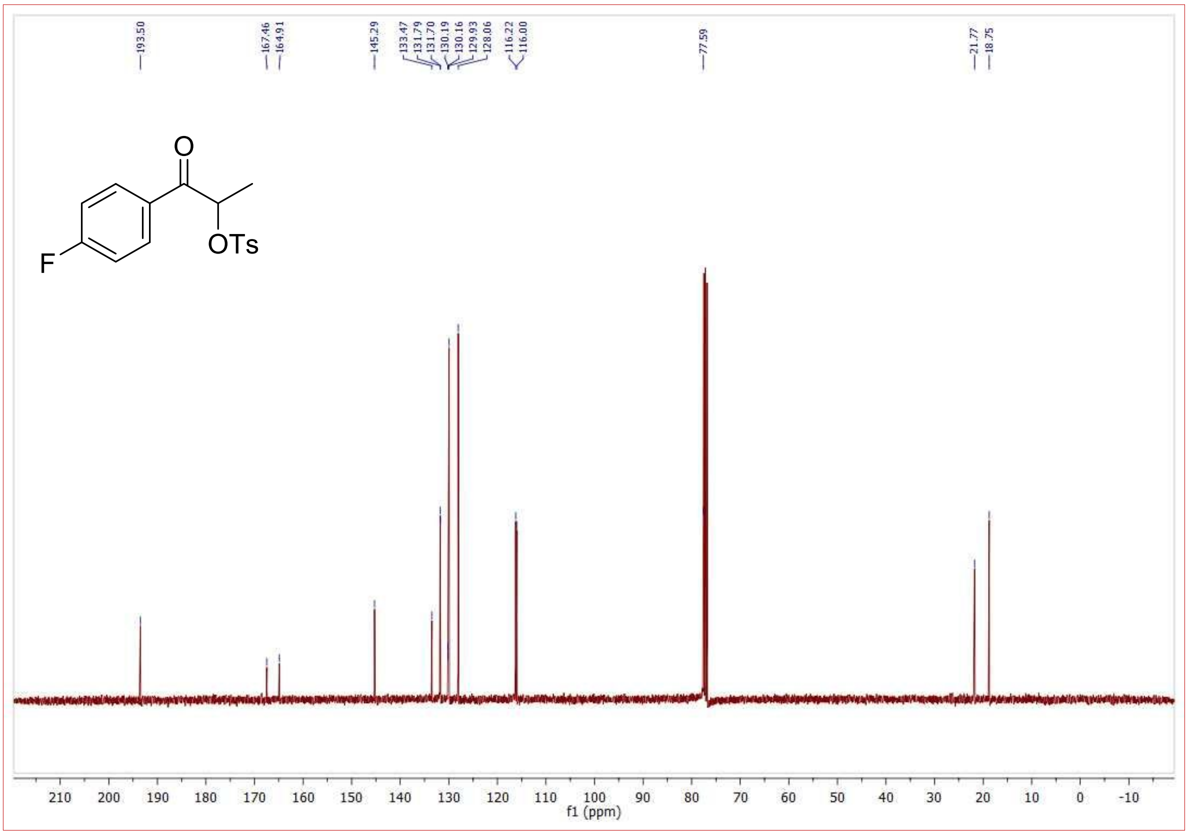

1-(4-Fluorophenyl)-1-oxopropan-2-yl 4-methylbenzenesulfonate (4c), ^{19}F NMR (376 MHz, $CDCl_3$)

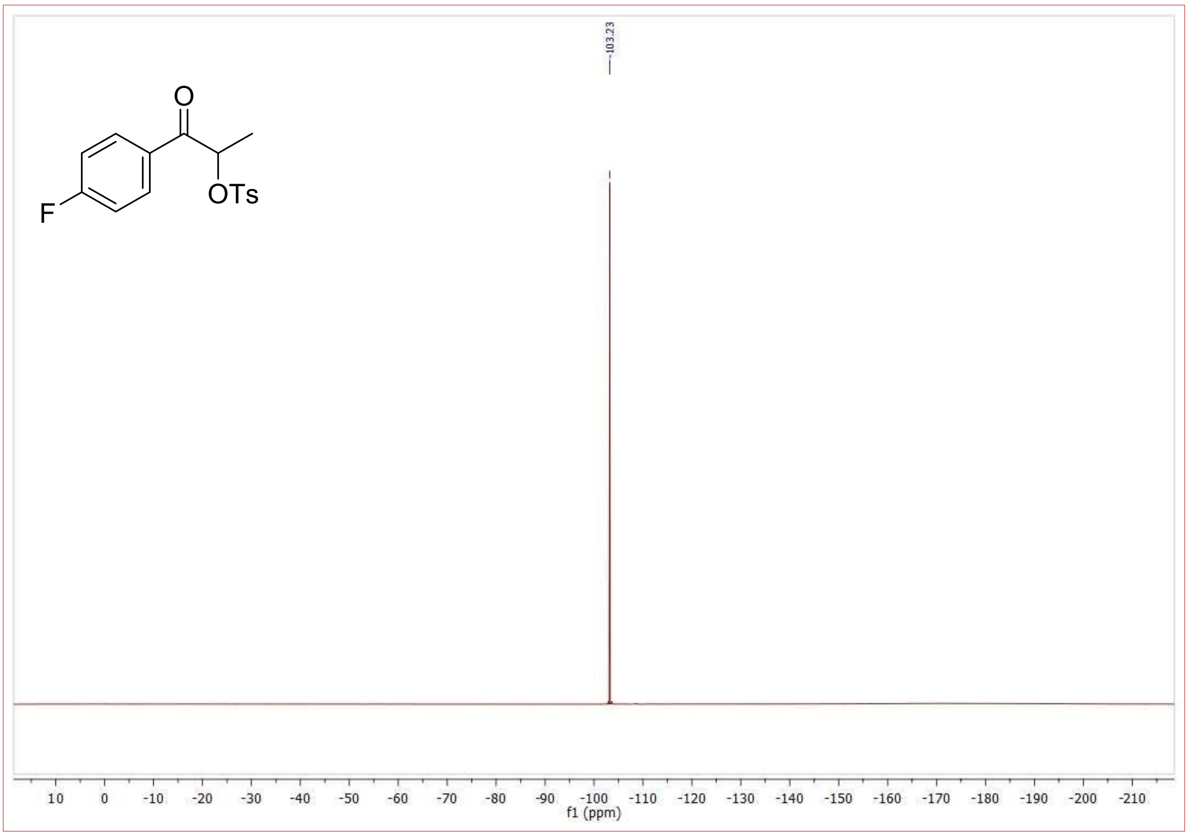

1-Oxo-1-(4-(trifluoromethyl)phenyl)propan-2-yl 4-methylbenzenesulfonate (4d), ^{1}H NMR (400 MHz, CDCl$_3$)

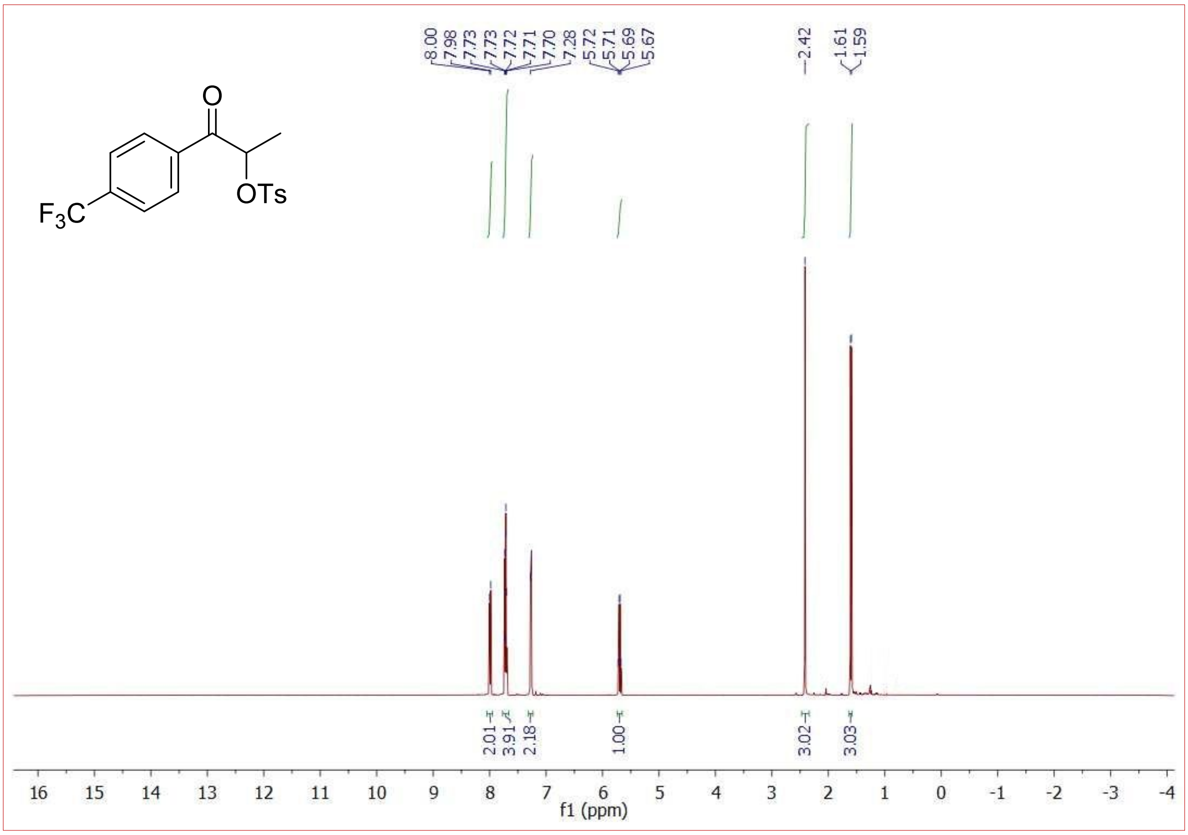

1-Oxo-1-(4-(trifluoromethyl)phenyl)propan-2-yl 4-methylbenzenesulfonate (4d), ^{13}C{^{1}H} NMR (101 MHz, CDCl$_3$)

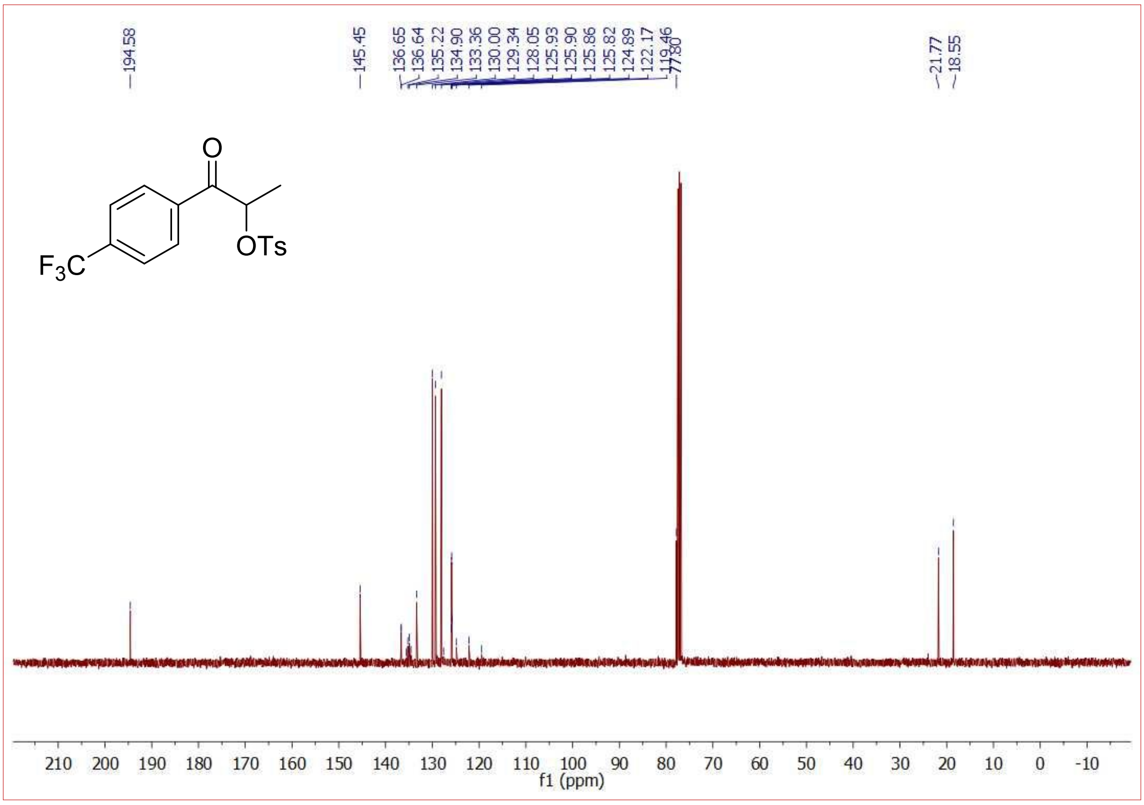

1-Oxo-1-(4-(trifluoromethyl)phenyl)propan-2-yl 4-methylbenzenesulfonate (4d), ^{19}F NMR (376 MHz, $CDCl_3$)

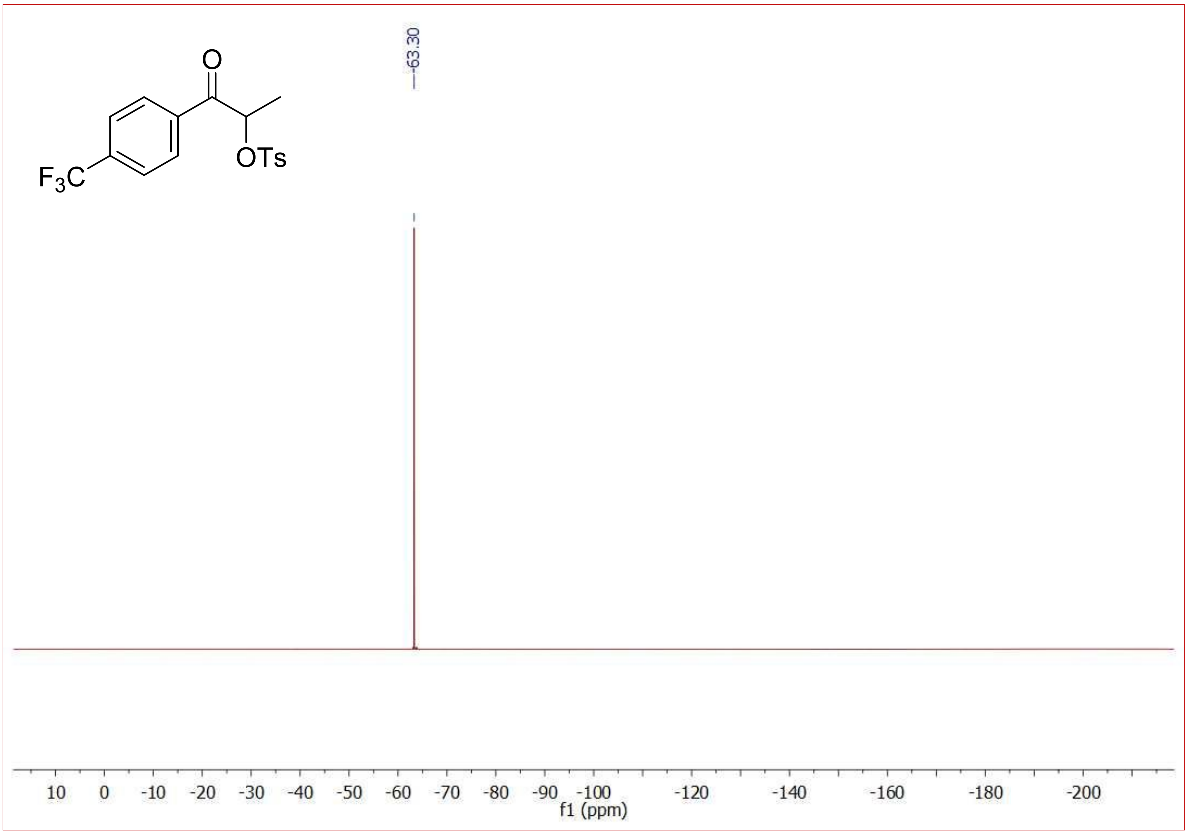

1-Oxo-1-(2-(trifluoromethyl)phenyl)propan-2-yl 4-methylbenzenesulfonate (4e), ^{1}H NMR (400 MHz, $CDCl_3$)

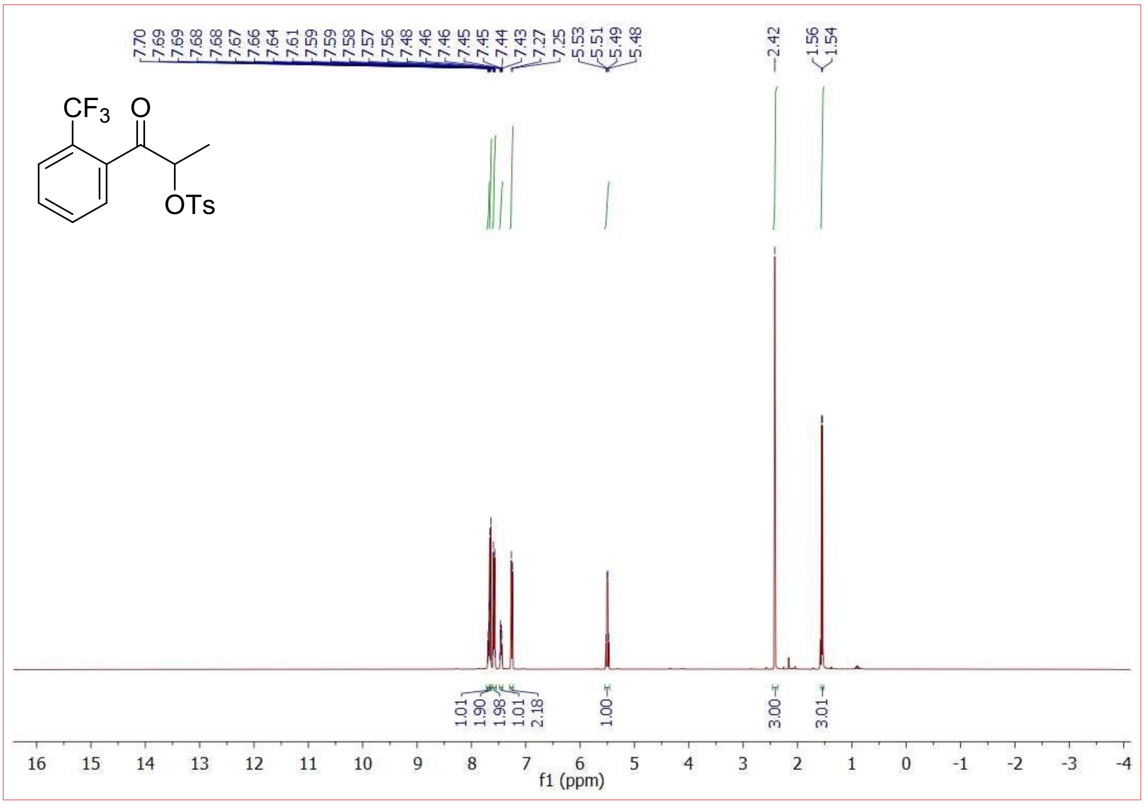

1-Oxo-1-(2-(trifluoromethyl)phenyl)propan-2-yl 4-methylbenzenesulfonate (4e), $^{13}C\{^1H\}$ NMR (101 MHz, CDCl$_3$)

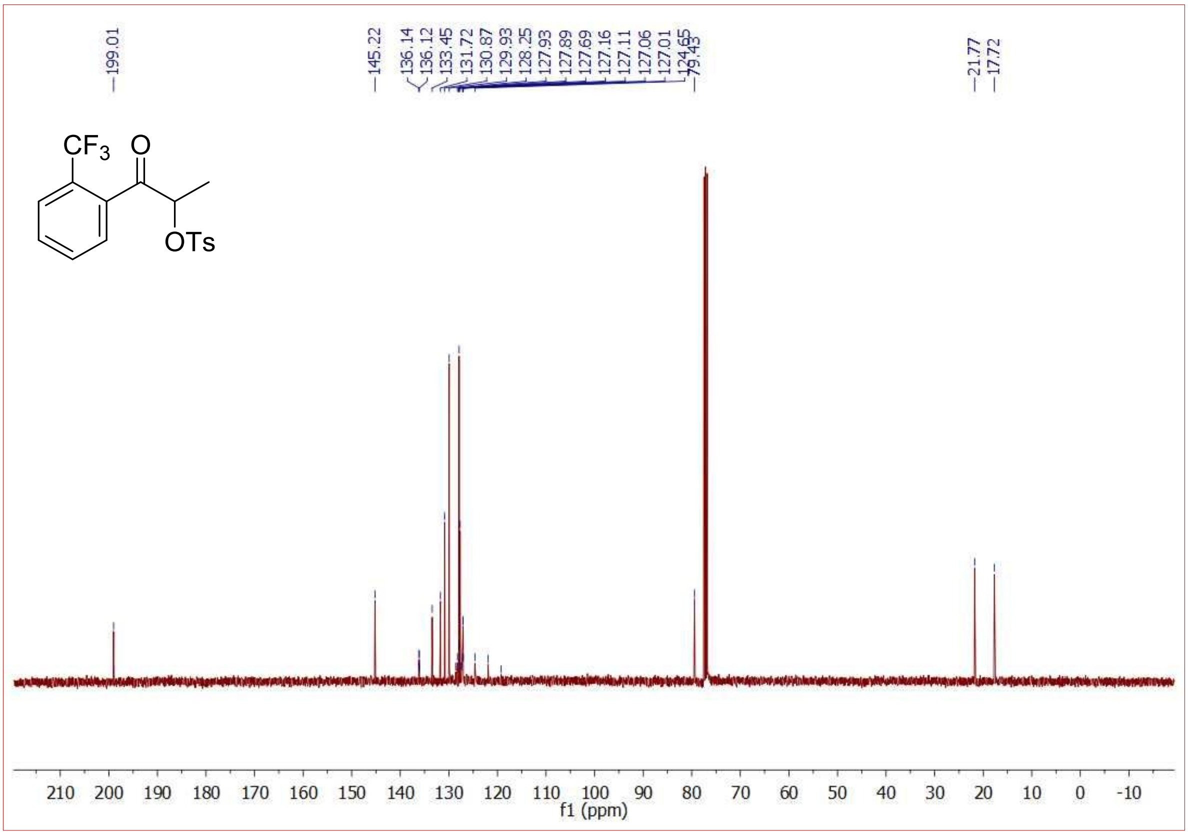

1-Oxo-1-(2-(trifluoromethyl)phenyl)propan-2-yl 4-methylbenzenesulfonate (4e), ^{19}F NMR (376 MHz, CDCl$_3$)

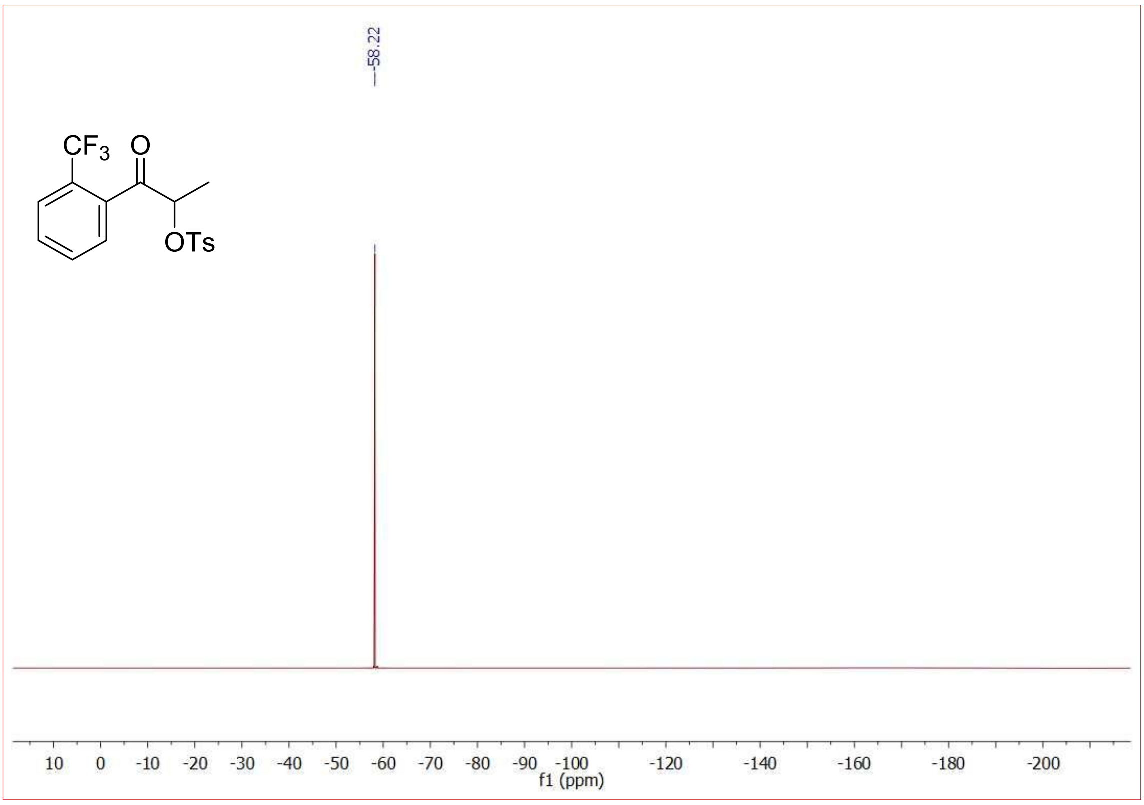

1-(2,3-Difluorophenyl)-1-oxopropan-2-yl 4-methylbenzenesulfonate (4f), ^{1}H NMR (400 MHz, CDCl$_3$)

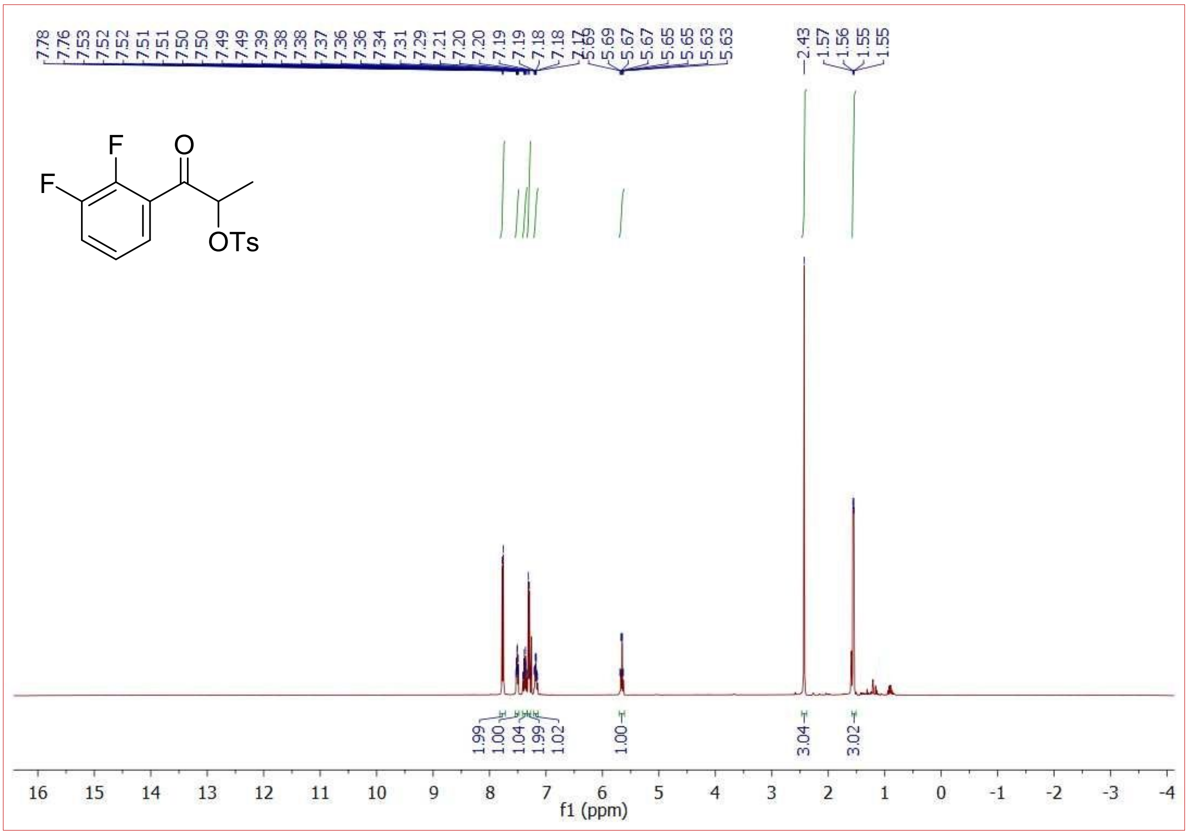

1-(2,3-Difluorophenyl)-1-oxopropan-2-yl 4-methylbenzenesulfonate (4f), ^{13}C{^{1}H} NMR (101 MHz, CDCl$_3$)

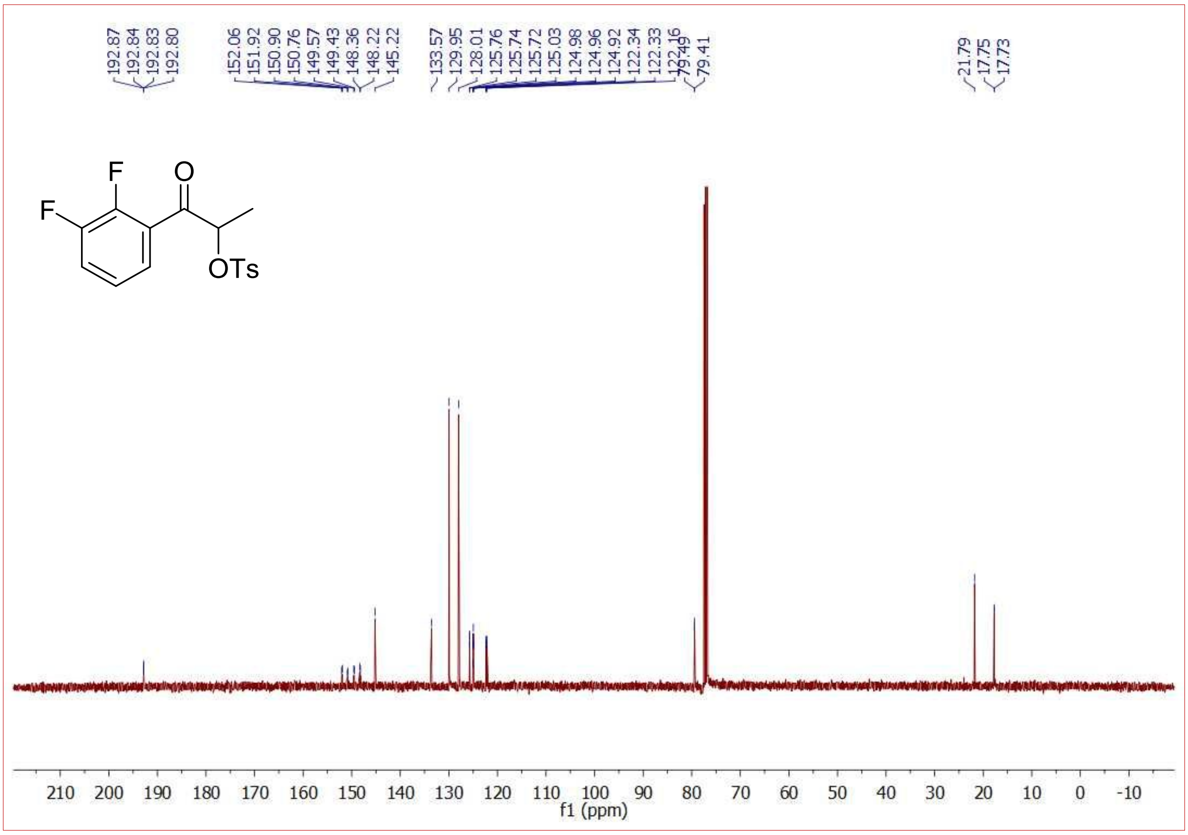

1-(2,3-Difluorophenyl)-1-oxopropan-2-yl 4-methylbenzenesulfonate (4f), ^{19}F NMR (376 MHz, CDCl$_3$)

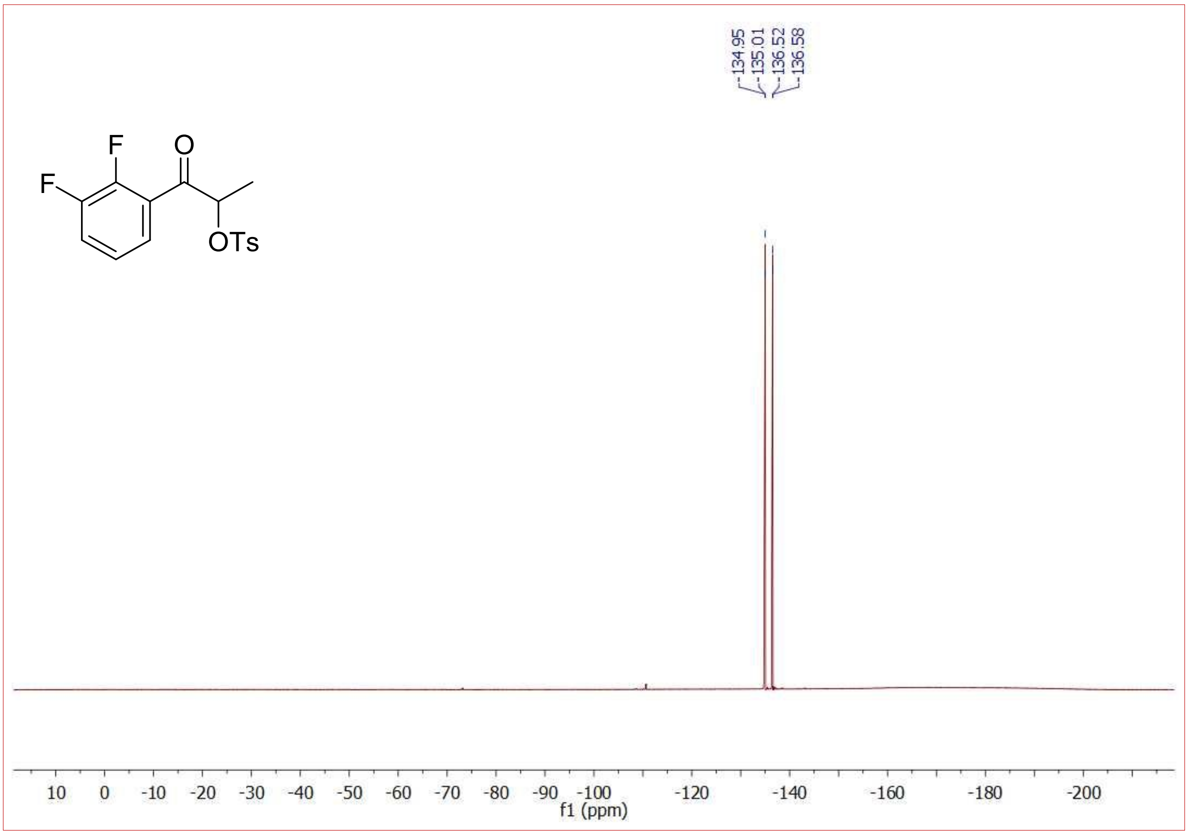

1-Oxo-1-phenylbutan-2-yl 4-methylbenzenesulfonate (4g), ^{1}H NMR (400 MHz, CDCl$_3$)

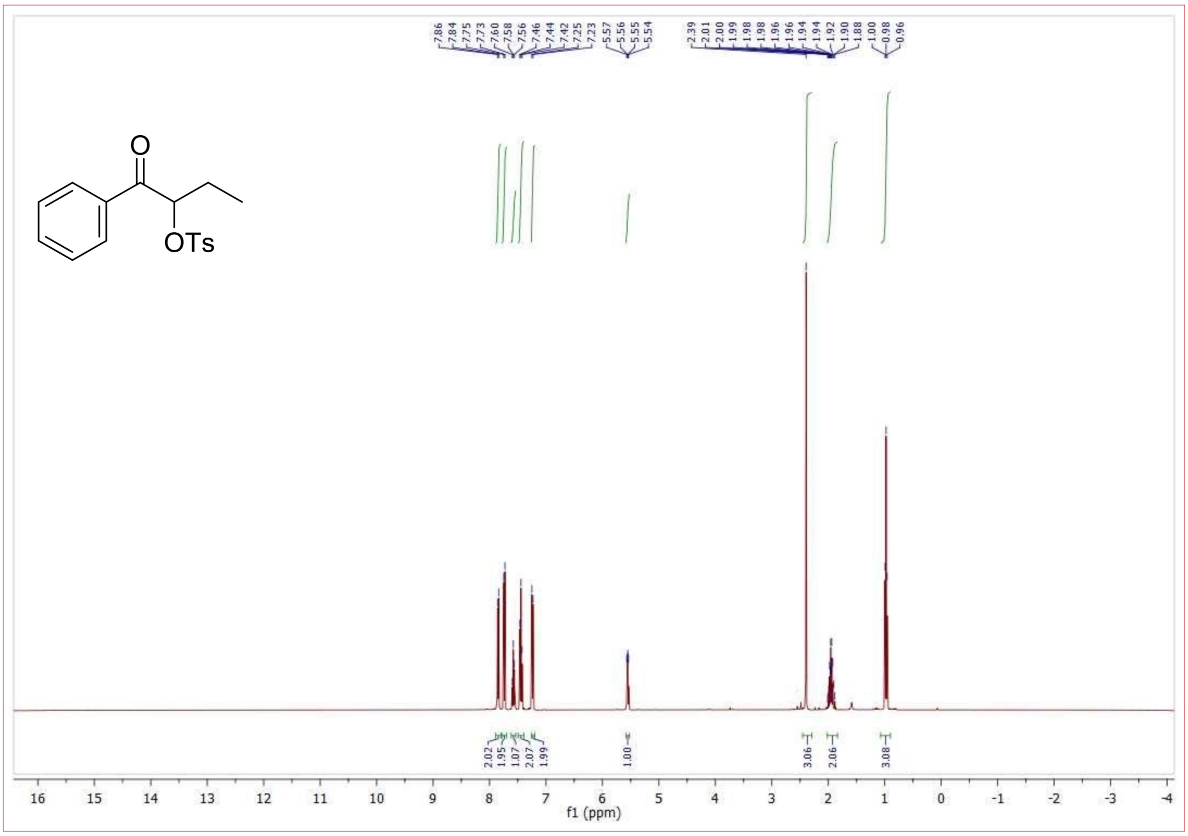

1-Oxo-1-phenylbutan-2-yl 4-methylbenzenesulfonate (4g), $^{13}C\{^{1}H\}$ NMR (101 MHz, $CDCl_3$)

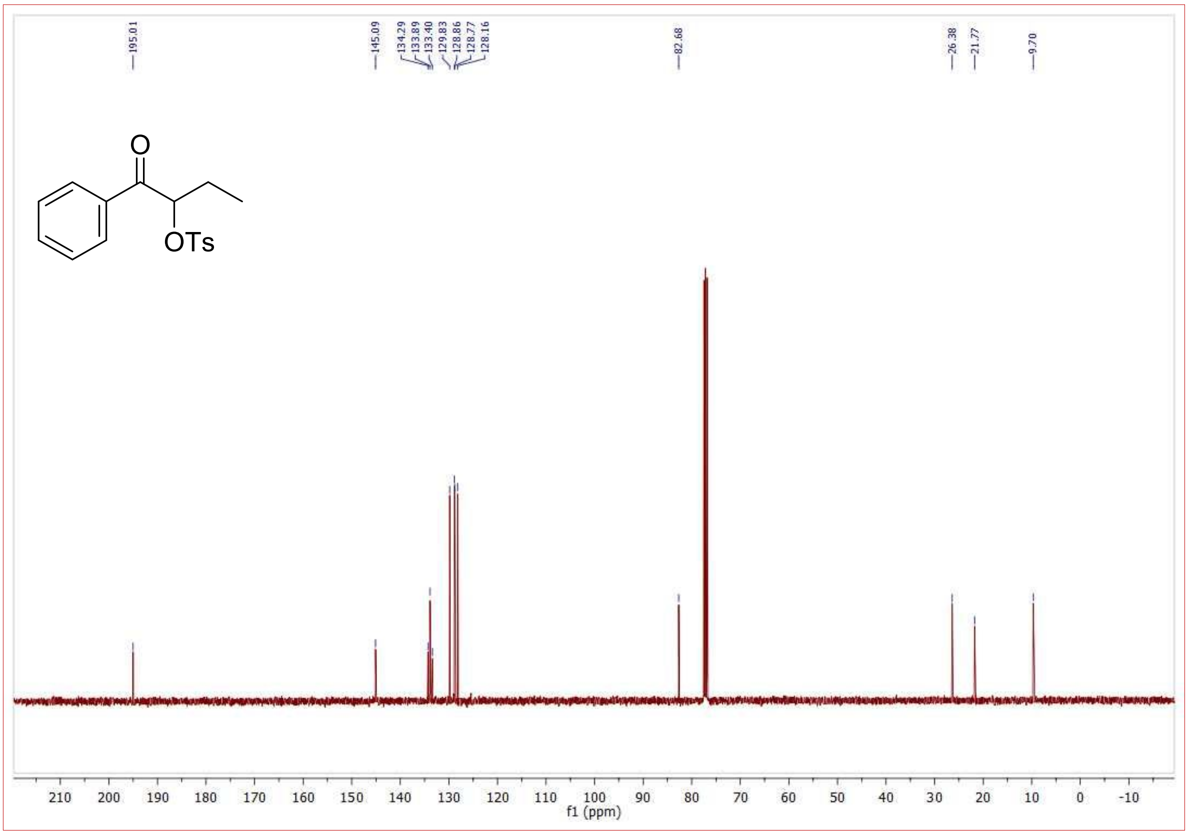

Supplement: Supplementary file 1 — jo2c02309_si_001.pdf [file jo2c02309_si_001.pdf]
